# Dehydrocoupling of phosphine–boranes using the [RhCp*Me(PMe_3_)(CH_2_Cl_2_)][BAr^F^_4_] precatalyst: stoichiometric and catalytic studies†
†Electronic supplementary information (ESI) available: Synthesis and characterisation data, computational details. CCDC 1423368–1423370. For ESI and crystallographic data in CIF or other electronic format see DOI: 10.1039/c5sc04150c


**DOI:** 10.1039/c5sc04150c

**Published:** 2015-12-21

**Authors:** Thomas N. Hooper, Andrew S. Weller, Nicholas A. Beattie, Stuart A. Macgregor

**Affiliations:** a Department of Chemistry , Chemistry Research Laboratories , University of Oxford , Mansfield Road , Oxford , OX1 3TA , UK . Email: andrew.weller@chem.ox.ac.uk; b Institute of Chemical Sciences , Heriot Watt University , Edinburgh , EH14 4AS , UK . Email: S.A.Macgregor@hw.ac.uk

## Abstract

We report a detailed, combined experimental and computational study on the fundamental B–H and P–H bond activation steps involved in the dehydrocoupling/dehydropolymerization of primary and secondary phosphine–boranes, H_3_B·PPhR′H (R = Ph, H), using [RhCp*(PMe_3_)Me(ClCH_2_Cl)][BAr^F^_4_], to either form polyphosphino-boranes [H_2_B·PPhH]_*n*_ (*M*_n_ ∼ 15 000 g mol^–1^, PDI = 2.2) or the linear diboraphosphine H_3_B·PPh_2_BH_2_·PPh_2_H. A likely polymer-growth pathway of reversible chain transfer step-growth is suggested for H_3_B·PPhH_2_. Using secondary phosphine–boranes as model substrates a combined synthesis, structural (X-ray crystallography), labelling and computational approach reveals: initial bond activation pathways (B–H activation precedes P–H activation); key intermediates (phosphido-boranes, α-B-agostic base-stabilized boryls); and a catalytic route to the primary diboraphosphine (H_3_B·PPhHBH_2_·PPhH_2_). It is also shown that by changing the substituent at phosphorus (Ph or Cy *versus*^*t*^Bu) different final products result (phosphido-borane or base stabilized phosphino-borane respectively). These studies provide detailed insight into the pathways that are operating during dehydropolymerization.

## Abstract 2

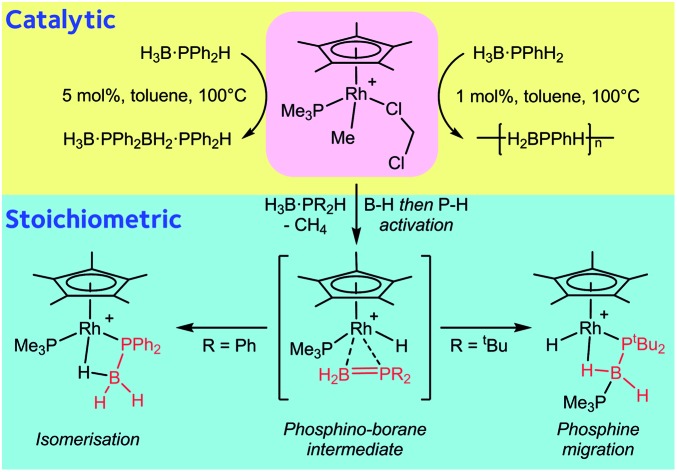
Detailed experimental and computational studies are reported on the fundamental B–H and P–H bond activation steps involved in the dehydrocoupling/dehydropolymerization of primary and secondary phosphine–boranes, H_3_B·PPhR′H (R = Ph, H), using the [RhCp*(PMe_3_)Me(ClCH_2_Cl)][BAr^F^_4_] catalyst.

## Introduction

**Scheme 1:**
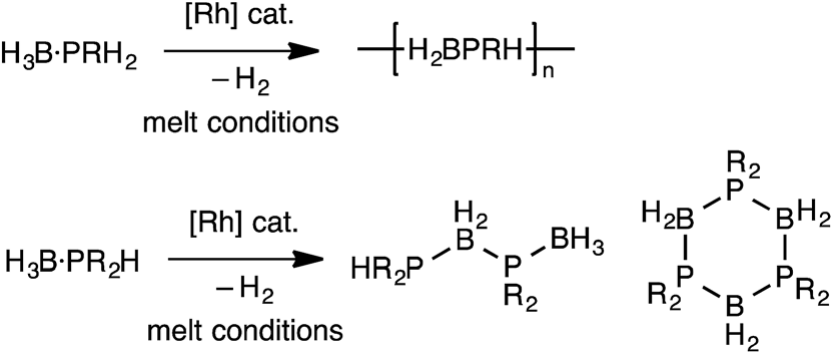
Rh-catalyzed dehydrocoupling of primary and secondary phosphine–boranes.

The polymerization of alkenes using transition metal-based catalysts to afford societally and technologically ubiquitous polyolefins is well-established, yet equivalent catalytic routes to polymeric materials containing main-group elements is considerably less developed.^1^,^2^ In particular, the group 13/15 mixed polymers provide one example that promises to lead to significant scientific and technological opportunities, given that polyphosphino-boranes, along with polyamino-boranes,^3^ are (valence) isoelectronic with polyolefins and are finding uses in a variety of applications from lithography to pre-ceramics.^4^,^5^ Ill-defined polyphosphino-boranes were first synthesised in 1959 through thermal dehydrocoupling of primary phosphine–boranes,^6^ but a faster and more selective dehydrocoupling/dehydropolymerization process was reported by Manners and co-workers in the early 2000's using transition metal pre-catalysts primarily based upon [Rh(COD)Cl]_2_ and [Rh(COD)_2_][OTf], operating under melt conditions.^7^–^10^ Others have since used similar catalyst systems to prepare related polyphosphino-boranes, or elegant demonstrations of highly selective cross-dehydrocouplings.^11^,^12^ For primary phosphine–boranes, H_3_B·PRH_2_, polyphosphino-boranes are formed, whereas for secondary phosphine–boranes, H_3_B·PR_2_H, linear diboraphosphines or cyclic oligomers form (Scheme 1). Although catalysis has been shown to be homogenous rather than heterogeneous,^13^,^14^ the melt conditions required for effective dehydrocoupling meant that resolving intermediates/resting states or kinetics was challenging. In contrast, the mechanism of amine–borane dehydrocoupling using transition metal catalysts is much better understood as catalysis can be performed in solution at room temperature.^15^ Very recently the non-metal-catalyzed addition polymerization of *in situ* generated phosphino-boranes, such as [H_2_BP^*t*^Bu_2_], has been described,^16^ that avoids the use of melt conditions.

**Scheme 2:**
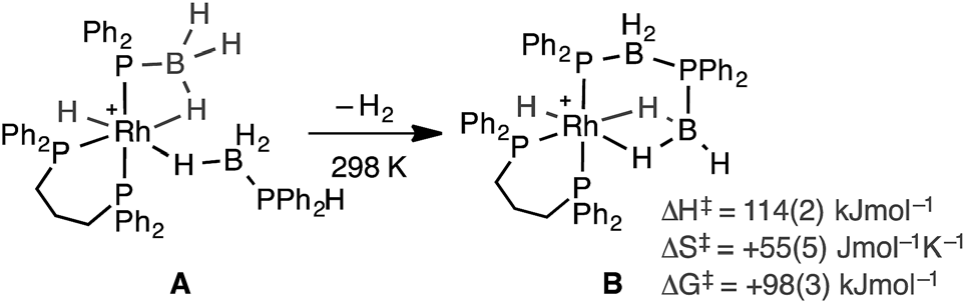
Intermediates observed in the dehydrocoupling of H_3_B·PPh_2_H using the [Rh(Ph_2_P(CH_2_)_3_PPh_2_)]^+^ fragment. [BAr^F^_4_]^–^ anions not shown.

Recently, *in situ* sampling using ESI-MS (electrospray ionisation mass spectrometry) led to the identification of a [Rh(PHR_2_)_2_]^+^ fragment as an active species in the dehydrocoupling of secondary phosphine–boranes under melt conditions to form H_3_B·PR_2_H_2_B·PHR_2_ when using [Rh(COD)_2_][BAr^F^_4_] as the precatalyst [R = Ph, ^*t*^Bu; Ar^F^ = 3,5-(CF_3_)_2_C_6_H_3_].^17^ This arises from cleavage of the relatively weak P–B bond in the substrate.^18^ Simple replacement of the monodentate phosphine ligands with a bidentate phosphine produced a metal fragment, *i.e.* [Rh(Ph_2_P(CH_2_)_3_PPh_2_)]^+^, which did not suffer from ligand redistribution, allowing for a detailed study of the mechanism, including isolation of intermediates, isotopic labelling studies and determination of activation parameters.^19^,^20^ Thus intermediate complexes that relate to overall P–H activation of H_3_B·PPh_2_H at a Rh(i) center (**A**Scheme 2), and subsequent P–B bond formation (**B**), were isolated, while B–H activation of the second phosphine–borane to form a boryl intermediate was proposed to be involved in the rate-determining step that follows from **A**. However, because of relatively rapid H/D exchange between P and B the elementary P–H/B–H activation steps could not be delineated using labelling studies. In addition, although this dehydrocoupling occurred at room temperature, melt conditions were required for turnover. This same fragment was also found to dehydrocouple primary phosphine–boranes under melt conditions to produce ill-defined low molecular weight polymer. The mechanism was proposed to be the same as with secondary phosphine–boranes, but with the added complexity of diastereomer formation caused by P–H activation of the prochiral phosphorus centre.^20^

**Scheme 3:**
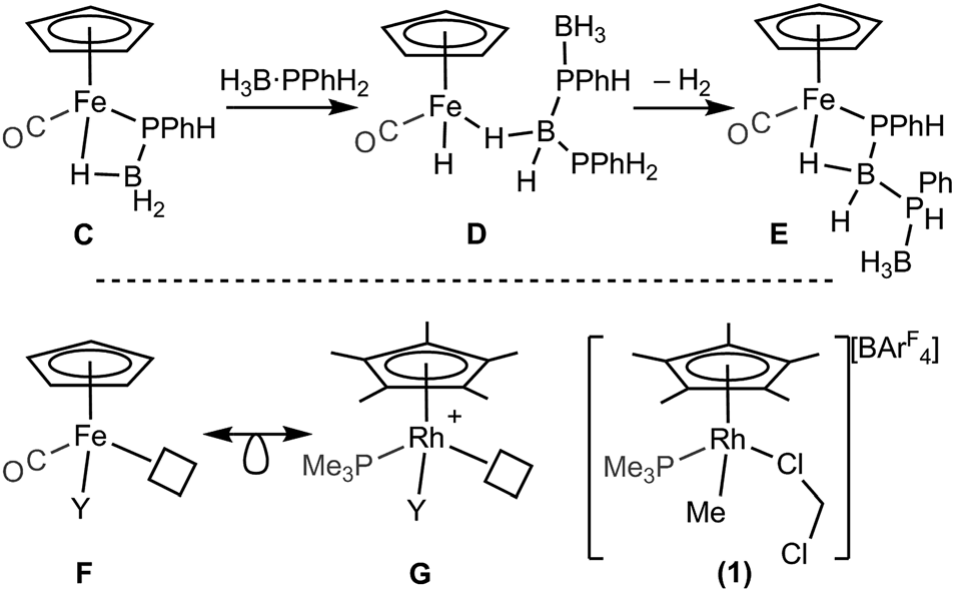
(Top) Intermediates (isolated and suggested) in the dehydropolymerization of H_3_B·PPhH_2_ as catalysed by FeCp(CO)_2_OTf. (Bottom) Relationship between FeCp(CO)Y and [RhCp*(PMe_3_)Y]^+^ (Y = anionic ligand).

A catalytic system which does not require melt conditions, produces well-defined, high molecular weight polyphosphino-borane (*M*_n_ = 59 000 g mol^–1^, PDI = 1.6) and operates *via* a chain growth process was reported in 2015 by Manners *et al.* using the FeCp(CO)_2_(OTf) catalyst.^5^ Heating (toluene, 100 °C) in the presence of phosphine–borane was required to promote CO and [OTf]^–^ loss and the formation of an initial phosphido-borane complex (**C**, Scheme 3, isolated for the H_3_B·PPh_2_ analogue). In the mechanism it was suggested that the Fe centre adopts a constant oxidation state with B–H/P–H activation and P–B coupling proposed (**D** and **E**), using DFT calculations, to proceed *via* multiple sigma-complex assisted metathesis steps.^21^,^22^

Central to control of the dehydropolymerization process is a detailed understanding of the fundamental, elementary, steps that are occurring. Inspired by this recent report by Manners on the FeCp(CO)_2_(OTf) system, and also aware that this system still required heating to promote CO loss, we turned to [RhCp*Me(PMe_3_)(CH_2_Cl_2_)][BAr^F^_4_] (**1**, Scheme 3, Cp* = η^5^-C_5_Me_5_)^23^,^24^ as an alternative entry point (*cf.* structures **F** and **G**), proposing that B–H/P–H activation may be studied at ambient temperature under solution conditions. This complex provides a latent vacant site through CH_2_Cl_2_ dissociation and also a methyl group that is well set up for loss as methane after B–H or P–H transfer. It is also well-established to mediate bond activation processes *via* sigma-bond metathesis, and related, processes,^23^,^24^ while the {RhCp*} fragment more generally catalyzes C–H, B–H, and P–H activation and bond coupling.^25^–^28^

We report here that complex **1** is an effective precatalyst for the dehydropolymerization of H_3_B·PPhH_2_, and also allows for a study of the elementary B–H/P–H activation processes occurring *via* a combined experimental and computational approach. In particular the order of B–H/P–H activation is determined in these systems, as well as a subsequent isomerization and P–B bond forming events. This provides insight into both the order of events and the likely intermediates involved in dehydropolymerization of phosphine–boranes.

## Results and discussion

### Catalysis: dehydrocoupling of H_3_B·PPhH_2_

Initial catalytic screening showed that complex **1** was an active precatalyst (1 mol%, 0.01 M, toluene, 100 °C, 72 h, system open to Ar) for the dehydropolymerization of H_3_B·PPhH_2_. After work-up, by precipitation into hexanes, the ^31^P{^1^H} NMR spectrum of the resulting solid shows a well-defined peak at *δ* –49.5, while in the ^11^B NMR spectrum a broad peak at *δ* –34.0 is observed (CDCl_3_), in good agreement with that reported by Manners *et al.* for polymer formed using the FeCp(CO)_2_(OTf)^5^ and [Rh(COD)_2_][OTf]^8^ catalysts. A simple doublet observed in the ^31^P NMR spectrum [*J*(HP) = 346 Hz] suggests a linear [H_2_BPPhH]_*n*_ structure to the polymer, rather than a branched structure that would invoke a quaternary phosphorus;^8^,^29^ although a low intensity ill-defined broad shoulder is observed between *δ* –50 to –60 that is suggestive of a small proportion of shorter chain oligomers or some branching. Consistent with this NMR data, the isolated polymer was shown by GPC to consist of a moderate molecular weight fraction (*M*_n_ = 15 000 g mol^–1^, PDI = 2.2) alongside lower molecular weight material (less than 1000 g mol^–1^). Although similar to that reported for the [Rh(COD)_2_][OTf] catalyst (*M*_w_ = 30 000 g mol^–1^)^7^,^8^ it falls short of the FeCp(CO)_2_(OTf) system at 1 mol% (*M*_n_ = 59 000 g mol^–1^, PDI = 1.6).^5^ The organometallic species in the catalytic mixture could not be identified. However, a signal corresponding to H_3_B·PMe_3_ was observed,^30^ suggesting dissociation (or substitution) of PMe_3_ in complex **1** during catalysis. If dehydropolymerization is carried out at a higher catalyst loading (5 mol%, 0.05 M, 72 hours) moderate molecular weight polymer is also formed as measured by GPC of hexane-precipitated material (*M*_n_ = 13 000 g mol^–1^, PDI = 1.5), and low molecular weight polyphosphino-borane is again present (less that 1000 g mol^–1^). The isolated polymer was also analysed by ESI-MS with a broad range of molecular weight chains [H{PPhHBH_2_}_*n*_PPhH_2_]^+^ and clear repeat units of {PHPhBH_2_} (*m*/*z* = 122) observed. The highest molecular weight polymer measured by this technique was *n* = 20, *m*/*z* = 2551.9.

**Scheme 4:**
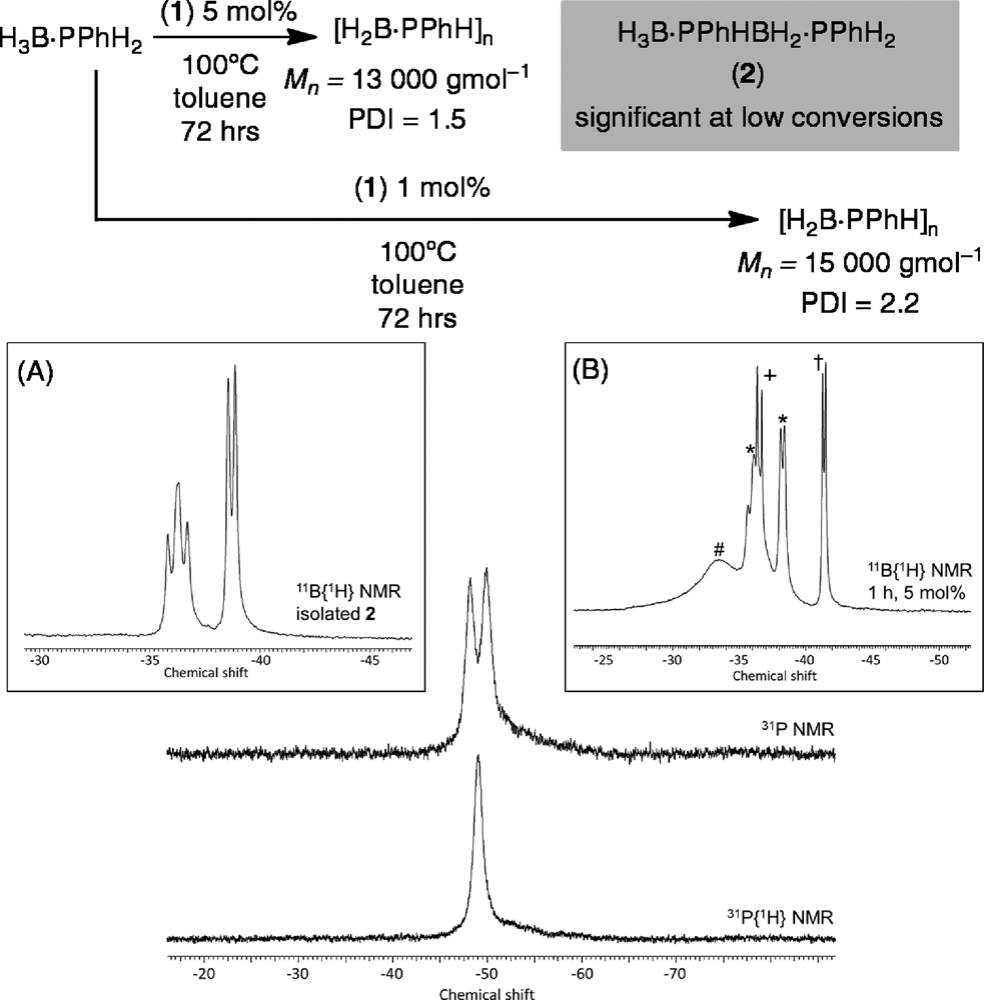
Purified [H_2_BPPhH]_*n*_ from the dehydrocoupling of H_3_B·PPhH_2_ catalysed by **1**, 1 mol%. Inset (A) purified **2**; (B) ^11^B{^1^H} NMR after 1 h: * H_3_B·PPhHBH_2_·PPhH_2_**2**, † H_3_B·PPhH_2_, + H_3_B·PMe_3_, # short chain oligomers.

Monitoring this reaction by ^11^B NMR spectroscopy shows that the H_3_B·PPhH_2_ monomer is consumed after only four hours, suggesting its relatively rapid oligomerization, but the slower formation of higher molecular weight polymer. If dehydropolymerization is stopped after only 1 hour the ^11^B{^1^H} NMR spectrum now shows signals due to H_3_B·PPhH_2_, a broad signal at *δ* –33.6 assigned to oligomer/polymer, H_3_B·PMe_3_ and significant amounts of a new compound assigned to the primary diboraphosphine H_3_B·PPhHBH_2_·PPhH_2_**2** (Scheme 4). Compound **2** is present in significantly greater amounts at 5 mol% loading [H_3_B·PPhH_2_ : **2**; 1 : 1, 5 mol%; 6 : 1, 1 mol%], and could be isolated in 25% yield by removing the toluene *in vacuo* and extracting with hexane to give a very pale yellow oil that could be fully characterized by NMR spectroscopy [*e.g.*^11^B{^1^H} *δ* –36.5 vt, *J* (PB) ∼ 70 Hz; –38.9 (d, *J* (PB) ∼ 50 Hz)], with data similar to both the secondary diboraphosphine H_3_B·PPh_2_BH_2_·PPh_2_H,^8^ and the primary analogue, H_3_B·PCyHBH_2_·PCyH_2_.^20^ The thermal dehydrogenation of H_3_B·PPhH_2_ in the absence of **1** (toluene, 0.625 M) produces **2** only slowly (∼50% conversion after 16 h) alongside a small amount of oligomeric product and unreacted H_3_B·PPhH_2_.

**Scheme 5:**
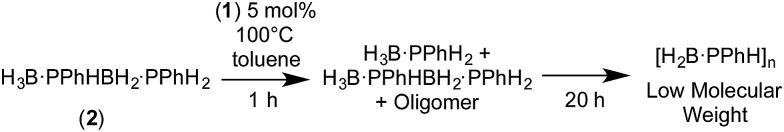
P–B bond cleavage and polymerisation of **2** as catalysed by **1**.

The lack of significant change in *M*_n_ on increasing the catalyst loading from 1 to 5 mol% suggests that a coordination chain-growth type mechanism is not operating, in which the polymer chain grows on the metal centre by successive monomer insertion events, as suggested for FeCp(CO)_2_(OTf) system for phosphine–borane and [Rh(xanthphos)]^+^ for amine–borane dehydropolymerization.^5^,^31^ Under this mechanistic model lower catalyst loadings would be expected to lead to higher molecular weight polymer, although such an analysis can be complicated by the fact that the metal has to both dehydrogenate and couple the reactive monomers.^32^ Instead, that at short reaction times **2** is observed in significant quantities, especially at higher catalyst loadings, and H_3_B·PPhH_2_ is completely consumed after only 4 hours hints at a step-growth-type mechanism, as suggested for [Rh(COD)Cl]_2_-catalyzed systems.^29^ Under this regime, a greater catalyst loading might be expected to increase the molecular weight of the resulting polymer.^29^,^32^ However the analysis of the mechanism of polymer growth is further complicated by the fact that both isolated **2** and higher *M*_n_ polymer undergo P–B bond cleavage in the presence of **1**. For example, heating **2** in the presence of 5 mol% **1** for 1 hour (100 °C, toluene) resulted in a mixture of **2**, H_3_B·PPhH_2_ (approx. 3 : 1 ratio by ^11^B{^1^H} NMR spectroscopy) and signals assigned to oligomers. Further heating overnight resulted in complete consumption of **2** and H_3_B·PPhH_2_ to reveal signals in the ^11^B NMR spectrum consistent with low molecular weight polymer, Scheme 5. Heating a sample of high molecular weight polymer (100 °C, toluene) with 5 mol% **1** also resulted in P–B cleavage events, with lower molecular weight species observed by ^31^P NMR spectroscopy. Linear diborazanes have also been observed to undergo B–N bond cleavage and product redistribution processes through both thermal and metal catalysed pathways, with a mixture of monomeric amine–borane and oligomeric products generated.^33^

On balance we thus suggest that a process in which reversible chain transfer between an oligomer (polymer) bound to a metal centre and free H_3_B·PPhH_2_, either initially present or generated by P–B bond cleavage, accounts best for these observations. We have previously demonstrated similar behaviour (as monitored by ESI-MS) using H_3_B·NH_3_ and a [Ir(PCy_3_)_2_(H)_2_]^+^ fragment.^34^

### Catalysis: dehydrocoupling of H_3_B·PPh_2_H

**Scheme 6:**
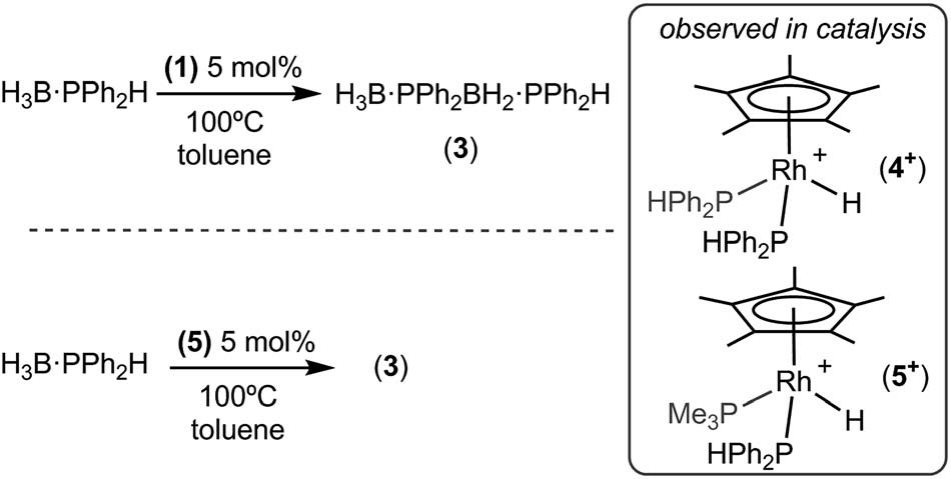
The dehydrocoupling of H_3_B·PPh_2_H as catalysed by **1** and **5** to form **3**.

To further probe the mechanism of dehydrocoupling using **1** the secondary phosphine–borane H_3_B·PPh_2_H was used, which has been shown to afford the diboraphosphine H_3_B·PPh_2_BH_2_·PPh_2_H **3** or cyclic species depending on dehydrocoupling conditions.^8^ Treatment of precatalyst **1** (5 mol%, 0.0313 M, 100 °C, toluene, 16 h) with H_3_B·PPh_2_H resulted in almost full conversion to **3** (95% by ^31^P and ^11^B NMR spectroscopy), Scheme 6. Analysis of the ^31^P{^1^H} NMR spectrum post-catalysis showed one dominant phosphine-containing organometallic species, as a doublet at *δ* 26.7 [*J* (RhP) = 139 Hz] which splits into a doublet of doublets in the ^31^P NMR spectrum [*J* (PH) = 391 Hz], demonstrating a direct P–H bond. H_3_B·PMe_3_ was also observed to be formed. The ^1^H NMR spectrum of the reaction mixture showed a doublet of triplets at *δ* –11.36 which simplified to a doublet upon ^31^P decoupling, suggesting a rhodium-bound hydride coupling to two phosphorus centres. ESI-MS showed one dominant peak at *m*/*z* = 611.15, with an isotope pattern that corresponds to the cation [RhCp*(H)(PPh_2_H)_2_]^+^, **4^+^**, fully consistent with the NMR data. Species closely related to cationic **4^+^** have been previously structurally characterised.^35^,^36^ Addition of Hg to the catalytic mixture after 4 hours resulted in no significant change to the overall conversion or rate, suggesting that the catalyst is not colloidal.^14^

The diphenylphosphine ligands required for the formation of cation **4^+^** likely result from P–B cleavage of the starting material H_3_B·PPh_2_H and resulting exchange at the metal centre to release PMe_3_, which is trapped as H_3_B·PMe_3_. Following the temporal evolution of catalysis using ^31^P{^1^H} NMR spectroscopy and ESI-MS^37^ showed that after 1 hour **4^+^** was present, but also a pair of doublet of doublet resonances at *δ* 19.2 and 2.3 were observed, that correlate with signals in the ESI-MS spectrum assigned to the cation [RhCp*(H)(PMe_3_)(PPh_2_H)]^+^ (**5^+^**). After 4 hours at 100 °C complex **4^+^** was dominant, suggesting that the cation **5^+^** evolves to give **4^+^** during catalysis. The ESI-MS also revealed signals with isotopic patterns which correspond to [RhCp*(PPh_2_·BH_3_)(PPh_2_H)_2_]^+^ (at *m*/*z* = 809.23) and [RhCp*(PPh_2_·BH_2_PPh_2_·BH_3_)(PPh_2_H)_2_]^+^ (*m*/*z* = 1007.31) which we assume are Rh–P bound (*vide infra*). Phosphido-borane species have been detected and proposed as catalytic intermediates in phosphine–borane dehydrocoupling in systems based on the {Rh(Ph_2_P(CH_2_)_3_PPh_2_)}^+^ and {FeCp(CO)}^+^ fragments.^5^,^19^,^20^ Addition of a further 20 equivalents of H_3_B·PPh_2_H to this reaction mixture post catalysis and heating to 100 °C resulted in complete conversion to diboraphosphine **3** after 22 h, suggesting that cation **4^+^** is active in catalysis. Further evidence for complexes of general formula [RhCp*(H)(PR_3_)_2_]^+^ being the active species comes from the isolation of **5** as pure material as the [BAr^F^_4_]^–^ salt (*vide infra*). Complex **5** is also a competent precatalyst for the dehydrocoupling of H_3_B·PPh_2_H (5 mol%, 100 °C) reaching completion within 22 hours. Again, cation **4^+^** is observed to be formed in the reaction mixture by ^31^P NMR spectroscopy, and the associated release of PMe_3_ was confirmed by the detection of H_3_B·PMe_3_. Addition of PPh_3_ (10 equivalents) to complex **5** and monitoring by ESI-MS shows, after 2 hours at 298 K, the formation of [RhCp*(H)(PMe_3_)(PPh_3_)]^+^ (*m*/*z* = 577.17) showing that phosphine exchange also occurs at 298 K. At room temperature, neither *in situ* generated **4**, or pure **5**, displayed any reactivity towards one equivalent of H_3_B·PPh_2_H. This suggests that under these conditions phosphine–borane is not a competitive ligand with phosphine, requiring higher temperatures and a large excess to promote reactivity at the metal center when there are two phosphines bound. The generation of vacant sites has been suggested to be important in the mode of action of FeCp(CO)_2_(OTf) in dehydrocoupling.^5^ Consistent this we show next that **1**, which is a masked source of {RhCp*Me(PMe_3_)}^+^ and thus does not require phosphine dissociation, reacts very rapidly with H_3_B·PHPh_2_.

Overall these data show that the {RhCp*Me(PMe_3_)}^+^ precatalyst, and related species formed during catalysis such as cation **4^+^**, are implicated in the dehydrocoupling/dehydropolymerization of both primary and secondary phosphine–boranes. In order to determine the role the metal fragment plays in this, the stoichiometric reactivity was studied, as is described next.

### Stoichiometric reactivity with H_3_B·PPh_2_H

**Scheme 7:**
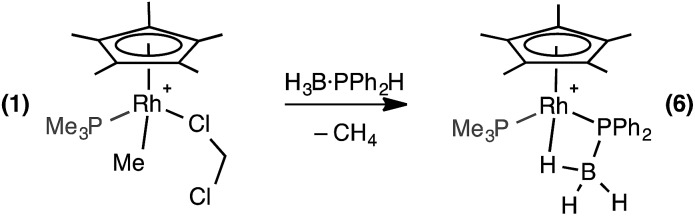
Complex **6**. [BAr^F^_4_]^–^ anions are not shown.

Reaction of 1 equivalent of H_3_B·PPh_2_H with **1** at room temperature in CD_2_Cl_2_ solution resulted in rapid effervescence and a colour change from orange to yellow. ^31^P{^1^H} NMR spectroscopy of the resulting solution showed one sharp doublet of doublets at *δ* –6.6 [*J* (RhP) = 139 Hz, *J* (PP) = 22 Hz] assigned to the PMe_3_ ligand and one broad peak at *δ* 6.9 [fwhm = 222 Hz] assigned to a phosphine–borane moiety, which was essentially unchanged in line shape in the ^31^P NMR spectrum. The ^1^H NMR spectrum demonstrated a lack of P–H and Rh–Me signals, and dissolved CH_4_ was detected (*δ* 0.21^38^). A very broad peak was observed at *δ* 0.3 (relative integral 2H) which sharpens on ^11^B decoupling and splits into two distinct resonances at *δ* 0.49 and *δ* –0.03 in a 1 : 1 ratio. A broad peak is observed at *δ* –10.81 that also sharpens on decoupling ^11^B, under which conditions it also resolves into a broad doublet of doublet of doublets. These 3 upfield resonances are assigned to a BH_3_ unit binding to the metal centre through one Rh–H–B 3 centre-2 electron bond that is not undergoing exchange on the NMR timescale between terminal and bridging environments. In the ^11^B NMR spectrum a signal at *δ* –45.5 was observed, shifted slightly upfield from H_3_B·PPh_2_H [*δ* –40.1]. Overall, these data are consistent with the formation of a phosphido-borane complex which also has a rather tight β-B-agostic interaction: [RhCp*(PPh_2_·BH_3_)(PMe_3_)][BAr^F^_4_] (**6**), Scheme 7.

**Fig. 1:**
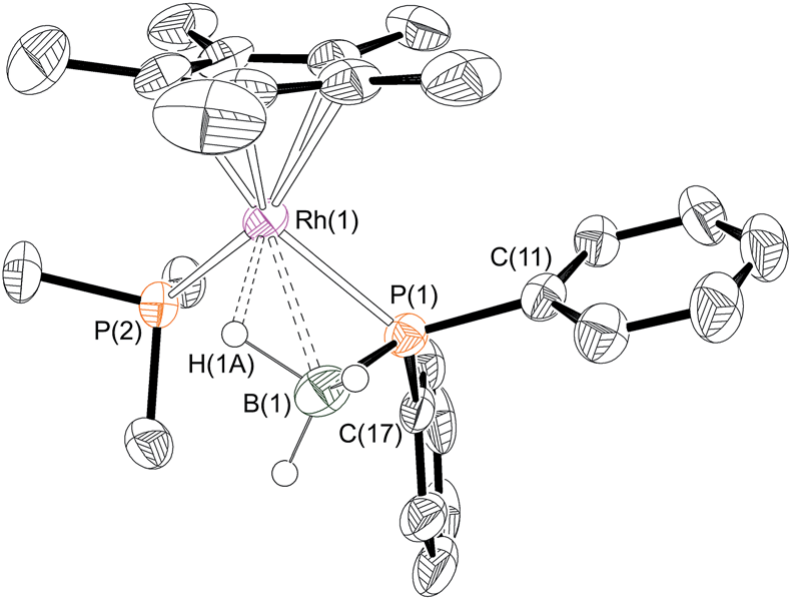
X-ray molecular structure of [RhCp*(PPh_2_·BH_3_)(PMe_3_)][BAr^F^_4_] **6**. [BAr^F^_4_]^–^ anion and selected hydrogen atoms omitted for clarity. Ellipsoids shown at 50% probability. Selected bond lengths (Å) and angles (°): P(1)–B(1) 1.896(4), Rh(1)–P(1) 2.302(1), Rh(1)–B(1) 2.464(4), Rh(1)–P(2) 2.3241(10), Rh(1)–Cp* (centroid) 1.859; P(1)–Rh(1)–P(2) 95.35(3), Rh(1)–P(1)–B(1) 71.13(13), B(1)–P(1)–C(11) 116.09(19), B(1)–P(1)–C(17) 119.55(19), C(11)–P(1)–C(17) 103.34(15).

Yellow crystals were grown from the reaction mixture and isolated in good yield (76%). A resulting single-crystal X-ray diffraction study (Fig. 1) confirmed the structure as a phosphido-borane species with a β-B-agostic interaction. Although the B–H hydrogen atoms were located in the difference map, in the final refinement they were placed at fixed positions. The P–B distance in **6** [1.896(4) Å] is slightly shorter than the reported P–B bonds in H_3_B·P(Mes)_2_H [1.938 Å]^39^ and in H_3_B·P(*p*-CF_3_C_6_H_4_)_2_H [1.917(2) Å]^10^ (the structure of H_3_B·PHPh_2_ has not been reported) but longer than most of the crystallographically characterised monomeric phosphino-boranes, which usually bear bulky substituents to prevent oligomerisation (1.76–1.88 Å).^40^,^41^ The NMR data are also characteristic of a four-coordinate boron, indicating a β-B-agostic structure rather a phosphino-borane complex with concomitant hydride transfer to Rh. Further evidence for a β-B-agostic structure was obtained from DFT calculations^42^ which revealed a significant lengthening of the agostic B(1)–H(1A) bond (1.39 Å) compared to the non-agostic B(1)–H(1B)/H(1C) bonds (both 1.21 Å), as well as a short Rh(1)L–H(1A) contact of 1.72 Å. Other heavy atom bond metrics were in good agreement with experiment, including a computed P(1)–B(1) distance of 1.92 Å (see ESI† for full details). β-B-agostic interactions of this type have been previously observed in phosphido-borane complexes with Mo,^43^,^44^ Fe,^45^ Ti,^46^ Rh^20^ and alkaline earth metals,^47^–^49^ but the structure of **6**, and the salient NMR data, most closely resemble the neutral compound [FeCp(PPh_2_·BH_3_)(CO)].^5^ Finally, the β-B-agostic interaction observed in **6** is in contrast with valence isoelectronic [RhCp*(H)(H_2_C=CH_2_)P(OMe)_3_][BF_4_] that although in equilibrium with the corresponding β-agostic complex, favours the former.^50^ Complex **6** is stable in CD_2_Cl_2_ solution for at least 2 weeks.

**Scheme 8:**
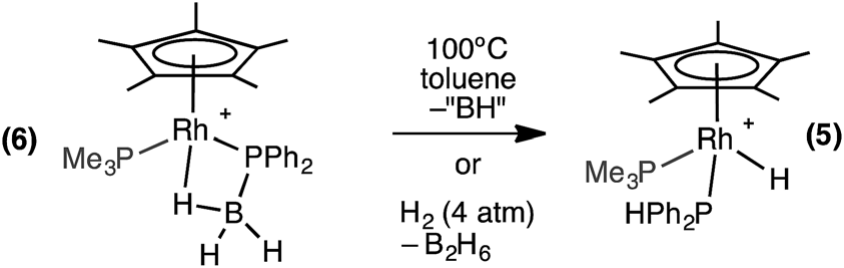
Reactivity of complex **6**. [BAr^F^_4_]^–^ anions are not shown.

The β-B-agostic interaction in **6** could be viewed as a source of masked highly reactive, phosphino-borane *i.e.* {H_2_BPPh_2_}/{Cp*RhH(PMe_3_)}^+^ in which Rh–H acts as a Lewis base to boron and phosphorus a Lewis base to the Rh-center. The parent H_2_BPH_2_ has been shown to oligomerise at [Ti] centres,^51^–^53^ or form polymeric materials when generated *in situ*.^16^ To explore whether phosphino-borane H_2_BPPh_2_ could be liberated, as signalled by the formation of [Ph_2_PBH_2_]_*n*_ (*n* = 3 or 4),^8^,^16^ complex **6** was heated to 100 °C in toluene for 4 hours. However, the only product that could be observed by NMR spectroscopy was the P–B cleavage product **5**, while the fate of the remaining {BH} is unclear (Scheme 8). This process is therefore the likely route to formation of **5** from **1** under catalytic conditions. Complex **5** could also be formed cleanly by pressurising a 1,2-difluorobenzene solution of **6** with H_2_ (∼4 atm) at room temperature for 16 hours. In this case the boron-containing by-product of P–B cleavage was determined to be B_2_H_6_ by ^11^B NMR spectroscopy.^54^ Complex **6** does not react with H_3_B·PPh_2_H at 298 K, reflecting the strong Rh···H–B interaction.

### Stoichiometric reactivity with H_3_B·PCy_2_H

**Fig. 2:**
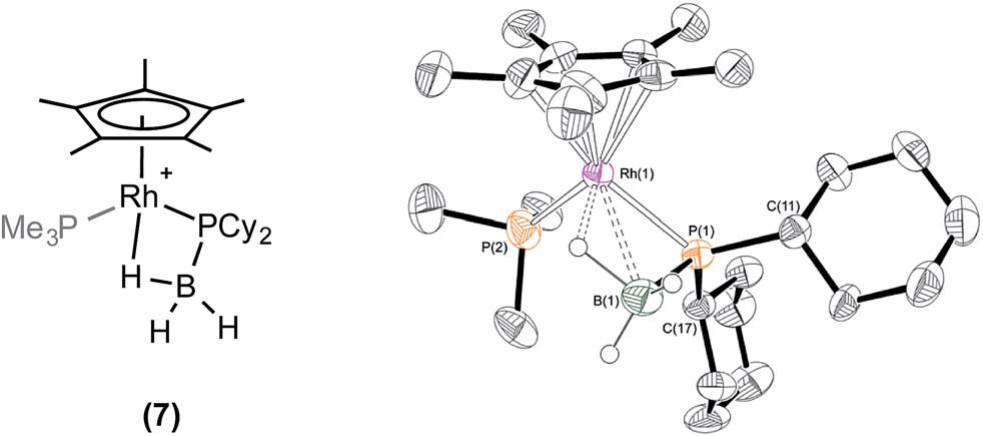
X-ray molecular structure of [RhCp*(PCy_2_·BH_3_)(PMe_3_)][BAr^F^_4_] (**7**). [BAr^F^_4_]^–^ anion and selected hydrogen atoms omitted for clarity. Ellipsoids shown at 50% probability. Selected bond lengths (Å) and angles (°): P(1)–B(1) 1.910(7), Rh(1)–P(1) 2.3425(14), Rh(1)–B(1) 2.468(7), Rh(1)–P(2) 2.2878(16), Rh(1)–Cp*(centroid) 1.875; P(1)–Rh(1)–P(2) 93.94(6), Rh(1)–P(1)–B(1) 70.1(2), B(1)–P(1)–C(11) 109.3(3), B(1)–P(1)–C(17) 118.3(3), C(11)–P(1)–C(17) 110.0(2).

Reaction of one equivalent of H_3_B·PCy_2_H with **1** in CD_2_Cl_2_ resulted in rapid effervescence (methane). Analysis by NMR spectroscopy after 5 minutes indicated the formation of a complex very similar to **6**: [RhCp*(PCy_2_·BH_3_)(PMe_3_)][BAr^F^_4_], **7**, in particular an upfield signal in the ^1^H NMR spectrum is observed at *δ* –11.42, assigned to the β-B-agostic interaction. Single crystals of **7** suitable for X-ray diffraction were grown from a cooled CH_2_Cl_2_/pentane solution, and the solid state structure confirms a β-B-agostic phosphido-borane ligand chelating with the rhodium centre (Fig. 2). The bond lengths and angles in the structure were broadly similar to those found in **6**, and this was also borne out when comparing the DFT-optimised structures (ESI†). In contrast to complex **6**, **7** is not stable in CD_2_Cl_2_ solution, decomposing fully after approximately 24 hours to form a mixture, from which the major component could be characterised spectroscopically as [RhCp*(H)(PCy_2_H)(PMe_3_)][BAr^F^_4_] **8**, *i.e.* the analogue of **5**. This low temperature instability to P–B cleavage can be contrasted with **6**, that only decomposes upon heating. P–B bond cleavage in phosphine–borane complexes has previously been noted to be a function of both the electron withdrawing nature and the steric bulk of the P–substituents, the latter suggested to be dominating here.^3^,^20^

### Stoichiometric reactivity of H_3_B·P^*t*^Bu_2_H

**Fig. 3:**
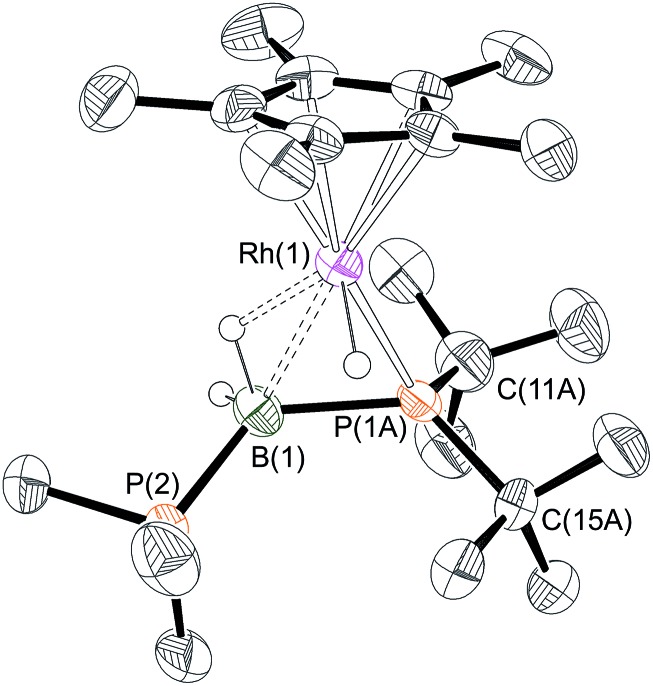
X-ray molecular structure of (**9**). The P^*t*^Bu_2_ unit is disordered over 2 sites, only the major component labelled, *i.e.* P(1A), C(11A), is shown. [BAr^F^_4_]^–^ anion and selected hydrogen atoms omitted for clarity. Selected bond lengths (Å) and angles (°): P(1A)–B(1) 1.99(2), P(1B)–B(1) 1.901(14), B(1)–P(2) 1.918(5), Rh(1)–P(1A) 2.30(3), Rh(1)–P(1B) 2.258(14), Rh(1)–B(1) 2.431(5), Rh(1)–Cp* (centroid) 1.870; P(1A)–B(1)–P(2) 126.2(7).

One equivalent of H_3_B·P^*t*^Bu_2_H was added to complex **1** to explore further the effect of increasing the steric bulk at the phosphorus center. After mixing, the yellow solution rapidly turned dark red and effervescence was observed. Over the course of two hours at 298 K this intense colour was lost to give a yellow/orange solution. Analysis by ^31^P{^1^H} NMR spectroscopy of this final solution showed two broad peaks at *δ* 54.8 and –7.8, alongside minor unidentified species. The ^1^H NMR spectrum showed two resonances in the hydride region at *δ* –10.79 and –13.76 (the former being considerably broader but sharpened on decoupling ^11^B) which, in contrast to **6** and **7**, suggest the presence of both Rh–H–B and Rh–H groups respectively. A broad peak at *δ* 0.50 (BH, integral 1H) was also observed, in addition to phosphine and Cp* resonances. Moreover the ^11^B{^1^H} NMR spectrum revealed a broad virtual triplet at *δ* –45.4 [*J* (BP) ≈ 95 Hz] suggestive of coupling to two phosphorus centres. The structure of this new species was resolved by a single-crystal X-ray diffraction study (Fig. 3) to be [RhCp*(H)(P^*t*^Bu_2_BH_2_·PMe_3_)][BAr^F^_4_] **9**, in which the PMe_3_ ligand has migrated to the boron centre to afford a Lewis-base stabilised phosphino-borane, chelating to the rhodium centre through P^*t*^Bu_2_ and a β-B-agostic interaction. The P^*t*^Bu_2_ unit is disordered over two sites meaning that the P–B bond metrics cannot be discussed in detail, but it is similar to those observed in the phosphido-borane species **6** and **7**, suggesting a single P–B bond. DFT calculations on **9** provide optimised P(1A)–B(1) and P(2)–B(1) distances of 1.95 Å and 1.96 Å, respectively, consistent with single bond character. Lewis-base stabilised phosphino-boranes were first synthesised by Burg in 1978,^55^ and have recently been used by Scheer and coworkers to form metal complexes^16^,^51^,^53^ that can also undergo P–B coupling reactions.^52^ Similar phosphine ligand migration to a boron centre in a transient phosphino-borane has been previously proposed in the formation of [Rh(PPh_3_)_2_(PPh_2_BH_2_·PPh_3_)][BAr^F^_4_]^56^ which also has a Lewis base-stabilised phosphino-borane with a β-B-agostic interaction to the Rh(i) centre [Rh–B: 2.407(5); B–P: 1.915(5), 1.945(5) Å].

**Scheme 9:**
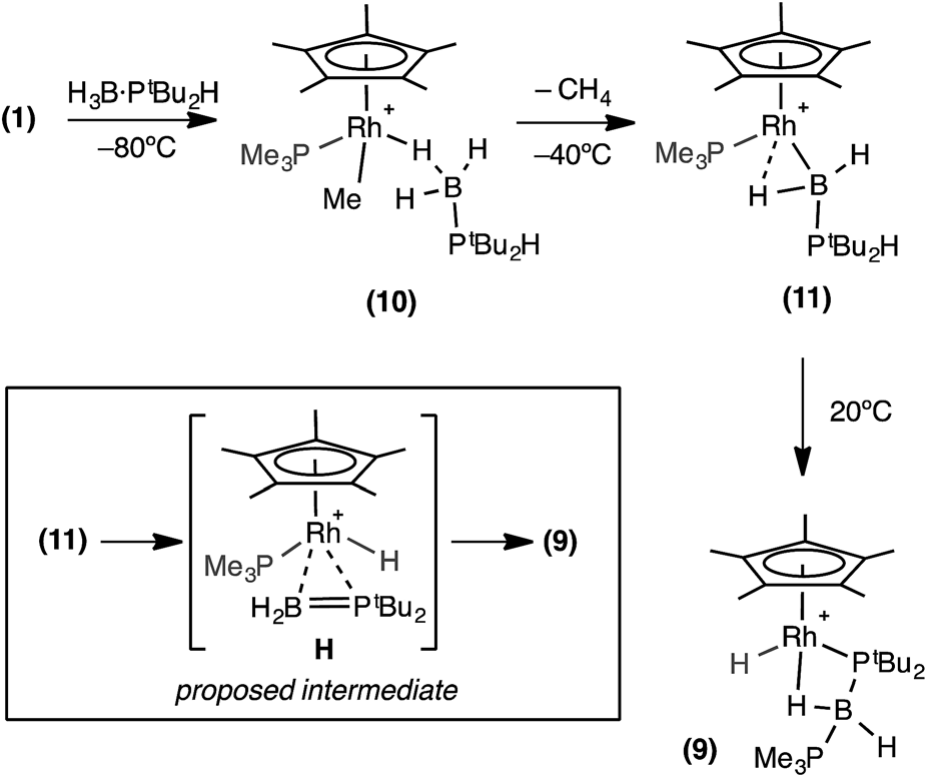
Formation of complex (**9**). Observed and proposed intermediates. [BAr^F^_4_]^–^ anions are not shown.

A low temperature NMR spectroscopy study was performed to help elucidate the mechanism by which **9** is formed, and in particular the identity of the observed dark red intermediate. CD_2_Cl_2_ solutions of H_3_B·P^*t*^Bu_2_H and **1** were combined at –78 °C to form a yellow solution after mixing. After loading into a precooled NMR spectrometer the ^31^P{^1^H} NMR spectrum at –80 °C showed a new species by a sharp doublet *δ* 8.3 and a broad signal *δ* 35.4, consistent with Rh–PMe_3_ and H_3_B·PR_2_H environments respectively. The ^1^H NMR spectrum was more revealing with a very broad upfield peak observed at *δ* –4.01 (3H relative integral) consistent with a Rh···H_3_B unit. A broad signal was also observed at *δ* 0.79 (3H relative integral), assigned to Rh–Me. The P–H bond is still intact, as shown by a doublet at *δ* 4.08 [*J*(HP) = 363 Hz] which collapsed to a singlet on ^31^P decoupling. These data suggest that this species is an η^1^-sigma complex with the bound dichloromethane molecule of **1** replaced by the phosphine–borane to form [RhCp*Me(PMe_3_)(η^1^-H_3_B·P^*t*^Bu_2_H)][BAr^F^_4_], **10**, Scheme 9 That only one B–H environment is observed, even at –80 °C, suggests rapid terminal/bridging B–H exchange on the NMR timescale. η^1^-Sigma binding with a variety of metal-ligand fragments has been observed for both phosphine- and amine–boranes, with low–energy exchange between bridging and terminal B–H sites observed on the NMR timescale.^57^–^60^ The ^11^B{^1^H} NMR spectrum shows a chemical shift at *δ* –44.8, characteristic^61^ of an η^1^-M···H_3_B·PR_3_ interaction, being barely shifted from free phosphine–borane (*δ* –42.9).

When this solution was warmed to –40 °C inside the spectrometer after approximately one hour a new species, **11**, was formed at the expense of complex **10**. The ^31^P{^1^H} NMR spectrum showed two new resonances at *δ* 25.1 and –1.9, as a broad peak and a sharp doublet respectively. The ^1^H NMR spectrum revealed the disappearance of the Rh–Me signal with concomitant appearance of dissolved CH_4_ (*δ* 0.15).^38^ Two broad peaks (both 1H relative integral) at *δ* 7.1 and *δ* –12.76 [d, *J* (RhH) = 38 Hz] were observed, both of which sharpen on decoupling ^11^B, and a doublet of multiplets at *δ* 4.68 [*J* (RhP) 380 Hz], consistent with a P–H group. In the ^11^B{^1^H} NMR spectrum there is a peak at *δ* 47.6, downfield shifted by 92.4 ppm compared to **10**. These data suggest that **11** corresponds to a base-stabilized boryl complex, [RhCp*(PMe_3_)(H_2_B·P^*t*^Bu_2_H)][BAr^F^_4_], featuring a strong α-B-agostic interaction, as the two, now diastereotopic, B–H groups do not undergo exchange.

As far as we are aware there is only one other reported base-stabilised α-B-agostic boryl complex, albeit featuring a dimeric motif,^62^ although examples that may be described as having α-B-agostic amino-boryl limiting structures have been discussed.^63^,^64^ DFT calculations on the dehydrogenation of H_3_B·NMe_2_H using the {Ir(PCy_3_)_2_(H)_2_}^+^ fragment suggest intermediates with structures closely related to **11**.^65^ Similar B–H activation and elimination of methane (under photolytic conditions) has been reported by Shimoi and co-workers to form M(η^5^-C_5_R_5_)(CO)_*n*_(BH_2_·PMe_3_) [*n* = 2, M = Mn; *n* = 3 W, Mo, R = H, Me] from the corresponding metal methyl precursors.^66^,^67^ Interestingly these, and other closely related complexes,^68^,^69^ only show small (*ca.* 13 ppm) downfield shifts, when compared to free H_3_B·PMe_3_, on formation of the boryl moiety, in contrast to the *ca.* 92 ppm shift observed between **10** and **11**. In fact the ^11^B chemical shift is more similar to complexes featuring 3-coordinate boron (*e.g. δ* 30–50).^63^,^70^–^72^ The ^1^H NMR spectrum of **11** shows a large *J* (RhH) coupling in the low field hydride-like signal [*J* (RhH) 38 Hz], whereas in complexes **6** and **7** no such coupling is observed. Moreover the other BH group resonates at rather low field (*δ* 7.11), compared with **6** (*δ* 0.49 and –0.03). In comparison, Shimoi's M(η^5^-C_5_R_5_)(CO)_*n*_(BH_2_·PMe_3_) species (which do not feature an α-B-agostic interaction) exhibit BH chemical shifts around 1.5,^66^ whereas hydrido-amino-boryls Ir(PMe_3_)_3_(H)Cl{B(H)(NCy_2_)}^70^ and [Rh(κ^3^-_P,O,P_-xantphos)(H){B(H)(N^i^Pr_2_)}(NCMe)][BAr^F^_4_]^31^ (featuring 3-coordinate boron) show B–H and ^11^B chemical shifts more like **11** [*δ*(^11^B) 43, 49 respectively]. These data suggest that complex **11** could also be described as a hydrido base–stabilised borylene complex, at least in a limiting form. However, it is also possible that a tight α-B-agostic interaction could induce a downfield shift in the ^11^B NMR spectrum, similar to α-C-agostic interactions probed by ^13^C NMR spectroscopy.^73^

**Fig. 4:**
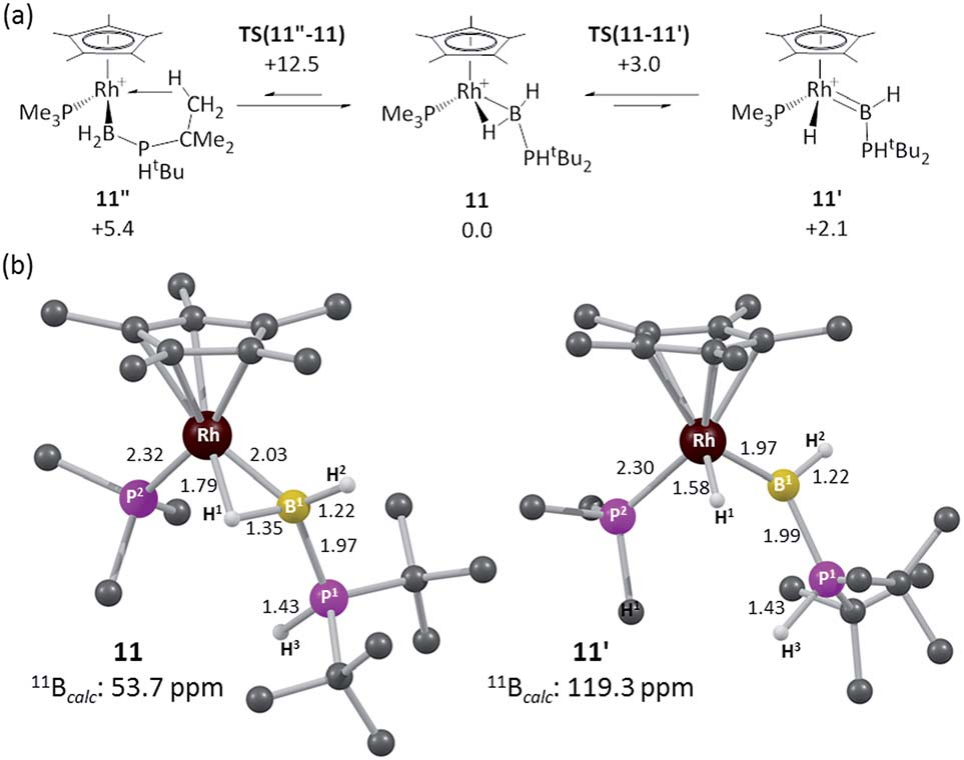
(a) Computed isomers and interconversions of [RhCp*(PMe_3_)(H_2_B·PH^*t*^Bu_2_)]^+^; (b) computed structures of α-B-agostic boryl complex **11** and hydrido base-stabilised borylene complex, **11′**. Selected distances are in Å and C-bound H atoms are omitted for clarity. Free energies are quoted relative to **11** set to 0.0 kcal mol^–1^ and are at the BP86-D3 (CH_2_Cl_2_) level; computed ^11^B chemical shifts are at the B3LYP(BS2)//BP86(BS1) level (see ESI for full details).†

In an attempt to resolve this structural ambiguity, dark red single crystals of **11** were grown at –20 °C, however the resulting structure was of poor quality and only showed the connectivity of the heavy atoms that demonstrate a Rh–B interaction (see ESI†). Instead both limiting forms were characterized *via* DFT calculations which revealed the α-B-agostic boryl (**11**) to lie 2.1 kcal mol^–1^ below the hydrido base-stabilised borylene complex (**11′**, see Fig. 4).^74^,^75^ This preference was reproduced with a range of other functionals. A third form, **11′′**, featuring an agostic interaction with one ^*t*^Bu C–H bond was also located and was 5.4 kcal mol^–1^ above **11** (see ESI†). Computed barriers suggest rapid interconversion between all three species, with **11** being the dominant species in solution. The computed structure of **11** exhibits a strong α-B-agostic interaction, with a short RhL–H^1^ contact of 1.79 Å and significant elongation of the B^1^–H^1^ bond (1.35 Å) compared to the terminal B^1^–H^2^ bond (1.22 Å). Further support for the α-B-agostic assignment was seen in the computed ^11^B chemical shifts, the value for **11** (*δ* 53.7 ppm) being both in good absolute agreement with experiment (*δ* 47.6) and significantly better than that computed for **11′** (*δ* 119.3 ppm).

Removal of the NMR tube from the spectrometer while at low temperature showed complex **11** to be responsible for the intermediate deep red colour observed. Warming to room temperature over two hours produced the yellow/orange solution in which **9** was the major product (Scheme 9). The formation of complex **9** was signalled in the ^11^B NMR spectrum by a dramatic upfield shift to *δ* –45.4 (computed value = –49.1). Complex **9** forms from **11** by P–H activation and migration of the PMe_3_ ligand to the boron centre. We suggest that this may occur *via* a phosphino-borane intermediate (**H**, Scheme 9) that then undergoes intramolecular attack by PMe_3_. A structural analogue of **H** has been reported by Bourissou and co-workers in [Cy_2_PB(C_6_F_5_)_2_Pt(PMe_3_)_2_].^76^

**Fig. 5:**
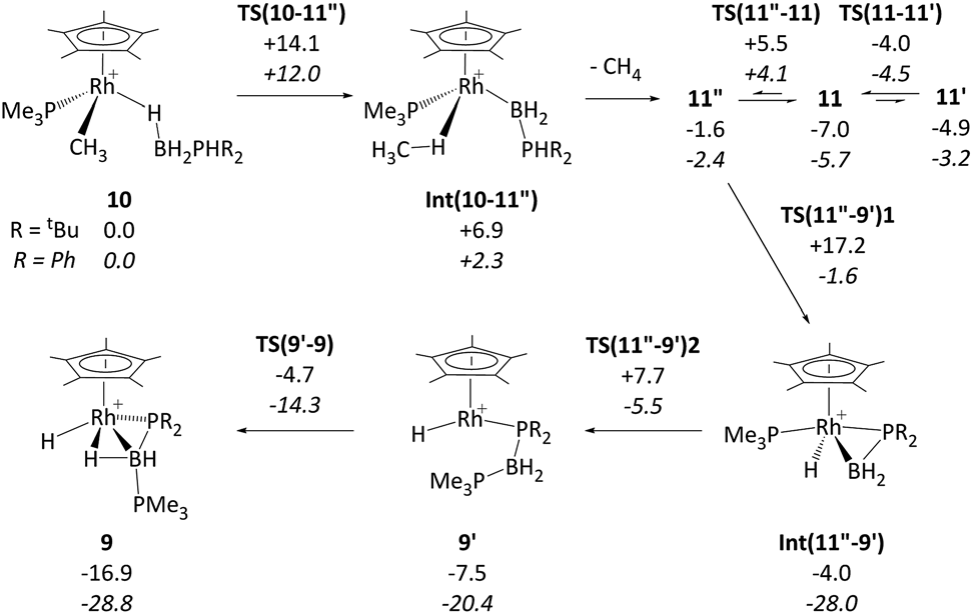
Computed free energy reaction profile (kcal mol^–1^, BP86-D3 (CH_2_Cl_2_) level) for formation of **9** from **10** (R = ^*t*^Bu) with equivalent data for R = Ph provided in italics. All free energies are quoted relative to **10** + free H_3_B·PHR_2_ at 0.0 kcal mol^–1^; see Fig. 4 for details of species **11**, **11′** and **11′′** when R = ^*t*^Bu.

**Fig. 6:**
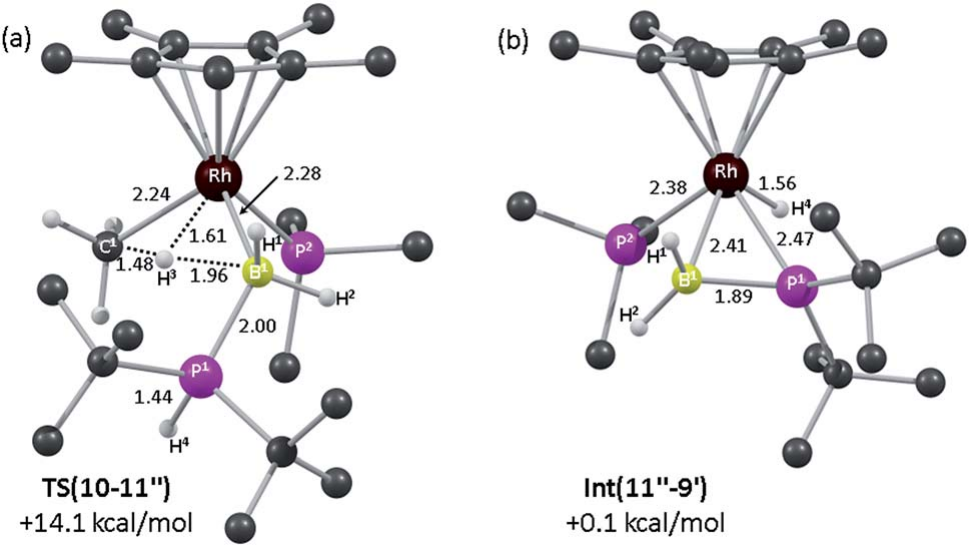
Computed structures and free energies (BP86-D3 (CH_2_Cl_2_)) for (a) **TS(10-11′′)** and (b) **Int(11′′-9′)**; selected distances are in Å and C-bound H atoms are omitted for clarity.

DFT calculations were employed to assess this proposed mechanism and the results are summarised in Fig. 5 (which also presents data for the analogous reaction of H_3_B·PHPh_2_ that will be discussed below). Starting from species **10** (set to 0.0 kcal mol^–1^) B–H activation involves a sigma-CAM process^21^*via***TS(10-11′′)** (*G* = +14.1 kcal mol^–1^) to generate intermediate **Int(10-11′′)** (*G* = +6.9 kcal mol^–1^) featuring both phosphine-stabilised boryl and methane ligands. **TS(10-11′′)** exhibits a short Rh–H^3^ distance of 1.61 Å, indicative of significant Rh(v) character at this point (see Fig. 6(a) which also gives the labelling scheme employed). Facile loss of CH_4_ initially yields the C–H agostic species **11′′** (*G* = –1.6 kcal mol^–1^) which readily isomerizes to **11** at –7.0 kcal mol^–1^.

The onward reaction of **11** requires an initial rearrangement back to **11′′**. This proves to be necessary as it swaps the strong α-B-agostic interaction in **11** for a weak C–H agostic in **11′′** which then allows the transfer of H^4^ from P^1^ to Rh *via***TS(11′′-9′)1** (*G* = +17.2 kcal mol^–1^). The intermediate generated, **Int(11′′-9′)** (*G* = –4.0 kcal mol^–1^, Fig. 6(b)), features a {^*t*^Bu_2_PBH_2_} phosphino-borane moiety and is equivalent to the postulated intermediate **H** of Scheme 8. **Int(11′′-9′)** exhibits a P^1^–B^1^ distance of 1.89 Å, lying between the computed B–P distances of H_3_B–P^*t*^Bu_2_H (1.96 Å) and H_2_B=P^*t*^Bu_2_ (1.83 Å), see ESI.† This suggests a degree of back-bonding from the metal to the phosphinoborane, but perhaps less than is implied in [Cy_2_PB(C_6_F_5_)_2_Pt(PMe_3_)_2_],^76^ for which a P–B distance of 1.917(3) Å has been determined crystallographically. It is also notable that the hydride and {BH_2_} unit in **Int(11′′-9′)** are orientated *trans*, while the PMe_3_ and BH_2_ are *cis*. Thus B^1^–P^2^ coupling can occur *via***TS(11′′-9′)2** with a modest barrier of only +11.7 kcal mol^–1^ to give **9′**, which is related to the observed species **9** (*G* = –16.9 kcal mol^–1^) *via* rotation about the new B–PMe_3_ bond. The overall barrier for the formation of **9** from **11** is 24.2 kcal mol^–1^, and so is somewhat higher than that for the formation of **11** from **10** (14.1 kcal mol^–1^). These relative barriers are qualitatively consistent with the rapid formation of **11** at low temperature, compared to the onwards slower generation of **9** (room temperature, 2 hours). The higher barrier for P–H activation (from **11**), compared to the initial B–H activation (from **10**) is also consistent with previous experimental and computational studies on related amine–borane chemistry,^65^,^77^ and for H_3_B·P^*t*^Bu_2_H dehydrocoupling using the [Rh(Ph_2_P(CH_2_)_3_PPh_2_)]^+^ fragment.^19^

### Reactions with H_3_BPCy_3_

**Scheme 10:**
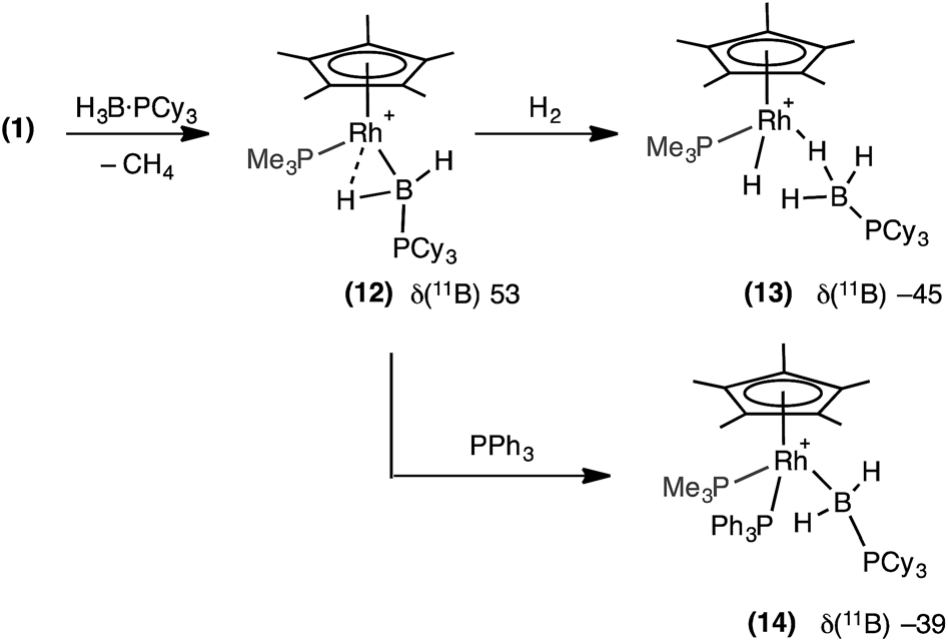
Spectroscopically observed boryl complex (**12**) and reactivity with H_2_ and PPh_3_. [BAr^F^_4_]^–^ anions are not shown.

In an attempt to produce a stable boryl complex, H_3_B·PCy_3_ was reacted with **1** in the anticipation that the lack of a P–H group would stop onward reactivity. Reaction formed a deep red phosphine–boryl complex which was characterised spectroscopically as [RhCp*(PMe_3_)(H_2_B·PCy_3_)][BAr^F^_4_], **12**, which was stable at room temperature for 4 hours before any decomposition (to unidentified products) was observed (Scheme 10). The NMR spectra of complex **12** are very similar to **11**. In particular in the ^1^H NMR spectrum a broad upfield peak at *δ* –13.57 is observed,^78^ along with the characteristic downfield shift of the ^11^B NMR resonance (*δ* 53.0). Attempts to crystallise **12** resulted in intractable oils. Addition of H_2_ (4 atm) to **12** resulted in loss of the deep red colour to form an orange/brown solution, which was characterised spectroscopically as [RhCp*H(PMe_3_)(H_3_B·PCy_3_)][BAr^F^_4_], **13**. ^11^B NMR spectroscopy at room temperature revealed a considerable upfield shift in the ^11^B NMR shift in which the boryl signal had been replaced by one at *δ* –45.6, characteristic of a σ-phosphine–borane. In the ^1^H NMR spectrum (under a H_2_ atmosphere) one very broad upfield signal was observed at *δ* –4.14. Cooling to –60 °C resolved this into a quadrupolar broadened peak at *δ* –4.07 (relative integral 3H), assigned to a Rh···H_3_B unit, and a sharp doublet of doublets at *δ* –11.53 (integral 1H), assigned to Rh–H. These are exchanging at room temperature, and we suggest that the mechanism for this is likely be through a boryl-dihydrogen complex [RhCp*(PMe_3_)(H_2_B·PCy_3_)(H_2_)][BAr^F^_4_], operating *via* a sigma-CAM mechanism.^21^ Addition of PPh_3_ to **12** results in a loss of the high-field signal, and the appearance of two signals at *δ* 2.42 and 0.23 in the ^1^H{^11^B} NMR spectrum assigned to RhB*H*_2_PCy_3_. Furthermore the ^11^B NMR spectrum shows a significant upfield shift to *δ* –39.5, consistent with previously reported, non-α-B-agostic, base-stabilised boryls.^66^–^69^ These, and associated ^31^P{^1^H} NMR data, signal the formation of complex **14**: [RhCp*(PMe_3_)(PPh_3_)(H_2_B·PCy_3_)][BAr^F^_4_].

### D-labelling experiments

**Scheme 11:**
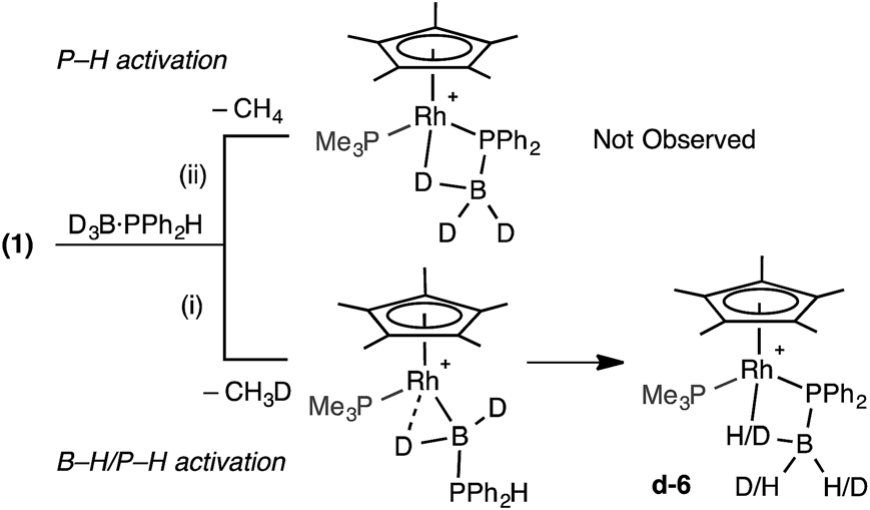
D-labelling experiments.

The observation of the α-B-agostic boryl intermediate **11** en route to complex **9** led us to speculate upon the mechanism of formation of the phosphido-borane species **6** (and **7**), and whether Ph- and Cy-analogues of **11** are intermediates in the formation of these species from **1** and the corresponding phosphine–borane. To probe this D_3_B·PHPh_2_ was added to **1**. Two scenarios follow: (i) B–D activation followed by P–H activation would lead to a {HD_2_BPR_2_} unit in the final product and the release of CH_3_D, or (ii) initial P–H activation would result in liberation of CH_4_ and no incorporation of ^1^H into the borane (Scheme 11). ^31^P and ^11^B NMR spectroscopy confirmed clean formation of the phosphido-borane product; while ^1^H and ^2^H NMR spectroscopy (ESI†) showed H and D in all positions of the β-B-agostic borane, with an overall relative integral of 1H measured from the ^1^H NMR spectrum indicating a H : D ratio of 1 : 2. This suggests route (i) is operating, as observed spectroscopically for complex **11**. That ^1^H signals are observed in all 3 B–H positions of the final product **d-6** suggests slow exchange between terminal and bridging positions which was confirmed by a spin saturation ^1^H NMR exchange experiment.^79^ CH_3_D is observed [*δ* 0.19, t, *J* (HD) 2.0 Hz, CD_2_Cl_2_], that disappears on degassing the solution.

**Fig. 7:**
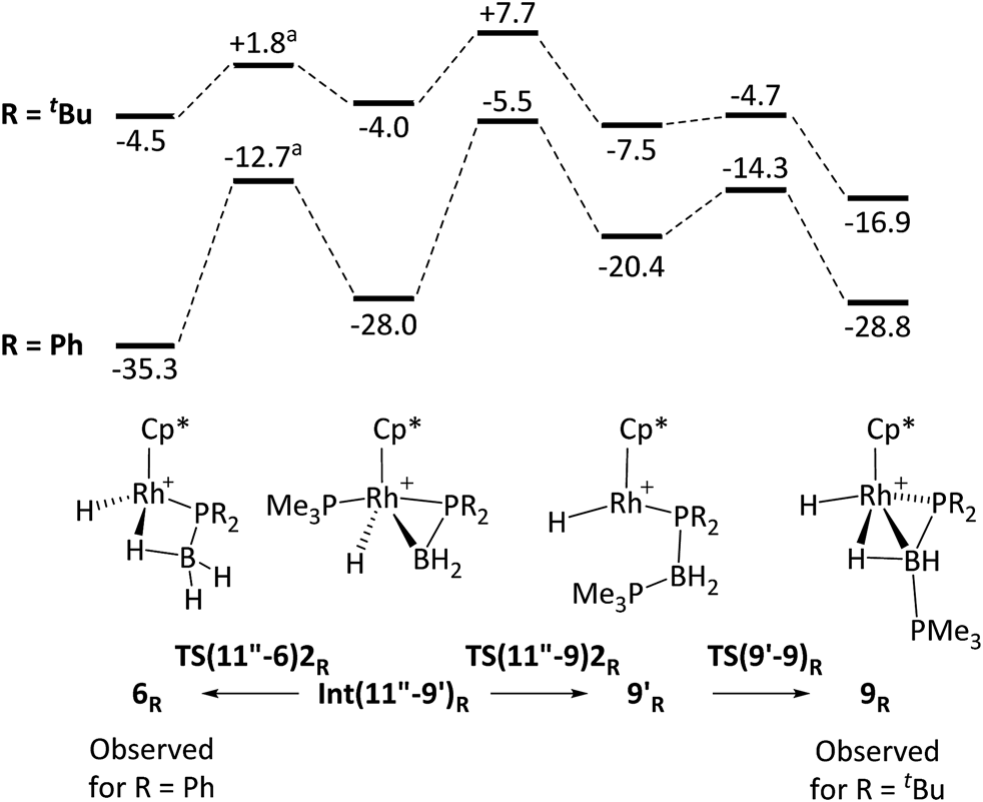
Computed free energy reaction profiles (kcal mol^–1^, BP86-D3 (DCM) level) for formation of **6_R_** and **9_R_** from phosphino-borane adducts **Int(11′′-9′)_R_**: (R = ^*t*^Bu and Ph). All free energies are quoted relative to **10** set to 0.0 kcal mol^–1^. ^a^An intermediate corresponding to a *ca.* 90° rotation of the phosphino-borane ligand was located between **Int(11′′-9′)_R_** and **6_R_** and only the energy of the higher-lying transition state is indicated. See text and ESI† for full details.

The observation of a phosphido-borane complex [RhCp*(PR_2_·BH_3_)(PMe_3_)]^+^ when R = Ph (**6**) and Cy (**7**) is in sharp contrast to the formation of [RhCp*(H)(PR_2_·BH_2_·PMe_3_)]^+^ when R = ^*t*^Bu (**9**). The above labelling studies (R = Ph) and calculations (R = ^*t*^Bu and Ph, Fig. 5) are all consistent with initial B–H activation to form [RhCp*(H_2_B·PHR_2_)(PMe_3_)]^+^, **11_R_**, as a common intermediate. Fig. 5 also indicates that the reaction profile for the formation of **9_Ph_** from **11_Ph_** would follow a similar course to the ^*t*^Bu system, although significantly different energetics are seen around the β-H transfer step from **11′′_R_**, which has a much lower barrier and is far more exergonic when R = Ph. The onward reactivities of the resultant phosphino-borane intermediates **Int(11′′-9′)_R_** are compared in Fig. 7. The stability of **Int(11′′-9′)_Ph_** (*G* = –28.0 kcal mol^–1^) means the subsequent P–B coupling step towards **9_Ph_** encounters a significant barrier of 22.5 kcal mol^–1^*via***TS(11′′-9′)2_Ph_** at –5.5 kcal mol^–1^. Alternatively, we found that the phosphino-borane ligand in **Int(11′′-9′)_Ph_** can undergo a two-step rotation that leads directly to **6_Ph_**. This process involves first a transition state **TS(11′′-6)2_Ph_** at –12.7 kcal mol^–1^ which leads to an intermediate in which the phosphino-borane ligand lies parallel to the Rh–Cp* (centroid) direction with the {BH_2_} moiety adjacent to the Cp* ring (**Int(11′′-6)2_Ph_**, *G* = –17.4 kcal mol^–1^). The rotation is completed *via* a transition state at –15.9 kcal mol^–1^ and this second step was also found to be coupled to B–H bond formation involving the Rh–H ligand, resulting in the formation of **6_Ph_**. Note that for clarity only the energy of **TS(11′′-6)2_Ph_** (the highest point in the rotation process) is indicated in Fig. 7; full details are provided in the ESI.† Overall this rotation process is kinetically favoured over P–B bond coupling towards **9_Ph_** by 7.2 kcal mol^–1^; moreover the formation of **6_Ph_** is also thermodynamically favoured over **9_Ph_** by 6.5 kcal mol^–1^.

In the light of these results phosphino-borane rotation in **Int(11''-9)2*t*_Bu_** was also assessed and was found to proceed with a low overall barrier of 5.8 kcal mol^–1^. This also involves two steps, although in this case the rotated phosphino-borane intermediate has the {P^*t*^Bu_2_} moiety adjacent to the Cp* ring. The resultant phosphido-borane, **6*t*_Bu_**, is located at –4.5 kcal mol^–1^ and so can readily revert to **Int(11′′-9)2*t*_Bu_** with a barrier of only 6.3 kcal mol^–1^, from which it can access the competing P–B bond coupling *via***TS(11′′-9)2*t*_Bu_**. The overall barrier for this (from **6*t*_Bu_**) is therefore only 12.2 kcal mol^–1^ and leads to first **9′*t*_Bu_** and then **9*t*_Bu_** in processes that are both significantly exergonic. The calculations therefore suggest rapid, but reversible formation of **6*t*_Bu_** before the thermodynamically favoured pathway to **9*t*_Bu_** takes over.^80^

The differences in the reaction profiles when R = ^*t*^Bu and Ph in Fig. 7 can be attributed to the greater steric encumbrance of the ^*t*^Bu system. This is particularly apparent for **6*t*_Bu_**, the formation of which is 31 kcal mol^–1^ less accessible than **6_Ph_**. The combination of the steric bulk derived from both the ^*t*^Bu substituents and the Cp* ligands is important in this: thus with H_3_B·PMe_2_H (*i.e.* exchanging Me for ^*t*^Bu) the formation of **6_Me_** becomes exergonic by 17.5 kcal mol^–1^, while the equivalent reaction of [RhCp(Me)(H_3_B·P^*t*^Bu_2_H)(PMe_3_)]^+^ (*i.e.* retaining the ^*t*^Bu substituents but exchanging Cp for Cp*) is downhill by 27.6 kcal mol^–1^. Similar arguments explain the greater relative stability of **9_Ph_** over **9*t*_Bu_**. In these systems, however, a PMe_3_ ligand has migrated from Rh onto B to be replaced by a much smaller hydride. The accumulative steric effect around the metal is therefore much less significant meaning that **9*t*_Bu_** is only 11.9 kcal mol^–1^ less accessible than **9_Ph_**; moreover, the formation of **9*t*_Bu_** becomes thermodynamically viable. Calculations also show that H_3_B·PHCy_2_ follows the pattern of behaviour computed for H_3_B·PHPh_2_, consistent with the observed formation of **7** in this case (see ESI† for full details).

### Comments on mechanism of dehydropolymerization of H_3_B·PRH_2_

**Scheme 12:**
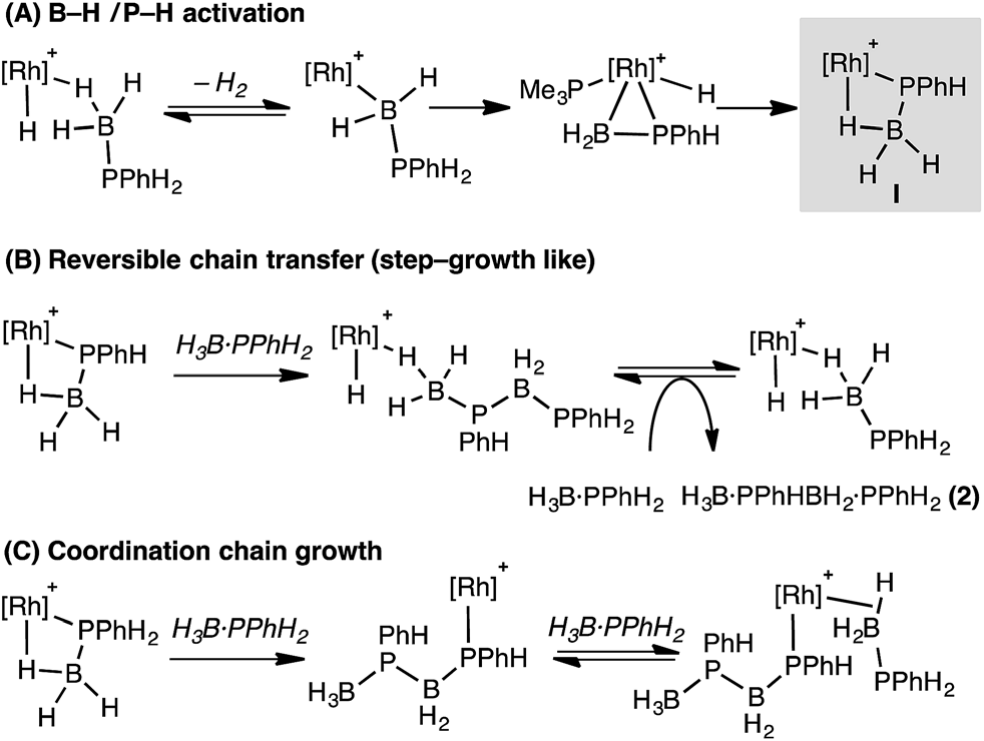
Suggested mechanisms for dehydropolymerization. [Rh] = Rh(PR_3_)Cp* (PR_3_ = PMe_3_ or PPhH_2_).

These studies suggest that the two likely limiting mechanisms for dehydropolymerization of H_3_B·PPhH_2_, step-growth-like *via* reversible chain transfer or coordination chain-growth, both likely flow from a common phosphido-borane intermediate (**I**, Scheme 12) that is an analogue of complex **6**. Stoichiometric, labelling and computational studies on secondary phosphine–borane systems suggest that such a species is likely formed from initial B–H activation of a phosphine–borane, followed by P–H transfer and rearrangement of a resultant hydrido phosphino-borane intermediate, modelled in this study as **Int(11′′-9′)**.

The observation of significant amounts of oligomer **2** at short reaction times, alongside the rapid consumption of H_3_B·PPhH_2_, point to reversible chain transfer (Scheme 12B) as a likely mechanism. That *M*_n_ is essentially unchanged with catalyst loading suggests this mechanism could be further modified by (observed) increasingly more P–B cleavage of the polymer at higher catalyst loadings. Based on our observations a coordination chain growth mechanism (Scheme 12C) appears less likely; as H_3_B·PPhH_2_ would be expected to be consumed gradually throughout the whole polymerization, **2** should not form in significant quantities, and *M*_n_ should increase with decreased catalyst loadings. If chain growth was occuring, slow propagation and faster termination/chain transfer steps would be required to account for our observations. We cannot discount a scenario where both mechanisms operate in ensemble, or there is a change from reversible chain transfer (step growth) to chain growth at lower [H_3_B·PPhH_2_]/higher [oligomer]. Related dual mechanisms have been discussed before with regard to polymer growth kinetics.^81^,^82^

**Scheme 13:**
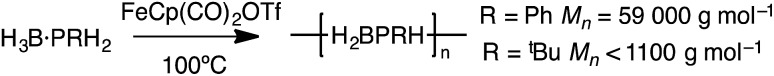
Manners and co-workers observations on polymer molecular weight and P–R substituent.

The contrast with Manners' FeCp(CO)_2_(OTf) system is interesting,^5^ as this shows coordination chain-growth-type polymerisation kinetics. We currently do not have a clear reason why this would be, although cationic Rh *versus* neutral Fe, and PR_3_*versus* CO ligands, are obvious electronic differences. Common to both Rh and Fe systems is the implication of β-B-agostic phosphido-borane complexes of the type [MCp(L)(PRHBH_3_)]^*n*+^, and we thus suggest that such species, as well as precursor metal-bound phosphino-boranes such as [MCp(L)(H)(PRHBH_2_)]^*n*+^, play a role in dehydropolymerization. As shown here the reactivity of such phosphino-borane intermediates is dependent on the steric bulk at phosphorus: for R = Ph phosphido-boranes are favoured thermodynamically, whereas for bulkier R = ^*t*^Bu this is the kinetic product, and the thermodynamic product arises from transfer of a metal bound ancillary ligand (PMe_3_) to the phosphino-borane. In this regard it is interesting to compare the differences in reported dehydropolymerization efficacy for FeCp(CO)_2_(OTf).^5^,^16^ For H_3_B·PPhH_2_ high molecular weight polymer is formed (*M*_n_ 59 000 g mol^–1^ in 24 h), whereas for H_3_B·P^*t*^BuH_2_ only short chain oligomers [H_2_BP^*t*^BuH]_*x*_ (*x* < 10) are formed after 172 h. Given our observations presented here we speculate that this may be due to deactivation routes that are modelled by complexes such as **9** when R = ^*t*^Bu (Scheme 13), that in turn arise from differing reactivity pathways of the corresponding phosphino-boranes.

## Conclusions

By choosing a system that can produce well-defined, moderate molecular weight, poly-[H_2_BPPhH]_*n*_, and is also designed to be latent low-coordinate, the intimate details of initial phosphine–borane activation in dehydropolymerization can be studied. Studies on model systems with secondary phosphine–boranes show that B–H activation precedes P–H activation, to give the kinetic product of a base-stabilised α-B-agostic boryl complex, subsequent P–H transfer, that operates *via* a hydrido-phosphino-borane species, leads to the observed phosphido-borane as the thermodynamic product. Together these three species offer many possibilities for pathways operating during dehydropolymerization.

Given the ambiguity related to the mechanism of dehydropolymerisation (step or chain growth-like) in this system we are reluctant to say definitively which mechanism is operating, but our general observations are consistent with those recently proposed mechanisms operating for FeCp(CO)_2_(OTf) and [Rh(Ph_2_P(CH_2_)_3_PPh_2_)]^+^,^5^,^19^ in as much that the proposed species that undergo the P–B bond forming event have M–P bonds (*i.e.* phosphido-boranes). Moreover, given that boryl, phosphino-borane and phosphido-boranes are all accessible they should all be considered as viable intermediates in catalytic dehydrocoupling and dehydropolymerization processes. This work also lends insight into related amine–borane dehydropolymerization in which amido–boranes, structurally related to **6** have been proposed as actual catalysts, and proposed to form *via* a N–H activation from a sigma-amine borane precursor,^31^,^83^ similar to that described in detail here for phosphido-boranes. The ubiquity of B-agostic interactions in the systems discussed here, whether α- or β-, also shows that such interactions also need to be explicitly considered when discussing the mechanism of dehydropolymerization. This mirrors olefin polymerisation, in which α- and β-agostic interactions play key roles in migratory insertion and polymerization processes.^84^,^85^ Such detail makes a further step towards fully understanding the mechanisms of group 13/15 dehydropolymerizations, and thus the further development of catalysts that can deliver tailored new polymeric materials.^2^

## Supplementary Material

Supplementary information

Supplementary information

Crystal structure data
