# Correction: Dehydrocoupling of phosphine–boranes using the [RhCp*Me(PMe_3_)(CH_2_Cl_2_)][BAr^F^_4_] precatalyst: stoichiometric and catalytic studies

**DOI:** 10.1039/c6sc90014c

**Published:** 2016-02-09

**Authors:** Thomas N. Hooper, Andrew S. Weller, Nicholas A. Beattie, Stuart A. Macgregor

**Affiliations:** a Department of Chemistry , Chemistry Research Laboratories , University of Oxford , Mansfield Road , Oxford , OX1 3TA , UK . Email: andrew.weller@chem.ox.ac.uk; b Institute of Chemical Sciences , Heriot Watt University , Edinburgh , EH14 4AS , UK . Email: S.A.Macgregor@hw.ac.uk

## Abstract

Correction for ‘Dehydrocoupling of phosphine–boranes using the [RhCp*Me(PMe_3_)(CH_2_Cl_2_)][BAr^F^_4_] precatalyst: stoichiometric and catalytic studies’ by Thomas N. Hooper *et al.*, *Chem. Sci.*, 2016, DOI: 10.1039/c5sc04150c.



## 

The authors regret that in the original article the structures of two of the compounds in Scheme 12 contained errors. A corrected version of Scheme 12 is presented herein, where a –PMe_3_ ligand has been removed from the third compound in part A and a hydrogen atom has been removed from the –PPhH group of the first compound in part C.

**Scheme 12:**
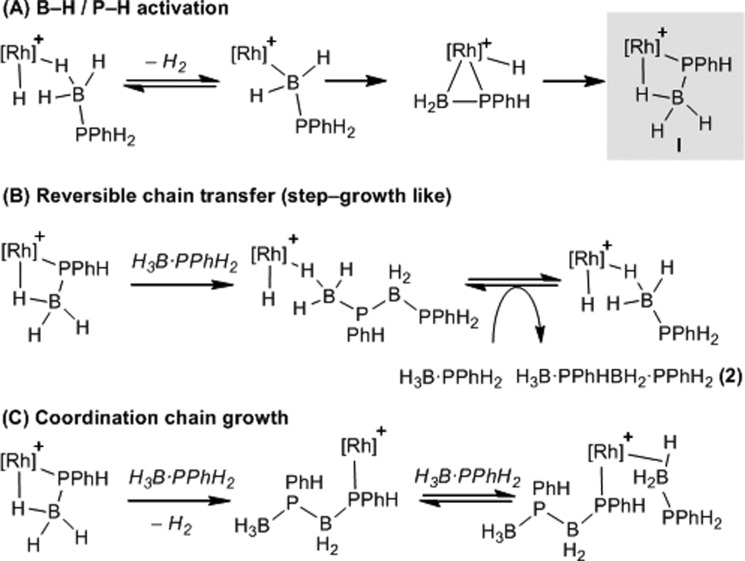
Suggested mechanisms for dehydropolymerization. [Rh] = Rh(PR_3_)Cp* (PR_3_ = PMe_3_ or PPhH_2_).

The Royal Society of Chemistry apologises for these errors and any consequent inconvenience to authors and readers.

