# Supplementary material for: Dehydrocoupling of phosphine–boranes using the [RhCp*Me(PMe3)(CH2Cl2)][BArF4] precatalyst: stoichiometric and catalytic studies
Source: Chem Sci. 2015 Dec 21;7(3):2414–26. doi: 10.1039/c5sc04150c (PMC6003611; doi:10.1039/c5sc04150c)
Supplement: Supplementary file 2 [file SC-007-C5SC04150C-s002.pdf]

Electronic Supplementary Material (ESI) for Chemical Science.
This journal is © The Royal Society of Chemistry 2015

Supporting Information for:

Dehydrocoupling of Phosphine-Boranes Using $\{RhCp^*(PR_3)\}^{2+}$. Stoichiometric Reactivity and Catalytic Studies

T.N. Hooper,[a] A.S. Weller,*[a] N.A. Beattie[b] and S.A. Macgregor[b]

a) Department of Chemistry, Inorganic Chemistry Laboratory, University of Oxford, Oxford, OX1 6QR, UK.

b) School of Chemical Sciences, Heriot Watt University, Edinburgh, EH14 4AS, UK.

General Experimental	**S-2**
Catalytic Reactions	**S-2**
Synthesis and Characterisation of New Compounds	**S-4**
Crystallography	**S-9**
Selected Spectra	**S-12**
Experimental References	**S-29**
Computational Details	**S-30**
Computational References	**S-30**
Breakdown of Energy Contributions	**S-31**
Functional Testing on **11** and its Isomers	**S-35**
Computed ^{11}B Chemical Shifts	**S-36**
Cartesian Coordinates and Energies for all Stationary Points	**S-37**

General Experimental

All manipulations, unless otherwise stated, were performed under an atmosphere of argon, using standard Schlenk and glove-box techniques. Glassware was oven dried at 130 °C overnight and flamed under vacuum prior to use. Dichloromethane, hexane and pentane were dried using a Grubbs type solvent purification system (MBraun SPS-800) and degassed by successive freeze-pump-thaw cycles.[1] CD_2Cl_2 was distilled under vacuum from CaH_2 and stored over 3 Å molecular sieves. NMR spectra were recorded on a Bruker AVD 500 MHz spectrometer at room temperature unless otherwise stated. ^{31}P and ^{11}B NMR spectra were referenced against 85% H_3PO_4 (external) and $Et_2O{\cdot}BF_3$ (external) respectively. Chemical shifts are quoted in ppm and coupling constants in Hz. ESI-MS were recorded on a Bruker MicrOTOF instrument. In all ESI-MS spectra there was a good fit to both the principal molecular ion and the overall isotopic distribution.

Gel permeation chromatography (GPC) was performed in collaboration with Dr T. Jurca and Prof. Ian Manners (University of Bristol) on a Viscotek RImax chromatograph, equipped with an automatic sampler, a pump, an injector and inline degasser. The columns were contained within an oven (35 °C) and consisted of styrene/divinyl benzene gels with pore sizes ranging from 500 Å to 100,000 Å. THF containing 1 % w/w [$^{n}Bu_4N$]Br and 1% v/v toluene was used as the eluent at a flow rate of 1.0 cm^3 min^{-1}. All samples analysed by GPC were dissolved in the eluent (2.5 and 5 mg cm^{-3}), stirred for 1 h at room temperature and passed through a membrane filter (200 nm pores) before analysis. The calibration was conducted using a series of monodisperse polystyrene standards obtained from Sigma-Aldrich.

$H_3B{\cdot}PPh_2H$ and $H_3B{\cdot}P^{t}Bu_2H$ were purchased from Sigma-Aldrich and used without further purification. All other phosphine-boranes were synthesised from the parent phosphine and $BH_3{\cdot}$THF using the same method as described for $H_3B{\cdot}PMe_3$.[2]
[$RhCp^*Me(PMe_3)(CH_2Cl_2)$][$BAr^F_4$] (**1**) was synthesised in a slight adaptation of the method described by Taw *et al*.[3] In a typical experiment [$RhCp^*(Cl)(Me)(PMe_3)$] (50.0 mg, 0.137 mmol) and Na[BAr^F_4] (121.5 mg, 0.137 mmol) were added to a Schlenk flask which was cooled in an ice bath. CH_2Cl_2 (10 cm^3) was added and the solution stirred for 30 minutes. The red solution was filtered to remove NaCl and the filtrate concentrated *in vacuo* to approximately 3 cm^3. The solution was carefully layered with pentane and stored at -18 °C overnight to form red crystalline material (**1**). The mother liquor was removed by cannula and the solid washed with pentane. The material was dried *in vacuo* for 10 minutes (the material darkened during this time) and isolated in the glove box (148 mg, 85%). The NMR spectra of the material matched that reported previously[3] and the solid was stored in the glove box freezer (-26 °C).

Catalytic Reactions

Catalytic Dehydrocoupling of $H_3B{\cdot}PPhH_2$ to form $[H_2BPPhH]_n$

In a typical experiment, $H_3B{\cdot}PPhH_2$ (1.0 cm^3, 1.0 M solution in toluene, 1.0 mmol) was added to a long thin Young's tube containing a stirrer bar and [$RhCp^*Me(PMe_3)(CH_2Cl_2)$][$BAr^F_4$] (**1**) (1 mol%, 12.8 mg, 0.01 mmol). The solid was dissolved to form a yellow solution. The solution was placed in an oil bath preheated to 100

°C and stirred for 72 h, with the tap open to a slow flow of argon. At the end of the reaction the solution was cooled and added by cannula to stirred hexane (100 cm^3) and a precipitate formed. The solvent was removed by filter cannula and the resulting polymer dried *in vacuo* to yield a pale yellow solid (26 mg).

^{1}H NMR (500 MHz, $CDCl_3$): δ 7.22 (br, Ar-**H**), 7.11 (br, Ar-**H**), 4.30 (br d, $^1J_{HP}$ = 346 Hz, $[H_2BPPh\mathbf{H}]_n$), 1.50 (v br, $[\mathbf{H}_2BPPhH]_n$).

^{31}P{^{1}H} NMR (202 MHz, $CDCl_3$): δ -49.5 (br, $[H_2B\mathbf{P}PhH]_n$).

^{31}P NMR (202 MHz, $CDCl_3$): δ -49.5 (br d, $^1J_{PH}$ = 346 Hz, $[H_2B\mathbf{P}PhH]_n$).

^{11}B{^{1}H} NMR (160 MHz, $CDCl_3$): δ -34.0 (v br, $[H_2\mathbf{B}PPhH]_n$).

Signals for the $[BAr^F{}_4]^-$ ion are visible in the ^{1}H and ^{11}B{^{1}H} NMR spectra originating from the precatalyst.

Table S1: GPC data for polymer samples prepared as above with catalyst (**1**) loadings of 1 and 5 mol%. The GPC data was measured at 2 polymer concentrations in THF solution.

Sample	Conc. (mg cm^{-3})	Component	M_n	M_w	PDI
1 mol% **1**, 72 h	2.5	Overall	1 200	24 00	20.4
		High molecular mass	15 000	33 000	2.2
		[a]Low molecular mass	300	700	2.2
	[b]5.0	Overall	1 400	16 000	6.6
5 mol% **1**, 72 h	2.5	Overall	1 800	10 000	5.7
		High molecular mass	13 000	19 000	1.5
		[a]Low molecular mass	900	1 500	1.7
	5	Overall	1 700	6 500	3.9
		High molecular mass	6 500	11 000	1.7
		[a]Low molecular mass	900	1 300	1.5

[a] *These values are below the GPC detection limit, so they have little value other than to reinforce that there is a reasonably high amount of low molecular mass product in the sample.*

[b] *For this sample, there was significant overlap between the low and high molecular mass components, and no clear distinction could be made.*

See figures S2-5 for polymer NMR spectroscopy, ESI-MS and GPC data.

$H_3B\cdot PPhHBH_2\cdot PPhH_2$ (2)

A toluene solution of $H_3B\cdot PPhH_2$ (0.50 cm^3, 1.0 M solution, 0.50 mmol) was transferred by cannula to a long, thin schlenk tube containing a small stirrer bar and $[RhCp^*Me(PMe_3)(CH_2Cl_2)][BAr^F{}_4]$ (**1**) (31.9 mg, 0.025 mmol, 5 mol%). Stirring dissolved the solid and formed a yellow solution and the portion of the tube containing solvent was placed in an oil bath preheated to 100 °C. After 1 h, the solution was cooled and the solvent removed *in vacuo*. Hexane (5 cm^3) was added with vigorous stirring and sonication, then the solvent was again evaporated to dryness to remove any traces of toluene. Hexane (20 cm^3) was added and the solution stirred vigorously to give a pale, almost colourless solution and a

yellow oil. The supernatant was carefully transferred by filter cannula to another schlenk tube, ensuring no yellow oil was transferred. The solvent was removed *in vacuo* to produce a pale yellow oil. A cold finger was introduced into the schlenk and this was cooled to -78 °C. The product was carefully heated (to approx. 50 °C) to remove traces of $H_3B·PPhH_2$ which sublimed onto the cold finger. Pure $H_3B·PPhHBH_2·PPhH_2$ was left in the schlenk tube as an oil and could be dissolved to form a standard solution for use.

^{1}H NMR (500 MHz, CD_2Cl_2): δ 7.66 (m, 5 H, Ar-**H**), 7.49 (m, 2 H, Ar-**H**), 7.37 (m, 3 H, Ar-**H**), 5.85 (dm, $^1J_{HP}$ = 405.6 Hz, 2 H, $H_3B·PHPhBH_2·P\mathbf{H}_2Ph$), 4.79 (dm, $^1J_{HP}$ = 343.3 Hz, 1 H, $H_3B·P\mathbf{H}PhBH_2·PH_2Ph$), 1.79 (v br m, 2 H, $H_3B·PHPhB\mathbf{H}_2·PH_2Ph$), 0.72 (br m, 3 H, $\mathbf{H}_3B·PHPhBH_2·PH_2Ph$).

$^{31}P\{^1H\}$ NMR (202 MHz, CD_2Cl_2): δ -51.4 (br, $H_3B·PHPhBH_2·\mathbf{P}H_2Ph$), -53.4 (br, $H_3B·\mathbf{P}HPhBH_2·PH_2Ph$).

$^{11}B\{^1H\}$ NMR (160 MHz, CD_2Cl_2): δ -36.5 (vt, $^1J_{BP}$ ≈ 70 Hz, $H_3B·PHPh\mathbf{B}H_2·PH_2Ph$), -38.9 (d, $^1J_{BP}$ ≈ 50 Hz, $H_3\mathbf{B}·PHPhBH_2·PH_2Ph$).

See figure S6 NMR spectroscopy data and figure S7 for detection of **2** during catalysis.

Catalytic Dehydrocoupling of $H_3B·PPh_2H$ to form $H_3B·PPh_2BH_2·PPh_2H$

In a typical experiment $[RhCp^*Me(PMe_3)(CH_2Cl_2)][BAr^F_4]$ (8.0 mg, 0.0063 mmol) and $H_3B·PPh_2H$ (25.0 mg, 0.125 mmol) were added to a high pressure NMR tube. Toluene (0.2 cm^3) was added and the tube shaken to dissolve the solids and form a yellow solution. The tube was placed in an oil bath preheated to 100 °C for the prescribed period of time. Before the NMR spectra were taken a further 0.2 cm^3 of toluene was added to the tube to ensure all compounds were in solution at room temperature.

See figure S9 NMR spectroscopy data and figure S10 for mercury poisoning experiment showing catalysis to be homogeneous.

Synthesis and Characterisation of New Compounds

$[RhCp^*(PPh_2·BH_3)(PMe_3)][BAr^F_4]$ (6)

A solution of $H_3B·PPh_2H$ (4.7 mg, 0.0235 mmol) in CH_2Cl_2 (2 cm^3) was added to $[RhCp^*Me(PMe_3)(CH_2Cl_2)][BAr^F_4]$ (30.0 mg, 0.0235 mmol) and effervescence was observed. The solution was stirred for 15 minutes to form a yellow solution. The solution was layered with pentane and stored at -18 °C. After 72 h the mother liquor was removed from the solid and the yellow crystals were washed with pentane and dried *in vacuo*. Isolation in the glove box yielded 24.7 mg of $[Cp^*Rh(PPh_2·BH_3)(PMe_3)][BAr^F_4]$ (76%).

^{1}H NMR (500 MHz, CD_2Cl_2): δ 7.73 (s, 8 H, BAr^F_4), 7.69 (m, 2 H, Ar-H), 7.57 (s, 4 H, BAr^F_4), 7.55 (m, 4 H, Ar-H), 7.45 (m, 4 H, Ar-H), 1.66 (s, 15 H, Cp*), 1.05 (d, $^2J_{HP}$ = 10.4 Hz, 9 H, PMe_3), 0.27 (v br, 2 H, Rh-H-B$\mathbf{H}_2$), -10.81 (br, 1 H, Rh-**H**-BH_2).

$^{31}P\{^1H\}$ NMR (MHz, CD_2Cl_2): δ 6.9 (br, $PPh_2{\cdot}BH_3$), -6.6 (dd, J_{PRh} = 139 Hz, J_{PP} = 22 Hz, PMe_3).

$^{11}B\{^1H\}$ NMR (160 MHz, CD_2Cl_2): δ -6.60 (s, $BAr^F{}_4$), -45.5 (br, Rh-H_3B).

ESI-MS (CH_2Cl_2, 60 ºC): positive ion: m/z = 501.13 (calc. 501.13, M^+-BH)

Microanalysis found: C 49.86%, H 3.50%. **Calculated for $C_{57}H_{49}B_2F_{24}P_2Rh$**: C 49.73%, H 3.59%.

See figure S11 for NMR spectroscopy experiment to show degenerate exchange of BH_3 protons in **6**. Selected spectra showing $\mathbf{6^D}$ formation are shown in figures S14 and S15.

[RhCp*($PCy_2{\cdot}BH_3$)(PMe_3)][$BAr^F{}_4$] (7)

A solution of $H_3B{\cdot}PPh_2H$ (2.5 mg, 0.0117 mmol) in CH_2Cl_2 (1 cm^3) was added to [RhCp*Me(PMe_3)(CH_2Cl_2)][$BAr^F{}_4$] (15.0 mg, 0.0117 mmol) and effervescence was observed. The solution was stirred for 5 minutes to form a bright yellow solution which was then cooled to -20 °C to prevent decomposition. The solution was layered with cold pentane and stored at -18 °C. After 72 h the mother liquor was removed from the solid and the yellow crystals were washed with pentane and dried *in vacuo*. Isolation in the glove box yielded 9.4 mg of [RhCp*($PCy_2{\cdot}BH_3$)(PMe_3)][$BAr^F{}_4$] (58%).

^1H NMR (500 MHz, CD_2Cl_2): δ 7.72 (s, 8 H, $BAr^F{}_4$), 7.56 (s, 4 H, $BAr^F{}_4$), 2.27 to 1.01 (m, 22 H, Cy-H), 1.91 (s, 15 H, Cp*), 1.41 (d, $^2J_{HP}$ = 10.1 Hz, 9 H, PMe_3), -0.41 (br, 2 H, Rh-H-B**H**$_2$), -11.42 (br, 1 H, Rh-**H**-BH_2).

$^{31}P\{^1H\}$ NMR (202 MHz, CD_2Cl_2): δ 19.5 (br, $PCy_2{\cdot}BH_3$), -8.8 (dd, J_{PRh} = 142 Hz, J_{PP} = 21 Hz, PMe_3).

$^{11}B\{^1H\}$ NMR (160 MHz, CD_2Cl_2): δ -6.61 (s, $BAr^F{}_4$), -42.8 (br, Rh-H_3B).

ESI-MS (CH_2Cl_2, 60 ºC): positive ion: m/z = 525.25 (calc. 525.25, M^+).

Microanalysis found: C 49.36%, H 4.39%. **Calculated for $C_{57}H_{61}B_2F_{24}P_2Rh$**: C 49.29%, H 4.43%.

[RhCp*H($PHPh_2$)(PMe_3)][$BAr^F{}_4$] (5)

[RhCp*($PPh_2{\cdot}BH_3$)(PMe_3)][$BAr^F{}_4$] (15.0 mg, 0.0109 mmol) was dissolved in 1,2-$C_6H_4F_2$ (0.4 cm^3) in a high-pressure NMR tube. The tube was degassed using the freeze/pump/thaw method (x 3) and placed under an atmosphere of H_2 (approximately 4 atm). The tube was mixed by inversion for 16 h and degassed again. The solvent was removed *in vacuo* and CD_2Cl_2 was added. A yellow solid (6.4 mg, 43%) was isolated by layering the dichloromethane solution with pentane and storage at -18 °C for 24 h.

^1H NMR (500 MHz, CD_2Cl_2): δ 7.73 (s, 8 H, $BAr^F{}_4$), 7.57 (s, 4 H, $BAr^F{}_4$), 7.59 to 7.46 (m, 6 H, Ar-**H**), 7.29 (m, 2 H, Ar-**H**) 7.18 (m, 1 H, Ar-**H**) 7.12 (m, 1 H, Ar-**H**), 6.64 (d, $^1J_{HP}$ = 377.8 Hz,

1 H, P**H**Ph_2), 1.74 (s, 15 H, Cp*), 1.34 (d, $^2J_{HP}$ = 10.4 Hz, 9 H, PMe_3), -12.24 (v dt, J_{HRh} = 22.6 Hz, J_{HP} = 35.1 Hz, 1 H, Rh-**H**).

$^{31}P\{^1H\}$ NMR (202 MHz, CD_2Cl_2): δ 20.2 (dd, J_{PRh} = 140 Hz, J_{PP} = 37 Hz, $PHPh_2$), -1.0 (dd, J_{PRh} = 133 Hz, J_{PP} = 37 Hz, PMe_3).

ESI-MS (CH_2Cl_2, 60 ºC): positive ion: m/z = 501.13 (calc. 501.13, M^+).

Microanalysis found: C 50.25%, H 3.42%. **Calculated for $C_{57}H_{48}BF_{24}P_2Rh$**: C 50.16%, H 3.54%.

[RhCp*H($PHCy_2$)(PMe_3)][BAr^F_4] (8)

[RhCp*(PCy_2·BH_3)(PMe_3)][BAr^F_4] (12.0 mg, 0.0087 mmol) was dissolved in CD_2Cl_2 (0.4 cm^3) in a high-pressure NMR tube and allowed to stand at room temperature for 48 h.

5 is formed as the major component of a mixture of compounds. We were unable to identify the other products but the NMR signals for **5** are reported below.

^1H NMR (500 MHz, CD_2Cl_2): δ 7.72 (s, 8 H, BAr^F_4), 7.56 (s, 4 H, BAr^F_4), 3.88 (dm, $^1J_{HP}$ = 336.3 Hz, 1 H, P**H**Cy_2), 2.25 to 1.16 (m, 22 H, Cy-H), 1.88 (s, 15 H, Cp*), 1.46 (d, $^2J_{HP}$ = 9.8 Hz, 9 H, PMe_3), -12.95 (ddd, J_{HRh} = 21.3 Hz, J_{HP} = 30.5 Hz, J_{HP} = 38.8 Hz, 1 H, Rh-**H**).

$^{31}P\{^1H\}$ NMR (202 MHz, CD_2Cl_2): δ 42.7 (br d, J_{PRh} ≈ 140 Hz, $PHCy_2$), -0.4 (br dd, J_{PRh} ≈ 130 Hz, J_{PP} ≈ 25 Hz, PMe_3).

ESI-MS (CH_2Cl_2, 60 ºC): positive ion: m/z = 513.23 (calc. 513.23, M^+).

[RhCp*H($P^tBu_2BH_2$·PMe_3)][BAr^F_4] (9)

A solution of H_3B·P^tBu_2H (3.8 mg, 0.0235 mmol) in CH_2Cl_2 (2 cm^3) was added to [RhCp*Me(PMe_3)(CH_2Cl_2)][BAr^F_4] (30.0 mg, 0.0235 mmol). The solution was stirred for 4 hours changing from a deep red to form a bright yellow solution. The solution was layered with pentane and stored at -18 °C. After 72 h the mother liquor was removed from the solid and the yellow crystals were washed with pentane and dried *in vacuo*. Isolation in the glove box yielded 17.4 mg of [RhCp*(PPh_2·BH_3)(PMe_3)][BAr^F_4] (55%).

^1H NMR (500 MHz, CD_2Cl_2): δ 7.73 (s, 8 H, BAr^F_4), 7.57 (s, 4 H, BAr^F_4), 2.01 (s, 15 H, Cp*), 1.45 (d, $^2J_{HP}$ = 11.3 Hz, 9 H, PMe_3), 1.31 (br m, 18 H, ^{t}Bu-H), 0.50 (br, 1 H, Rh-H-B**H**), -10.79 (br, 1 H, Rh-**H**-BH), -13.76 (br, 1 H, Rh-**H**).

$^{31}P\{^1H\}$ NMR (202 MHz, CD_2Cl_2): δ 54.8 (br, **P**$^tBu_2BH_2$·PMe_3), -7.8 (br, $P^tBu_2BH_2$·**P**Me_3).

$^{11}B\{^1H\}$ NMR (160 MHz, CD_2Cl_2): δ -6.61 (s, BAr^F_4), -45.4 (br vt, $^1J_{BP}$ ≈ 95 Hz, P^tBu_2**B**H_2·PMe_3).

ESI-MS (CH_2Cl_2, 60 ºC): positive ion: m/z = 473.21 (calc. 473.21, M^+).

Microanalysis found: C 47.68%, H 4.33%. **Calculated for $C_{53}H_{57}B_2F_{24}P_2Rh$**: C 47.62%, H 4.30%.

[RhCp*Me(PMe$_3$)(H$_3$B·P^{t}Bu$_2$H)][BAr$^F{}_4$] (10)

$H_3B·P^tBu_2H$ (1.9 mg, 0.0117 mmol) and [RhCp*Me(PMe$_3$)(CH$_2$Cl$_2$)][BAr$^F{}_4$] (15.0 mg, 0.0117 mmol) were dissolved in separate high pressure NMR tubes in CD_2Cl_2 (each in 0.2 cm^3). The solutions were cooled to -78 °C and the solution of $H_3B·P^tBu_2H$ transferred to the tube containing [RhCp*Me(PMe$_3$)(CH$_2$Cl$_2$)][BAr$^F{}_4$] by cannula. The solutions were mixed carefully to ensure they remained at -78 °C to produce a yellow solution. The cold tube was loaded into the NMR spectrometer which was precooled to -80 °C.

Due to the instability of [RhCp*Me(PMe$_3$)(H$_3$B·P^{t}Bu$_2$H)][BAr$^F{}_4$] at higher temperatures, we did not attempt isolation of this product. The NMR data is reported on the freshly prepared sample which was kept cold.

^{1}H NMR (500 MHz, CD$_2$Cl$_2$, -80 °C): δ 7.71 (s, 8 H, BAr$^F{}_4$), 7.52 (s, 4 H, BAr$^F{}_4$), 4.08 (d, $^1J_{HP}$ = 362.9 Hz, 1 H, P**H**^{t}Bu$_2$), 1.50 (s, 15 H, Cp*), 1.25 (d, $^2J_{HP}$ = 10.1 Hz, 9 H, PMe$_3$), 1.16 (m, 18 H, C-C**H**$_3$), 0.79 (m, 3 H, Rh-C**H**$_3$) -4.01 (br, 3 H, Rh-**H**$_3$B).

^{31}P{^{1}H} NMR (202 MHz, CD$_2$Cl$_2$, -80 °C): δ 35.4 (br s, PH^{t}Bu$_2$·BH$_3$), 8.3 (d, J_{PRh} = 149 Hz, PMe$_3$).

^{11}B{^{1}H} NMR (160 MHz, CD$_2$Cl$_2$, -80 °C): δ -6.8 (s, BAr$^F{}_4$), -44.8 (br, Rh-H$_3$B).

[RhCp*(H$_2$B·PH^{t}Bu$_2$)(PMe$_3$)][BAr$^F{}_4$] (11)

$H_3B·P^tBu_2H$ (1.9 mg, 0.0117 mmol) and [RhCp*Me(PMe$_3$)(CH$_2$Cl$_2$)][BAr$^F{}_4$] (15.0 mg, 0.0117 mmol) were dissolved in separate high pressure NMR tubes in CD_2Cl_2 (each in 0.2 cm^3). The solutions were cooled to -78 °C and the solution of $H_3B·P^tBu_2H$ transferred to the tube containing [RhCp*Me(PMe$_3$)(CH$_2$Cl$_2$)][BAr$^F{}_4$] by cannula. The solutions were mixed carefully to ensure they remained at -78 °C to produce a yellow solution. The solution was warmed to -40 °C to produce a very deep red solution. The cold tube was loaded into the NMR spectrometer which was precooled to -40 °C.

[RhCp*Me(PMe$_3$)(H$_3$B·P^{t}Bu$_2$H)][BAr$^F{}_4$] could be crystallised by layering the cold solution (-40 °C) with pentane and storing at -18 °C for 48 h. However, attempts to isolate these crystals resulted in discolouration of the dark red crystals and decomposition. The NMR data is reported on the freshly prepared sample which was kept cold. The molecular ion of **11** has an identical mass to that of **9**, since **11** converts to **9** in solution ESI-MS analysis could not definitively confirm the presence of **11** rather than its isomer **9**.

^{1}H NMR (500 MHz, CD$_2$Cl$_2$, -40 °C): δ 7.72 (s, 8 H, BAr$^F{}_4$), 7.54 (s, 4 H, BAr$^F{}_4$), 7.11 (br s, 1 H, Rh-H-B**H**), 4.68 (dm, $^1J_{HP}$ = 379.9 Hz, 1 H, P**H**^{t}Bu$_2$), 1.74 (s, 15 H, Cp*), 1.42 (d, $^2J_{HP}$ = 9.5 Hz, 9 H, PMe$_3$), 1.30 (d, $^3J_{HP}$ = 14.7 Hz, 9 H, ^{t}Bu), 1.20 (d, $^3J_{HP}$ = 11.9 Hz, 9 H, ^{t}Bu), 0.15 (s, C**H**$_4$), -12.76 (br d, $^1J_{HRh}$ = 37.8 Hz, 1 H, Rh-**H**-BH).

$^{31}P\{^1H\}$ NMR (202 MHz, CD_2Cl_2, -40 °C): δ 25.1 (br s, $PH^tBu_2{\cdot}BH_2$), -1.9 (d, J_{PRh} = 187 Hz, PMe_3).

$^{11}B\{^1H\}$ NMR (160 MHz, CD_2Cl_2, -40 °C): δ 47.6 (br, Rh-BH_2), -6.7 (s, $BAr^F{}_4$).

See figure S16 for following of reaction and formation of compounds **9**, **10** and **11** by NMR spectroscopy.

$[RhCp^*(H_2B{\cdot}PCy_3)(PMe_3)][BAr^F{}_4]$ (12)

$H_3B{\cdot}PCy_3$ (3.5 mg, 0.0117 mmol) and $[RhCp^*Me(PMe_3)(CH_2Cl_2)][BAr^F{}_4]$ (15.0 mg, 0.0117 mmol) were added to a high pressure NMR tube. CD_2Cl_2 (0.4 cm^3) was added and the solution mixed to form a very deep red solution.

The NMR data of $[RhCp^*(H_2B{\cdot}PCy_3)(PMe_3)][BAr^F{}_4]$ is reported on the freshly prepared sample. Attempts to isolate as a solid product **12** resulted in formation of an oil which decomposed upon being placed under vacuum. The molecular ion could not be observed by ESI-MS due to decomposition.

^1H NMR (500 MHz, CD_2Cl_2): δ 7.73 (s, 8 H, $BAr^F{}_4$), 7.57 (s, 4 H, $BAr^F{}_4$), 2.32 to 1.23 (m, 34 H, Cy-**H** and Rh-H-B**H**) 1.86 (s, 15 H, Cp*), 1.52 (d, $^2J_{HP}$ = 9.5 Hz, 9 H, PMe_3), -13.57 (br, 1 H, Rh-**H**-BH).

$^{31}P\{^1H\}$ NMR (202 MHz, CD_2Cl_2): δ 11.3 (br s, $PCy_3{\cdot}BH_2$), -1.3 (d, J_{PRh} = 189 Hz, PMe_3).

$^{11}B\{^1H\}$ NMR (160 MHz, CD_2Cl_2): δ 53.0 (br, Rh-BH_2), -6.6 (s, $BAr^F{}_4$).

$[RhCp^*H(H_3B{\cdot}PCy_3)(PMe_3)][BAr^F{}_4]$ (13)

$H_3B{\cdot}PCy_3$ (3.5 mg, 0.0117 mmol) and $[RhCp^*Me(PMe_3)(CH_2Cl_2)][BAr^F{}_4]$ (15.0 mg, 0.0117 mmol) were added to a high pressure NMR tube. CD_2Cl_2 (0.4 cm^3) was added and the solution mixed to form a very deep red solution. The solution was degassed using the freeze/pump/thaw method (x 3) and placed under a hydrogen atmosphere (~ 4 atm). The tube was vigorously shaken to produce an orange solution.

At room temperature an exchange process was occurring on the NMR timescale in which the terminal Rh-H is exchanging with the sigma-bound Rh-H_3B proton environments. The exchange was frozen out by cooling to -60 °C and the NMR data is reported at this temperature under an H_2 atmosphere.

^1H NMR (500 MHz, CD_2Cl_2, -60 °C): δ 7.72 (s, 8 H, $BAr^F{}_4$), 7.55 (s, 4 H, $BAr^F{}_4$), 4.56 (s, H_2), 1.86 to 1.10 (m, 33 H, Cy-**H**) 1.72 (s, 15 H, Cp*), 1.35 (d, $^2J_{HP}$ = 10.7 Hz, 9 H, PMe_3), -4.07 (br, 3 H, Rh-**H_3**B), -11.53 (dd, $^1J_{HRh}$ = 20.5 Hz, $^2J_{HP}$ = 42.4 Hz, 1 H, Rh-**H**).

$^{31}P\{^1H\}$ NMR (202 MHz, CD_2Cl_2, -60 °C): δ 18.7 (br s, $PCy_3{\cdot}BH_3$), 8.7 (d, J_{PRh} = 137 Hz, PMe_3).

$^{11}B\{^1H\}$ NMR (160 MHz, CD_2Cl_2, -60 °C): δ -6.8 (s, $BAr^F{}_4$), -45.3 (br, Rh-H_3B).

See figure S17 NMR spectroscopy data.

[RhCp*(H_2B·PCy_3)(PMe_3)(PPh_3)][$BAr^F{}_4$] (14)

12 was prepared as above and transferred via cannula to a NMR tube containing PPh_3 (3.1 mg, 0.0117 mmol) and the tube shaken to produce a yellow/orange solution. The NMR data is reported of the material generated in situ and attempts at isolation resulted in the formation of an oil.

^{1}H NMR (500 MHz, CD_2Cl_2): δ 7.74 (s, 8 H, $BAr^F{}_4$), 7.61 (m, 5 H, Ar-**H**), 7.57 (s, 4 H, $BAr^F{}_4$), 7.48 (m, 3 H, Ar-**H**), 7.41 (m, 5 H, Ar-**H**), 7.32 (m, 2 H, Ar-**H**), 2.42 (br m, 1 H, B-**H**), 2.08 to 1.24 (m, 33 H, Cy-**H**), 2.12 (s, 3 H, Cp*-C**H_3**), 1.79 (s, 3 H, Cp*-C**H_3**), 1.78 (s, 3 H, Cp*-C**H_3**), 1.66 (s, 3 H, Cp*-C**H_3**), 0.88 (d, $^2J_{HP}$ = 8.5 Hz, 6 H, P-C**H_3**), 0.72 (s, 3 H, Cp*-C**H_3**), 0.58 (d, $^2J_{HP}$ = 4.3 Hz, 3 H, P-C**H_3**), 0.23 (br m, 1 H, B-**H**).

^{31}P{^{1}H} NMR (202 MHz, CD_2Cl_2): δ 37.6 (dd, J_{PRh} = 196 Hz, J_{PP} = 40 Hz, PPh_3), 21.2 (br s, PCy_3·BH_2), -19.1 (dd, J_{PRh} = 182 Hz, J_{PP} = 40 Hz, PMe_3).

^{11}B{^{1}H} NMR (160 MHz, CD_2Cl_2): δ -6.6 (s, $BAr^F{}_4$), -39.5 (br, Rh-BH_2).

See figure S18 NMR spectroscopy data.

Crystallography

X-ray crystallography data for all compounds was collected on an Enraf Nonius Kappa CCD diffractometer using graphite monochromated Mo Kα radiation (λ = 0.71073 Å) and a low-temperature device [150(2) K];[4] data were collected using COLLECT, reduction and cell refinement was performed using DENZO/SCALEPACK.[5] The structures were solved by direct methods (SHELXS-97) and refined by full matrix least squares using SHELXL-14.[6] Crystallographic data have been deposited with the Cambridge Crystallographic Data Centre under **CCDC** 1423368-70. These data can be obtained free of charge from the Cambridge Crystallographic Data Centre via www.ccdc.cam.ac.uk/data_request/cif.

Table S2: Crystallographic data

Compound	**6**	**7**	**9**
CCDC No.	1423368	1423369	1423370
Formula	$C_{57}H_{49}B_2F_{24}P_2Rh$	$C_{57}H_{61}B_2F_{24}P_2Rh$	$C_{53}H_{57}B_2F_{24}P_2Rh$
M	1376.43	1388.52	1336.45
Crystal System	Monoclinic	Monoclinic	Triclinic
Space group	*P* 21/c	*P* 21/c	*P*-1
T [K]	150(2)	150(2)	150(2)
a [Å]	12.721(3)	13.155(3)	12.494(3)
b [Å]	26.545(5)	13.139(3)	13.356(3)
c [Å]	18.270(4)	35.677(7)	18.152(4)
α [°]	90	90	102.66(3)
β [°]	107.20(3)	95.41(3)	92.36(3)
γ [°]	90	90	90.85(3)
V [Å^3]	5894(2)	6139(2)	2952.0(11)
Z	4	4	2
Density [g cm^{-3}]	1.551	1.502	1.504
μ (mm^{-1})	0.458	0.440	0.454
θ range [deg]	$5.091 \leq \theta \leq 27.459$	$5.128 \leq \theta \leq 27.491$	$5.109 \leq \theta \leq 27.497$
Reflns collected	25072	26843	23746
R_{int}	0.0666	0.1310	0.0365
Completeness	99.1%	99.0 %	99.1 %
No. of data/restr/param	13368/543/907	13953/346/851	13438/977/1019
R_1 [$I > 2\sigma(I)$]	0.0499	0.0625	0.0571
wR_2 [all data]	0.1224	0.1869	0.1738
GoF	0.989	0.853	0.867
Largest diff. pk and hole [eÅ^{-3}]	0.80, -0.61	1.58, -0.52	1.09, -0.80

Structure of Boryl Complex [RhCp*($H_2B \cdot PH^tBu_2$)(PMe_3)][BAr^F_4] (11)

Red block crystals were grown from a solution of **11** which was kept at -20 °C and layered with pentane and stored at -18 °C for 48 hours. Crystals were kept cold, isolated and quickly transferred to the cryostream of the diffractometer. The crystals diffracted strongly at low angle but very weakly at high angle. Analysis of the complete data set did not suggest signs of twinning or other problems so the poor quality data appears to be due to poor crystal quality. The data presented below is presented for connectivity purposes and bond length and angle data is not presented.

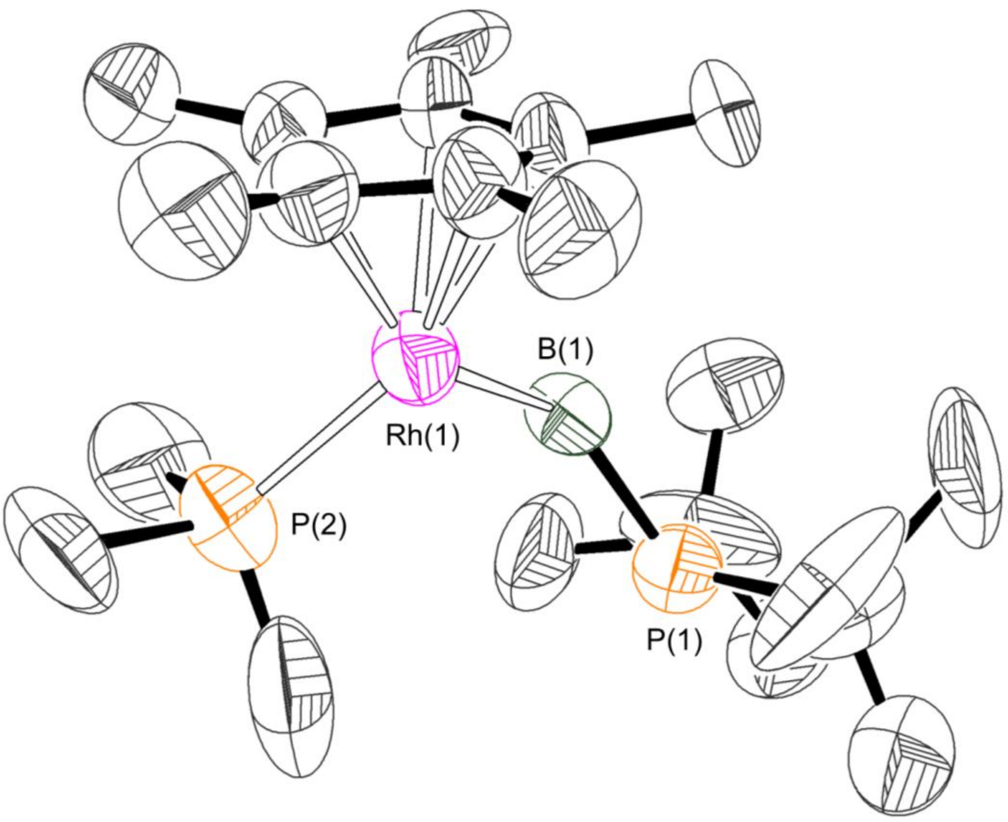

Figure S1: X-ray molecular structure of [RhCp*($H_2B{\cdot}P^tBu_2H$)(PMe_3)][$BAr^F{}_4$] (**11**). [$BAr^F{}_4$]$^-$ anion and hydrogen atoms omitted.

Single crystal X-ray diffraction data for **7**: $C_{53}H_{57}B_2F_{24}P_2Rh$, M = 1336.45, monoclinic, a = 12.669(3), b = 25.975(5), c = 19.200(4) Å, β = 105.48(3)°, V = 6089(2) Å^3, T = 150(2) K, space group $P2_1/c$ (no. 14), Z = 4, reflections measured 27088, 13896 unique (R_{int} = 0.0649) which were used in all calculations. The final $R1$ was 0.1997 [for 7154 reflections with $I>2\sigma(I)$] and the final $wR(F_2)$ was 0.5225 (all data).

Selected Spectra

Polymer $[H_2BPPhH]_n$

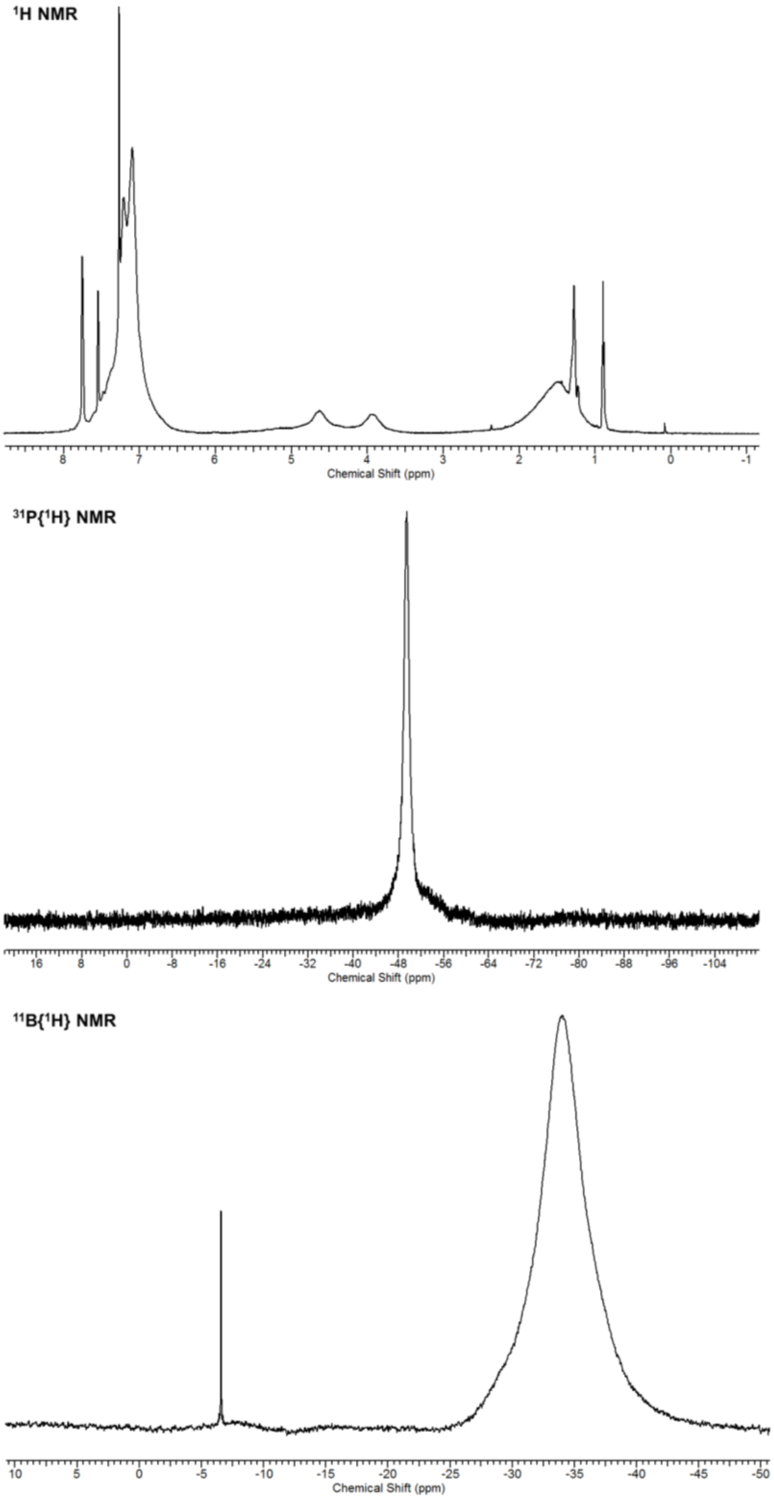

Figure S2: NMR spectra of isolated $[H_2BPPhH]_n$. Top, ^1H NMR spectrum in $CDCl_3$; middle, $^{31}P\{^1H\}$ NMR spectrum in $CDCl_3$; bottom, $^{11}B\{^1H\}$ NMR spectrum in $CDCl_3$.

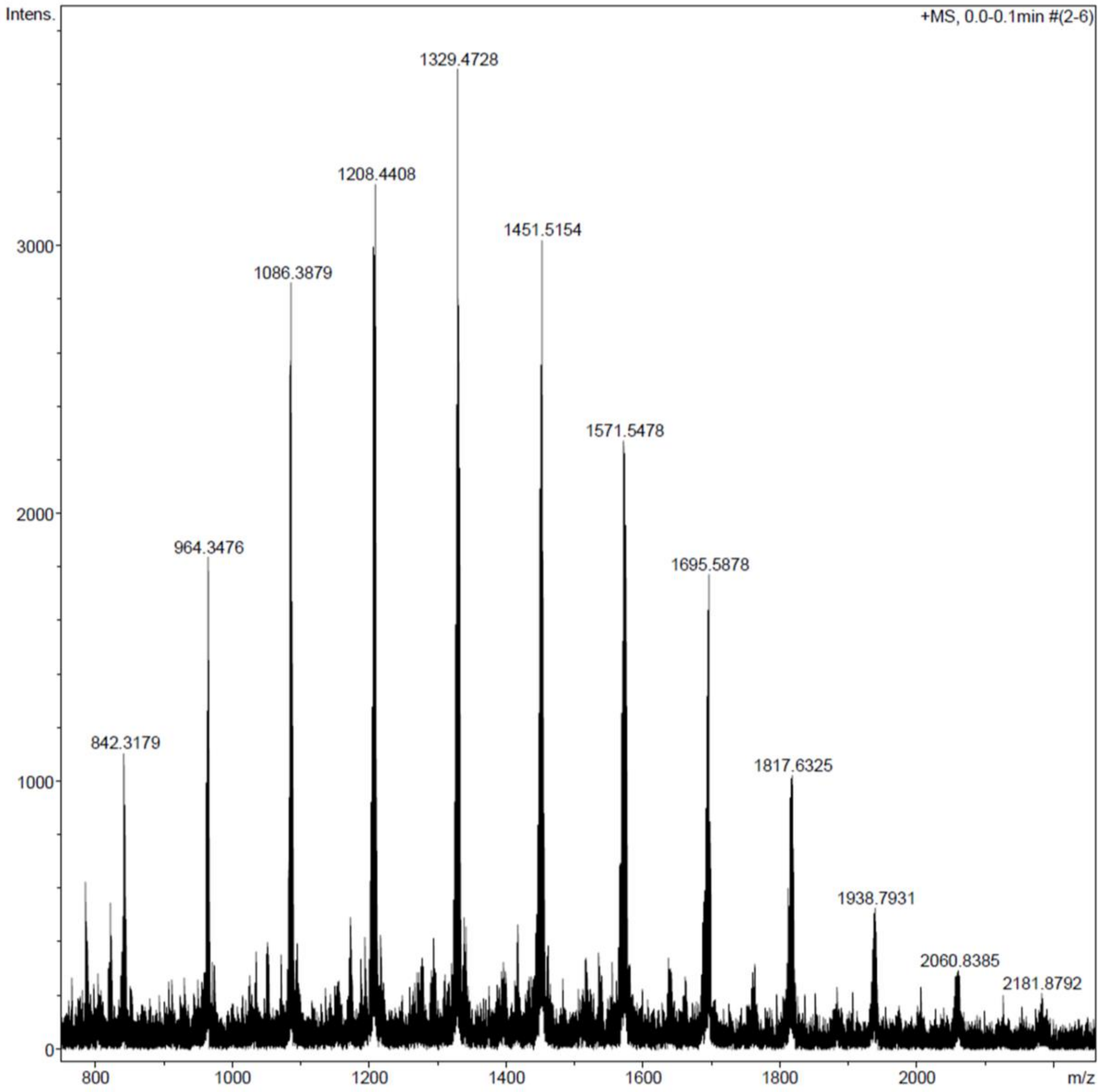

Figure S3: ESI-MS spectrum for $[H(PPhHBH_2)_nPPhH_2]^+$ (n = 6-17) sample in toluene. Sample prepared with 1 mol% **1** (72 h, 100 °C). Polymer repeat units of *m*/*z* 122.1 can be observed.

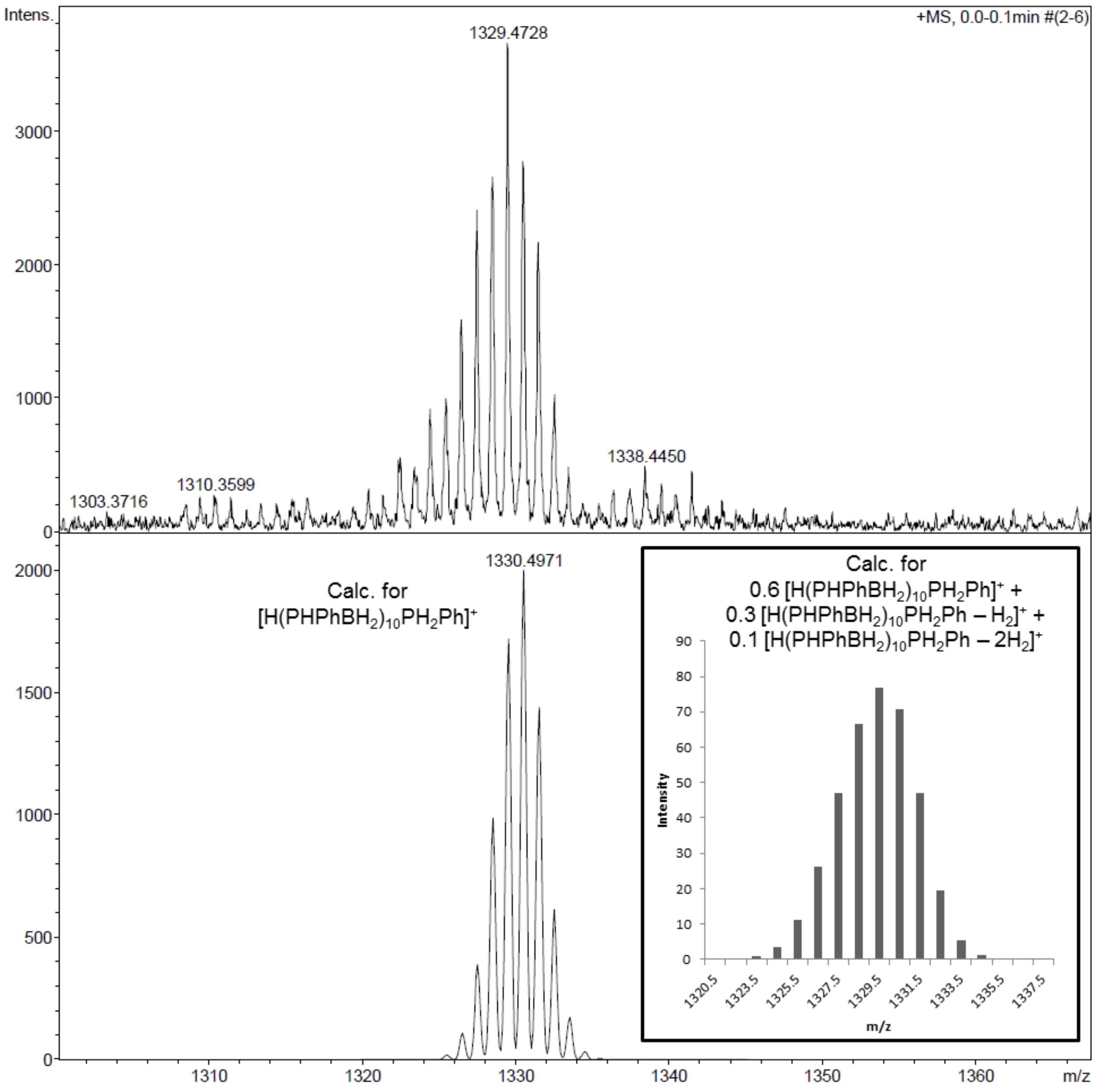

Figure S4: Magnification of ESI-MS spectrum for $[H(PPhHBH_2)_{10}PPhH_2]^+$ sample in toluene (top) and calculated isotopic pattern (bottom). The calculated isotopic pattern does not match exactly and the inset shows a calculated pattern with overlapping contributions from $[H(PPhHBH_2)_{10}PPhH_2]^+$, $[H(PPhHBH_2)_{10}PPhH_2 – H_2]^+$ and $[H(PPhHBH_2)_{10}PPh\ H_2 – 2H_2]^+$ which is a better fit for the experimental data.

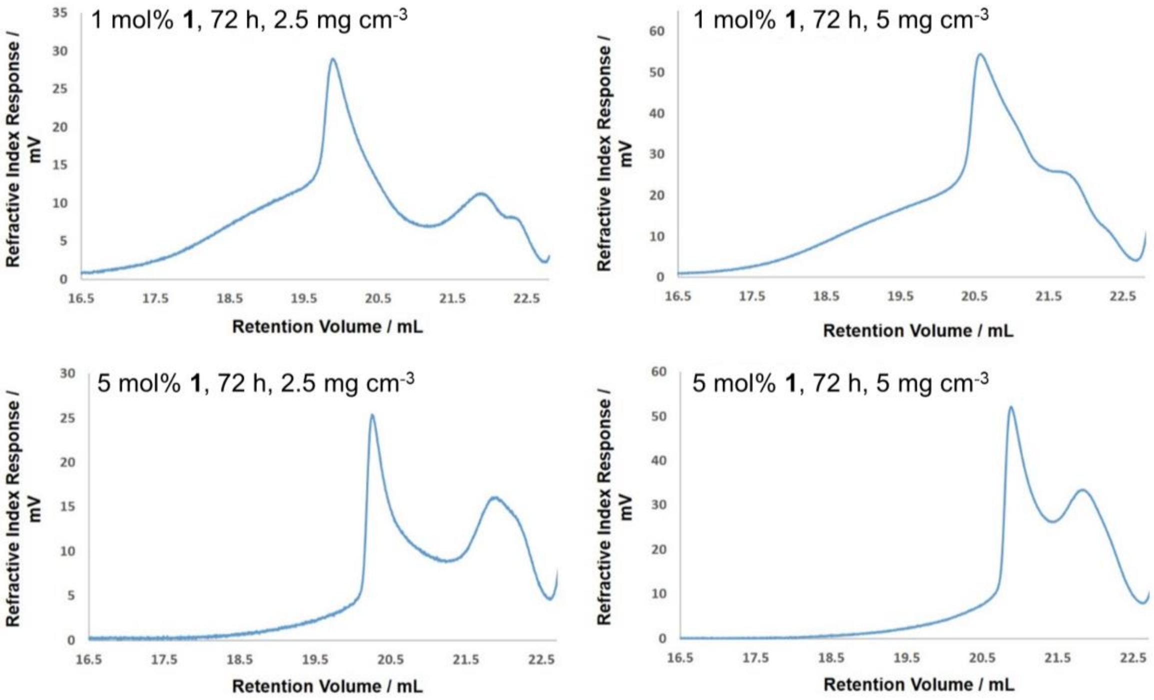

Figure S5: GPC traces for $[H_2BPPhH]_n$ samples in THF. Samples prepared with 1 mol% **1** (top) and 5 mol% **1** (bottom) are shown. The samples were measured at 2 polymer concentrations, 2.5 mg cm^{-3} (left) and 5 mg cm^{-3} (right). The data quality is relatively low due to the high polydispersity of the samples.

$H_3B{\cdot}PPhHBH_2{\cdot}PPhH_2$ (2)

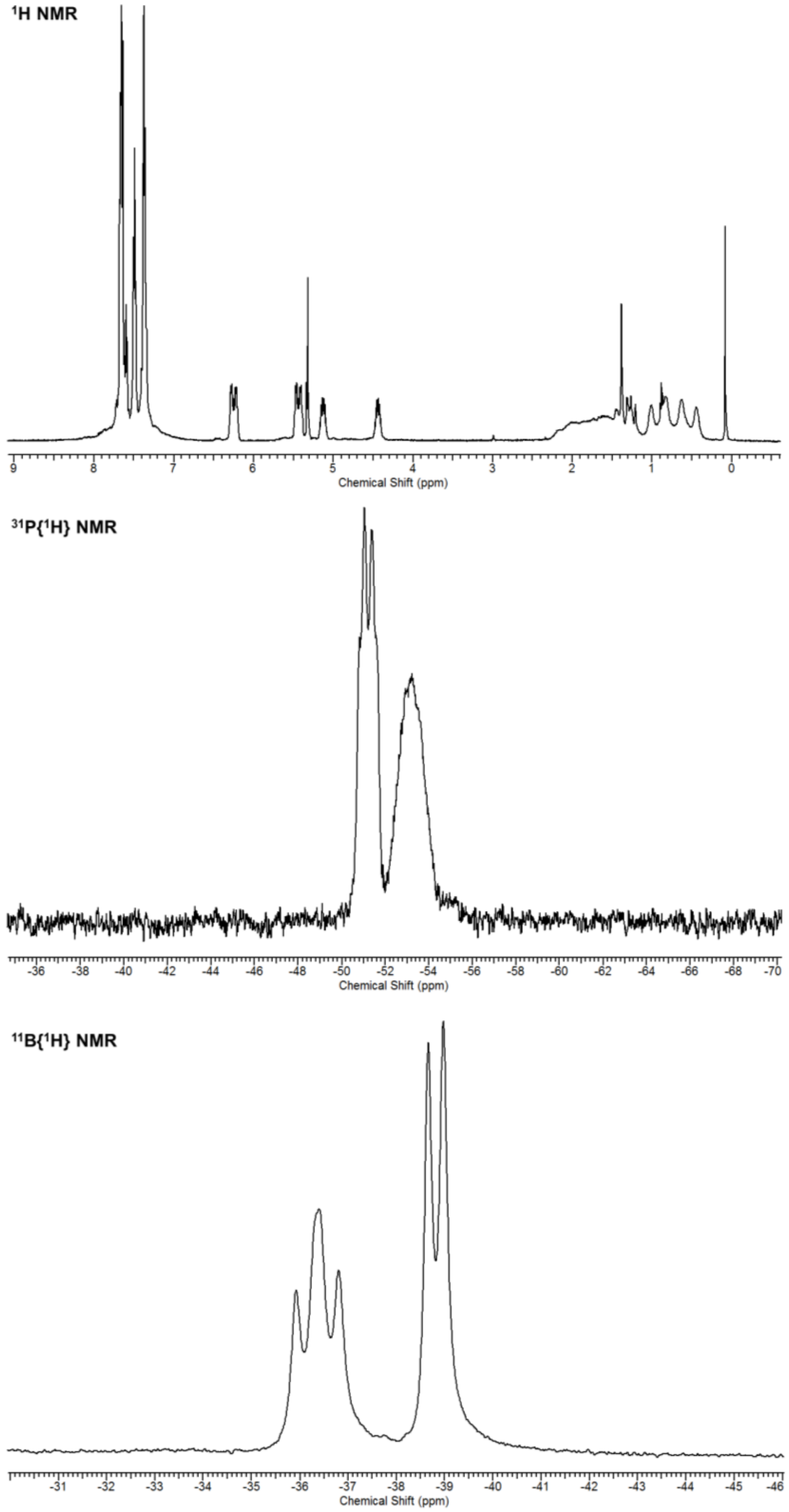

Figure S6: NMR spectra of isolated $H_3B{\cdot}PPhHBH_2{\cdot}PPhH_2$. Top, ^{1}H NMR spectrum in CD_2Cl_2; middle, $^{31}P\{^{1}H\}$ NMR spectrum in CD_2Cl_2; bottom, $^{11}B\{^{1}H\}$ NMR spectrum in CD_2Cl_2.

Detection of $H_3B{\cdot}PPhHBH_2{\cdot}PPhH_2$ (2) – Reaction Between $H_3B{\cdot}PPhH_2$ and 1 mol% 1 after 1 h

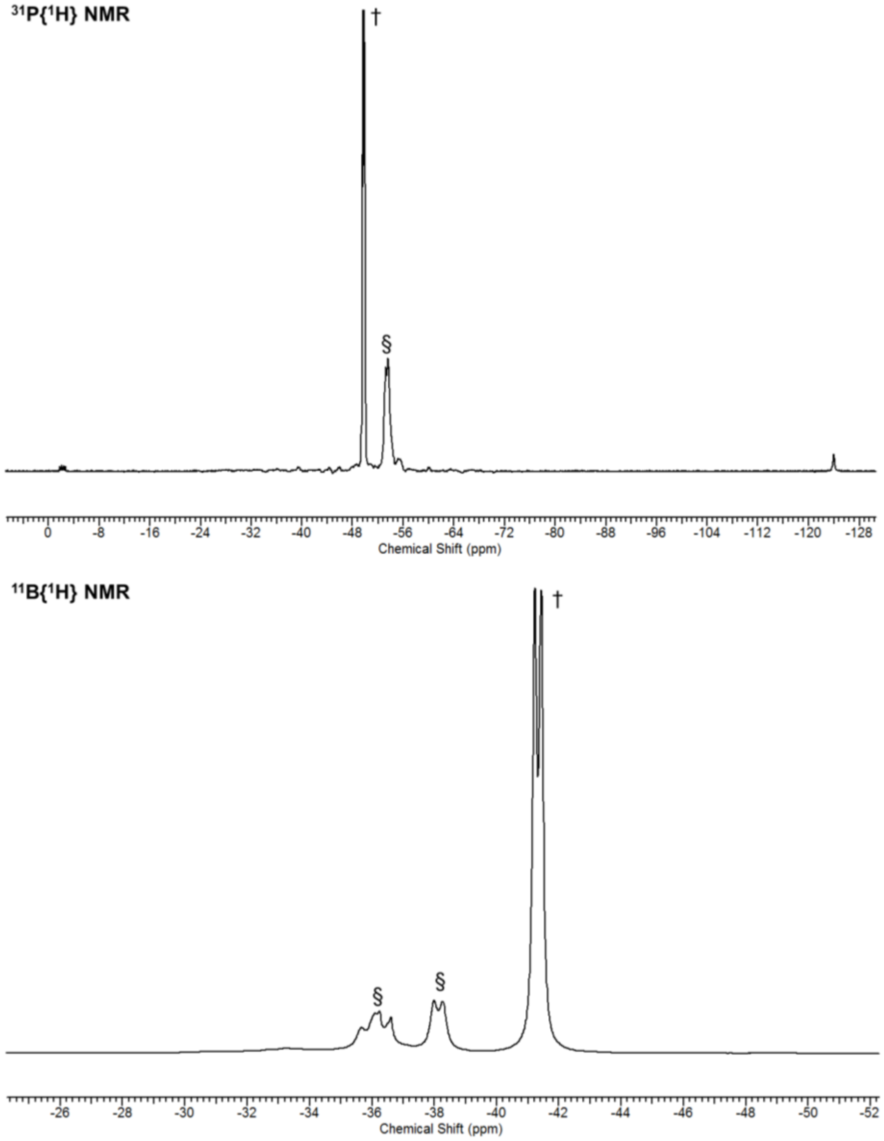

Figure S7: NMR spectra of reaction mixture of $H_3B{\cdot}PPhH_2$ and 1 mol% **1** in toluene after heating for 1 h at 100 °C. Top, $^{31}P\{^1H\}$ NMR spectrum; bottom, $^{11}B\{^1H\}$ NMR spectrum. Signals for $H_3B{\cdot}PPhH_2$ labelled † and signals for $H_3B{\cdot}PPhHBH_2{\cdot}PPhH_2$ labelled §. Ratio of $H_3B{\cdot}PPhHBH_2{\cdot}PPhH_2$ to $H_3B{\cdot}PPhH_2$ approximately 1:6 from $^{11}B\{^1H\}$ NMR integration.

Reaction of $[H_2BPPhH]_n$ with Precatalyst 1 to show Polymer Scission

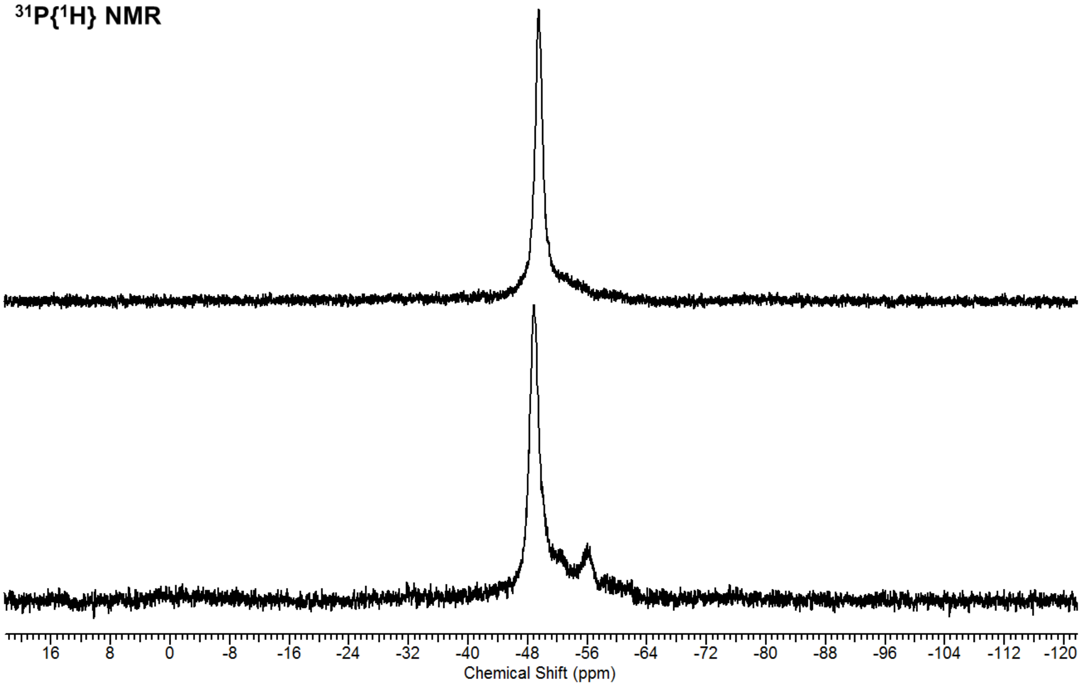

Figure S8: Top, $^{31}P\{^1H\}$ NMR spectrum of isolated $[H_2BPPhH]_n$. Bottom $^{31}P\{^1H\}$ NMR spectrum of $[H_2BPPhH]_n$ after heating with 5 mol% **1** in toluene (100 °C, 24 h).

Catalytic Dehydrocoupling of $H_3B{\cdot}PPh_2H$

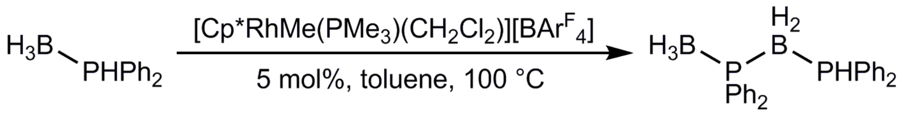

Scheme S1: Catalytic dehydrocoupling of $H_3B{\cdot}PPh_2H$ with 5 mol% **1**.

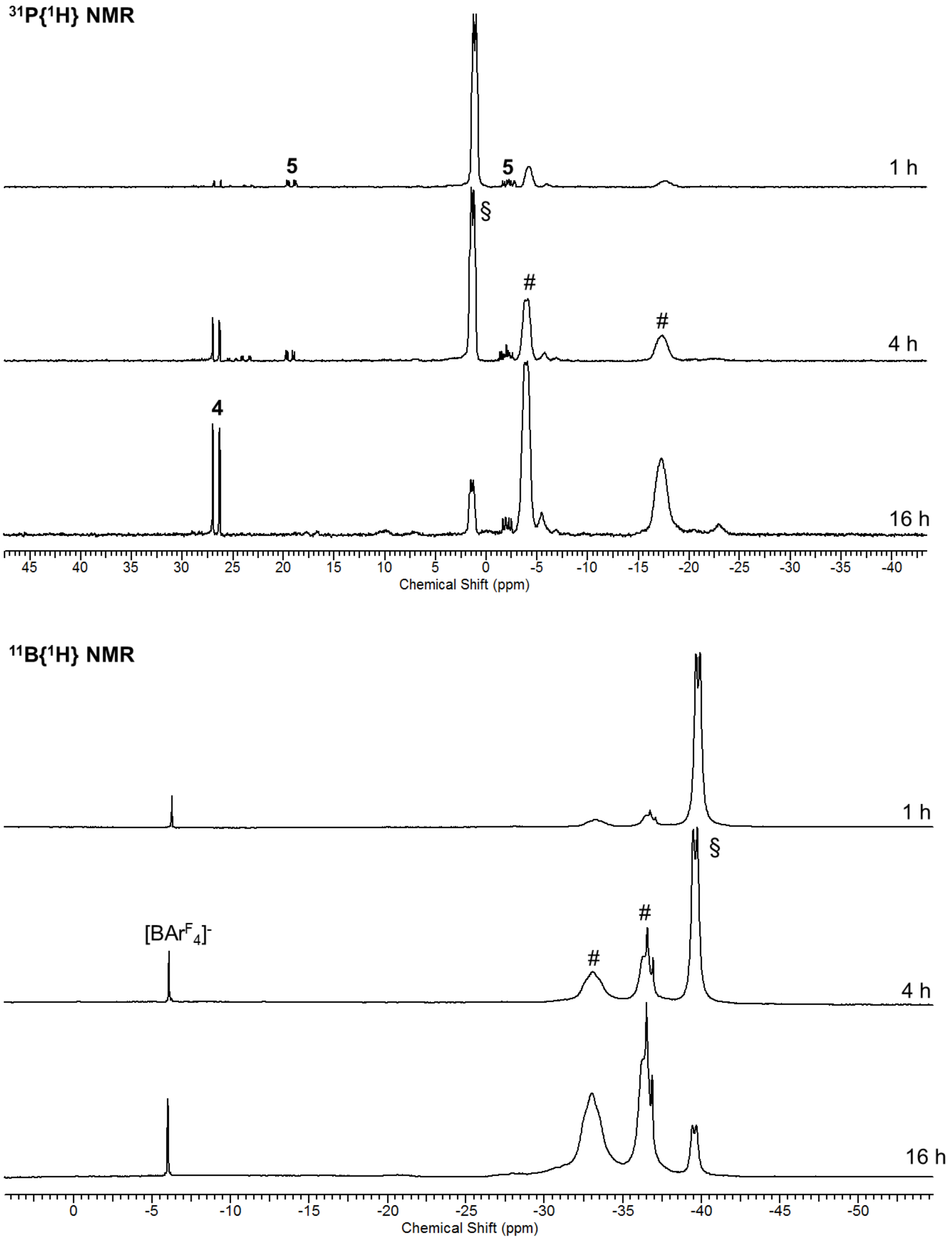

Figure S9: Top $^{31}P\{^1H\}$ NMR; and bottom $^{11}B\{^1H\}$ NMR spectra of catalytic dehydrocoupling of $H_3B{\cdot}PPh_2H$ with 5 mol% **1** in toluene at 100 °C measured at 1, 4 and 16 h. Signals corresponding to compounds **4** and **5** are labelled. Signals corresponding to $H_3B{\cdot}PPh_2H$ (§) and $H_3B{\cdot}PPh_2BH_2{\cdot}PPh_2H$ (#) are labelled.

Addition of Mercury Catalytic Dehydrocoupling of $H_3B{\cdot}PPh_2H$

The reaction was repeated as in scheme S1 with NMR spectra taken to ensure the reaction had begun. Mercury metal (approx. 0.2 cm^3) was added to one sample after 4 hours and the reaction was stirred for a further 20 h at 100 °C and NMR spectra taken of the control and mercury added reactions. The addition of mercury had no measurable effect on the rate of dehydrocoupling.

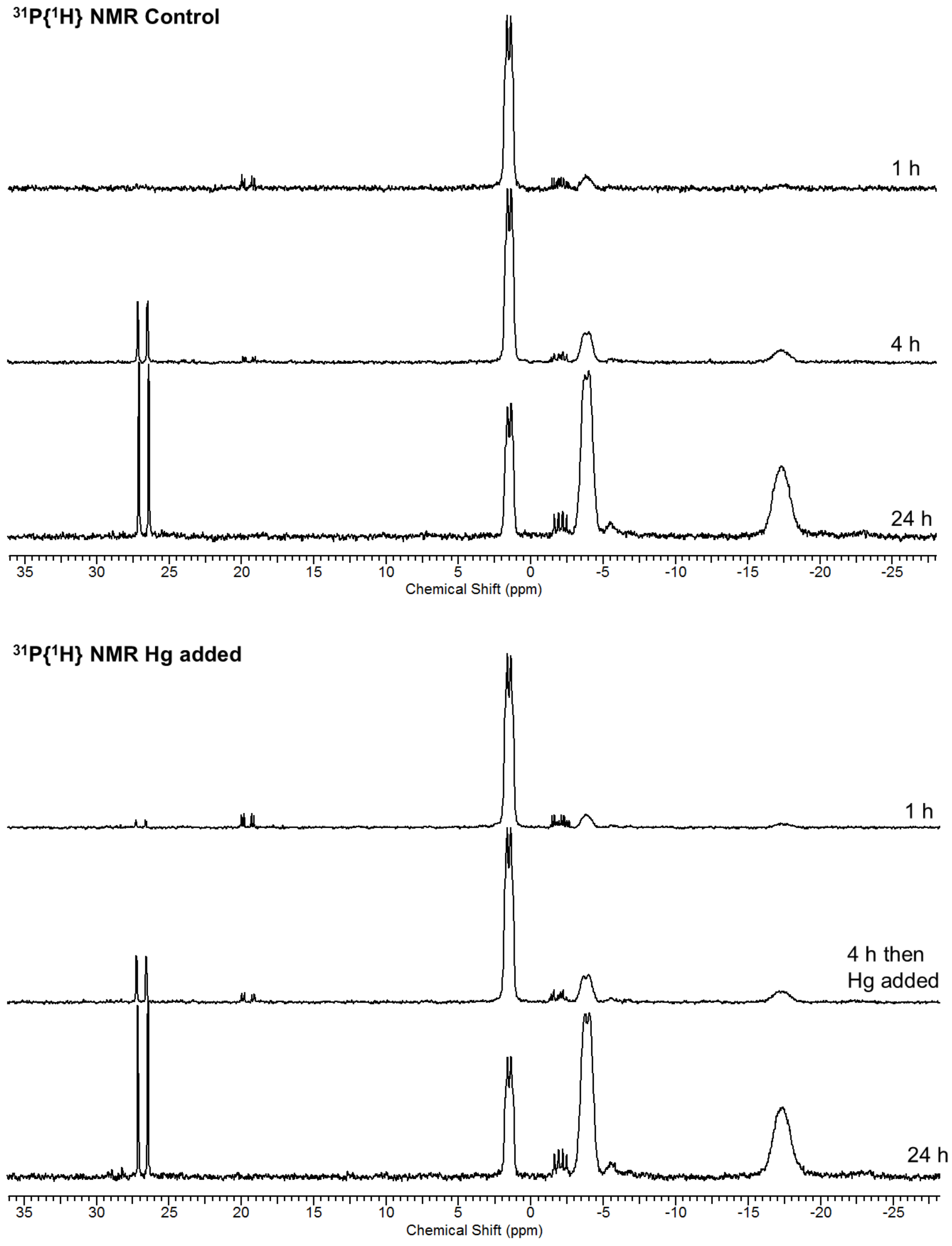

Figure S10: $^{31}P\{^1H\}$ NMR spectra of catalytic dehydrocoupling of $H_3B{\cdot}PPh_2H$ with 5 mol% **1** in toluene at 100 °C measured at 1, 4 and 24 h. Top, control reaction and bottom, reaction with mercury added after 4 h.

$^{1}H\{^{11}B\}$ NMR Steady State Spin Saturation Experiment to Determine Degenerate Exchange in $[RhCp^*(PPh_2{\cdot}BH_3)(PMe_3)][BAr^F{}_4]$ (6)

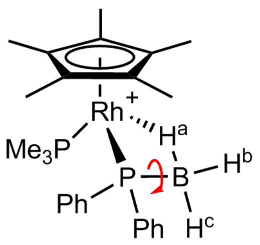

Scheme S2: Degenerate exchange of B-H environments in **6**. $[BAr^F{}_4]^-$ not shown.

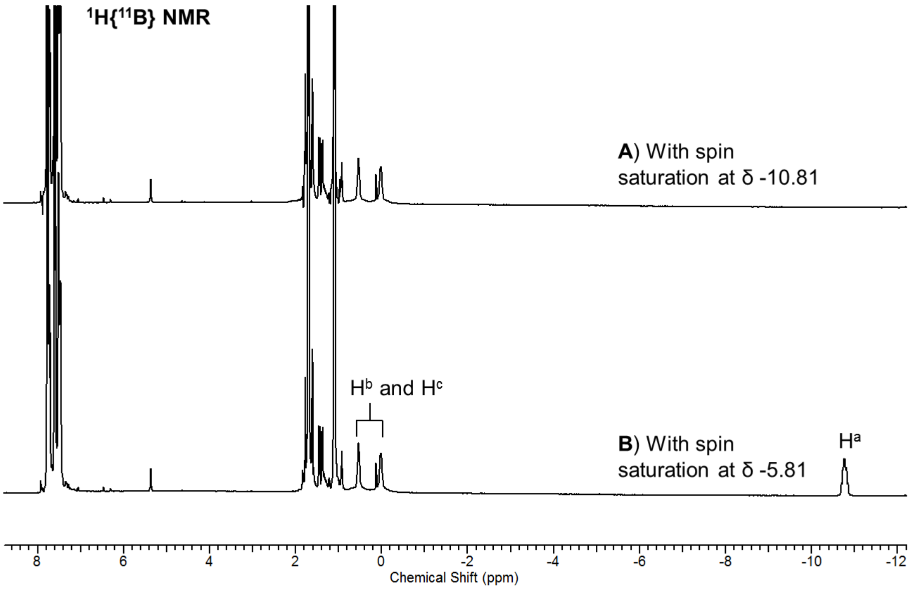

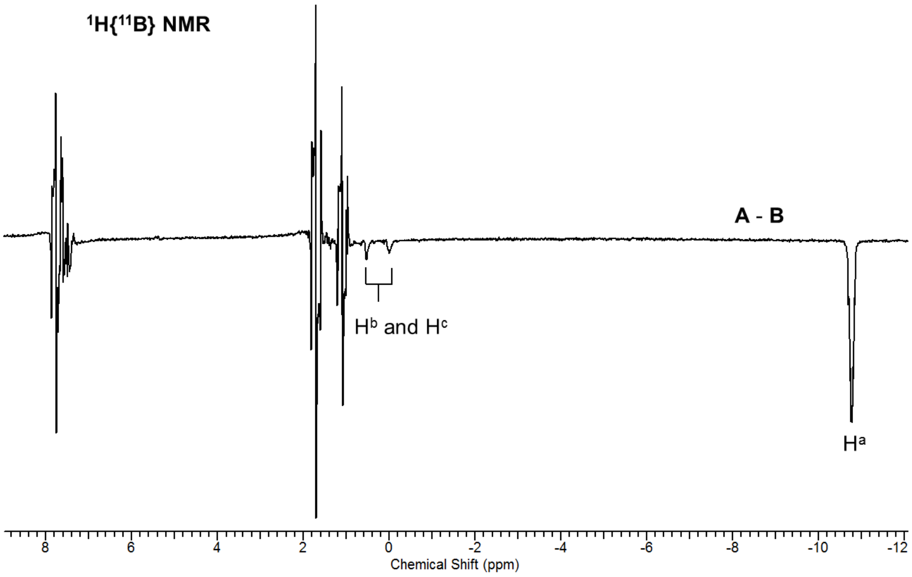

Figure S11: Top, $^{1}H\{^{11}B\}$ NMR spectra **6** in CD_2Cl_2 at 25 °C with spin saturation at δ -10.81 and -5.81. Bottom, subtraction of the spectra with negative peaks for H^a, H^b and H^c. Peaks with positive and negative regions result from subtraction errors of large peaks.

Decomposition of 6 to form 5 – Boron Containing Products

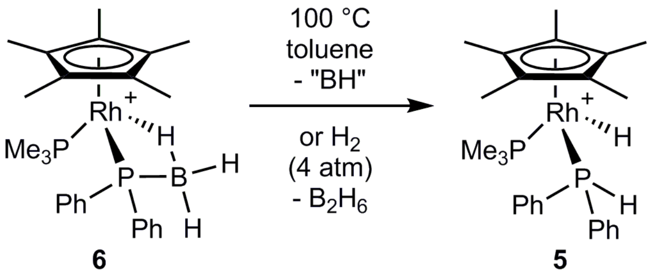

Scheme S3: Decomposition of **6** to form **5**. $[BAr^F_4]^-$ anions not shown.

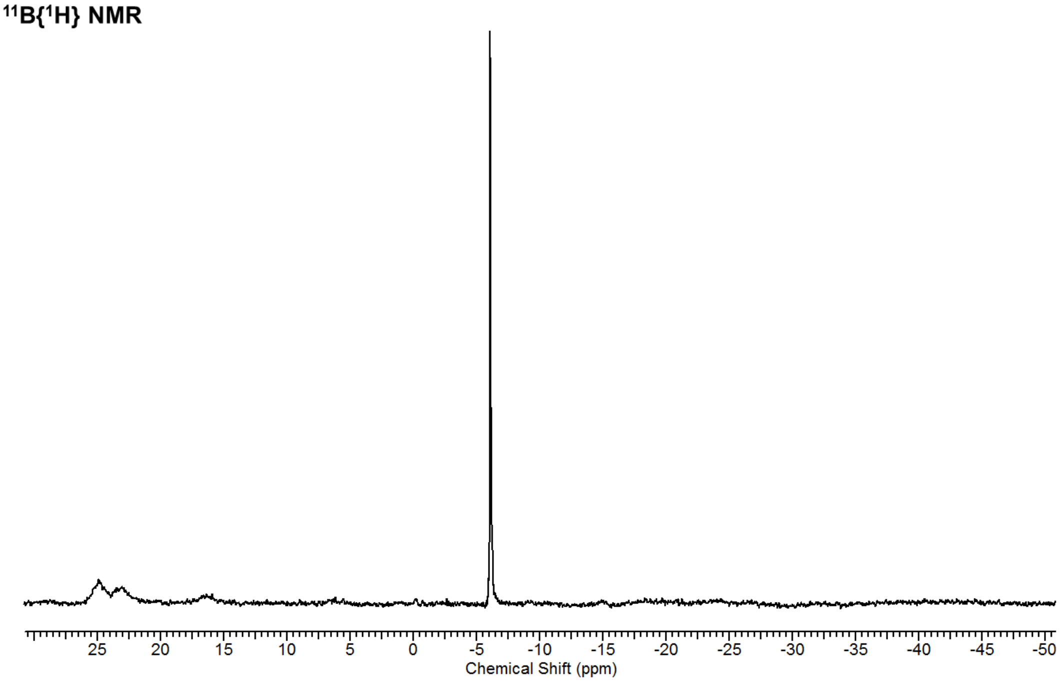

Figure S12: $^{11}B\{^1H\}$ NMR spectrum of the reaction mixture after heating **6** in toluene at 100 °C for 4 h to form **5**.

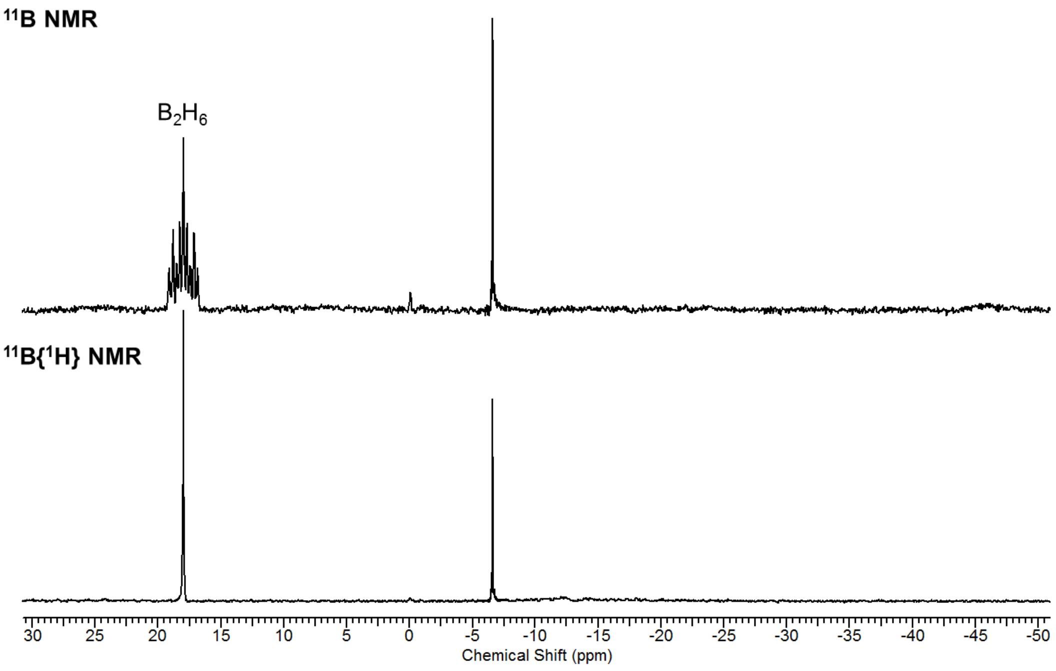

Figure S13: ^{11}B (top) and $^{11}B\{^{1}H\}$ NMR (bottom) spectra of the reaction mixture after mixing **6** with H_2 (4 atm) in fluorobenzene at 25 °C for 16 h to form **5**. Product characterised as B_2H_6.[7]

NMR spectra from Reaction of 1 with $D_3B·PPh_2H$

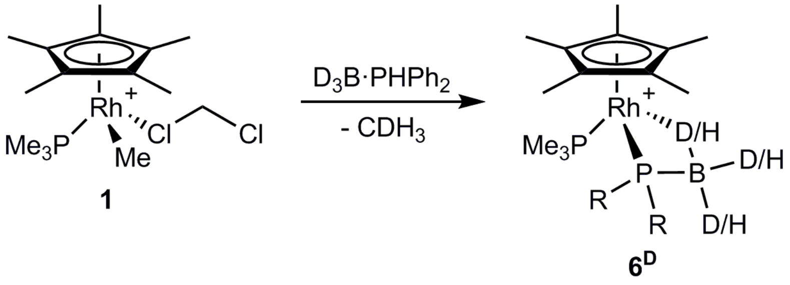

Scheme S4: Reaction of **1** with $D_3B·PHPh_2$ to form $\mathbf{6^D}$ and CDH_3. $[BAr^F{}_4]^-$ anions not shown.

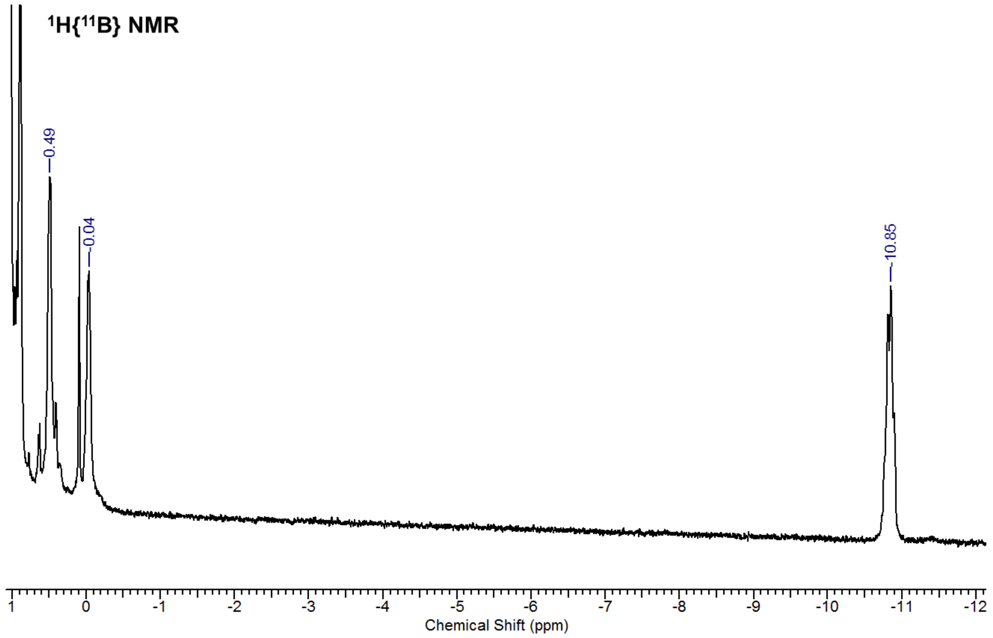

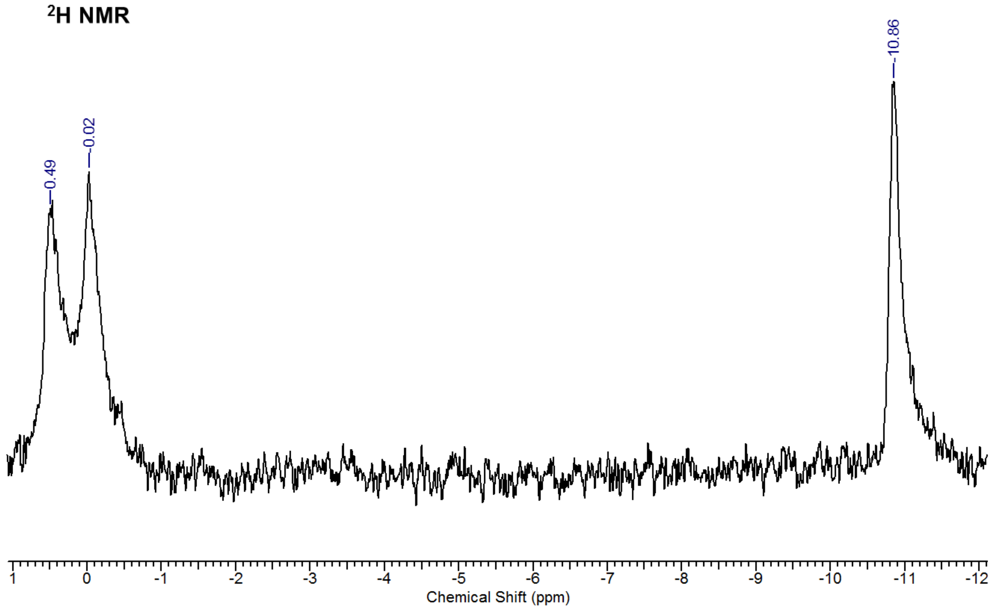

Figure S14: $^1H\{^{11}B\}$ (top) and ^2H NMR (bottom) spectra (upfield region) of the reaction mixture **1** with $D_3B{\cdot}PHPh_2$ to form $\mathbf{6^D}$ showing H and D in each of the 3 B-H environments.

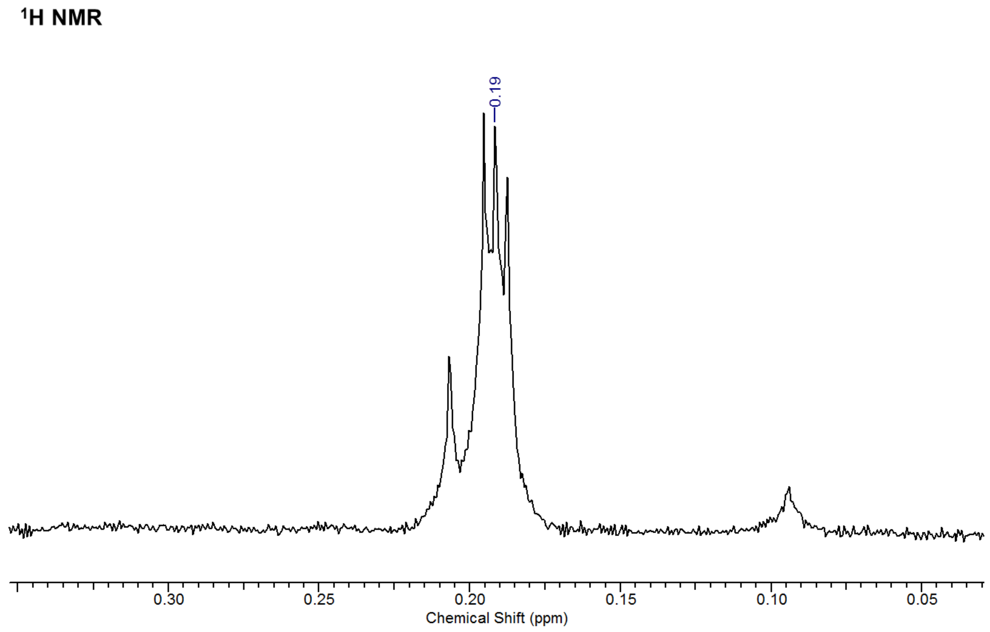

Figure S15: ^{1}H NMR spectrum showing CDH_3 product from reaction of **1** with $D_3B{\cdot}PHPh_2$. δ = 0.19 ($^{2}J_{HD}$ = 2.0 Hz).[8] This peak was not observed after degassing of the sample.

Reaction of 1 with $H_3B{\cdot}P^tBu_2H$ Followed by $^{31}P\{^{1}H\}$ NMR spectroscopy

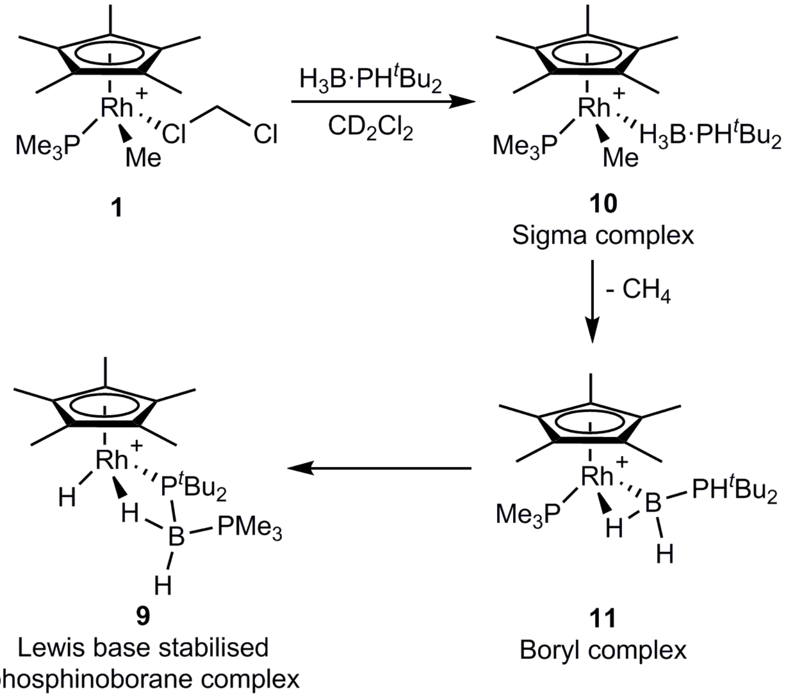

Scheme S5: Reaction of **1** with $H_3B{\cdot}P^tBu_2H$ followed by NMR spectroscopy. $[BAr^F{}_4]^-$ anions not shown.

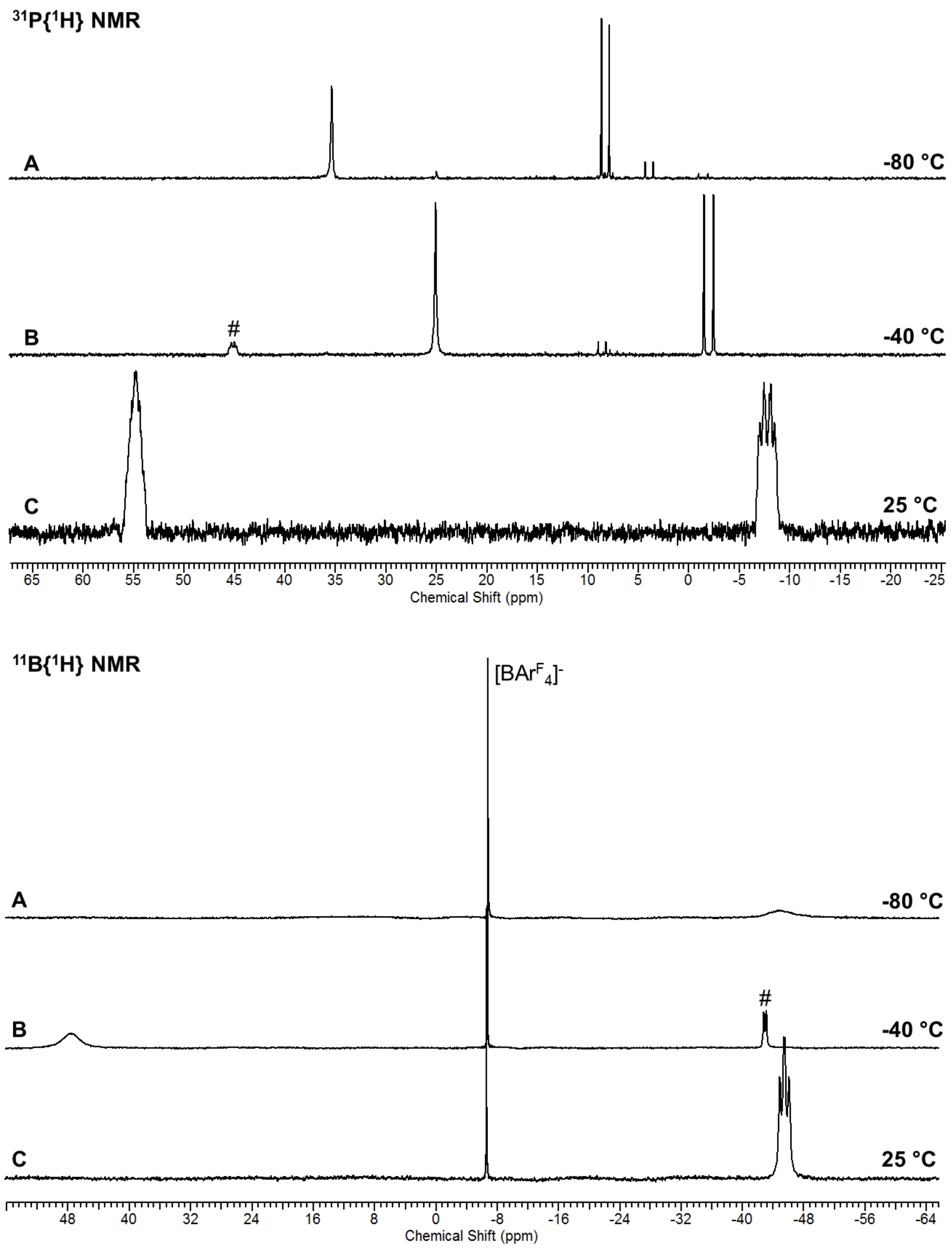

Figure S16: ^{31}P{^{1}H} NMR (top) and ^{11}B{^{1}H} NMR (bottom) spectra of reaction products from **1** and $H_3B\cdot PH^tBu_2$. **A**) Sigma complex [RhCp*Me(PMe$_3$)($H_3B\cdot P^tBu_2H$)][BAr$^F_4$] (**10**) at -80 °C; **B**) Boryl complex [RhCp*(PMe$_3$)($H_2B\cdot P^tBu_2H$)][BAr$^F_4$] (**11**) at -40 °C; **C**) Lewis base stabilised phosphinoborane complex [RhCp*H(P^{t}Bu$_2$BH$_2\cdot$PMe$_3$)][BAr$^F_4$] (**9**) at 25 °C (isolated material). Signals from free $H_3B\cdot P^tBu_2H$ labelled by #.

^{1}H NMR Spectra of $[RhCp^*(H_2B{\cdot}PCy_3)(PMe_3)][BAr^F{}_4]$ (12) under H_2 (4 atm) to form $[RhCp^*H(H_3B{\cdot}PCy_3)(PMe_3)][BAr^F{}_4]$ (13) at 25 and -60 °C

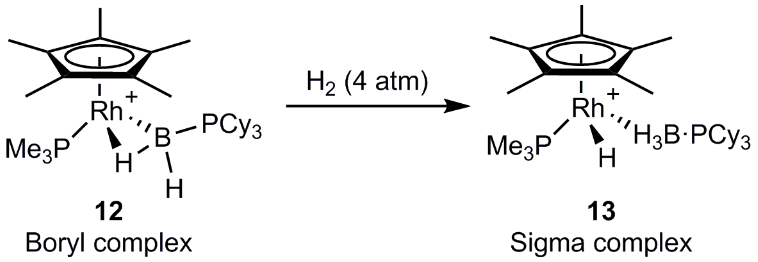

Scheme S6: Reaction of **12** with H_2 to form **13**. $[BAr^F{}_4]^-$ anions not shown.

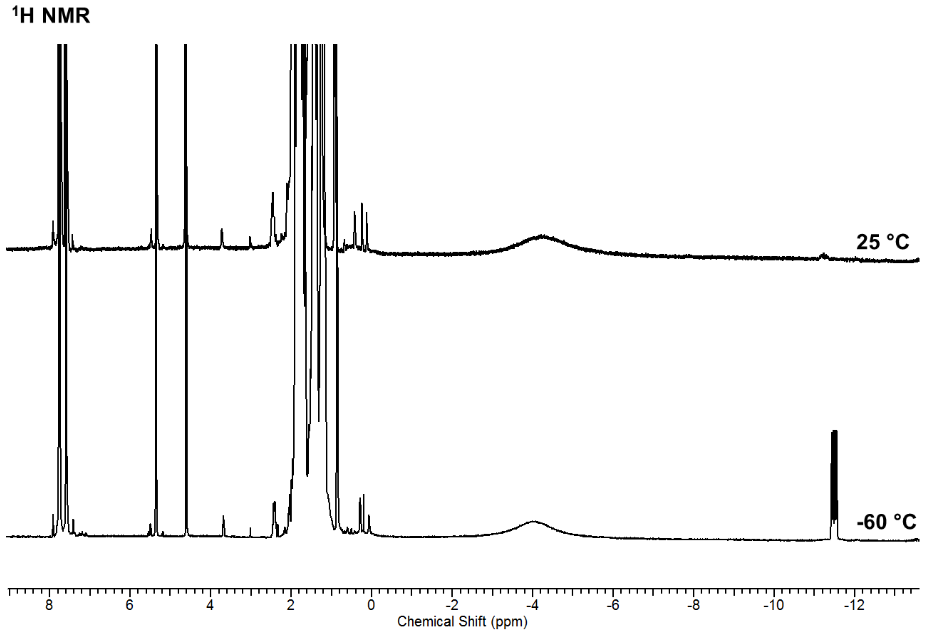

Figure S17: ^{1}H NMR spectra of **12** under H_2 (4 atm) to form **13** at 25 and -60 °C showing dynamic exchange process of Rh-H and B-H environments at 25 °C.

Addition of PPh_3 to $[RhCp^*(H_2B{\cdot}PCy_3)(PMe_3)][BAr^F{}_4]$ (12) to form $[RhCp^*(H_2B{\cdot}PCy_3)(PMe_3)(PPh_3)][BAr^F{}_4]$ (14)

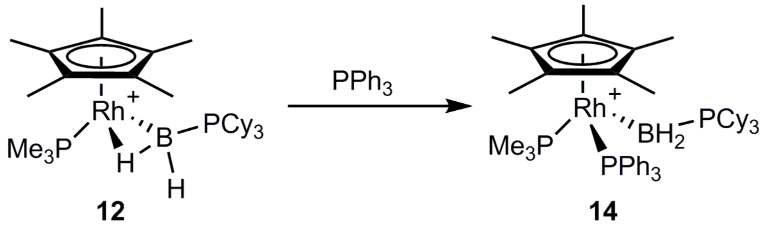

Scheme S7: Reaction of **12** with PPh_3 to form **14**. $[BAr^F{}_4]^-$ anions not shown.

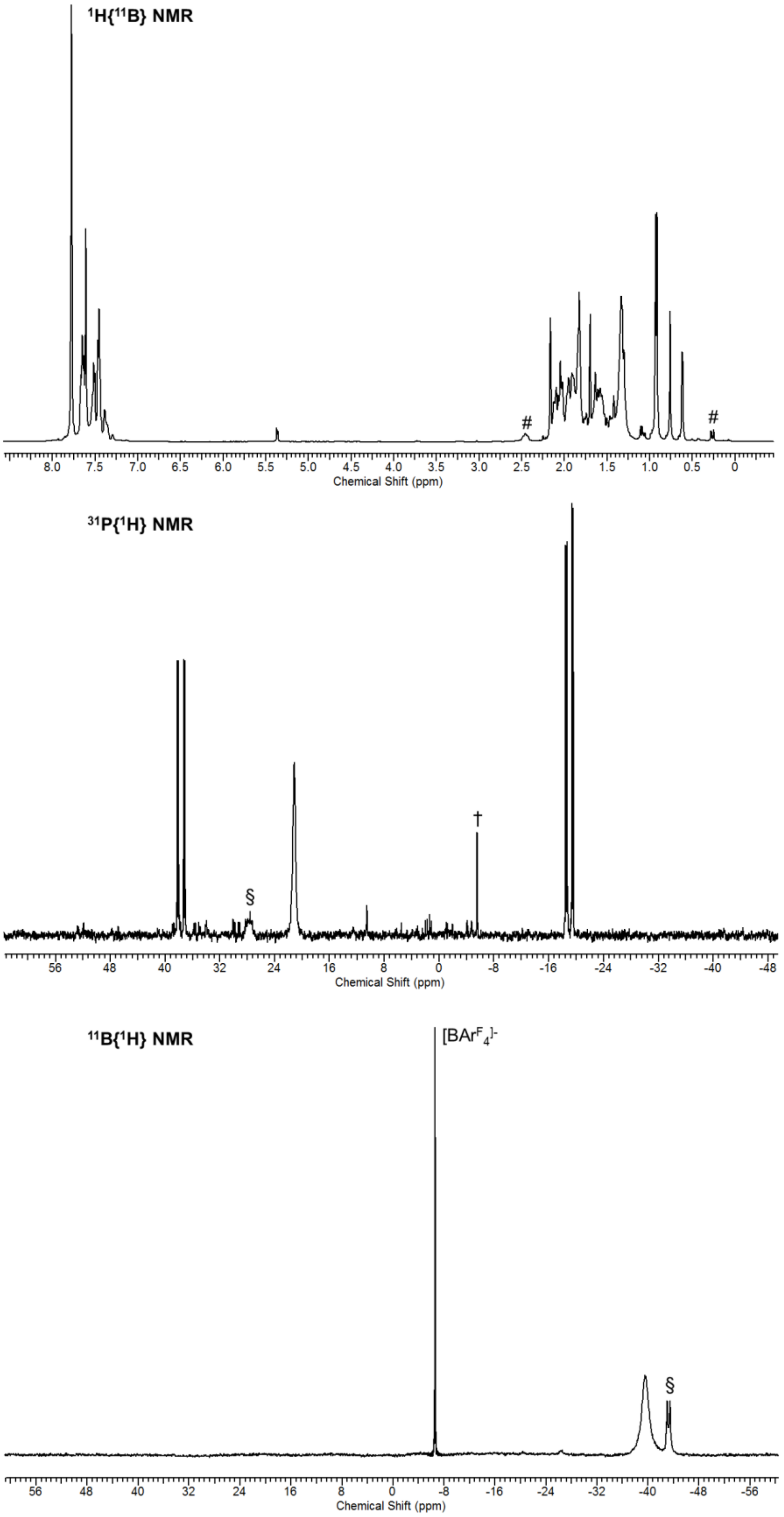

Figure S18: NMR spectra of isolated from reaction of 12 with PPh_3. Top, $^{1}H\{^{11}B\}$ NMR spectrum, B-H signals labelled with #; middle, $^{31}P\{^{1}H\}$ NMR spectrum; bottom, $^{11}B\{^{1}H\}$ NMR spectrum. Signals from free $H_3B{\cdot}PCy_3$ labelled by § and PPh_3 labelled by †.

Phosphine Exchange through Vacant Site Formation in $[RhCp^*(H)(PHPh_2)(PMe_3)][BAr^F_4]$ (5)

The formation of vacant sites (an hence an active catalytic species) in **5** was probed using a phosphine exchange experiment. **5** was mixed with PPh_3 (10 eq.) in toluene at room temperature for 2 hours (scheme S8). An ESI-MS experiment (figure S19) then showed several organometallic species resulting from phosphine exchange.

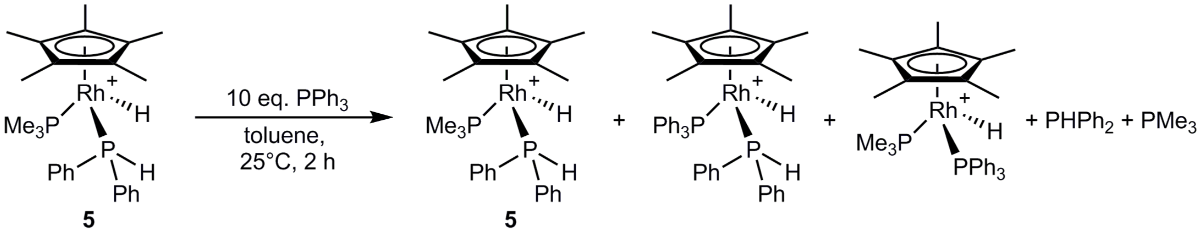

Scheme S8: Reaction of **5** with PPh_3 to form products resulting from ligand scrambling. $[BAr^F_4]^-$ anions not shown.

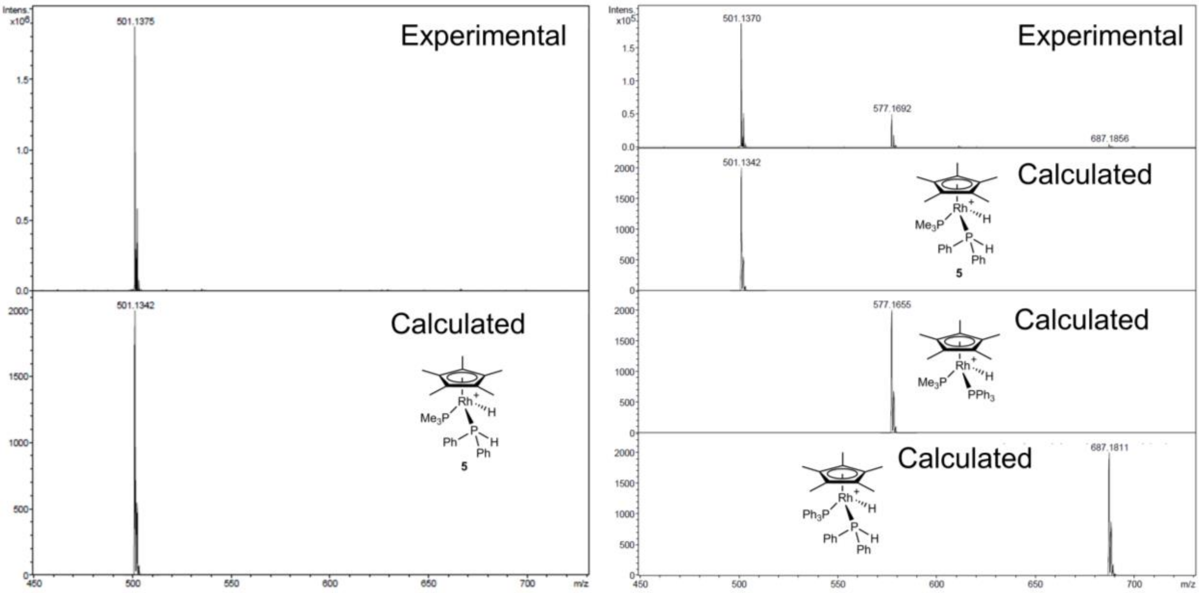

Figure S19: ESI-MS spectra (toluene) showing **5** before the reaction (left) and product mixture (right) after 2 h mixing in toluene.

Experimental References

1. A. B. Pangborn, M. A. Giardello, R. H. Grubbs, R. K. Rosen, F. J. Timmers, *Organometallics*, 1996, **15**, 1518.
2. H. Schmidbaur, E. Weiss, G. Muller, Synth. *React. Inorg. Met.–Org. Chem.,* **1985**, 401.
3. F. L. Taw, H. Mellows, P. S. White, F. J. Hollander, R. G. Bergman, M. Brookhart and D. M. Heinekey, *J. Am. Chem. Soc.*, 2002, **124**, 5100.
4. J. Cosier and A. M. Glazer, *J. App. Cryst.*, 1986, **19**, 105.
5. Z. Otwinowski and W. Minor, in *Macromolecular Crystallography, Pt A*, 1997, vol. 276, pp. 307.
6. G. M. Sheldrick, *Acta Cryst.*, 2008, **A64**, 112.
7. D.F. Gaines, *Inorg. Chem.*, 1963, **2**, 523.
8. H.C. Lo, M.A. Iron, J.M.L. Martin and E. Keinan, *Chem. Eur. J.*, 2007, **13**, 2812.

Computational Details

Calculations were run with Gaussian 03 Revision D.01[1] with PCM solvent corrections run with Gaussian 09, Revision D.01.[2] Geometry optimisations were performed using the BP86 functional[3] with Rh and P centres described with the Stuttgart RECPs and associated basis sets[4] (with added d-orbital polarisation on P (ζ = 0.387)[5] and 6-31G** basis sets for all other atoms.[6] All stationary points were fully characterized via analytical frequency calculations as either minima (all positive eigenvalues) or transition states (one negative eigenvalue) and IRC calculations and subsequent geometry optimizations were used to confirm the minima linked by each transition state. Frequency calculations also provided a free energy in the gas-phase, computed at 298.15 K and 1 atm. Energies reported in the text are based on the gas-phase free energies and incorporate a correction for dispersion effects using Grimme's D3 parameter set[7] (i.e. BP86-D3) as well as solvation (PCM approach) in CH_2Cl_2.

All geometries are provided as sets of Cartesian coordinates as well as the separate file All_PB_geometries.xyz readable by Chemcraft and Mercury.

Computational References

1. Gaussian 03, Revision D.01, M. J. Frisch, G. W. Trucks, H. B. Schlegel, G. E. Scuseria, M. A. Robb, J. R. Cheeseman, J. A. Montgomery, Jr., T. Vreven, K. N. Kudin, J. C. Burant, J. M. Millam, S. S. Iyengar, J. Tomasi, V. Barone, B. Mennucci, M. Cossi, G. Scalmani, N. Rega, G. A. Petersson, H. Nakatsuji, M. Hada, M. Ehara, K. Toyota, R. Fukuda, J. Hasegawa, M. Ishida, T. Nakajima, Y. Honda, O. Kitao, H. Nakai, M. Klene, X. Li, J. E. Knox, H. P. Hratchian, J. B. Cross, C. Adamo, J. Jaramillo, R. Gomperts, R. E. Stratmann, O. Yazyev, A. J. Austin, R. Cammi, C. Pomelli, J. W. Ochterski, P. Y. Ayala, K. Morokuma, G. A. Voth, P. Salvador, J. J. Dannenberg, V. G. Zakrzewski, S. Dapprich, A. D. Daniels, M. C. Strain, O. Farkas, D. K. Malick, A. D. Rabuck, K. Raghavachari, J. B. Foresman, J. V. Ortiz, Q. Cui, A. G. Baboul, S. Clifford, J. Cioslowski, B. B. Stefanov, G. Liu, A. Liashenko, P. Piskorz, I. Komaromi, R. L. Martin, D. J. Fox, T. Keith, M. A. Al-Laham, C. Y. Peng, A. Nanayakkara, M. Challacombe, P. M. W. Gill, B. Johnson, W. Chen, M. W. Wong, C. Gonzalez, and J. A. Pople, Gaussian, Inc., Wallingford CT, 2004.
2. Gaussian 09, Revision D.01, M. J. Frisch, G. W. Trucks, H. B. Schlegel, G. E. Scuseria, M. A. Robb, J. R. Cheeseman, G. Scalmani, V. Barone, B. Mennucci, G. A. Petersson, H. Nakatsuji, M. Caricato, X. Li, H. P. Hratchian, A. F. Izmaylov, J. Bloino, G. Zheng, J. L. Sonnenberg, M. Hada, M. Ehara, K. Toyota, R. Fukuda, J. Hasegawa, M. Ishida, T. Nakajima, Y. Honda, O. Kitao, H. Nakai, T. Vreven, J. A. Montgomery, Jr., J. E. Peralta, F. Ogliaro, M. Bearpark, J. J. Heyd, E. Brothers, K. N. Kudin, V. N. Staroverov, R. Kobayashi, J. Normand, K. Raghavachari, A. Rendell, J. C. Burant, S. S. Iyengar, J. Tomasi, M. Cossi, N. Rega, J. M. Millam, M. Klene, J. E. Knox, J. B. Cross, V. Bakken, C. Adamo, J. Jaramillo, R. Gomperts, R. E. Stratmann, O. Yazyev, A. J. Austin, R. Cammi, C. Pomelli, J. W. Ochterski, R. L. Martin, K. Morokuma, V. G. Zakrzewski, G. A. Voth, P. Salvador, J. J. Dannenberg, S. Dapprich, A. D. Daniels, Ö. Farkas, J. B. Foresman, J. V. Ortiz, J. Cioslowski, and D. J. Fox, Gaussian, Inc., Wallingford CT, 2009.
3. (a) A. D. Becke, *Phys. Rev. A* **1988**, *38*, 3098. (b) J. P. Perdew, *Physical Review B* **1986**, *33*, 8822.
4. D. Andrae, U. Häußermann, M. Dolg, H. Stoll and H. Preuß, *Theor. Chim. Acta* **1990**, *77*, 123.
5. A. Hollwarth, M. Bohme, S. Dapprich, A. W. Ehlers, A. Gobbi, V. Jonas, K. F. Kohler, R. Stegmann, A. Veldkamp and G. Frenking, *Chem. Phys. Lett.* **1993**, *208*, 237.
6. (a) W. J. Hehre, R. Ditchfield and J. A. Pople, *J. Chem. Phys.* **1972**, *56*, 2257. (b) P. C. Hariharan and J. A. Pople, *Theor. Chim. Acta.* **1973**, *28*, 213.
7. S. Grimme, J. Antony, S. Ehrlich and H. Krieg, *J. Chem. Phys.* **2010**, *132*, 154104.

Breakdown of Energy Contributions

The following tables detail the evolution of the relative energies as the successive corrections to the initial SCF energy are included. Terms used are:

**ΔE**	SCF energy computed with the BP86 functional
**ΔH**	Enthalpy at 0 K
**ΔG**	Free energy at 298.15 K and 1 atm
**ΔG_{DCM}**	Free energy corrected for dichloromethane solvent
**ΔG_{D3}**	Free energy corrected for dispersion effects
**ΔG_{final}**	Free energy corrected for dichloroethane solvent and dispersion effects

ΔG_{final} is the final data used in the main article. Compound labels are given in Scheme S9.

Scheme S9. Stationary points for the reactions of **10_R** to give **6_R**, **9_R** and **11_R**, where R = Me, Cy, Ph and ^{t}Bu. The alternative reaction of **Int(11"-9')$_R$** to **6_R** via **Int(11"-6)$_R$** is also indicated.[a, b]

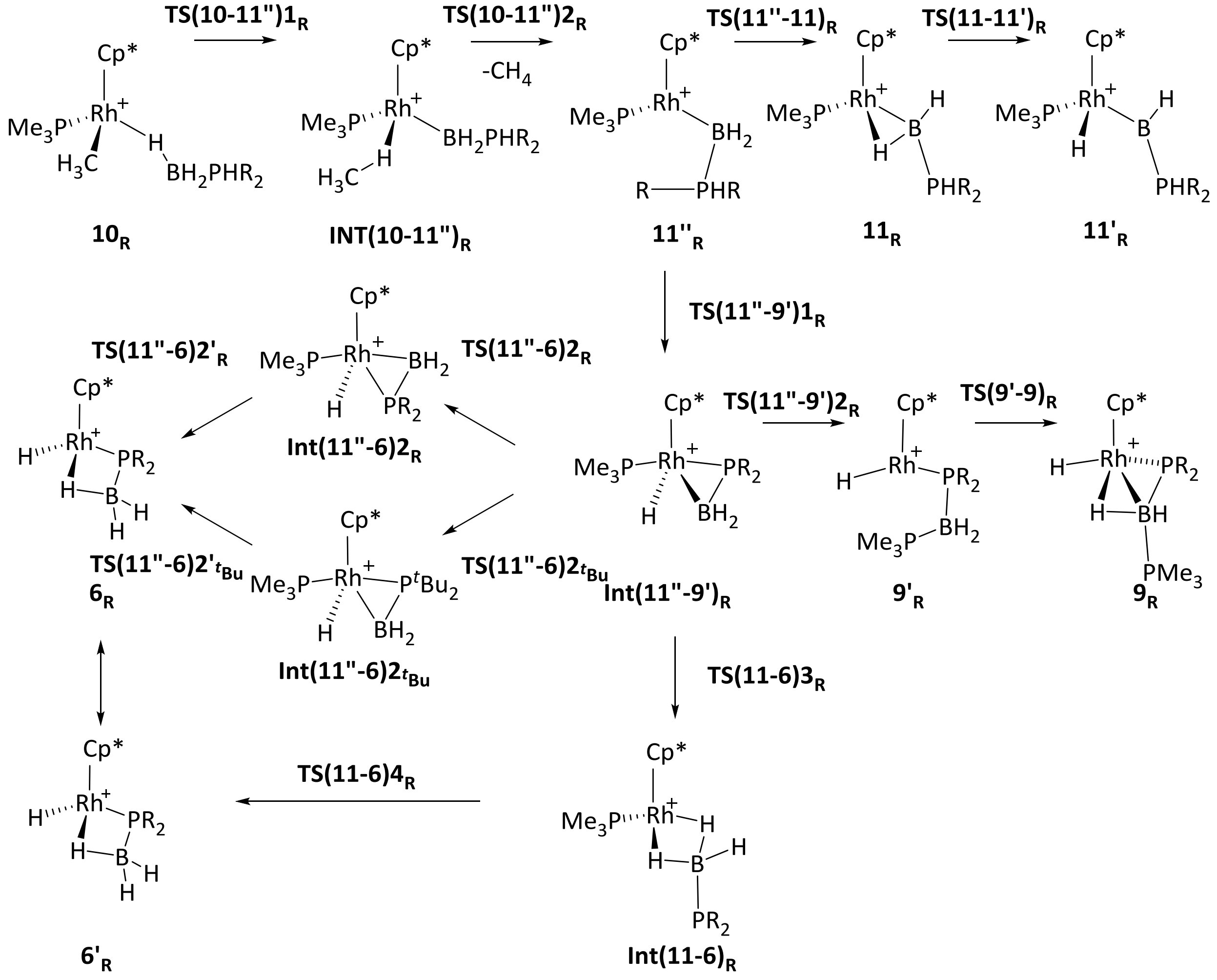

a. For R = Cy product **6_{Cy}** is equivalent to species **7** in the main paper.
b. **$6'_R$** is a higher energy conformer of **6_R**

Table S3. Computed relative energies (kcal/mol) for the reactions of **10_{tBu}**. All energies are quoted relative to **10_{tBu}** set to 0.0 kcal/mol.

R = ^{t}Bu	ΔE	ΔH	ΔG	ΔG_{DCM}	ΔG_{D3}	ΔG_{final}
**10_R**	0.00	0.00	0.00	0.00	0.00	0.00
**TS(10-11")1_R**	17.98	16.73	18.04	17.69	14.48	14.12
**INT(10-11")$_R$**	10.48	10.66	10.59	10.09	7.43	6.92
**TS(10-11")2_R**	10.68	10.00	9.13	8.56	6.42	5.86
**11"$_R$**	6.58	5.16	-4.90	-6.04	-0.45	-1.59
**TS(11"-11)$_R$**	11.20	9.28	-1.04	-2.35	6.84	5.52
**11_R**	-2.15	-3.73	-14.22	-14.72	-6.51	-7.01
**TS(11-11')$_R$**	1.56	-1.01	-10.87	-11.66	-3.20	-3.99
**11'$_R$**	0.75	-0.86	-11.25	-12.14	-3.98	-4.88
**TS(11"-9')1_R**	25.94	25.06	16.91	15.02	19.10	17.20
**INT(11"-9')$_R$**	5.77	5.54	-2.94	-4.10	-2.86	-4.02
**TS(11"-6)2_R**	9.07	8.55	0.95	-0.06	1.70	0.69
**INT(11"-6)2_R**	8.50	8.34	-0.33	-1.36	-0.26	-1.29
**TS(11"-6)2'$_R$**	10.10	9.72	2.32	1.24	2.91	1.83
**6_R**	4.65	5.07	-2.94	-4.88	-2.56	-4.50
**6'$_R$**	9.12	9,45	1.67	-0.22	2.74	0.85
**TS(11"-6)3_R**	31.90	28.45	19.46	18.74	25.34	24.63
**INT(11"-6)$_R$**	5.34	3.33	-6.72	-9.77	1.97	-1.09
**TS(11"-6)4_R**	18.38	16.18	6.47	4.52	13.86	11.91
**TS(11"-9')2_R**	13.13	12.79	4.91	5.76	6.81	7.67
**9'$_R$**	1.30	0.07	-9.31	-11.34	-5.50	-7.53
**TS(9'-9)$_R$**	3.80	1.92	-8.22	-10.31	-2.62	-4.72
**9_R**	-11.00	-12.27	-22.17	-23.57	-15.47	-16.88

Table S4. Computed relative energies (kcal/mol) for the reactions of $\mathbf{10_{Ph}}$. All energies are quoted relative to $\mathbf{10_{Ph}}$ set to 0.0 kcal/mol.

R = Ph	ΔE	ΔH	ΔG	ΔG_{DCM}	ΔG_{D3}	ΔG_{final}
**10_R**	0.00	0.00	0.00	0.00	0.00	0.00
**TS(10-11")1$_R$**	16.44	14.65	15.24	14.77	12.46	11.99
**INT(10-11")$_R$**	6.31	6.28	6.61	5.97	2.95	2.31
**TS(10-11")2$_R$**	6.41	6.14	7.01	6.36	4.11	3.45
**11"$_R$**	6.49	4.65	-4.68	-5.74	-1.36	-2.42
**TS(11"-11)$_R$**	9.87	7.75	-2.16	-3.76	5.70	4.10
**11_R**	-1.64	-3.08	-12.62	-12.92	-5.42	-5.72
**TS(11-11')$_R$**	2.58	-0.27	-9.93	-12.30	-2.11	-4.47
**11'$_R$**	1.79	-0.01	-10.50	-10.82	-3.11	-3.42
**TS(11"-9')1$_R$**	5.66	4.43	-4.48	-6.43	0.33	-1.63
**INT(11"-9')$_R$**	-16.67	-17.49	-24.91	-25.55	-27.35	-28.00
**TS(11"-6)2$_R$**	-3.31	-4.48	-11.44	-12.10	-12.02	-12.68
**INT(11"-6)2$_R$**	-5.31	-6.16	-13.74	-14.94	-16.17	-17.38
**TS(11"-6)2$_R$**	-5.27	-6.16	-12.42	-13.64	-14.67	-15.89
**6_R**	-23.29	-23.96	-31.67	-33.29	-33.67	-35.29
**TS(11"-6)3$_R$**	23.76	19.96	10.57	9.36	14.97	13.76
**INT(11"-6)$_R$**	-3.36	-5.40	-15.44	-17.99	-9.38	-11.94
**TS(11"-6)4$_R$**	12.10	9.60	0.37	-1.73	7.39	5.28
**TS(11"-9')2$_R$**	1.14	0.44	-6.29	-6.82	-4.97	-5.50
**9'$_R$**	-9.77	-11.81	-22.41	-24.69	-18.12	-20.40
**TS(9'-9)$_R$**	-8.24	-10.07	-18.72	-20.41	-12.65	-14.34
**9_R**	-22.95	-24.26	-33.38	-33.96	-28.26	-28.84

Table S5. Computed relative energies (kcal/mol) for the reactions of $\mathbf{10_{Cy}}$. All energies are quoted relative to $\mathbf{10_{Cy}}$ set to 0.0 kcal/mol.

R = Cy	ΔE	ΔH	ΔG	ΔG_{DCM}	ΔG_{D3}	ΔG_{final}
$\mathbf{10_R}$	0.00	0.00	0.00	0.00	0.00	0.00
**TS(10-11")1**$_R$	17.50	15.94	16.75	15.17	13.84	12.27
**INT(10-11")**$_R$	9.45	9.93	10.90	10.29	7.97	7.36
**TS(10-11")2**$_R$	10.19	9.31	8.80	7.85	7.93	6.97
**11"**$_R$	6.20	4.05	-6.28	-7.37	-2.58	-3.66
**TS(11"-11)**$_R$	11.11	8.93	-1.33	-2.52	7.76	6.57
$\mathbf{11_R}$	-1.78	-3.48	-14.34	-14.76	-5.22	-5.64
**TS(11-11')**$_R$	2.16	-0.68	-11.28	-12.12	-2.38	-3.21
**11'**$_R$	1.54	-0.22	-10.57	-11.27	-1.94	-2.64
**TS(11"-9')1**$_R$	8.68	6.88	-2.64	-4.74	1.84	-0.26
**INT(11"-9')**$_R$	-5.29	-5.41	-13.23	-14.70	-13.33	-14.80
**TS(11"-6)2**$_R$	4.13	2.84	-4.69	-5.64	-3.64	-4.59
**INT(11"-6)2**$_R$	2.37	1.76	-6.18	-7.39	-5.42	-6.63
$\mathbf{6_R}$ **(=7)**	-14.48	-14.77	-23.07	-25.39	-23.09	-25.41
**TS(11"-6)3**$_R$	26.82	22.47	11.85	10.48	18.73	17.36
**INT(11"-6)**$_R$	1.49	-0.57	-10.99	-13.23	-2.22	-4.47
**TS(11"-6)4**$_R$	17.15	13.91	2.72	0.63	11.92	9.83
**TS(11"-9')2**$_R$	8.71	7.26	-1.39	-2.81	1.33	-0.09
**9'**$_R$	-2.41	-4.40	-14.96	-17.60	-9.70	-12.34
**TS(9'-9)**$_R$	-1.75	-3.51	-12.93	-14.94	-5.16	-7.17
$\mathbf{9_R}$	-14.59	-16.19	-26.04	-27.39	-19.56	-20.92

Table S6. Functional Testing on the Relative Free Energies (kcal/mol) of **11**$_{t\text{Bu}}$, **TS(11-11')**$_{t\text{Bu}}$ and **11'**$_{t\text{Bu}}$.

**Functional**	**11**$_{t\text{Bu}}$	**TS(11-11')**$_{t\text{Bu}}$	**11'**$_{t\text{Bu}}$
BP86	0.00	3.35	2.97
BP86-D3(DCM)	0.00	3.02	2.13
PBE0	0.00	4.18	-1.11
PBE0-D3(DCM)	0.00	3.67	2.08
B3LYP	0.00	3.45	3.02
B3LYP-D3(DCM)	0.00	2.91	1.79
M06	0.00	10.70	6.64
M06(DCM)	0.00	5.91	5.72
B97D	0.00	3.79	0.77
B97D(DCM)	0.00	3.08	0.23

Table S7. Computed ^{11}B NMR Chemical Shifts (ppm) for $\mathbf{10_R}$, $\mathbf{11''_R}$, $\mathbf{11'_R}$, $\mathbf{11_R}$, $\mathbf{9_R}$ and $\mathbf{6_R}$.

Species	R = Me	R = ^{t}Bu	R = Ph	R = Cy
$\mathbf{10_R}$	-41.07	-44.76	-46.13	-42.84
$\mathbf{11''_R}$	-9.45	-14.29	-10.32	-11.78
$\mathbf{11'_R}$	115.51	119.33	112.51	114.59
$\mathbf{11_R}$	49.33	53.73	51.97	53.99
$\mathbf{9_R}$	-48.81	-49.14	-49.88	-49.23
$\mathbf{6_R}$	-51.63	-37.13	-52.28	-49.01 (=**7**)

Table S8. Functional Testing on the Computed ^{11}B NMR Chemical Shifts (ppm) of $\mathbf{11}_{t\mathbf{Bu}}$ and $\mathbf{11'}_{t\mathbf{Bu}}$ and $\mathbf{11''}_{t\mathbf{Bu}}$.[a]

Methodology	11-1	11-2	11-3
BP86/BS1//BP86/BS1	7.29	115.85	66.25
BP86/BS2//BP86/BS1	15.66	134.73	79.05
B3LYP/BS2//BP86/BS1	-14.29	119.33	53.73
B97D/BS2//BP86/BS1	16.46	129.04	76.64
M06/BS2//BP86/BS1	17.92	148.80	86.77
PBE0/BS2//BP86/BS1	10.30	144.75	79.30

a. BS1 = SDD on Rh and P, with d-orbital polarisation on P; 6-31g** on other atoms;
BS2 = SDD on Rh and P, with d-orbital polarisation on P; 6-311++g** on other atoms.

Cartesian Coordinates (Å) and computed energies (in Hartrees) for all optimised structures. All structures can be visualized via the All_PB_geometries.xyz file provided.

10_{tBu}
SCF Energy = -1016.40413259
Enthalpy 0K = -1015.759217
Enthalpy 298K = -1015.717744
Free Energy 298K = -1015.828783
SCF(DCM) = -1016.44920397
SCF(BP86-D3) = -0.12421940
Lowest Frequencies = 18.2674 26.0989 cm-1

```
C  -1.90044 -0.93186  1.90629
C  -1.51515 -2.03622  1.01683
C  -2.41551 -2.06927 -0.09644
C  -3.31224 -0.92659  0.03110
C  -3.01877 -0.27538  1.31715
Rh -1.30407 -0.09461 -0.21074
B   1.13732  0.31971  0.58719
P   2.96585  0.37278 -0.18637
C   3.40966 -1.05683 -1.37880
C   4.91012 -1.10720 -1.73101
C  -0.46973 -3.06035  1.34955
C  -2.50238 -3.12935 -1.15577
C  -4.53077 -0.69567 -0.81741
C  -3.87637  0.78201  1.95167
C  -1.29960 -0.66974  3.25757
P  -1.47574  2.18005 -0.64321
C  -3.00049  2.71991 -1.56642
C  -1.48325  3.29172  0.85237
C  -0.12211  2.94985 -1.66616
C   4.25187  0.73443  1.19084
C   3.62008  1.78963  2.13039
C   4.54020 -0.55898  1.98206
C   5.54648  1.31886  0.58441
C   2.95522 -2.39081 -0.74676
C   2.59704 -0.77972 -2.66561
H  -1.55696 -3.68386 -1.26139
H  -2.76044 -2.70897 -2.14103
H  -3.28794 -3.86220 -0.89518
H  -5.34737 -1.37584 -0.51075
H  -4.32774 -0.88957 -1.88304
H  -4.91066  0.33324 -0.72555
H  -4.28076  1.49125  1.21173
H  -3.33461  1.35182  2.72214
H  -4.74497  0.30770  2.44555
H  -1.63996 -1.42601  3.98917
H  -1.58591  0.31953  3.64658
H  -0.19879 -0.71264  3.22499
H  -0.87173 -3.77913  2.08796
H   0.42380 -2.59682  1.79620
H  -0.15839 -3.63699  0.46501
H   1.19829 -0.52211  1.45548
H   3.14350  1.51462 -1.03703
H   2.91995  0.15108 -3.16259
H   1.51375 -0.72109 -2.47473
H   2.76416 -1.60914 -3.37611
H   5.06343 -1.88349 -2.50239
H   5.53939 -1.37951 -0.86882
H   5.27409 -0.15370 -2.14854
H   1.87962 -2.38235 -0.50674
H   3.51255 -2.63194  0.17297
H   3.13582 -3.20843 -1.46734
H   6.23046  1.59091  1.40846
H   5.35011  2.23666  0.00430
H   6.07870  0.60694 -0.06217
H   2.71658  1.41339  2.63376
H   3.36001  2.72076  1.59791
H   4.36209  2.04961  2.90649
H   3.61787 -1.00915  2.38542
H   5.19360 -0.31690  2.83909
H   5.06516 -1.31464  1.37527
H  -0.28717  4.03651 -1.74666
H  -0.11275  2.51120 -2.67476
H   0.84660  2.76240 -1.18026
H  -2.96776  3.80337 -1.76595
H  -3.89796  2.49322 -0.97091
H  -3.06298  2.17369 -2.52004
H  -0.55926  3.12312  1.42643
H  -2.34647  3.05720  1.49158
H  -1.53959  4.34885  0.54391
C  -1.16932 -0.31251 -2.31502
H  -2.17223 -0.18159 -2.75578
H  -0.47927  0.39300 -2.80435
H  -0.82116 -1.33742 -2.52542
H   0.45647 -0.06068 -0.45204
H   0.96427  1.44491  0.99519
```

TS(10-11'')1_{tBu}
SCF Energy = -1016.37548654
Enthalpy 0K = -1015.732552
Enthalpy 298K = -1015.691617
Free Energy 298K = -1015.800038
SCF(DCM) = -1016.42112067
SCF(BP86-D3) = -0.12989451
Lowest Frequencies = -918.4650 21.5576 cm-1

```
C  -2.27615 -0.50481  1.71895
C  -1.58852 -1.72462  1.36150
C  -2.08998 -2.15464  0.08119
C  -3.17387 -1.25979 -0.31554
C  -3.29818 -0.25652  0.69519
Rh -1.17762 -0.06532 -0.21494
B   0.91784  0.25117  0.63719
P   2.68931  0.11224 -0.27596
C   3.29479 -1.67172 -0.65275
C   4.39672 -1.68132 -1.73464
C  -0.64186 -2.48417  2.24363
C  -1.73549 -3.42830 -0.63279
C  -4.04096 -1.44656 -1.52887
C  -4.40696  0.75057  0.81856
C  -2.16777  0.20069  3.04279
P  -1.22047  2.22306 -0.56495
C  -2.66183  2.81523 -1.59349
C  -1.31508  3.31548  0.93829
C   0.19573  2.96582 -1.51734
```

C 3.98345 1.18934 0.66250
C 3.59035 2.66216 0.39986
C 3.89802 0.89390 2.17632
C 5.41697 0.95911 0.14327
C 3.78312 -2.35040 0.64533
C 2.06646 -2.43801 -1.19339
H -0.74346 -3.80464 -0.34005
H -1.74778 -3.30598 -1.72874
H -2.47128 -4.21788 -0.39172
H -4.75989 -2.27214 -1.37499
H -3.44533 -1.70194 -2.42249
H -4.62418 -0.54195 -1.76171
H -4.89736 0.94997 -0.14673
H -4.06490 1.70898 1.24101
H -5.18376 0.36183 1.50305
H -2.75708 -0.32656 3.81630
H -2.54828 1.23285 2.99127
H -1.12414 0.24413 3.39229
H -1.21018 -3.18978 2.87822
H -0.07895 -1.81509 2.91182
H 0.08461 -3.07565 1.66414
H 1.03471 -0.61642 1.47690
H 2.77322 0.70537 -1.58349
H 1.70538 -2.01868 -2.14778
H 1.23307 -2.43789 -0.47292
H 2.35763 -3.48640 -1.38686
H 4.64418 -2.73020 -1.97964
H 5.32569 -1.19251 -1.40900
H 4.05903 -1.19713 -2.66689
H 3.02046 -2.31740 1.44139
H 4.71144 -1.90162 1.03271
H 3.99876 -3.41296 0.43318
H 6.09249 1.67387 0.64736
H 5.50303 1.13509 -0.94227
H 5.79080 -0.05177 0.36864
H 2.58211 2.89344 0.77943
H 3.64290 2.92252 -0.67166
H 4.30243 3.31788 0.93235
H 2.88220 1.06325 2.56766
H 4.58669 1.57283 2.71030
H 4.19647 -0.13761 2.42051
H 0.03923 4.04993 -1.63955
H 0.25506 2.49995 -2.51302
H 1.13676 2.79284 -0.97828
H -2.61038 3.90768 -1.73267
H -3.61058 2.55876 -1.10141
H -2.63139 2.32539 -2.57969
H -0.46414 3.09116 1.59862
H -2.24919 3.11453 1.48476
H -1.29242 4.37811 0.64595
C -0.61691 -0.35375 -2.36475
H -1.51438 0.10684 -2.80301
H 0.25835 0.10188 -2.85926
H -0.61270 -1.43307 -2.57076
H 0.15248 -0.22619 -1.10790
H 0.97138 1.37029 1.11160

INT(10-11’’)$_{tBu}$
SCF Energy = -1016.38743453
Enthalpy 0K = -1015.742225
Enthalpy 298K = -1015.700097
Free Energy 298K = -1015.811899
SCF(DCM) = -1016.43330718
SCF(BP86-D3) = -0.12926813
Lowest Frequencies = 19.6340
28.5919 cm-1
C -2.09511 -0.52098 1.70054
C -1.50176 -1.78479 1.27200
C -2.12302 -2.13306 0.03302
C -3.22910 -1.19827 -0.24000
C -3.23527 -0.23158 0.79162
Rh -1.14292 -0.03746 -0.16507
B 0.87641 0.35054 0.70096
P 2.60546 0.08331 -0.25757
C 3.16136 -1.73045 -0.56269
C 4.26210 -1.83347 -1.64130
C -0.56016 -2.62783 2.08036
C -1.85954 -3.36591 -0.78258
C -4.20620 -1.33502 -1.37362
C -4.29094 0.80405 1.05146
C -1.85956 0.12015 3.03720
P -1.17282 2.25030 -0.51792
C -2.67031 2.84392 -1.46682
C -1.19928 3.36632 0.97063
C 0.18898 2.98432 -1.55536
C 3.97679 1.17250 0.56141
C 3.63674 2.63949 0.20883
C 3.92238 0.98549 2.09400
C 5.38854 0.85510 0.02985
C 3.62354 -2.36501 0.76622
C 1.90865 -2.47295 -1.07902
H -0.86230 -3.78852 -0.58997
H -1.95229 -3.17348 -1.86417
H -2.60454 -4.14479 -0.53296
H -4.95056 -2.12511 -1.16140
H -3.70702 -1.61500 -2.31694
H -4.76088 -0.40035 -1.55138
H -4.86931 1.04603 0.14621
H -3.87504 1.73844 1.46081
H -5.00668 0.42092 1.80297
H -2.40971 -0.42669 3.82668
H -2.20245 1.16605 3.06168
H -0.78979 0.10473 3.29713
H -1.11773 -3.14449 2.88383
H 0.22716 -2.02256 2.55676
H -0.07415 -3.40434 1.46973
H 1.01508 -0.39499 1.65606
H 2.64641 0.59758 -1.60060
H 1.60109 -2.10564 -2.07264
H 1.05521 -2.36665 -0.39079
H 2.14719 -3.54715 -1.18571
H 4.44029 -2.90149 -1.86375
H 5.22123 -1.40170 -1.32440
H 3.95788 -1.34752 -2.58431
H 2.86031 -2.26705 1.55600
H 4.56422 -1.92637 1.13561
H 3.80628 -3.44293 0.60684
H 6.09829 1.58477 0.46074
H 5.45517 0.94173 -1.06763
H 5.73791 -0.14593 0.32772
H 2.65042 2.93993 0.59732
H 3.66928 2.82357 -0.87931

H   4.38996 3.29936 0.67583
H   2.92338 1.21764 2.49597
H   4.64908 1.67270 2.56335
H   4.18955 -0.03792 2.40171
H   0.02782 4.06808 -1.67548
H   0.18797 2.51082 -2.54912
H   1.15923 2.80948 -1.07198
H -2.63180 3.93791 -1.59906
H -3.58975 2.57914 -0.92575
H -2.69223 2.36329 -2.45707
H -0.32176 3.15468 1.59811
H -2.11151 3.17979 1.55776
H -1.18916 4.42247 0.65528
C -0.59729 -0.40456 -2.84389
H -1.65548 -0.11679 -2.87221
H -0.01657 0.24563 -3.51485
H -0.48750 -1.45671 -3.14084
H -0.10888 -0.30470 -1.82238
H   1.01730 1.50812 1.06537

TS(10-11’’)2_{tBu}
SCF Energy = -1016.38711640
Enthalpy 0K = -1015.743277
Enthalpy 298K = -1015.701092
Free Energy 298K = -1015.814235
SCF(DCM) = -1016.43309218
SCF(BP86-D3) = -0.12853091
Lowest Frequencies = -82.0360
15.5675 cm-1
C -2.13242 -0.53258 1.70190
C -1.51565 -1.79114 1.28583
C -2.09453 -2.13053 0.02532
C -3.19826 -1.19964 -0.27908
C -3.24676 -0.24280 0.75713
Rh -1.11859 -0.02005 -0.10621
B   0.87683 0.36246 0.76610
P   2.57956 0.08451 -0.23103
C   3.09643 -1.73528 -0.56280
C   4.17031 -1.84935 -1.66680
C -0.59172 -2.62825 2.12039
C -1.80032 -3.35290 -0.79461
C -4.13421 -1.33605 -1.44664
C -4.31369 0.78685 0.99174
C -1.94874 0.10320 3.04805
P -1.15097 2.26163 -0.50078
C -2.64229 2.84362 -1.46642
C -1.18700 3.39292 0.97566
C   0.22132 2.97215 -1.53999
C   3.98594 1.14598 0.56508
C   3.65991 2.62029 0.23058
C   3.96099 0.94872 2.09713
C   5.38096 0.81098 0.00094
C   3.57902 -2.38639 0.75076
C   1.81750 -2.44891 -1.05239
H -0.83635 -3.81102 -0.53011
H -1.79630 -3.13417 -1.87506
H -2.58859 -4.11065 -0.62579
H -4.88863 -2.12300 -1.25850
H -3.60092 -1.61815 -2.36949
H -4.67897 -0.40002 -1.64617
H -4.87146 1.02428 0.07261
H -3.91164 1.72409 1.40889
H -5.04486 0.40171 1.72725
H -2.53607 -0.44512 3.80977
H -2.28930 1.15007 3.06243
H -0.89193 0.08047 3.35472
H -1.16263 -3.12875 2.92449
H   0.19424 -2.02028 2.59645
H -0.10249 -3.41674 1.52806
H   1.02259 -0.39240 1.71492
H   2.59411 0.60887 -1.57009
H   1.49380 -2.07392 -2.03801
H   0.98386 -2.31993 -0.34419
H   2.02795 -3.52867 -1.16335
H   4.32386 -2.91846 -1.90178
H   5.14386 -1.43779 -1.36722
H   3.85448 -1.34989 -2.59878
H   2.83686 -2.28042 1.55942
H   4.53524 -1.96478 1.09934
H   3.74038 -3.46607 0.58076
H   6.11068 1.52679 0.42169
H   5.42606 0.90439 -1.09712
H   5.72119 -0.19723 0.28484
H   2.68486 2.93058 0.63958
H   3.67576 2.81285 -0.85647
H   4.43013 3.26613 0.68937
H   2.97280 1.18843 2.52068
H   4.70412 1.62510 2.55641
H   4.22373 -0.07938 2.39279
H   0.04674 4.04680 -1.71238
H   0.24904 2.45148 -2.50947
H   1.18332 2.83494 -1.02885
H -2.59344 3.93359 -1.62583
H -3.56460 2.60105 -0.91983
H -2.66840 2.33806 -2.44396
H -0.31111 3.18937 1.60817
H -2.10020 3.20522 1.56125
H -1.18070 4.44644 0.65160
C -0.55136 -0.35971 -3.09950
H -1.43186 0.27379 -3.26622
H   0.32817 0.06686 -3.60324
H -0.74472 -1.37623 -3.47151
H -0.31300 -0.44467 -2.00559
H   1.03127 1.51731 1.13604

$11''_{tBu}$
SCF Energy = -975.877462273
Enthalpy 0K = -975.278704
Enthalpy 298K = -975.240015
Free Energy 298K = -975.344616
SCF(DCM) = -975.924470500
SCF(BP86-D3) = -0.11694904
Lowest Frequencies = 25.1664
36.7629 cm-1
Rh -1.00276 -0.02416 -0.04636
C -2.69017 -1.58224 -0.65586
C -3.34115 -0.46153 -0.10041
C -2.73394 -0.22937 1.22697
C -1.85064 -1.36278 1.53772
C -1.75140 -2.14194 0.34970
C -2.95347 -2.19863 -1.99902
H -2.02821 -2.56394 -2.47356

H -3.43634 -1.48916 -2.68837
H -3.62564 -3.07126 -1.89479
C -4.50866 0.28320 -0.68424
H -5.45431 -0.22751 -0.42201
H -4.46186 0.33448 -1.78413
H -4.58305 1.31016 -0.29796
C -3.24461 0.73487 2.25750
H -3.63355 1.66203 1.80987
H -2.46762 0.99556 2.99199
H -4.08156 0.26909 2.81292
C -1.29429 -1.68269 2.89357
H -2.07096 -2.17907 3.50532
H -0.96917 -0.77700 3.42849
H -0.43060 -2.36060 2.83123
C -1.01115 -3.43989 0.19554
H -1.66999 -4.29043 0.45326
H -0.13531 -3.48732 0.86144
H -0.66854 -3.60077 -0.83913
B 0.85564 0.01541 1.09134
H 0.92979 0.85812 1.96400
H 1.02065 -1.11761 1.51470
P 2.46684 0.23490 -0.02238
H 2.61360 1.51557 -0.66006
C 2.44330 -0.90892 -1.55884
C 1.02836 -0.75119 -2.16031
H 0.86232 0.25261 -2.57756
H 0.22473 -0.98340 -1.40112
H 0.87483 -1.48272 -2.97527
C 3.46910 -0.48183 -2.63261
H 3.31933 -1.09212 -3.54167
H 4.50761 -0.63290 -2.30461
H 3.34939 0.57657 -2.92069
C 2.67935 -2.37047 -1.12364
H 1.97336 -2.68724 -0.33859
H 3.70422 -2.52351 -0.74921
H 2.54452 -3.03843 -1.99353
C 4.08535 0.19728 1.01923
C 5.35252 0.15162 0.14426
H 6.23686 0.27287 0.79604
H 5.38005 0.96481 -0.60067
H 5.46707 -0.81199 -0.37774
C 4.06019 1.51881 1.82558
H 3.15444 1.60558 2.44746
H 4.12944 2.40477 1.17069
H 4.93426 1.53879 2.50078
C 4.04672 -1.00434 1.98744
H 3.15522 -0.97954 2.63342
H 4.94079 -0.96739 2.63516
H 4.06304 -1.97050 1.45707
P -0.83523 2.27077 -0.43198
C -0.00821 2.81101 -2.02354
H -0.08014 3.90685 -2.12249
H -0.50995 2.33985 -2.88326
H 1.05440 2.52613 -2.02716
C -2.45802 3.17545 -0.61669
H -2.27076 4.23986 -0.83382
H -3.03929 3.10345 0.31472
H -3.04023 2.73417 -1.43912
C 0.01854 3.35333 0.82160
H 1.06841 3.04669 0.93120
H -0.47445 3.23351 1.79840
H -0.03133 4.40928 0.50956

TS(11''-11)$_{tBu}$
SCF Energy = -975.870094237
Enthalpy 0K = -975.272141
Enthalpy 298K = -975.233597
Free Energy 298K = -975.338465
SCF(DCM) = -975.917383375
SCF(BP86-D3) = -0.11148692
Lowest Frequencies = -196.0516 16.4516 cm-1
Rh -1.19274 -0.07129 -0.13097
C -3.07586 -1.18950 -0.93488
C -3.56693 -0.21439 -0.03816
C -2.84015 -0.40353 1.22480
C -2.09129 -1.67662 1.14988
C -2.17450 -2.11626 -0.19710
C -3.46421 -1.36499 -2.37194
H -2.60362 -1.66034 -2.99410
H -3.89749 -0.44557 -2.79442
H -4.22027 -2.16732 -2.46950
C -4.66292 0.77865 -0.30236
H -5.64920 0.30463 -0.14161
H -4.64774 1.15195 -1.33916
H -4.61190 1.64593 0.37217
C -3.09315 0.35498 2.49329
H -3.38876 1.39760 2.29780
H -2.20700 0.36002 3.14715
H -3.91977 -0.11950 3.05672
C -1.46356 -2.39651 2.30501
H -2.19381 -3.09532 2.75435
H -1.13846 -1.69973 3.09276
H -0.58623 -2.98243 1.99060
C -1.59231 -3.37218 -0.77546
H -2.35178 -4.17616 -0.79149
H -0.73499 -3.72895 -0.18529
H -1.25270 -3.22367 -1.81363
B 0.80392 -0.69108 0.36245
H 0.87713 -1.11271 1.49750
H 0.73584 -1.59553 -0.47263
P 2.58396 0.10064 0.00274
H 2.65317 1.53449 -0.09668
C 3.31952 -0.38598 -1.70962
C 2.17188 -0.30190 -2.74275
H 1.76914 0.72059 -2.83779
H 1.34204 -0.98412 -2.50205
H 2.57186 -0.58667 -3.73276
C 4.43700 0.59339 -2.13196
H 4.78361 0.32300 -3.14578
H 5.31170 0.55993 -1.46654
H 4.07601 1.63543 -2.17532
C 3.85203 -1.83397 -1.65226
H 3.08422 -2.54404 -1.30143
H 4.73797 -1.92574 -1.00313
H 4.15599 -2.14606 -2.66747
C 3.77429 -0.15028 1.49181
C 5.20265 0.34932 1.19817
H 5.78955 0.31467 2.13391
H 5.21895 1.39162 0.83721
H 5.72531 -0.28631 0.46556
C 3.15708 0.69927 2.62895

H 2.11295 0.41439 2.84005
H 3.19502 1.77937 2.40247
H 3.74108 0.53799 3.55291
C 3.80268 -1.63642 1.91248
H 2.79517 -2.02112 2.13613
H 4.41337 -1.73268 2.82800
H 4.25742 -2.28102 1.14399
P -0.76985 2.21297 -0.17880
C 0.24414 2.90198 -1.58957
H 0.28648 4.00158 -1.51928
H -0.21926 2.61739 -2.54705
H 1.26670 2.50043 -1.55710
C -2.27701 3.30855 -0.30661
H -1.96995 4.36649 -0.35178
H -2.91881 3.16527 0.57564
H -2.85200 3.05798 -1.21060
C 0.05258 2.95680 1.32010
H 1.03883 2.49720 1.47526
H -0.56953 2.74634 2.20399
H 0.16408 4.04763 1.20368

11_{tBu}
SCF Energy = -975.891367404
Enthalpy 0K = -975.292879
Enthalpy 298K = -975.254147
Free Energy 298K = -975.359464
SCF(DCM) = -975.937370782
SCF(BP86-D3) = -0.11174809
Lowest Frequencies = 21.4642
29.4636 cm-1
C -2.07785 -1.66683 1.05558
C -2.03733 -2.12623 -0.30309
C -2.93226 -1.27177 -1.10438
C -3.51075 -0.29708 -0.24979
C -2.91146 -0.46576 1.07917
Rh -1.14926 -0.05706 -0.19347
B 0.80475 -0.60920 -0.19087
P 2.60492 0.17596 -0.03324
C 3.67636 -0.12468 -1.60285
C 5.15455 0.24330 -1.36947
C -1.37645 -3.37992 -0.80051
C -3.25674 -1.49089 -2.55430
C -4.62273 0.64699 -0.61304
C -3.34943 0.24376 2.33043
C -1.48373 -2.35158 2.25427
P -0.83514 2.23009 0.00577
C -2.38419 3.27135 -0.02700
C -0.02315 2.91228 1.54385
C 0.12492 3.05419 -1.36654
C 3.39455 -0.37061 1.62659
C 2.26962 -0.30236 2.68685
C 3.91897 -1.81936 1.52045
C 4.52873 0.59789 2.02845
C 3.53630 -1.59947 -2.03836
C 3.07832 0.79766 -2.69096
H -2.38281 -1.84913 -3.12047
H -3.62074 -0.56962 -3.03490
H -4.04934 -2.25578 -2.65910
H -5.59720 0.12805 -0.54399
H -4.52822 1.02270 -1.64492
H -4.67362 1.51339 0.06277
H -3.66664 1.27944 2.13177
H -2.54984 0.26793 3.08742
H -4.21523 -0.27799 2.78066
H -2.21556 -3.05503 2.69482
H -1.20549 -1.63231 3.04034
H -0.58734 -2.93236 1.98746
H -2.07322 -4.23549 -0.72919
H -0.48109 -3.63172 -0.21085
H -1.07256 -3.29013 -1.85554
H 1.02836 -1.80035 -0.08192
H 2.65783 1.60573 0.07314
H 3.20720 1.86593 -2.44543
H 2.00732 0.59862 -2.86628
H 3.60924 0.61386 -3.64209
H 5.69735 0.14820 -2.32734
H 5.64484 -0.43018 -0.64924
H 5.27927 1.28321 -1.02299
H 2.48714 -1.86828 -2.24200
H 3.93130 -2.30119 -1.28748
H 4.11138 -1.74952 -2.96921
H 4.91211 0.29933 3.02065
H 4.17042 1.63796 2.11381
H 5.37795 0.58012 1.32939
H 1.42877 -0.97300 2.44744
H 1.86984 0.71875 2.80454
H 2.68924 -0.60782 3.66248
H 3.13467 -2.52438 1.19942
H 4.26744 -2.14159 2.51786
H 4.77513 -1.90853 0.83234
H 0.20356 4.14026 -1.19297
H -0.39875 2.87206 -2.31799
H 1.13373 2.62406 -1.44234
H -2.11715 4.34039 0.01337
H -3.01922 3.03256 0.83936
H -2.94928 3.07309 -0.94982
H 1.01707 2.56086 1.60976
H -0.57017 2.54034 2.42401
H -0.03492 4.01499 1.54226
H 0.21587 -0.20997 -1.33849

$TS(11\text{-}11')_{tBu}$
SCF Energy = -975.885463285
Enthalpy 0K = -975.288537
Enthalpy 298K = -975.250093
Free Energy 298K = -975.354129
SCF(DCM) = -975.931920910
SCF(BP86-D3) = -0.11181336
Lowest Frequencies = -372.7439
28.2325 cm-1
C -2.13586 -1.46628 1.35751
C -1.93081 -2.15082 0.10354
C -2.75718 -1.49151 -0.91288
C -3.44911 -0.41014 -0.29310
C -3.01974 -0.34653 1.10469
Rh -1.09739 -0.06080 -0.12384
B 0.77527 -0.51210 0.27569
P 2.60026 0.20110 -0.05401
C 3.41536 -0.55184 -1.62618
C 4.92973 -0.26736 -1.67643
C -1.20254 -3.45336 -0.08130
C -2.94799 -1.97652 -2.32366

C -4.54313 0.40589 -0.92577
C -3.61259 0.54080 2.16616
C -1.62446 -1.90837 2.70100
P -0.86846 2.23092 -0.30552
C -2.44983 3.20970 -0.45978
C -0.04657 3.13971 1.10311
C 0.05833 2.86986 -1.79259
C 3.59787 0.06439 1.57505
C 2.62543 0.49825 2.69921
C 4.04336 -1.39560 1.80568
C 4.81188 1.01778 1.55348
C 3.13429 -2.06996 -1.65669
C 2.72099 0.13574 -2.82508
H -2.02936 -2.42746 -2.72962
H -3.25063 -1.16052 -2.99811
H -3.73916 -2.74876 -2.36143
H -5.49990 -0.14796 -0.88904
H -4.33695 0.62500 -1.98619
H -4.70664 1.36064 -0.40426
H -3.98015 1.49321 1.75277
H -2.88459 0.77197 2.95989
H -4.47710 0.04421 2.64550
H -2.32313 -2.63329 3.15913
H -1.52263 -1.06235 3.39851
H -0.64414 -2.40389 2.62003
H -1.87808 -4.30412 0.12495
H -0.34393 -3.54249 0.60307
H -0.83056 -3.57053 -1.11152
H 1.01582 -1.59496 0.78088
H 2.67518 1.60638 -0.33173
H 2.93781 1.21699 -2.86382
H 1.62716 -0.00774 -2.81603
H 3.10787 -0.31078 -3.75849
H 5.32080 -0.62672 -2.64556
H 5.48756 -0.79459 -0.88624
H 5.15625 0.81017 -1.61137
H 2.05307 -2.28567 -1.66188
H 3.58844 -2.60163 -0.80585
H 3.56610 -2.49145 -2.58168
H 5.31252 0.97303 2.53724
H 4.51085 2.06591 1.38523
H 5.55699 0.74362 0.79209
H 1.73105 -0.14528 2.75390
H 2.29524 1.54416 2.58223
H 3.15192 0.42707 3.66818
H 3.19244 -2.09662 1.80505
H 4.52994 -1.46356 2.79486
H 4.77736 -1.73232 1.05653
H 0.11046 3.97095 -1.77922
H -0.46347 2.53674 -2.70315
H 1.07758 2.45725 -1.81066
H -2.21593 4.28055 -0.57969
H -3.06424 3.07918 0.44387
H -3.01993 2.86284 -1.33410
H 0.99722 2.80529 1.19767
H -0.57396 2.89386 2.03758
H -0.06808 4.23026 0.94091
H -0.10267 -0.19360 -1.37883

$11'_{tBu}$
SCF Energy = -975.886749414
Enthalpy 0K = -975.288295
Enthalpy 298K = -975.249548
Free Energy 298K = -975.354732
SCF(DCM) = -975.933375459
SCF(BP86-D3) = -0.11245979
Lowest Frequencies = 22.7981
24.0440 cm-1
C -1.70148 -2.01303 0.82499
C -2.41023 -1.88543 -0.44642
C -3.31182 -0.77178 -0.34957
C -3.13193 -0.17691 0.96472
C -2.16329 -0.95782 1.69959
Rh -1.05691 -0.05676 -0.14944
P -1.03642 2.16914 -0.73591
C -1.12687 3.38701 0.67224
C -2.35185 -2.87257 -1.58046
C -4.37698 -0.41973 -1.35335
C -3.98172 0.92050 1.54623
C -1.80203 -0.80005 3.15076
C -0.85704 -3.18629 1.24049
B 0.77973 -0.19197 0.54020
P 2.58452 0.11358 -0.24248
C 3.71942 1.03494 0.99974
C 4.24850 0.05625 2.06943
C 3.28916 -1.49536 -1.01739
C 3.11698 -2.66257 -0.02132
C 2.43842 -1.75042 -2.28521
C 4.76868 -1.32368 -1.41394
C 4.88000 1.73691 0.26415
C 2.81146 2.09651 1.66633
C 0.38054 2.83882 -1.74105
C -2.47524 2.68979 -1.80178
H -1.36716 -3.35964 -1.65097
H -2.56371 -2.39450 -2.54926
H -3.10438 -3.66938 -1.43125
H -5.27904 -1.03982 -1.19256
H -4.04300 -0.59489 -2.38846
H -4.69559 0.63121 -1.27532
H -4.29730 1.65383 0.78691
H -3.46441 1.46083 2.35495
H -4.90507 0.49438 1.98098
H -2.43649 -1.44647 3.78550
H -1.93877 0.23694 3.49532
H -0.75377 -1.08259 3.34047
H -1.49252 -3.96987 1.69388
H -0.10263 -2.90374 1.99153
H -0.33872 -3.64323 0.38283
H 1.00117 -0.72665 1.61318
H 2.59181 0.99036 -1.37358
H 2.57147 -0.95584 -3.03911
H 1.36333 -1.83626 -2.05744
H 2.76985 -2.70006 -2.74213
H 5.09598 -2.23494 -1.94667
H 5.42927 -1.20267 -0.54081
H 4.92299 -0.47120 -2.09655
H 2.06109 -2.81155 0.25743
H 3.70096 -2.52061 0.90179
H 3.47339 -3.59182 -0.50016
H 5.46678 2.31868 0.99765
H 4.51635 2.44348 -0.50140
H 5.56759 1.02746 -0.21974

H 1.96317 1.63858 2.20356
H 2.41598 2.82250 0.93469
H 3.40584 2.66693 2.40279
H 3.43288 -0.47622 2.58631
H 4.80653 0.62938 2.83090
H 4.94221 -0.68820 1.64678
H 0.16770 3.87704 -2.04343
H 0.51928 2.21626 -2.63808
H 1.30480 2.82593 -1.14556
H -2.41306 3.76657 -2.03141
H -3.42077 2.48748 -1.27778
H -2.46069 2.11217 -2.73869
H -0.24602 3.25474 1.31882
H -2.02891 3.18165 1.26832
H -1.16458 4.42412 0.29948
H -0.30930 -0.23763 -1.52337

TS(11’’-9')1_{tBu}
SCF Energy = -975.846610588
Enthalpy 0K = -975.246999
Enthalpy 298K = -975.209592
Free Energy 298K = -975.309851
SCF(DCM) = -975.894832179
SCF(BP86-D3) = -0.12056329
Lowest Frequencies = -94.5339
22.4288 cm-1
C -1.82204 -1.59427 -1.21709
C -2.58383 -0.38140 -0.98308
C -2.92034 -0.34175 0.46735
C -2.26622 -1.42405 1.09564
C -1.60890 -2.21469 0.05319
Rh -0.66510 0.08812 -0.09168
B 0.78746 0.23631 -1.74478
P 2.06971 -0.10270 -0.27975
C 3.61363 1.12643 -0.14092
C 3.78889 1.61069 1.30917
C -3.32192 0.34579 -2.07422
C -3.99069 0.51131 1.08638
C -2.35957 -1.82673 2.54157
C -1.13974 -3.62508 0.26902
C -1.55793 -2.20218 -2.56512
P -0.92344 2.37418 0.27870
C -2.21753 3.29297 -0.70627
C -1.41026 2.72173 2.05134
C 0.44238 3.62561 0.09606
C 2.44972 -1.90556 0.40655
C 2.05811 -2.87533 -0.72669
C 3.91601 -2.18958 0.79475
C 1.59981 -2.08768 1.68055
C 4.88573 0.39005 -0.63450
C 3.40137 2.29486 -1.12312
H -4.01427 1.54023 0.69787
H -3.90345 0.55657 2.18320
H -4.97961 0.06912 0.86003
H -3.10744 -2.63024 2.67969
H -2.66004 -0.98413 3.18351
H -1.40091 -2.21301 2.92516
H -0.54604 -3.75128 1.18666
H -0.56450 -4.01772 -0.58057
H -2.03444 -4.26785 0.38058
H -2.40063 -2.86090 -2.84895
H -0.64231 -2.81341 -2.58108
H -1.45411 -1.43442 -3.34558
H -4.16210 -0.28153 -2.42926
H -2.67312 0.54811 -2.94035
H -3.75225 1.29760 -1.73585
H 0.66859 -0.67411 -2.52637
H 1.17870 0.36479 0.86140
H 2.49106 2.87121 -0.94127
H 3.37730 1.94313 -2.16635
H 4.25928 2.98356 -1.01669
H 4.67825 2.26339 1.37430
H 3.94225 0.77335 2.01022
H 2.92285 2.19442 1.66415
H 4.70966 -0.19605 -1.55322
H 5.33827 -0.25734 0.12497
H 5.63359 1.16122 -0.89094
H 1.65882 -3.14045 2.01101
H 0.54059 -1.82675 1.52210
H 1.98098 -1.45830 2.50333
H 2.75184 -2.78749 -1.57918
H 1.04365 -2.68999 -1.10514
H 2.10960 -3.91451 -0.35467
H 4.59388 -2.19265 -0.06967
H 3.94468 -3.20461 1.23104
H 4.30219 -1.50018 1.56232
H 0.06763 4.60266 0.44229
H 0.72924 3.70648 -0.96185
H 1.31458 3.34715 0.70294
H -2.20833 4.35416 -0.40861
H -3.22480 2.89161 -0.53459
H -1.97814 3.21337 -1.77778
H -0.57396 2.42833 2.70543
H -2.29452 2.13486 2.33425
H -1.62181 3.79503 2.19155
H 1.01694 1.33038 -2.20840

INT(11’’-9')$_{tBu}$
SCF Energy = -975.878740512
Enthalpy 0K = -975.278097
Enthalpy 298K = -975.239799
Free Energy 298K = -975.341487
SCF(DCM) = -975.925785814
SCF(BP86-D3) = -0.12392235
Lowest Frequencies = 28.3665
44.8960 cm-1
C -1.27055 -2.15169 -0.69568
C -2.42390 -1.37424 -0.35533
C -2.36296 -1.04287 1.07227
C -1.14563 -1.60844 1.59507
C -0.48274 -2.31034 0.51582
Rh -0.59851 0.03194 0.11568
B 0.74494 1.19765 -1.60474
P 1.81212 0.41595 -0.28470
C 2.65758 1.43774 1.13615
C 1.89185 2.76022 1.31901
C -3.60853 -1.21231 -1.26606
C -3.50005 -0.57342 1.94087
C -0.76665 -1.67969 3.04963
C 0.59074 -3.33261 0.76045
C -1.09079 -2.86705 -2.01006
P -1.70456 2.00391 -0.60957

```
C  -2.19030  2.15052 -2.40460
C  -3.38413  2.19568  0.19726
C  -1.08676  3.70685 -0.15816
C   3.03781 -0.87187 -1.08774
C   2.24980 -1.77357 -2.05481
C   4.05040 -0.07057 -1.94792
C   3.78442 -1.72438 -0.04222
C   2.59034  0.61704  2.44330
C   4.12611  1.79105  0.80606
H  -4.32541 -0.13679  1.36282
H  -3.17958  0.16201  2.69573
H  -3.91317 -1.44062  2.48888
H  -1.17901 -2.59742  3.50964
H  -1.16016 -0.82018  3.61328
H   0.32504 -1.70313  3.18924
H   1.29111 -3.01465  1.54679
H   1.16537 -3.59807 -0.13679
H   0.10339 -4.25919  1.12076
H  -1.99008 -3.46553 -2.24202
H  -0.23908 -3.56235 -1.98620
H  -0.93168 -2.17260 -2.85166
H  -4.22981 -2.12718 -1.22155
H  -3.30298 -1.08338 -2.31608
H  -4.26279 -0.37358 -0.98666
H   0.60050  0.57243 -2.62236
H  -0.26863  0.99486  1.29675
H   4.21469  2.37994 -0.12108
H   4.77987  0.90940  0.73217
H   4.51995  2.41228  1.63082
H   2.35924  3.32547  2.14556
H   0.83716  2.59354  1.58948
H   1.93520  3.38698  0.41428
H   3.13257 -0.33948  2.36910
H   1.54663  0.40494  2.72796
H   3.05395  1.19826  3.26104
H   4.45121 -2.43114 -0.56902
H   3.10005 -2.31567  0.58300
H   4.41931 -1.11869  0.62268
H   1.82556 -1.20067 -2.89289
H   1.43140 -2.30450 -1.55749
H   2.94156 -2.52506 -2.47683
H   3.53979  0.55318 -2.69902
H   4.68350 -0.79800 -2.48933
H   4.72074  0.56861 -1.35926
H  -1.80318  4.43783 -0.56700
H  -0.08824  3.91408 -0.55832
H  -1.07137  3.80139  0.93842
H  -2.84231  3.02981 -2.53467
H  -2.74161  1.24612 -2.70379
H  -1.29593  2.24810 -3.03359
H  -3.27833  2.19475  1.29163
H  -4.06485  1.38988 -0.10291
H  -3.81759  3.15786 -0.12122
H   0.60665  2.38625 -1.62831
```

TS(11’’-6)2_{tBu}
SCF Energy = -975.873494954
Enthalpy 0K = -975.273296
Enthalpy 298K = -975.235756
Free Energy 298K = -975.335297
SCF(DCM) = -975.920298264
SCF(BP86-D3) = -0.12283801
Lowest Frequencies = -43.4147 30.2826 cm-1

```
C   1.23613 -1.92431  1.08628
C   2.41492 -1.36932  0.49754
C   2.25132 -1.33530 -0.95744
C   0.96317 -1.90569 -1.26012
C   0.33171 -2.26029 -0.00095
Rh  0.60902  0.05800 -0.19928
B  -0.87271  2.03033  0.56572
P  -1.80132  0.49628  0.07847
C  -2.83489  0.60963 -1.56364
C  -2.04480  1.43370 -2.59895
C   3.69134 -1.15906  1.26415
C   3.33013 -1.12655 -1.98676
C   0.51721 -2.32565 -2.63456
C  -0.86250 -3.16377  0.11393
C   1.13858 -2.34996  2.52747
P   1.85051  2.05358  0.24647
C   2.21320  2.43073  2.03638
C   3.58405  1.88975 -0.44621
C   1.49738  3.74458 -0.47445
C  -2.85537 -0.10224  1.61465
C  -1.86408 -0.49823  2.72281
C  -3.67889  1.11373  2.11093
C  -3.80625 -1.27551  1.30519
C  -3.09400 -0.80081 -2.12925
C  -4.17462  1.33031 -1.28486
H   4.19387 -0.57325 -1.59386
H   2.95628 -0.59112 -2.87379
H   3.70300 -2.10892 -2.33286
H   1.04909 -3.24864 -2.93340
H   0.73515 -1.55876 -3.39361
H  -0.55904 -2.54738 -2.67235
H  -1.62052 -2.95971 -0.65634
H  -1.34072 -3.10704  1.10212
H  -0.52659 -4.20913 -0.02604
H   1.93168 -3.08626  2.75834
H   0.17848 -2.83574  2.75391
H   1.26340 -1.50900  3.22902
H   3.99850 -2.12306  1.71142
H   3.58183 -0.45114  2.10207
H   4.52240 -0.82542  0.62642
H  -0.71728  2.26493  1.73384
H   0.38398  0.79783 -1.54899
H  -4.02437  2.33665 -0.86140
H  -4.83594  0.75918 -0.61576
H  -4.71142  1.44799 -2.24395
H  -2.65005  1.50343 -3.52109
H  -1.08756  0.95598 -2.86537
H  -1.84205  2.45859 -2.25073
H  -3.66187 -1.44005 -1.43626
H  -2.14665 -1.30339 -2.37917
H  -3.68212 -0.71792 -3.06119
H  -4.31472 -1.56952  2.24161
H  -3.28243 -2.16343  0.92205
H  -4.59544 -1.00220  0.58747
H  -1.13862  0.30446  2.93277
H  -1.30547 -1.40734  2.46530
H  -2.42782 -0.69543  3.65281
H  -3.02797  1.94669  2.41762
```

H -4.26523 0.79614 2.99338
H -4.39059 1.48838 1.36167
H 2.44032 4.31423 -0.48691
H 0.74988 4.28068 0.12270
H 1.12342 3.63362 -1.50364
H 3.00298 3.19668 2.10242
H 2.55061 1.51578 2.54687
H 1.30046 2.79427 2.52840
H 3.52924 1.76618 -1.53792
H 4.10906 1.03500 -0.00630
H 4.14741 2.80855 -0.21602
H -0.79919 2.88474 -0.26333

INT(11’’-6)2$_{tBu}$
SCF Energy = -975.874394716
Enthalpy 0K = -975.273643
Enthalpy 298K = -975.235387
Free Energy 298K = -975.337323
SCF(DCM) = -975.921239875
SCF(BP86-D3) = -0.12393654
Lowest Frequencies = 24.2308 45.9601 cm-1
C 0.95930 -1.66211 1.46606
C 2.23773 -1.19136 1.06896
C 2.40905 -1.45794 -0.36855
C 1.22696 -2.14166 -0.83200
C 0.30970 -2.23448 0.27718
Rh 0.62548 -0.01791 -0.28732
B -0.93408 2.03950 -0.58197
P -1.79262 0.46538 -0.09657
C -2.98669 -0.11525 -1.53341
C -2.11459 -0.59890 -2.70674
C 3.30357 -0.73496 2.02671
C 3.70609 -1.45724 -1.13364
C 1.09354 -2.82089 -2.16789
C -0.90501 -3.11899 0.30207
C 0.51441 -1.82820 2.89240
P 1.83324 2.04477 -0.30215
C 1.91199 2.96019 1.32006
C 3.65904 1.78884 -0.62466
C 1.57359 3.39200 -1.57286
C -2.64377 0.64398 1.64825
C -1.57651 1.22946 2.59544
C -3.81440 1.64571 1.51374
C -3.17590 -0.68185 2.22790
C -3.93575 -1.24594 -1.08837
C -3.82131 1.10301 -1.99934
H 4.48929 -0.85958 -0.64876
H 3.58363 -1.09401 -2.16636
H 4.08542 -2.49433 -1.19951
H 1.56958 -3.81871 -2.12851
H 1.58478 -2.24960 -2.97001
H 0.04287 -2.97277 -2.45656
H -1.36439 -3.21672 -0.69312
H -1.67187 -2.76748 1.00617
H -0.60310 -4.13464 0.62213
H 0.93577 -2.76351 3.30904
H -0.57779 -1.89627 2.99392
H 0.86313 -1.00417 3.53496
H 3.69469 -1.60735 2.58310
H 2.91678 -0.02613 2.77694
H 4.16393 -0.27116 1.52291
H -0.72722 2.88583 0.24304
H 0.45542 0.27596 -1.80476
H -3.18502 1.91305 -2.38833
H -4.45697 1.51514 -1.20103
H -4.49092 0.77618 -2.81654
H -2.77633 -0.88108 -3.54584
H -1.50945 -1.47736 -2.43489
H -1.43325 0.18887 -3.06651
H -4.66178 -0.90800 -0.33188
H -3.39973 -2.12362 -0.69485
H -4.52067 -1.57909 -1.96493
H -3.63482 -0.47605 3.21207
H -2.37595 -1.41878 2.39293
H -3.94880 -1.14640 1.59919
H -1.28345 2.24821 2.30041
H -0.67060 0.60366 2.62761
H -1.99759 1.28293 3.61574
H -3.49165 2.60195 1.07186
H -4.21613 1.85519 2.52258
H -4.64538 1.24189 0.91376
H 2.45559 4.05255 -1.56073
H 0.67002 3.97575 -1.36245
H 1.47692 2.92927 -2.56702
H 2.60283 3.81459 1.23169
H 2.28028 2.27689 2.10086
H 0.91385 3.32159 1.60198
H 3.80369 1.33848 -1.61722
H 4.10748 1.14239 0.13942
H 4.16117 2.76966 -0.59797
H -0.88598 2.29244 -1.75243

TS(11’’-6)2’$_{tBu}$
SCF Energy = -975.871852295
Enthalpy 0K = -975.271438
Enthalpy 298K = -975.234002
Free Energy 298K = -975.333110
SCF(DCM) = -975.918774957
SCF(BP86-D3) = -0.12309083
Lowest Frequencies = -76.6395 40.9283 cm-1
C 1.00111 -1.40137 1.68984
C 2.27395 -1.02213 1.17777
C 2.39836 -1.53062 -0.19549
C 1.21005 -2.30090 -0.48347
C 0.33640 -2.20528 0.66072
Rh 0.64370 -0.07782 -0.24508
B -0.95946 1.50592 -1.50425
P -1.79735 0.43501 -0.21835
C -3.09633 -0.66512 -1.18994
C -2.32032 -1.57251 -2.16324
C 3.37600 -0.47043 2.03822
C 3.65383 -1.59253 -1.02381
C 1.04974 -3.20001 -1.67864
C -0.85157 -3.07936 0.94078
C 0.59262 -1.28100 3.13318
P 1.82284 1.97250 -0.57613
C 1.37031 3.56912 0.28633
C 3.64008 1.96880 -0.12880
C 1.99942 2.46628 -2.36753
C -2.55319 1.32527 1.32790

```
C  -1.51428  2.35766  1.80151
C  -3.86304  2.05547  0.95573
C  -2.81446  0.31182  2.46068
C  -3.99369 -1.51773 -0.27062
C  -3.98721  0.29016 -2.02363
H   4.42425 -0.88940 -0.67545
H   3.45748 -1.38861 -2.08837
H   4.09449 -2.60506 -0.96107
H   1.60192 -4.14393 -1.51173
H   1.45150 -2.73997 -2.59461
H  -0.00007 -3.46562 -1.86830
H  -1.33666 -3.44527  0.02542
H  -1.60543 -2.57178  1.56061
H  -0.50952 -3.96893  1.50361
H   1.20722 -1.95673  3.75873
H  -0.45744 -1.56641  3.29083
H   0.73126 -0.26083  3.52568
H   3.67772 -1.25155  2.76160
H   3.05444  0.40123  2.63080
H   4.27254 -0.19902  1.46555
H  -0.64897  2.62719 -1.22532
H   0.46260  0.05145 -1.80009
H  -3.39660  0.87501 -2.74521
H  -4.56769  0.98744 -1.40294
H  -4.70995 -0.32144 -2.59516
H  -3.04689 -2.12035 -2.79062
H  -1.70432 -2.31255 -1.63406
H  -1.66459 -0.99756 -2.83618
H  -4.64734 -0.90137  0.36681
H  -3.42487 -2.20514  0.37299
H  -4.65763 -2.13424 -0.90367
H  -3.22864  0.84886  3.33348
H  -1.88269 -0.17515  2.78506
H  -3.53958 -0.46633  2.17992
H  -1.39568  3.17454  1.07382
H  -0.52791  1.89344  1.97291
H  -1.85857  2.80108  2.75339
H  -3.72682  2.75322  0.11371
H  -4.19581  2.64381  1.83076
H  -4.67926  1.35871  0.70881
H   2.60662  3.38321 -2.44015
H   1.01844  2.63382 -2.82995
H   2.51130  1.64951 -2.90052
H   2.12922  4.32785  0.03409
H   1.36844  3.40727  1.37483
H   0.38245  3.92888 -0.02688
H   4.16214  1.12759 -0.60418
H   3.78057  1.92851  0.95925
H   4.07585  2.90984 -0.50233
H  -1.05600  1.20095 -2.66386
```

6_{tBu}

```
SCF Energy = -975.873406916
Enthalpy 0K = -975.271860
Enthalpy 298K = -975.234240
Free Energy 298K = -975.334151
SCF(DCM) = -975.921616869
SCF(BP86-D3) = -0.12233296
Lowest Frequencies = 28.7385
43.6464 cm-1
C   2.92583 -0.06674 -0.58210
C   2.30842 -1.13882 -1.31940
C   1.74046 -2.08407 -0.37143
C   1.99575 -1.58932  0.95878
C   2.70462 -0.34321  0.84134
Rh  0.69207  0.06447 -0.29218
B  -0.93980 -0.68376 -1.90055
P  -1.85156 -0.31058 -0.25685
C  -2.56091 -1.78096  0.80610
C  -3.45017 -1.27107  1.96092
C   2.42269 -1.36997 -2.79915
C   1.27330 -3.45855 -0.75501
C   1.83353 -2.33030  2.25585
C   3.41279  0.30102  2.00480
C   3.90135  0.91167 -1.17956
P   0.56965  2.29705  0.46022
C  -0.04942  2.43443  2.21546
C   2.18891  3.22006  0.58997
C  -0.35908  3.65201 -0.42908
C  -3.26103  1.01145 -0.59853
C  -2.86961  1.84948 -1.84095
C  -4.56553  0.26876 -0.98191
C  -3.52909  1.91584  0.62233
C  -3.32885 -2.76887 -0.10288
C  -1.36793 -2.51059  1.43685
H   0.40306 -3.42913 -1.43311
H   1.01473 -4.07015  0.12163
H   2.08183 -3.98275 -1.29637
H   2.82050 -2.70604  2.58848
H   1.16635 -3.19811  2.16637
H   1.44936 -1.68334  3.06077
H   2.72658  0.67517  2.78226
H   4.06834  1.12585  1.69079
H   4.06030 -0.45003  2.49371
H   4.83257  0.37977 -1.45025
H   4.17887  1.71301 -0.48196
H   3.51450  1.37669 -2.10036
H   3.29136 -2.02356 -3.00472
H   2.57529 -0.42972 -3.34996
H   1.52745 -1.86536 -3.20398
H  -1.31602 -0.06746 -2.86967
H   0.11704  0.34476 -1.82465
H  -0.79099 -1.83933  2.09089
H  -0.69944 -2.92527  0.67171
H  -1.74421 -3.35106  2.04906
H  -3.74741 -2.13792  2.57898
H  -4.37741 -0.78686  1.62354
H  -2.90603 -0.57151  2.61792
H  -2.69440 -3.13578 -0.92586
H  -4.24074 -2.33717 -0.53858
H  -3.63515 -3.64178  0.50348
H  -4.35086  2.61547  0.38378
H  -2.65299  2.52017  0.89947
H  -3.83313  1.34352  1.51134
H  -3.05211  1.28769 -2.76902
H  -1.81866  2.16477 -1.85829
H  -3.50073  2.75659 -1.86936
H  -4.40742 -0.43056 -1.81856
H  -5.29443  1.02832 -1.32095
H  -5.03250 -0.27153 -0.14588
H  -0.09011  4.61353  0.03788
H  -1.44429  3.52119 -0.36767
```

```
H  -0.05621  3.67024 -1.48761
H  -0.06079  3.48693  2.54429
H   0.61887  1.85682  2.87338
H  -1.05984  2.00811  2.28924
H   2.61801  3.35490 -0.41328
H   2.90565  2.69430  1.23104
H   1.98641  4.21182  1.02610
H  -0.56291 -1.80821 -2.10050
```

6′$_{tBu}$

```
SCF Energy = -975.880531208
Enthalpy 0K = -975.278854
Enthalpy 298K = -975.241097
Free Energy 298K = -975.341493
SCF(DCM) = -975.928825744
SCF(BP86-D3) = -0.12343504
Lowest Frequencies = 20.2555
55.6507 cm-1
C   0.70073 -1.61052  1.52645
C   1.93303 -0.87982  1.45194
C   2.58399 -1.21797  0.17583
C   1.73114 -2.14333 -0.51786
C   0.57695 -2.40862  0.31390
Rh  0.58797 -0.12728 -0.21177
B  -0.87095  0.84639 -2.00476
P  -1.78566  0.42377 -0.36336
C  -3.09005 -0.95817 -0.80273
C  -2.41308 -1.97165 -1.75798
C   2.56396 -0.18639  2.62914
C   4.01437 -0.93783 -0.19200
C   2.03848 -2.83006 -1.81912
C  -0.31371 -3.61215  0.18079
C  -0.13423 -1.77729  2.76451
P   1.71425  2.00290 -0.52532
C   0.98282  3.70464 -0.83269
C   2.98751  2.54170  0.73326
C   2.72435  1.82849 -2.08607
C  -2.51327  1.68859  0.92305
C  -1.66464  2.96591  0.87290
C  -3.98171  2.06198  0.62989
C  -2.38838  1.07229  2.33327
C  -3.64396 -1.66321  0.45210
C  -4.25987 -0.32366 -1.60181
H   4.36024  0.05977  0.11640
H   4.18708 -1.03988 -1.27463
H   4.67177 -1.67288  0.30955
H   2.55307 -3.79116 -1.63374
H   2.69473 -2.22148 -2.45987
H   1.12240 -3.04899 -2.38908
H  -0.36821 -3.98743 -0.85120
H  -1.33744 -3.43829  0.54018
H   0.11591 -4.42354  0.79984
H   0.24497 -2.63880  3.34754
H  -1.19105 -1.97607  2.53424
H  -0.08790 -0.89443  3.41922
H   2.82019 -0.93891  3.39791
H   1.88413  0.54112  3.09989
H   3.49419  0.33209  2.36343
H  -0.55541  1.98182 -2.22751
H   0.32742  0.11692 -1.87402
H  -3.89540  0.27776 -2.45077
H  -4.92885  0.29639 -0.99110
H  -4.86900 -1.14730 -2.01824
H  -2.99261 -2.91209 -1.75402
H  -1.37714 -2.19912 -1.47849
H  -2.39965 -1.59292 -2.79030
H  -4.23242 -0.98311  1.08905
H  -2.84969 -2.10569  1.07485
H  -4.32203 -2.47948  0.14354
H  -2.77181  1.79481  3.07694
H  -1.33465  0.85984  2.57664
H  -2.96634  0.14237  2.44731
H  -1.72681  3.46551 -0.10567
H  -0.61093  2.74555  1.10368
H  -2.03530  3.67170  1.63856
H  -4.12218  2.44463 -0.39355
H  -4.27796  2.86491  1.32970
H  -4.67398  1.22325  0.79605
H   3.37525  2.70804 -2.21916
H   2.03107  1.75412 -2.93806
H   3.33989  0.91902 -2.04948
H   1.77614  4.30408 -1.30907
H   0.70189  4.17817  0.11770
H   0.11325  3.66232 -1.49904
H   3.78545  1.79848  0.85471
H   2.50129  2.70971  1.70607
H   3.43886  3.48680  0.39070
H  -1.25154  0.19027 -2.94371
```

TS(11′′-6)3$_{tBu}$

```
SCF Energy = -975.837106474
Enthalpy 0K = -975.241596
Enthalpy 298K = -975.203569
Free Energy 298K = -975.305793
SCF(DCM) = -975.883450862
SCF(BP86-D3) = -0.11466235
Lowest Frequencies = -829.5529
24.5673 cm-1
C  -2.21753 -1.42854  0.93639
C  -1.77986 -2.10290 -0.26923
C  -2.36435 -1.39457 -1.39433
C  -3.23801 -0.34871 -0.89211
C  -3.14007 -0.36474  0.53824
Rh -0.97178  0.00925 -0.26439
B   0.84770 -0.65883 -1.06813
P   2.31674  0.07029 -0.01169
C   3.81804  0.63200 -1.18453
C   5.09152  0.98845 -0.40050
C  -1.07957 -3.43050 -0.35215
C  -2.21657 -1.78977 -2.83445
C  -4.14252  0.50314 -1.73799
C  -4.00274  0.39420  1.50829
C  -2.01856 -1.90119  2.34700
P  -0.82927  2.23274  0.53379
C  -2.33405  3.25981  0.12893
C  -0.64835  2.47308  2.37282
C   0.51553  3.32168 -0.13515
C   2.90177 -1.19548  1.33388
C   1.64907 -1.81218  1.98805
C   3.77416 -2.32666  0.75168
C   3.67765 -0.41012  2.41708
C   4.08311 -0.49716 -2.20208
```

```
C    3.31282  1.87899 -1.93890
H   -1.21984 -2.21064 -3.03891
H   -2.37972 -0.93781 -3.51245
H   -2.96242 -2.56559 -3.09009
H   -5.04354 -0.06338 -2.03802
H   -3.64257  0.83292 -2.66305
H   -4.48387  1.40150 -1.20170
H   -4.55938  1.20957  1.02268
H   -3.43094  0.81375  2.35197
H   -4.75196 -0.29193  1.94515
H   -2.83840 -2.58653  2.63655
H   -2.02324 -1.06430  3.06284
H   -1.07250 -2.44839  2.46855
H   -1.82844 -4.23493 -0.47567
H   -0.50928 -3.65357  0.56191
H   -0.39114 -3.48454 -1.20936
H    0.90097 -1.59645 -1.82727
H    0.79958  0.53203  0.58601
H    3.19893  2.74967 -1.27307
H    2.35003  1.69735 -2.44810
H    4.04960  2.15242 -2.71630
H    5.83765  1.40669 -1.10183
H    5.55587  0.11030  0.07527
H    4.90582  1.75217  0.37356
H    3.17820 -0.75541 -2.77771
H    4.46527 -1.41618 -1.73363
H    4.84678 -0.15120 -2.92426
H    3.98606 -1.10915  3.21612
H    3.04967  0.37035  2.87929
H    4.58800  0.06871  2.02862
H    1.08453 -2.43594  1.27721
H    0.96706 -1.04354  2.38753
H    1.96985 -2.45526  2.82872
H    3.25724 -2.87511 -0.05313
H    4.00654 -3.05054  1.55487
H    4.73673 -1.96063  0.36157
H    0.43280  4.32715  0.30796
H    0.42372  3.39234 -1.22945
H    1.49267  2.88626  0.11929
H   -2.18872  4.29127  0.48995
H   -3.22827  2.83575  0.60614
H   -2.47920  3.27347 -0.96199
H    0.31499  2.04928  2.69531
H   -1.45772  1.95030  2.90417
H   -0.68247  3.54487  2.62836
H    0.10579  0.38217 -1.57250
```

INT(11′′-6)$_{tBu}$
SCF Energy = -975.879430036
Enthalpy 0K = -975.281619
Enthalpy 298K = -975.243173
Free Energy 298K = -975.347513
SCF(DCM) = -975.929493073
SCF(BP86-D3) = -0.11019777
Lowest Frequencies = 17.7088 34.2506 cm-1

```
C    2.81848 -1.31188 -0.81293
C    1.92013 -2.21780 -0.10072
C    1.87129 -1.80227  1.27302
C    2.75249 -0.64009  1.43590
C    3.37216 -0.38389  0.15563
Rh   1.12131 -0.08842  0.00072
B   -1.04394 -0.51132 -0.03225
P   -2.73526  0.41821 -0.37012
C   -3.40088  0.83655  1.43255
C   -4.90989  1.14500  1.34850
C    1.22019 -3.40732 -0.68582
C    1.13103 -2.49292  2.37998
C    3.09845  0.01181  2.74362
C    4.53243  0.53647 -0.09520
C    3.25752 -1.46488 -2.23975
P    1.19059  2.15212 -0.69998
C    2.24640  3.29286  0.32360
C    1.88465  2.31877 -2.41857
C   -0.39631  3.09912 -0.81090
C   -3.79515 -0.97556 -1.22080
C   -2.92270 -1.45580 -2.40487
C   -4.17233 -2.17661 -0.33513
C   -5.06649 -0.31329 -1.80098
C   -3.14928 -0.28090  2.46692
C   -2.65985  2.11518  1.87658
H    0.23654 -3.01470  2.00876
H    0.82053 -1.78974  3.16790
H    1.78874 -3.24730  2.85061
H    3.91269 -0.54342  3.24627
H    2.23793  0.02692  3.43011
H    3.44046  1.04911  2.60726
H    4.64528  1.28906  0.69898
H    4.47069  1.05304 -1.06625
H    5.46392 -0.05933 -0.10923
H    4.06050 -2.22282 -2.30847
H    3.65788 -0.52552 -2.65005
H    2.43338 -1.79867 -2.88871
H    1.78359 -4.33002 -0.45602
H    1.13209 -3.33267 -1.78022
H    0.20687 -3.52427 -0.26865
H   -1.11238 -1.71207  0.09960
H   -0.17958 -0.24611 -1.11356
H   -2.86541  2.96118  1.19970
H   -1.56721  1.96124  1.93287
H   -3.00084  2.40380  2.88851
H   -5.25336  1.53938  2.32338
H   -5.50748  0.24451  1.13434
H   -5.13626  1.90423  0.58128
H   -2.07096 -0.46264  2.61393
H   -3.61938 -1.23440  2.18316
H   -3.57362  0.02081  3.44408
H   -5.61142 -1.04679 -2.42420
H   -4.81732  0.55142 -2.43923
H   -5.75835  0.03088 -1.01720
H   -2.01352 -1.98218 -2.06595
H   -2.61876 -0.61689 -3.05654
H   -3.50276 -2.16415 -3.02501
H   -3.28459 -2.65344  0.11349
H   -4.68933 -2.94142 -0.94531
H   -4.86148 -1.89135  0.47631
H   -0.22941  4.02207 -1.39027
H   -0.73031  3.36037  0.20387
H   -1.18268  2.48271 -1.27753
H    2.18311  4.31524 -0.08424
H    3.29641  2.96889  0.31512
H    1.87471  3.29343  1.35978
```

H 1.20659 1.81438 -3.12423
H 2.87323 1.83959 -2.47612
H 1.97782 3.38199 -2.69472
H -0.37974 0.21088 0.89921

TS(11''-6)4_{tBu}
SCF Energy = -975.858656966
Enthalpy 0K = -975.261138
Enthalpy 298K = -975.222776
Free Energy 298K = -975.326488
SCF(DCM) = -975.906969151
SCF(BP86-D3) = -0.11226886
Lowest Frequencies = -75.1671
22.5702 cm-1
C -3.14809 -0.00710 0.93833
C -2.46273 -1.14724 1.53390
C -2.09073 -2.04056 0.47210
C -2.56642 -1.48030 -0.79390
C -3.23480 -0.24639 -0.51273
Rh -1.09316 0.01181 0.16607
B 1.00812 -0.64298 1.24113
P 2.41995 -0.06549 0.09395
C 2.84369 -1.41213 -1.23727
C 4.03423 -0.95463 -2.10618
C -2.21960 -1.37265 2.99691
C -1.46292 -3.39334 0.63435
C -2.48091 -2.15735 -2.12925
C -4.08422 0.51421 -1.49212
C -3.87382 1.05916 1.70262
P -0.65898 2.16816 -0.63770
C -0.17987 2.27532 -2.43579
C -2.07487 3.37977 -0.54377
C 0.66966 3.16064 0.19984
C 3.96414 0.65227 1.02741
C 3.42889 1.37607 2.28222
C 4.91153 -0.48396 1.47645
C 4.72009 1.66532 0.13949
C 3.15665 -2.77263 -0.57110
C 1.60327 -1.55976 -2.14319
H -0.89199 -3.47009 1.57126
H -0.78798 -3.64000 -0.20065
H -2.25288 -4.16730 0.65765
H -3.32987 -2.85683 -2.25429
H -1.55728 -2.74742 -2.23399
H -2.52500 -1.43568 -2.95926
H -3.60164 0.60830 -2.47794
H -4.34136 1.52011 -1.13080
H -5.03756 -0.02428 -1.64680
H -4.85304 0.67267 2.04430
H -4.07153 1.95001 1.08839
H -3.31417 1.37193 2.59816
H -2.96371 -2.08024 3.40613
H -2.29531 -0.43664 3.57044
H -1.21897 -1.79930 3.17603
H 1.14209 -0.68864 2.44381
H 0.04715 0.39698 1.34155
H 1.37689 -0.62510 -2.68259
H 0.70589 -1.85134 -1.57259
H 1.79736 -2.34768 -2.89471
H 4.19577 -1.69400 -2.91188
H 4.97225 -0.89122 -1.53301
H 3.84841 0.02295 -2.58180
H 2.30522 -3.13530 0.02630
H 4.03808 -2.72600 0.08587
H 3.36934 -3.52174 -1.35737
H 5.56954 2.08481 0.70932
H 4.07467 2.50789 -0.16111
H 5.12950 1.20852 -0.77415
H 2.90358 0.69118 2.96573
H 2.74864 2.20691 2.03232
H 4.28562 1.80966 2.82989
H 4.39448 -1.22719 2.10524
H 5.72916 -0.04508 2.07948
H 5.38168 -1.00639 0.62830
H 0.72027 4.16901 -0.24185
H 1.63272 2.64728 0.06510
H 0.44588 3.23867 1.27486
H -0.08117 3.33044 -2.74131
H -0.94369 1.78478 -3.05840
H 0.78143 1.75935 -2.57506
H -2.39944 3.49356 0.50165
H -2.92177 3.03735 -1.15472
H -1.74029 4.35976 -0.92154
H 0.55117 -1.63930 0.71163

TS(11''-9')2_{tBu}
SCF Energy = -975.867013891
Enthalpy 0K = -975.266539
Enthalpy 298K = -975.229255
Free Energy 298K = -975.328983
SCF(DCM) = -975.910851044
SCF(BP86-D3) = -0.12100372
Lowest Frequencies = -109.9520
21.1844 cm-1
C -1.68594 -1.96082 -0.92236
C -2.73351 -1.11352 -0.39549
C -2.53345 -0.92992 1.01890
C -1.34173 -1.68262 1.38747
C -0.84606 -2.34548 0.17550
Rh -0.57173 -0.06478 0.18551
B 0.89316 1.71459 -0.99541
P 1.71739 0.20494 -0.12594
C 2.80840 0.62285 1.42889
C 2.16792 1.79504 2.19630
C -3.95592 -0.74559 -1.18651
C -3.50649 -0.33094 1.99999
C -0.93576 -2.02885 2.79399
C 0.15513 -3.46618 0.18416
C -1.68361 -2.52250 -2.31878
P -1.04293 2.53517 -0.50010
C -0.86683 3.83668 -1.84512
C -2.88401 2.28634 -0.69417
C -0.95936 3.54385 1.05722
C 2.71265 -0.78031 -1.48093
C 1.69524 -1.27802 -2.52439
C 3.69691 0.18205 -2.19096
C 3.47604 -1.98024 -0.88307
C 2.84763 -0.62060 2.34472
C 4.24024 1.04135 1.02715
H -4.14784 0.43967 1.54387
H -2.99264 0.12338 2.86116
H -4.18084 -1.11414 2.39429

```
H  -1.53069 -2.88875  3.15616
H  -1.10421 -1.19122  3.48780
H   0.12485 -2.31502  2.85591
H   0.93584 -3.32401  0.94640
H   0.64180 -3.61387 -0.79099
H  -0.37024 -4.40869  0.43083
H  -2.55597 -3.18676 -2.46566
H  -0.78516 -3.12272 -2.52334
H  -1.74346 -1.73478 -3.08791
H  -4.57198 -1.65563 -1.31981
H  -3.71292 -0.38445 -2.19940
H  -4.58722  0.00041 -0.68419
H   0.87157  1.67970 -2.19766
H  -0.27859  0.74744  1.48611
H   4.24428  1.91248  0.35177
H   4.80974  0.22556  0.55756
H   4.78718  1.33013  1.94303
H   2.75394  1.97462  3.11581
H   1.13069  1.57785  2.50092
H   2.17872  2.72661  1.60782
H   3.29096 -1.49867  1.84916
H   1.83757 -0.88749  2.69526
H   3.46367 -0.39723  3.23471
H   3.95982 -2.53980 -1.70441
H   2.80952 -2.67905 -0.35383
H   4.27514 -1.67352 -0.19004
H   1.13470 -0.44826 -2.98361
H   0.97355 -1.98212 -2.08952
H   2.24206 -1.80029 -3.33034
H   3.17339  1.03927 -2.64269
H   4.19615 -0.37416 -3.00608
H   4.48466  0.56522 -1.52816
H  -1.61796  4.41987  0.94474
H   0.07079  3.88325  1.23430
H  -1.29397  2.93641  1.90998
H  -1.62691  4.62015 -1.69418
H  -1.02280  3.36419 -2.82731
H   0.13955  4.27623 -1.81864
H  -3.27693  1.72436  0.16036
H  -3.08620  1.73899 -1.62452
H  -3.36489  3.27727 -0.74677
H   1.26166  2.74265 -0.47414
```

9'$_{tBu}$

```
SCF Energy = -975.885874932
Enthalpy 0K = -975.286815
Enthalpy 298K = -975.248328
Free Energy 298K = -975.351644
SCF(DCM) = -975.934307615
SCF(BP86-D3) = -0.11795984
Lowest Frequencies = 24.3688
40.8081 cm-1
C  -2.74586  0.21667 -1.20501
C  -2.79863  1.31364 -0.20768
C  -2.79016  0.75186  1.10042
C  -2.60204 -0.69595  0.93519
C  -2.68079 -1.01088 -0.51278
Rh -0.82954  0.23194  0.19691
B   2.56240  0.59427 -0.67078
P   1.28035 -0.82704 -0.05536
C   2.02057 -1.55818  1.59595
C   2.36122 -0.37990  2.52923
C  -3.00725  2.75836 -0.55568
C  -3.01133  1.47055  2.40252
C  -2.70324 -1.70715  2.03428
C  -2.83791 -2.38965 -1.08380
C  -2.86628  0.42195 -2.68689
P   2.26438  2.47919 -0.27411
C   3.62175  3.44262 -1.08265
C   0.69915  3.10967 -1.02742
C   2.23272  3.08790  1.47281
C   1.39998 -2.12715 -1.51105
C   0.58256 -1.52409 -2.67420
C   2.84938 -2.34683 -2.00314
C   0.78689 -3.48018 -1.09513
C   0.96739 -2.44858  2.28714
C   3.31231 -2.36321  1.33896
H  -2.68602  2.52101  2.34943
H  -2.46622  0.99141  3.22990
H  -4.08608  1.46656  2.66212
H  -3.76369 -1.99741  2.17000
H  -2.34515 -1.30516  2.99421
H  -2.13637 -2.62200  1.80588
H  -2.38728 -3.15624 -0.43584
H  -2.39604 -2.48538 -2.08749
H  -3.91384 -2.63233 -1.17311
H  -3.90428  0.69790 -2.95166
H  -2.61088 -0.48787 -3.25018
H  -2.21629  1.23793 -3.04413
H  -4.08351  2.94860 -0.72960
H  -2.47903  3.04361 -1.47941
H  -2.68339  3.42855  0.25531
H   2.53004  0.63384 -1.88602
H  -0.06052  0.73510  1.47864
H   4.06865 -1.77456  0.79477
H   3.12636 -3.29794  0.78764
H   3.75130 -2.64472  2.31363
H   2.71875 -0.78237  3.49439
H   1.47884  0.24831  2.73789
H   3.16288  0.25176  2.11410
H   0.66093 -3.30560  1.66659
H   0.06970 -1.86566  2.54643
H   1.39183 -2.85360  3.22430
H   0.74511 -4.14675 -1.97634
H  -0.23957 -3.37370 -0.71030
H   1.39072 -3.99402 -0.33049
H   1.02358 -0.58110 -3.03843
H  -0.46105 -1.32500 -2.38128
H   0.57625 -2.23612 -3.51985
H   3.33483 -1.40429 -2.29793
H   2.81777 -2.99974 -2.89517
H   3.48583 -2.84484 -1.25842
H   2.14489  4.18596  1.48403
H   3.16207  2.79118  1.98218
H   1.37511  2.64192  1.99854
H   3.45302  4.52504 -0.97195
H   3.66283  3.17900 -2.15037
H   4.58110  3.17013 -0.61658
H  -0.13883  2.52609 -0.59232
H   0.72770  2.94209 -2.11511
H   0.54837  4.17997 -0.81583
H   3.67341  0.33099 -0.25342
```

TS(9'-9)$_{tBu}$
SCF Energy = -975.881893429
Enthalpy 0K = -975.283866
Enthalpy 298K = -975.245460
Free Energy 298K = -975.349903
SCF(DCM) = -975.930430668
SCF(BP86-D3) = -0.11511837
Lowest Frequencies = -28.2266
22.4566 cm-1

```
C  -2.74962 -0.53751  1.20321
C  -2.60216 -1.67538  0.26516
C  -2.70531 -1.19321 -1.07150
C  -2.76203  0.27248 -0.98511
C  -2.89713  0.64458  0.44501
Rh -0.86535 -0.29227 -0.20783
B   2.11752 -0.40274  1.02388
P   1.05587  1.01370  0.06287
C   1.88664  1.65264 -1.57568
C   2.42614  0.43453 -2.34938
C  -2.49570 -3.10791  0.69763
C  -2.80282 -2.00565 -2.33301
C  -3.00488  1.19958 -2.13697
C  -3.28277  2.00739  0.94054
C  -2.83062 -0.68180  2.69530
P   2.72619 -2.14700  0.36192
C   3.01995 -3.16068  1.88468
C   1.59892 -3.18601 -0.67456
C   4.36473 -2.20652 -0.50274
C   0.96304  2.43789  1.40256
C   0.14366  1.86827  2.58110
C   2.34004  2.87639  1.94918
C   0.22459  3.65275  0.80304
C   0.80712  2.34739 -2.43519
C   3.06570  2.61434 -1.31536
H  -2.31664 -2.98782 -2.22738
H  -2.33717 -1.49043 -3.18696
H  -3.86345 -2.18529 -2.58853
H  -4.09359  1.30425 -2.31128
H  -2.55450  0.81932 -3.06657
H  -2.60186  2.20552 -1.94384
H  -2.89361  2.80791  0.29331
H  -2.93565  2.19663  1.96759
H  -4.38543  2.10059  0.94302
H  -3.80532 -1.11525  2.98804
H  -2.73215  0.28734  3.20697
H  -2.04834 -1.35325  3.08684
H  -3.48464 -3.48408  1.02152
H  -1.81367 -3.22499  1.55635
H  -2.14730 -3.75779 -0.11964
H   1.43590 -0.74170  1.97533
H  -0.04359 -0.66204 -1.50052
H   3.82312  2.17462 -0.64575
H   2.74085  3.57804 -0.89381
H   3.56203  2.83519 -2.27797
H   2.76063  0.76146 -3.35063
H   1.65587 -0.34251 -2.49482
H   3.29662 -0.00702 -1.83983
H   0.38393  3.23654 -1.94290
H  -0.01874  1.65535 -2.66690
H   1.25829  2.67867 -3.38857
H   0.02578  4.38978  1.60263
H  -0.74372  3.36664  0.36163
H   0.81893  4.16636  0.03058
H   0.69154  1.07750  3.11755
H  -0.81709  1.44867  2.24416
H  -0.06438  2.68138  3.30050
H   2.89721  2.03450  2.38838
H   2.17349  3.61689  2.75345
H   2.97511  3.35715  1.19267
H   4.72722 -3.24620 -0.53340
H   5.08582 -1.58541  0.05035
H   4.27799 -1.82856 -1.53129
H   3.42926 -4.14863  1.62172
H   2.07064 -3.28332  2.42754
H   3.72982 -2.62855  2.53642
H   1.46336 -2.71220 -1.65765
H   0.61584 -3.24843 -0.18255
H   2.02066 -4.19613 -0.79988
H   3.15822  0.09309  1.41758
```

9$_{tBu}$
SCF Energy = -975.905470765
Enthalpy 0K = -975.306487
Enthalpy 298K = -975.267868
Free Energy 298K = -975.372129
SCF(DCM) = -975.952912616
SCF(BP86-D3) = -0.11336630
Lowest Frequencies = 20.9915
36.2900 cm-1

```
Rh  0.88516  0.27935  0.17759
C   2.75705 -0.38723  1.08340
C   2.77000 -0.96907 -0.26132
C   2.77761  0.09799 -1.21687
C   2.79251  1.35491 -0.47212
C   2.82934  1.06397  0.93737
C   2.95021 -1.14912  2.36609
H   2.45465 -2.13198  2.33773
H   2.55198 -0.59502  3.23017
H   4.02666 -1.32569  2.55114
C   2.93606 -2.43502 -0.54744
H   2.57927 -2.71199 -1.55105
H   2.41122 -3.05924  0.19208
H   4.00861 -2.70183 -0.49665
C   2.92686 -0.01921 -2.70889
H   3.98655  0.10064 -3.00312
H   2.35160  0.75445 -3.24274
H   2.59513 -1.00029 -3.08157
C   2.90631  2.71885 -1.09429
H   2.54386  3.50892 -0.41865
H   2.34658  2.78504 -2.04064
H   3.96469  2.94263 -1.32419
C   3.04315  2.05394  2.05048
H   4.12457  2.19512  2.23366
H   2.58833  1.71144  2.99247
H   2.61807  3.04106  1.81045
H   0.20823  0.65269  1.54883
P  -1.09162 -0.93805 -0.02673
C  -1.32551 -2.26086 -1.43407
C  -2.77009 -2.23072 -1.98439
H  -3.03808 -1.22782 -2.35644
H  -3.52299 -2.54318 -1.24680
```

H -2.83963 -2.92917 -2.83900
C -0.96089 -3.67048 -0.91958
H -1.01797 -4.38602 -1.75996
H -1.64577 -4.03505 -0.13859
H   0.06732 -3.70611 -0.52329
C -0.36262 -1.89334 -2.58225
H   0.68314 -1.86200 -2.24321
H -0.60520 -0.91981 -3.03615
H -0.44754 -2.66194 -3.37192
C -1.99983 -1.47609 1.61041
C -2.28334 -0.23225 2.47221
H -3.08644 0.38776 2.03958
H -1.38242 0.38826 2.61379
H -2.62839 -0.55943 3.46979
C -1.02523 -2.39868 2.37969
H -0.08311 -1.87288 2.60547
H -0.78361 -3.31750 1.82277
H -1.48925 -2.70182 3.33617
C -3.34041 -2.19746 1.35329
H -3.81231 -2.42165 2.32737
H -3.21904 -3.15552 0.82676
H -4.04646 -1.57315 0.78049
B -1.26242 0.84389 -0.81051
H -0.12085 1.49164 -0.59375
H -1.39798 0.78770 -2.01294
P -2.46308 2.31576 -0.32234
C -4.26005 1.88292 -0.39751
H -4.87601 2.78869 -0.28433
H -4.51038 1.16857 0.40096
H -4.47151 1.41608 -1.37198
C -2.24782 3.63706 -1.60125
H -2.47496 3.21857 -2.59348
H -1.20178 3.98045 -1.59294
H -2.91478 4.48904 -1.39620
C -2.17507 3.18562 1.28266
H -2.77142 4.11070 1.31943
H -1.10440 3.43138 1.35690
H -2.44698 2.53804 2.12711

10_{Ph}
SCF Energy = -1163.99812594
Enthalpy 0K = -1163.414270
Enthalpy 298K = -1163.374498
Free Energy 298K = -1163.487011
SCF(DCM) = -1164.03218883
SCF(BP86-D3) = -0.10800270
Lowest Frequencies = 10.8873 14.8529 cm-1
C   3.36786 -0.39084 1.38618
C   2.21912 -0.07593 2.16802
C   1.81737 1.29938 1.83518
C   2.74689 1.83175 0.88358
C   3.67545 0.76560 0.53064
Rh 1.68847 0.09194 -0.10561
C   1.46422 1.27420 -1.85679
C   1.59787 -0.91866 3.24493
C   0.71821 2.05935 2.52014
C   2.81546 3.24769 0.39033
C   4.91872 0.94291 -0.29503
C   4.22960 -1.61529 1.50885
B -0.72379 -0.69972 0.36927
P -2.43740 -0.32294 -0.52882
C -3.83951 -1.30241 0.15508
P   1.93541 -1.71018 -1.55471
C   0.57046 -1.98140 -2.79414
C -2.95807 1.43594 -0.60317
C   3.43812 -1.67296 -2.65431
C   2.03899 -3.39530 -0.76618
H   1.83796 3.75157 0.44831
H   3.16450 3.30296 -0.65286
H   3.52484 3.82898 1.00753
H   5.71041 1.43353 0.30188
H   4.73661 1.57632 -1.17796
H   5.32298 -0.01981 -0.64404
H   4.63945 -1.93394 0.53666
H   3.68271 -2.46242 1.95044
H   5.09293 -1.40243 2.16648
H   1.94600 -0.59278 4.24277
H   1.86017 -1.98220 3.13288
H   0.49882 -0.84187 3.23692
H   1.09170 2.49853 3.46406
H -0.12844 1.40294 2.77294
H   0.33959 2.88278 1.89518
H -0.88237 -0.31806 1.50591
H -2.40932 -0.65514 -1.92297
H   0.79772 -2.86330 -3.41483
H   0.46563 -1.10099 -3.44493
H -0.37354 -2.15276 -2.25629
H   3.44040 -2.53752 -3.33806
H   4.35300 -1.69873 -2.04305
H   3.43227 -0.74192 -3.24170
H   1.12472 -3.56853 -0.17827
H   2.90763 -3.44727 -0.09462
H   2.13305 -4.17454 -1.54044
H   2.45690 1.45131 -2.30392
H   0.80551 0.83671 -2.62384
H   1.03079 2.23859 -1.54611
H -0.03740 0.11968 -0.37061
H -0.55311 -1.89170 0.24380
C -3.10381 2.08735 -1.84569
C -3.48264 3.43871 -1.88613
C -3.72061 4.13819 -0.69239
C -3.57846 3.48922 0.54668
C -3.19531 2.14128 0.59675
H -2.92704 1.54384 -2.78015
H -3.59794 3.94063 -2.85167
H -4.02215 5.18965 -0.72633
H -3.77112 4.03322 1.47658
H -3.09154 1.63764 1.56361
C -5.16836 -0.97423 -0.19057
C -6.22589 -1.74351 0.31425
C -5.96397 -2.83153 1.16510
C -4.64284 -3.15216 1.51493
C -3.57614 -2.38983 1.01291
H -5.37589 -0.11919 -0.84217
H -7.25619 -1.49101 0.04548
H -6.79317 -3.42764 1.55868
H -4.43940 -3.99537 2.18216
H -2.54621 -2.63681 1.28680

TS(10-11'')1_{Ph}
SCF Energy = -1163.97193239

Enthalpy 0K = -1163.390923
Enthalpy 298K = -1163.351213
Free Energy 298K = -1163.462730
SCF(DCM) = -1164.01782790
SCF(BP86-D3) = -0.11242551
Lowest Frequencies = -896.3359
15.4424 cm-1

```
C    3.32326 -0.41338  1.24337
C    2.19739 -0.09682  2.09847
C    1.81491  1.27228  1.82495
C    2.73733  1.80387  0.83455
C    3.67295  0.77410  0.47232
Rh   1.54329  0.06024 -0.09506
C    0.81168  1.39582 -1.74773
C    1.63452 -0.96967  3.18328
C    0.76931  2.06003  2.56285
C    2.77103  3.22346  0.34509
C    4.89434  0.95440 -0.38733
C    4.16546 -1.65501  1.33794
B   -0.50879 -0.75067  0.43273
P   -2.20830 -0.32758 -0.48322
C   -3.63064 -1.28206  0.20919
P    1.75663 -1.72271 -1.56478
C    0.40706 -1.96375 -2.82587
C   -2.73852  1.43399 -0.57365
C    3.26752 -1.67022 -2.65963
C    1.86518 -3.42406 -0.81725
H    1.77970  3.70119  0.39061
H    3.14117  3.29588 -0.69032
H    3.45034  3.82443  0.97730
H    5.71676  1.41358  0.19280
H    4.70069  1.61373 -1.24972
H    5.27102 -0.00471 -0.77592
H    4.60636 -1.93914  0.36890
H    3.59530 -2.51212  1.72721
H    5.00656 -1.47951  2.03433
H    2.11254 -0.73329  4.15196
H    1.80434 -2.03873  2.98114
H    0.54963 -0.82153  3.30108
H    1.21142  2.57089  3.43842
H   -0.03692  1.40694  2.93149
H    0.31370  2.83237  1.92312
H   -0.74319 -0.33073  1.54874
H   -2.24975 -0.66734 -1.87662
H    0.67336 -2.78872 -3.50640
H    0.26845 -1.04510 -3.41523
H   -0.53159 -2.21500 -2.31079
H    3.29808 -2.55084 -3.32237
H    4.17687 -1.65616 -2.04020
H    3.24805 -0.75538 -3.27242
H    0.96708 -3.60343 -0.20741
H    2.75334 -3.49296 -0.17313
H    1.93396 -4.18595 -1.61091
H    1.79765  1.49077 -2.22408
H    0.09635  1.05762 -2.51443
H    0.48834  2.37348 -1.36370
H    0.14853  0.55078 -0.75041
H   -0.49611 -1.96628  0.36386
C   -3.05256  2.02948 -1.81364
C   -3.46142  3.37182 -1.86420
C   -3.56283  4.12076 -0.68089
C   -3.25300  3.52935  0.55632
C   -2.83872  2.19053  0.61427
H   -2.98470  1.44758 -2.73971
H   -3.70721  3.82830 -2.82804
H   -3.88859  5.16475 -0.72126
H   -3.34004  4.11086  1.47947
H   -2.60524  1.73137  1.58056
C   -4.92178 -1.10780 -0.33437
C   -5.99879 -1.83929  0.18457
C   -5.79462 -2.73699  1.24729
C   -4.51215 -2.90466  1.79201
C   -3.42552 -2.18047  1.27502
H   -5.08878 -0.39938 -1.15289
H   -6.99899 -1.70668 -0.23930
H   -6.63876 -3.30469  1.65105
H   -4.35330 -3.60113  2.62114
H   -2.42388 -2.31318  1.69430
```

INT(10-11'')$_{Ph}$
SCF Energy = -1163.98807808
Enthalpy 0K = -1163.404256
Enthalpy 298K = -1163.363581
Free Energy 298K = -1163.476475
SCF(DCM) = -1164.03425283
SCF(BP86-D3) = -0.11383316
Lowest Frequencies = 14.8129
22.2518 cm-1

```
C    3.47946  0.40039  0.94429
C    2.32605  0.15203  1.85021
C    1.40168  1.27713  1.70984
C    1.86942  2.05835  0.61013
C    3.18476  1.54430  0.17076
Rh   1.47174 -0.08342 -0.10199
C    0.90356  0.79643 -2.72438
C    2.31736 -0.83067  2.98433
C    0.28094  1.62645  2.64285
C    1.23292  3.31576  0.09294
C    4.05675  2.19937 -0.86205
C    4.77016 -0.36535  0.98016
B   -0.43835 -1.01348  0.48669
P   -2.04076 -0.36861 -0.45689
C   -3.58934 -1.25044  0.04215
P    1.92187 -2.20827 -0.92227
C    0.75867 -2.88492 -2.21375
C   -2.47985  1.42591 -0.47138
C    3.55253 -2.35929 -1.82533
C    2.03127 -3.59902  0.30841
H    0.14162  3.31070  0.23717
H    1.43961  3.47110 -0.97794
H    1.63946  4.19397  0.62949
H    4.58538  3.07133 -0.43353
H    3.47083  2.57285 -1.71855
H    4.82188  1.50941 -1.25094
H    5.34832 -0.24909  0.05031
H    4.61190 -1.44056  1.16246
H    5.40289  0.00652  1.80796
H    2.76657 -0.37350  3.88668
H    2.90129 -1.73390  2.74980
H    1.29222 -1.14070  3.23765
H    0.66266  2.27631  3.45281
H   -0.15728  0.73197  3.10950
```

```
H  -0.52533  2.17107  2.12787
H  -0.66769 -0.77252  1.65734
H  -1.98980 -0.61284 -1.86981
H   1.08865 -3.88939 -2.52510
H   0.76127 -2.22170 -3.09238
H  -0.25857 -2.94503 -1.80207
H   3.69805 -3.39103 -2.18606
H   4.38317 -2.09251 -1.15611
H   3.55930 -1.67322 -2.68678
H   1.08261 -3.66751  0.86021
H   2.84755 -3.39717  1.01935
H   2.23328 -4.55089 -0.20921
H   1.98209  0.61579 -2.80904
H   0.36385  0.21078 -3.48251
H   0.69111  1.86819 -2.84190
H   0.46997  0.50334 -1.71846
H  -0.56773 -2.21019  0.26915
C  -2.39570  2.18051 -1.65996
C  -2.72087  3.54728 -1.65352
C  -3.13720  4.16556 -0.46378
C  -3.23533  3.41456  0.72080
C  -2.90885  2.04961  0.72075
H  -2.09222  1.70068 -2.59689
H  -2.66213  4.12384 -2.58217
H  -3.39895  5.22818 -0.46126
H  -3.57769  3.89014  1.64529
H  -3.00865  1.46624  1.64229
C  -4.78803 -1.05175 -0.67660
C  -5.95439 -1.72209 -0.28297
C  -5.93146 -2.58376  0.82813
C  -4.74091 -2.77815  1.54531
C  -3.56608 -2.11513  1.15411
H  -4.81343 -0.37349 -1.53655
H  -6.88273 -1.57102 -0.84261
H  -6.84499 -3.10360  1.13348
H  -4.72314 -3.44824  2.41051
H  -2.63335 -2.26836  1.70528
```

TS(10-11’’)2_{Ph}
SCF Energy = -1163.98790744
Enthalpy 0K = -1163.404489
Enthalpy 298K = -1163.364391
Free Energy 298K = -1163.475840
SCF(DCM) = -1164.03409415
SCF(BP86-D3) = -0.11263080
Lowest Frequencies = -14.8238
14.4526 cm-1

```
C   3.49258  0.48083  0.86144
C   2.32320  0.45788  1.78263
C   1.44428  1.56781  1.41623
C   1.95736  2.10698  0.19686
C   3.25523  1.47139 -0.11570
Rh  1.47572 -0.12017 -0.09405
P   1.87532 -2.35040 -0.59876
C   0.71038 -3.14908 -1.81604
C   2.26071 -0.29216  3.07985
C   0.32369  2.11968  2.24605
C   1.38448  3.26735 -0.56269
C   4.16760  1.88668 -1.23454
C   4.74782 -0.31716  1.06512
B  -0.44199 -0.90250  0.62305
P  -2.01077 -0.29101 -0.39392
C  -3.54750 -1.24762 -0.01547
C  -2.50886  1.48682 -0.33875
C   3.51685 -2.67295 -1.43294
C   1.91393 -3.55492  0.81608
C   0.89692  0.39377 -3.02892
H   0.29311  3.34209 -0.44015
H   1.61031  3.20323 -1.63911
H   1.82926  4.21206 -0.19615
H   4.71407  2.81195 -0.97231
H   3.61296  2.09603 -2.16425
H   4.91861  1.11265 -1.45700
H   5.33136 -0.42023  0.13714
H   4.54384 -1.32454  1.46310
H   5.39626  0.18943  1.80469
H   2.73696  0.30269  3.88301
H   2.79175 -1.25520  3.02752
H   1.21933 -0.48873  3.37643
H   0.72579  2.83534  2.98786
H  -0.20366  1.32668  2.79728
H  -0.41791  2.65221  1.63203
H  -0.64973 -0.50762  1.75724
H  -1.89836 -0.47768 -1.81112
H   1.00910 -4.19563 -1.98989
H   0.75394 -2.60144 -2.77002
H  -0.31546 -3.12047 -1.42331
H   3.62255 -3.74321 -1.67674
H   4.34326 -2.37337 -0.77227
H   3.57566 -2.08425 -2.36144
H   0.95734 -3.50539  1.35551
H   2.72897 -3.28159  1.50367
H   2.08449 -4.57789  0.44327
H   1.93684  0.07999 -3.18363
H   0.21525 -0.24811 -3.60499
H   0.77441  1.44520 -3.32682
H   0.60666  0.32731 -1.94426
H  -0.61894 -2.10900  0.55129
C  -4.63582 -1.23507 -0.91413
C  -5.80216 -1.94920 -0.60579
C  -5.88958 -2.67012  0.59815
C  -4.80833 -2.68161  1.49313
C  -3.63333 -1.97517  1.18844
H  -4.57610 -0.66911 -1.85033
H  -6.64363 -1.94304 -1.30561
H  -6.80213 -3.22577  0.83574
H  -4.87510 -3.24548  2.42882
H  -2.78250 -1.99520  1.87676
C  -2.32346  2.32999 -1.45392
C  -2.69773  3.68268 -1.38337
C  -3.26187  4.19640 -0.20457
C  -3.45786  3.35579  0.90508
C  -3.08298  2.00536  0.84169
H  -1.90278  1.93097 -2.38345
H  -2.56098  4.33014 -2.25535
H  -3.56073  5.24796 -0.15383
H  -3.91110  3.75034  1.81987
H  -3.25193  1.35214  1.70433
```

11''$_{Ph}$
SCF Energy = -1123.47160186
Enthalpy 0K = -1122.934570

Enthalpy 298K = -1122.897388
Free Energy 298K = -1123.002494
SCF(DCM) = -1123.51856541
SCF(BP86-D3) = -0.10252866
Lowest Frequencies = 20.1660
25.2345 cm-1

C	1.82311	0.87868	2.04606
C	2.81628	1.38146	1.06287
C	3.59221	0.29860	0.59313
C	3.01958	-0.92201	1.18712
C	2.01274	-0.52505	2.18948
Rh	1.32213	-0.17153	0.09421
P	1.35846	-1.22369	-1.98738
C	0.53185	-2.87867	-2.20243
C	3.00812	2.83032	0.72456
C	4.82446	0.37687	-0.26371
C	3.61165	-2.29585	1.09005
C	1.42713	-1.43080	3.23098
C	0.90813	1.74706	2.86035
B	-0.54321	-1.15050	0.61182
P	-2.04502	-0.43088	-0.41848
C	-3.72076	-0.86245	0.20570
C	-1.99201	1.40132	-0.66842
C	0.61135	-0.20094	-3.36495
C	3.04052	-1.57775	-2.71418
H	2.05002	3.37335	0.69108
H	3.52234	2.96330	-0.24005
H	3.62576	3.32314	1.49938
H	5.71400	0.57213	0.36417
H	4.76518	1.19139	-1.00385
H	5.01508	-0.56232	-0.80393
H	4.07615	-2.47829	0.10905
H	2.85846	-3.07688	1.27428
H	4.40570	-2.41250	1.85293
H	2.13649	-1.53140	4.07397
H	1.22930	-2.43803	2.83365
H	0.48163	-1.03736	3.63130
H	1.44309	2.16288	3.73471
H	0.04487	1.17622	3.23528
H	0.52343	2.59732	2.27475
H	-0.62033	-2.35278	0.44402
H	-0.83311	-0.80045	1.74396
H	-2.14884	-0.91975	-1.76541
H	0.62881	-0.76420	-4.31252
H	1.19911	0.72303	-3.48084
H	-0.42550	0.07891	-3.12587
H	2.92859	-1.99947	-3.72647
H	3.57641	-2.30567	-2.08623
H	3.62706	-0.64911	-2.77101
H	-0.53554	-2.80331	-1.95238
H	0.99085	-3.60153	-1.51086
H	0.64803	-3.23277	-3.23968
C	-3.16227	2.17979	-0.77756
C	-3.06714	3.56323	-0.99828
C	-1.80983	4.17695	-1.11893
C	-0.63792	3.40825	-1.00989
C	-0.73723	2.02904	-0.77721
H	-4.14450	1.70554	-0.68555
H	-3.97944	4.16236	-1.07701
H	-1.73967	5.25498	-1.29403
H	0.34421	3.88199	-1.10551
H	0.20914	1.43803	-0.68757
C	-4.72874	-1.28928	-0.68580
C	-5.99912	-1.62670	-0.19379
C	-6.26606	-1.53965	1.18223
C	-5.26218	-1.11925	2.07125
C	-3.98851	-0.78443	1.58955
H	-4.52447	-1.36160	-1.75974
H	-6.77825	-1.95966	-0.88637
H	-7.25692	-1.80516	1.56358
H	-5.46911	-1.05891	3.14417
H	-3.20142	-0.47579	2.28462

TS(11''-11)$_{Ph}$
SCF Energy = -1123.46620337
Enthalpy 0K = -1122.929634
Enthalpy 298K = -1122.892661
Free Energy 298K = -1122.998479
SCF(DCM) = -1123.51402288
SCF(BP86-D3) = -0.09528986
Lowest Frequencies = -136.7872
21.2356 cm-1

C	2.78779	-0.92617	1.78084
C	3.78247	-0.15278	1.00480
C	3.81841	-0.69936	-0.29354
C	2.82518	-1.80617	-0.35187
C	2.30016	-2.02013	0.98650
Rh	1.59928	-0.08645	0.09115
P	1.27321	1.68662	-1.39827
C	0.60386	1.30270	-3.09608
C	4.61611	0.96493	1.56175
C	4.75786	-0.35835	-1.41150
C	2.64039	-2.71915	-1.52500
C	1.48076	-3.18155	1.46544
C	2.46919	-0.67267	3.22272
B	-0.41240	-0.79288	0.22294
P	-2.11169	-0.05881	-0.43465
C	-3.45127	-1.32687	-0.48147
C	-2.80653	1.41528	0.43790
C	0.19240	3.08985	-0.81359
C	2.82959	2.62407	-1.83223
H	4.02404	1.63134	2.21060
H	5.06414	1.57756	0.76409
H	5.44423	0.56708	2.17779
H	5.57325	-1.10494	-1.45371
H	5.22508	0.62868	-1.27626
H	4.25947	-0.38038	-2.39434
H	2.71544	-2.17560	-2.47987
H	1.66521	-3.22686	-1.49240
H	3.42914	-3.49669	-1.52482
H	2.12858	-3.92047	1.97126
H	0.97304	-3.69067	0.63300
H	0.70373	-2.86657	2.18016
H	3.28094	-1.06898	3.86242
H	1.53168	-1.16152	3.52608
H	2.38842	0.40605	3.43564
H	-0.48571	-1.95185	-0.13667
H	-0.50547	-0.67321	1.44744
H	-2.16038	0.38479	-1.79930
H	0.15662	3.88076	-1.58116
H	0.61670	3.50545	0.11389
H	-0.82528	2.73443	-0.59810

H   2.58459 3.47874 -2.48434
H   3.53757 1.96597 -2.35657
H   3.30237 2.99586 -0.91031
H -0.38527 0.82928 -3.01467
H   1.28436 0.59576 -3.59529
H   0.52607 2.22261 -3.69888
C -3.72687 2.26289 -0.21743
C -4.27894 3.35823 0.46233
C -3.92273 3.61025 1.79850
C -3.01175 2.76714 2.45464
C -2.45053 1.67180 1.77857
H -4.01598 2.06753 -1.25597
H -4.98983 4.01347 -0.05057
H -4.35754 4.46384 2.32777
H -2.73641 2.96064 3.49620
H -1.73838 1.01676 2.29009
C -4.22986 -1.51066 -1.64223
C -5.24397 -2.48130 -1.65740
C -5.48529 -3.26430 -0.51735
C -4.70825 -3.08330 0.63958
C -3.68861 -2.12096 0.66016
H -4.04630 -0.90290 -2.53516
H -5.84462 -2.62415 -2.56105
H -6.27806 -4.01866 -0.53054
H -4.89432 -3.69556 1.52746
H -3.07678 -1.99083 1.55891

11_{Ph}
SCF Energy = -1123.48454515
Enthalpy 0K = -1122.946896
Enthalpy 298K = -1122.910014
Free Energy 298K = -1123.015141
SCF(DCM) = -1123.53030499
SCF(BP86-D3) = -0.09634762
Lowest Frequencies = 15.9414
23.1703 cm-1
C   3.28559 0.89952 -0.64205
C   2.40434 0.75910 -1.80194
C   2.34007 -0.63952 -2.11569
C   3.26208 -1.35558 -1.21536
C   3.88292 -0.40902 -0.35663
Rh 1.53923 -0.30711 -0.03828
P   1.28555 0.11380 2.22629
C   0.38799 -1.21886 3.18077
C   1.79356 1.88554 -2.58664
C   1.61701 -1.25946 -3.27759
C   3.56291 -2.82494 -1.29231
C   5.02339 -0.67988 0.58421
C   3.75533 2.21375 -0.08228
B -0.43143 -0.35677 -0.49951
P -2.13785 -0.06251 0.42100
C -2.82716 1.57740 -0.05087
C -3.41115 -1.35019 0.11207
C   2.84977 0.22858 3.23838
C   0.43606 1.68938 2.76395
H   2.66194 -3.41657 -1.51905
H   3.99309 -3.20399 -0.35243
H   4.29535 -3.02431 -2.09724
H   5.98419 -0.63779 0.03755
H   4.95412 -1.67981 1.04223
H   5.08379 0.06326 1.39324
H   4.12275 2.11699 0.95124
H   2.95559 2.97083 -0.09351
H   4.59359 2.60780 -0.68775
H   2.49895 2.23147 -3.36586
H   1.56208 2.75136 -1.94680
H   0.86673 1.57552 -3.09276
H   2.27194 -1.28669 -4.16797
H   0.71428 -0.68877 -3.54633
H   1.30968 -2.29522 -3.06199
H -0.76071 -0.37370 -1.66898
H -2.11176 -0.05490 1.85355
H   0.25775 -0.92880 4.23656
H   0.98417 -2.14332 3.12617
H -0.59454 -1.42340 2.73166
H   2.59888 0.37395 4.30221
H   3.45751 1.08078 2.89826
H   3.43503 -0.69638 3.12771
H -0.55996 1.77233 2.30362
H   1.04082 2.54265 2.41776
H   0.34186 1.73516 3.86172
H   0.16894 -1.46683 -0.03368
C -4.20951 1.83737 0.05378
C -4.70135 3.10576 -0.28734
C -3.82264 4.10720 -0.73526
C -2.44770 3.84380 -0.84688
C -1.94385 2.57862 -0.50655
H -4.89653 1.05386 0.38921
H -5.77326 3.31063 -0.20716
H -4.21275 5.09401 -1.00328
H -1.76680 4.62259 -1.20424
H -0.87343 2.35965 -0.59645
C -4.07477 -1.96465 1.19553
C -5.03119 -2.96257 0.95164
C -5.32589 -3.34682 -0.36630
C -4.66407 -2.73583 -1.44555
C -3.70410 -1.74099 -1.21319
H -3.85168 -1.66531 2.22553
H -5.54625 -3.43677 1.79262
H -6.07258 -4.12451 -0.55378
H -4.89451 -3.03649 -2.47215
H -3.18762 -1.27092 -2.05617

$TS(11\text{-}11')_{Ph}$
SCF Energy = -1123.47781893
Enthalpy 0K = -1122.942405
Enthalpy 298K = -1122.905537
Free Energy 298K = -1123.010860
SCF(DCM) = -1123.52370313
SCF(BP86-D3) = -0.09535146
Lowest Frequencies = -361.2261
16.6121 cm-1
C -3.39888 -0.55034 -1.07498
C -2.46649 -0.01260 -2.04489
C -2.23490 1.37145 -1.70637
C -3.09448 1.70853 -0.56565
C -3.82840 0.54130 -0.20083
Rh -1.49484 0.12767 0.01998
P -1.32870 -1.22251 1.89222
C -0.65262 -0.40046 3.42606
C -1.93627 -0.72840 -3.25636
C -1.44511 2.35778 -2.52202

C -3.26736 3.08657 0.01101
C -4.95120 0.48495 0.79894
C -4.03452 -1.91208 -1.15458
B 0.39123 0.07954 -0.51255
P 2.11597 -0.09341 0.44517
C 3.16301 -1.41989 -0.27547
C 3.06115 1.48006 0.45508
C -2.91776 -1.92424 2.57412
C -0.30657 -2.77964 1.75898
H -2.33025 3.66335 -0.02012
H -3.60986 3.05164 1.05692
H -4.02278 3.65187 -0.56649
H -5.89676 0.81513 0.32934
H -4.77134 1.14572 1.66239
H -5.12208 -0.53474 1.17631
H -4.45791 -2.22859 -0.18894
H -3.31880 -2.68194 -1.48389
H -4.86766 -1.90397 -1.88242
H -2.58962 -0.54089 -4.12903
H -1.89801 -1.81786 -3.10130
H -0.92417 -0.38603 -3.52375
H -2.08467 2.80836 -3.30344
H -0.59368 1.87709 -3.02908
H -1.05103 3.17813 -1.90199
H 0.74333 0.37224 -1.64081
H 2.03418 -0.38352 1.84578
H -0.59102 -1.11340 4.26478
H -1.32266 0.43061 3.69597
H 0.34467 0.01948 3.22723
H -2.70198 -2.52876 3.47057
H -3.40339 -2.56344 1.82166
H -3.60000 -1.10622 2.84872
H 0.73605 -2.53444 1.50592
H -0.71896 -3.39478 0.94403
H -0.32807 -3.35207 2.70126
H -0.50016 1.14402 0.77304
C 4.54609 -1.47069 0.00126
C 5.32124 -2.50573 -0.54024
C 4.72433 -3.48180 -1.35734
C 3.34959 -3.42632 -1.63813
C 2.56338 -2.39586 -1.09953
H 5.01487 -0.70345 0.62594
H 6.39355 -2.54826 -0.32666
H 5.33502 -4.28548 -1.78020
H 2.88827 -4.18244 -2.28068
H 1.49140 -2.34820 -1.32195
C 3.28526 2.16643 1.66748
C 3.96176 3.39599 1.65464
C 4.41229 3.93894 0.44054
C 4.18894 3.25454 -0.76690
C 3.51037 2.02777 -0.76704
H 2.94193 1.74386 2.61788
H 4.14060 3.92552 2.59534
H 4.94176 4.89658 0.43474
H 4.54436 3.67662 -1.71180
H 3.34024 1.49660 -1.70917

$11'_{Ph}$
SCF Energy = -1123.47907794
Enthalpy 0K = -1122.941990
Enthalpy 298K = -1122.904877
Free Energy 298K = -1123.011773
SCF(DCM) = -1123.52485086
SCF(BP86-D3) = -0.09603834
Lowest Frequencies = 9.3909
21.8762 cm-1
C -3.49613 -1.23696 1.69994
C -3.29353 -1.24156 0.30145
C -3.99379 -2.15831 -0.51331
C -4.89312 -3.06313 0.07075
C -5.09703 -3.05502 1.46054
C -4.40156 -2.14255 2.27197
P -2.09727 -0.07328 -0.44872
C -2.86915 1.59438 -0.60857
C -4.21020 1.81100 -0.23439
C -4.76389 3.09438 -0.36567
C -3.98778 4.15156 -0.86741
C -2.65009 3.93215 -1.23776
C -2.08167 2.65670 -1.10489
B -0.37698 0.03080 0.53048
Rh 1.48168 0.06412 -0.07656
P 1.31759 -1.69235 -1.56375
C 0.28787 -3.16844 -1.06649
C 3.33423 -0.44223 1.26617
C 2.36958 0.29591 2.04520
C 2.20128 1.59350 1.42333
C 3.12034 1.66672 0.28686
C 3.82826 0.42752 0.20244
C 3.91126 -1.78018 1.64440
C 1.76326 -0.13915 3.34994
C 1.43133 2.75155 1.99869
C 3.37932 2.89385 -0.54433
C 4.99509 0.14323 -0.70488
C 0.66341 -1.28317 -3.26144
C 2.92581 -2.52354 -2.00941
H 2.47454 3.51107 -0.65446
H 3.73850 2.63573 -1.55263
H 4.15328 3.52359 -0.06706
H 5.92802 0.54488 -0.26688
H 4.87505 0.61218 -1.69503
H 5.15240 -0.93586 -0.85456
H 4.35116 -2.30187 0.77958
H 3.15503 -2.44245 2.09460
H 4.72137 -1.65312 2.38677
H 2.37915 0.21632 4.19696
H 1.69671 -1.23585 3.42563
H 0.75038 0.27213 3.48788
H 2.06250 3.31358 2.71216
H 0.53547 2.41527 2.54344
H 1.10891 3.45672 1.21693
H -0.70671 0.26011 1.67909
H -2.00880 -0.56508 -1.79049
H 0.62269 -2.18543 -3.89353
H 1.33000 -0.53846 -3.72326
H -0.34214 -0.84230 -3.18931
H 2.74407 -3.33347 -2.73509
H 3.38788 -2.94611 -1.10472
H 3.61123 -1.78546 -2.45175
H -0.76052 -2.86727 -0.91412
H 0.67151 -3.56578 -0.11389
H 0.33209 -3.95540 -1.83798
H 0.82582 0.88769 -1.25374

H -4.81766 0.98769 0.15306
H -5.80567 3.26478 -0.07750
H -4.42544 5.14937 -0.96940
H -2.04542 4.75629 -1.62872
H -1.03596 2.48746 -1.38387
H -3.84381 -2.16332 -1.59834
H -5.43609 -3.77195 -0.56171
H -5.79997 -3.76135 1.91272
H -4.56112 -2.13701 3.35446
H -2.95261 -0.53146 2.33606

TS(11''-9')1_{Ph}
SCF Energy = -1123.47292465
Enthalpy 0K = -1122.934918
Enthalpy 298K = -1122.898617
Free Energy 298K = -1123.002166
SCF(DCM) = -1123.52131614
SCF(BP86-D3) = -0.10016299
Lowest Frequencies = -10.2344
18.4428 cm-1
C -1.83500 2.06184 -0.34159
C -1.94360 1.73955 1.04336
C -2.96166 0.68716 1.13900
C -3.61641 0.55175 -0.18887
C -2.89733 1.35131 -1.09579
Rh -1.38207 -0.14829 -0.01601
P -1.53396 -2.46339 -0.24187
C -0.03266 -3.33472 -0.92548
C -1.25695 2.41484 2.19351
C -3.48966 0.11857 2.42083
C -4.87446 -0.22476 -0.44956
C -3.16170 1.54018 -2.56114
C -0.92933 3.09245 -0.94562
B 0.34439 -0.55956 1.20518
P 1.75796 -0.12063 -0.10683
C 2.36540 1.62122 -0.10319
C 3.24798 -1.22020 -0.05221
C -1.90069 -3.41228 1.31566
C -2.85337 -3.07861 -1.41072
H -4.94783 -1.12292 0.18445
H -4.96227 -0.53482 -1.50248
H -5.75716 0.39995 -0.21635
H -3.63327 2.52283 -2.74999
H -3.83748 0.76739 -2.95814
H -2.23016 1.51373 -3.15104
H -0.60826 2.81251 -1.96209
H -0.03139 3.26012 -0.33308
H -1.46540 4.05713 -1.03196
H -1.86469 3.26922 2.54576
H -0.26649 2.80081 1.90697
H -1.11480 1.72900 3.04226
H -4.25181 0.79877 2.84904
H -2.68942 -0.00202 3.16647
H -3.97429 -0.85835 2.26893
H 0.48616 0.23214 2.11576
H 1.40335 -0.25940 -1.49358
H -0.22323 -4.41909 -0.97867
H 0.83766 -3.14915 -0.27963
H 0.17801 -2.96093 -1.94004
H -1.93649 -4.49441 1.10888
H -2.87189 -3.08902 1.72184
H -1.11544 -3.19490 2.05462
H -2.67646 -2.66049 -2.41385
H -3.84672 -2.75741 -1.06554
H -2.83067 -4.17965 -1.46664
H 0.58273 -1.70920 1.52644
C 2.55532 2.28230 1.12887
C 3.06462 3.59046 1.14856
C 3.38137 4.24285 -0.05490
C 3.19361 3.58594 -1.28258
C 2.68939 2.27643 -1.30995
H 2.30580 1.77491 2.06629
H 3.21593 4.09894 2.10587
H 3.77707 5.26303 -0.03619
H 3.44165 4.09201 -2.22079
H 2.54936 1.76897 -2.27077
C 4.04367 -1.41581 -1.20084
C 5.19465 -2.21311 -1.12250
C 5.55463 -2.81219 0.09705
C 4.76152 -2.61923 1.23967
C 3.60383 -1.82809 1.16883
H 3.77111 -0.94805 -2.15334
H 5.80999 -2.36745 -2.01434
H 6.45378 -3.43348 0.15459
H 5.04027 -3.08829 2.18837
H 2.97528 -1.68851 2.05441

INT(11''-9')$_{Ph}$
SCF Energy = -1123.50850409
Enthalpy 0K = -1122.969860
Enthalpy 298K = -1122.933265
Free Energy 298K = -1123.034725
SCF(DCM) = -1123.55480898
SCF(BP86-D3) = -0.11171788
Lowest Frequencies = 24.8318
34.1598 cm-1
C -1.43236 -2.24730 -0.33969
C -2.59032 -1.48030 -0.72143
C -3.10439 -0.83677 0.47872
C -2.24187 -1.17911 1.58748
C -1.19224 -2.04226 1.08333
Rh -0.97094 -0.01389 0.08037
P -1.64172 2.16788 -0.44188
C -0.37212 3.48981 -0.74235
C -3.27676 -1.53371 -2.05779
C -4.44322 -0.16428 0.59232
C -2.48684 -0.84645 3.03451
C -0.19731 -2.79610 1.91908
C -0.72620 -3.24728 -1.20407
B 0.15800 0.20928 -2.07665
P 1.29673 0.11583 -0.58946
C 2.38733 -1.34766 -0.34608
C 2.28557 1.56410 -0.02205
C -2.77711 2.34911 -1.90448
C -2.61201 2.94605 0.94521
H -2.98663 0.12660 3.15701
H -1.54862 -0.81764 3.60889
H -3.13559 -1.61373 3.49604
H -0.61387 -3.78020 2.20461
H 0.04436 -2.25791 2.84862
H 0.74339 -2.97747 1.37807
H 0.33353 -3.35863 -0.93073

H -0.78418 -2.98254 -2.27061
H -1.20586 -4.23659 -1.08058
H -3.93925 -2.41775 -2.11295
H -2.55250 -1.60805 -2.88330
H -3.90255 -0.64600 -2.23652
H -5.23384 -0.93642 0.63954
H -4.67730 0.47466 -0.27367
H -4.52973 0.43907 1.50866
H -0.06872 -0.81318 -2.66564
H -0.35199 0.78824 1.28663
H -0.89814 4.44069 -0.92745
H 0.25191 3.24104 -1.61107
H 0.27259 3.58996 0.14276
H -3.09426 3.39994 -2.00613
H -3.66631 1.71606 -1.76892
H -2.24664 2.02979 -2.81328
H -1.97005 3.00515 1.83738
H -3.49638 2.33870 1.18212
H -2.93598 3.96008 0.65890
H -0.00566 1.27112 -2.61284
C 3.05528 -1.55290 0.88190
C 3.93892 -2.63222 1.02820
C 4.16322 -3.51222 -0.04494
C 3.50787 -3.30739 -1.26995
C 2.62343 -2.22768 -1.42600
H 2.90026 -0.86061 1.71553
H 4.45768 -2.78289 1.98010
H 4.85773 -4.35000 0.07082
H 3.69453 -3.97984 -2.11314
H 2.13172 -2.05338 -2.38768
C 3.14732 2.18339 -0.95354
C 3.96021 3.25484 -0.55114
C 3.92533 3.70726 0.77817
C 3.07547 3.08769 1.70991
C 2.25548 2.01896 1.31290
H 3.17971 1.83100 -1.98968
H 4.62136 3.73575 -1.27876
H 4.56102 4.54221 1.08878
H 3.05009 3.43601 2.74737
H 1.58555 1.54415 2.03845

TS(11’’-6)2_{Ph}
SCF Energy = -1123.48721559
Enthalpy 0K = -1122.949114
Enthalpy 298K = -1122.913062
Free Energy 298K = -1123.013258
SCF(DCM) = -1123.53355311
SCF(BP86-D3) = -0.10874758
Lowest Frequencies = -24.9793 22.0761 cm-1
C -2.31699 1.39393 1.45155
C -2.58986 -0.02546 1.69857
C -3.14023 -0.57438 0.49836
C -3.19733 0.48539 -0.51489
C -2.67445 1.69340 0.08807
Rh -1.01509 0.13705 -0.13284
P -0.51160 -1.61230 -1.57775
C 1.03534 -1.42173 -2.59075
C -2.58531 -0.70367 3.04122
C -3.77015 -1.93511 0.38530
C -3.97484 0.44684 -1.80252
C -2.70364 3.06841 -0.52318
C -2.12933 2.45057 2.50709
B 0.54235 0.73681 2.28403
P 1.23700 0.31080 0.58999
C 2.27134 1.68641 -0.09434
C 2.37132 -1.15723 0.70990
C -0.34813 -3.32076 -0.85715
C -1.75231 -1.90023 -2.93874
H -4.11546 -0.57619 -2.17918
H -3.49115 1.04154 -2.59346
H -4.98210 0.87406 -1.64047
H -3.63971 3.58948 -0.24779
H -2.65812 3.02601 -1.62185
H -1.86380 3.68803 -0.17343
H -1.47752 3.27081 2.17042
H -1.72320 2.04936 3.44574
H -3.12030 2.89138 2.73056
H -3.58297 -0.61487 3.51186
H -1.85369 -0.25713 3.72985
H -2.35972 -1.77878 2.95925
H -4.82004 -1.89059 0.73058
H -3.25486 -2.68075 1.01120
H -3.79234 -2.30710 -0.65025
H 0.39648 1.89267 2.54462
H -0.44900 0.96911 -1.33087
H 1.12551 -2.27460 -3.28292
H 1.92113 -1.39212 -1.94084
H 0.97406 -0.48688 -3.16843
H -0.18537 -4.05356 -1.66498
H -1.26931 -3.57890 -0.31307
H 0.50239 -3.34734 -0.15996
H -1.93557 -0.96515 -3.48809
H -2.70185 -2.27124 -2.52670
H -1.35119 -2.65762 -3.63209
H 0.30904 -0.14566 3.05991
C 2.02039 2.27827 -1.34726
C 2.82989 3.32986 -1.80812
C 3.89354 3.79693 -1.02071
C 4.14966 3.21137 0.23084
C 3.34553 2.16016 0.69445
H 1.18113 1.92724 -1.95713
H 2.62538 3.78476 -2.78245
H 4.52098 4.61879 -1.37931
H 4.97395 3.57642 0.85136
H 3.55333 1.70837 1.67018
C 2.02443 -2.23614 1.55344
C 2.89134 -3.33086 1.69017
C 4.10752 -3.36339 0.98537
C 4.45611 -2.29350 0.14711
C 3.59450 -1.19179 0.00782
H 1.08527 -2.21060 2.11569
H 2.62028 -4.15564 2.35700
H 4.78353 -4.21647 1.09771
H 5.40501 -2.30767 -0.39823
H 3.88609 -0.35152 -0.62994

INT(11’’-6)2_{Ph}
SCF Energy = -1123.49039441
Enthalpy 0K = -1122.951797
Enthalpy 298K = -1122.915172
Free Energy 298K = -1123.034725

SCF(DCM) = -1123.53758248
SCF(BP86-D3) = -0.11169754
Lowest Frequencies = 14.7821
30.1235 cm-1
C -2.44621 -0.46129 -1.84237
C -2.51807 0.90513 -1.30811
C -3.00839 0.82792 0.02792
C -3.21033 -0.58835 0.36805
C -2.84671 -1.37013 -0.78770
Rh -0.98844 -0.36590 0.05052
P -0.27831 0.34243 2.15208
C 1.27933 -0.40890 2.83044
C -2.33905 2.15680 -2.11910
C -3.45211 1.99799 0.86156
C -3.98665 -1.12108 1.54141
C -3.05703 -2.85033 -0.95716
C -2.50181 -0.82703 -3.30375
B 0.27669 -0.53739 -2.30011
P 1.24213 -0.15948 -0.73873
C 2.50756 -1.45537 -0.35678
C 2.06684 1.49241 -0.62432
C 0.02380 2.17008 2.31658
C -1.43975 -0.00508 3.56657
H -3.99966 -0.42412 2.39088
H -3.59457 -2.08801 1.89356
H -5.03878 -1.28502 1.24224
H -4.04974 -3.04686 -1.40393
H -3.01542 -3.38017 0.00648
H -2.29957 -3.29378 -1.62150
H -2.04399 -1.80481 -3.51213
H -2.02394 -0.07846 -3.94989
H -3.56848 -0.89282 -3.59467
H -3.28113 2.41778 -2.63731
H -1.56803 2.03140 -2.89558
H -2.05906 3.01843 -1.49311
H -4.52012 2.21022 0.66760
H -2.89039 2.91314 0.61769
H -3.35776 1.81098 1.94249
H 0.22413 -1.68837 -2.62630
H -0.47661 -1.76066 0.54017
H 1.47247 0.00489 3.83351
H 2.13570 -0.19411 2.17733
H 1.15815 -1.50002 2.89993
H 0.31704 2.41347 3.35136
H -0.89398 2.71986 2.05953
H 0.82540 2.46691 1.62440
H -1.66756 -1.08070 3.60578
H -2.37670 0.55716 3.44951
H -0.95954 0.30148 4.51048
H -0.05612 0.37174 -3.00342
C 2.17040 -2.66461 0.28456
C 3.13212 -3.67505 0.44072
C 4.43823 -3.48808 -0.03828
C 4.77834 -2.28975 -0.68663
C 3.82080 -1.27791 -0.85208
H 1.15020 -2.82155 0.65001
H 2.85717 -4.61037 0.93840
H 5.18741 -4.27573 0.08766
H 5.79060 -2.14121 -1.07527
H 4.09537 -0.35735 -1.37608
C 1.54696 2.56601 -1.38160
C 2.11207 3.84510 -1.27622
C 3.19947 4.07064 -0.41476
C 3.72410 3.00786 0.33764
C 3.16177 1.72444 0.23796
H 0.70828 2.39659 -2.06260
H 1.70865 4.66588 -1.87767
H 3.64135 5.06873 -0.33767
H 4.57751 3.17268 1.00290
H 3.59670 0.90230 0.81484

TS(11’’-6)2’$_{Ph}$
SCF Energy = -1123.49032984
Enthalpy 0K = -1122.951789
Enthalpy 298K = -1122.916032
Free Energy 298K = -1123.014828
SCF(DCM) = -1123.53754405
SCF(BP86-D3) = -0.11140946
Lowest Frequencies = -36.7240
29.9921 cm-1
C -2.44452 -0.35214 -1.86132
C -2.49981 0.98989 -1.26464
C -2.99806 0.85922 0.06345
C -3.22083 -0.56842 0.33754
C -2.86334 -1.30206 -0.85052
Rh -0.99051 -0.36723 0.03600
P -0.28374 0.20377 2.17680
C 1.26016 -0.60769 2.81881
C -2.29745 2.27438 -2.01665
C -3.42333 1.99589 0.95112
C -4.00995 -1.14318 1.48249
C -3.09867 -2.76872 -1.08939
C -2.50716 -0.64882 -3.33811
B 0.26639 -0.45826 -2.30764
P 1.24211 -0.12891 -0.74144
C 2.50692 -1.43747 -0.40204
C 2.07049 1.51641 -0.57929
C 0.03893 2.01462 2.45164
C -1.45613 -0.21172 3.56325
H -4.02914 -0.47929 2.35813
H -3.62422 -2.12434 1.80070
H -5.05966 -1.29176 1.16728
H -4.09523 -2.92581 -1.54312
H -3.06503 -3.34399 -0.15192
H -2.35031 -3.19306 -1.77606
H -2.05545 -1.61816 -3.59387
H -2.02734 0.12703 -3.94955
H -3.57520 -0.69441 -3.62787
H -3.22948 2.56570 -2.53689
H -1.51566 2.17644 -2.78638
H -2.02021 3.10550 -1.34945
H -4.48381 2.24404 0.75861
H -2.83670 2.90796 0.75978
H -3.34483 1.75196 2.02210
H 0.21356 -1.59723 -2.67387
H -0.48642 -1.79067 0.44394
H 1.45145 -0.25443 3.84506
H 2.12529 -0.37170 2.18475
H 1.12199 -1.69922 2.82491
H 0.36064 2.18885 3.49175
H -0.88119 2.58488 2.25611
H 0.82347 2.35117 1.75805

```
H  -1.70711 -1.28235  3.53045
H  -2.38023  0.37715  3.48059
H  -0.97313  0.02180  4.52633
H  -0.06270  0.47398 -2.98140
C   2.15033 -2.69485  0.12747
C   3.11067 -3.71123  0.24647
C   4.43573 -3.48296 -0.15715
C   4.79568 -2.23633 -0.69350
C   3.83950 -1.21761 -0.82227
H   1.11646 -2.88295  0.43552
H   2.82005 -4.68357  0.65661
H   5.18389 -4.27560 -0.05977
H   5.82309 -2.05414 -1.02366
H   4.13103 -0.25894 -1.26141
C   1.60918  2.59520 -1.36609
C   2.18124  3.86807 -1.22587
C   3.21820  4.08103 -0.30166
C   3.68561  3.01255  0.48025
C   3.11466  1.73638  0.34722
H   0.81359  2.43312 -2.09834
H   1.82425  4.69318 -1.85028
H   3.66674  5.07380 -0.19860
H   4.50069  3.16777  1.19413
H   3.50400  0.90860  0.94817
```

6_{Ph}

```
SCF Energy = -1123.51904626
Enthalpy 0K = -1122.980163
Enthalpy 298K = -1122.943569
Free Energy 298K = -1123.045502
SCF(DCM) = -1123.56689731
SCF(BP86-D3) = -0.11101529
Lowest Frequencies = 23.6461
30.8301 cm-1
C   2.58425 -0.52417  1.33394
C   3.24014 -0.75851  0.04783
C   2.50469 -1.80124 -0.62528
C   1.41871 -2.23123  0.23182
C   1.46599 -1.43479  1.44917
Rh  1.09937 -0.03138 -0.28805
P   1.30467  2.31552 -0.17875
C   0.38032  3.31632 -1.44093
C   4.57213 -0.21783 -0.39198
C   2.85184 -2.41572 -1.95022
C   0.56965 -3.44542  0.00024
C   0.63336 -1.66616  2.67801
C   3.12414  0.32680  2.44857
B  -0.33315 -0.01719 -2.31518
P  -1.24674 -0.03117 -0.62327
C  -2.35366  1.38055 -0.18007
C  -2.23697 -1.53799 -0.22606
C   0.84707  3.13633  1.42691
C   3.03770  2.94526 -0.45497
H   0.32025 -3.57646 -1.06375
H  -0.37081 -3.41249  0.56808
H   1.12617 -4.34583  0.32245
H   1.11115 -2.43056  3.31938
H  -0.37308 -2.03218  2.42287
H   0.52584 -0.75068  3.27992
H   2.33309  0.64919  3.14314
H   3.64862  1.22093  2.07819
H   3.85729 -0.25637  3.03640
H   5.36890 -0.94674 -0.15273
H   4.83166  0.72146  0.11687
H   4.60802 -0.04063 -1.47826
H   3.43047 -3.34500 -1.79447
H   3.46315 -1.74040 -2.56738
H   1.94815 -2.67821 -2.52200
H  -0.49546  0.98619 -2.96596
H   0.98940  0.22980 -1.98071
H   0.60122  4.38420 -1.28116
H  -0.70214  3.14836 -1.35090
H   0.70374  3.01873 -2.44947
H   1.09792  4.20940  1.38777
H   1.39640  2.66539  2.25579
H  -0.23380  3.02013  1.59447
H   3.42263  2.57235 -1.41607
H   3.70402  2.61143  0.35359
H   3.02588  4.04751 -0.47081
H  -0.30092 -1.08225 -2.88298
C  -2.80924 -1.72043  1.05373
C  -3.58986 -2.85476  1.32258
C  -3.81033 -3.81405  0.31914
C  -3.25392 -3.63206 -0.95703
C  -2.46892 -2.50091 -1.23272
H  -2.66942 -0.96714  1.83521
H  -4.03355 -2.98475  2.31475
H  -4.42456 -4.69502  0.52953
H  -3.43813 -4.36701 -1.74702
H  -2.04645 -2.35560 -2.23076
C  -2.52297  1.79498  1.15936
C  -3.43099  2.81939  1.47232
C  -4.17023  3.44088  0.45183
C  -4.00472  3.03425 -0.88224
C  -3.10312  2.00563 -1.20148
H  -1.94818  1.31518  1.95920
H  -3.56161  3.13154  2.51342
H  -4.87675  4.23976  0.69713
H  -4.58255  3.51327 -1.67891
H  -2.98178  1.68464 -2.24103
```

TS(11’’-6)3_{Ph}

```
SCF Energy = -1123.44407230
Enthalpy 0K = -1122.910165
Enthalpy 298K = -1122.873453
Free Energy 298K = -1122.978181
SCF(DCM) = -1123.49127509
SCF(BP86-D3) = -0.10081643
Lowest Frequencies = -756.0128
12.3439 cm-1
C  -3.03474  0.79533 -0.66094
C  -2.06943  1.75121 -0.12837
C  -2.05785  1.58557  1.30914
C  -3.07670  0.59198  1.65631
C  -3.70041  0.13529  0.45727
Rh -1.34214 -0.30859  0.31412
P  -1.21101 -2.18683 -1.10696
C   0.05837 -3.44698 -0.59680
C  -1.36435  2.81796 -0.91281
C  -1.34600  2.47075  2.29426
C  -3.44597  0.19087  3.05452
C  -4.90091 -0.76607  0.37898
```

C -3.47593 0.73441 -2.09783
B 0.44373 -0.16291 1.42371
P 1.79285 -0.23936 0.02368
C 2.13728 1.52354 -0.42117
C 3.46702 -0.97459 0.39790
C -2.75278 -3.23043 -1.20777
C -0.81502 -1.89513 -2.90326
H -2.57586 0.21253 3.72904
H -3.88464 -0.81851 3.08867
H -4.19620 0.89155 3.46686
H -5.82280 -0.19393 0.59319
H -4.85234 -1.58826 1.11117
H -5.02396 -1.20634 -0.62187
H -3.86122 -0.26010 -2.37482
H -2.65860 0.99494 -2.78805
H -4.29467 1.45752 -2.27201
H -2.02327 3.70102 -1.02104
H -1.10207 2.47439 -1.92500
H -0.43703 3.14684 -0.42144
H -1.99249 3.32599 2.56588
H -0.41060 2.87437 1.87726
H -1.09747 1.93531 3.22343
H 0.61864 0.29572 2.52280
H 0.38349 -0.46475 -0.75900
H 0.09259 -4.26935 -1.32940
H -0.20975 -3.84718 0.39315
H 1.05288 -2.97698 -0.53628
H -2.55738 -4.12878 -1.81655
H -3.57009 -2.65806 -1.67139
H -3.05947 -3.53510 -0.19569
H 0.19026 -1.45384 -2.98375
H -1.54688 -1.19267 -3.32947
H -0.84615 -2.84163 -3.46720
H -0.41760 -1.20940 1.47279
C 4.60423 -0.65465 -0.37477
C 5.82597 -1.29031 -0.11052
C 5.91892 -2.24780 0.91485
C 4.78671 -2.57221 1.68052
C 3.55902 -1.94521 1.41849
H 4.53967 0.09886 -1.16685
H 6.70979 -1.03411 -0.70344
H 6.87527 -2.73951 1.11805
H 4.85945 -3.31159 2.48448
H 2.67769 -2.19781 2.02052
C 2.03889 1.93490 -1.76727
C 2.34748 3.25812 -2.12530
C 2.74769 4.17722 -1.14185
C 2.84745 3.77264 0.20091
C 2.54841 2.44985 0.56333
H 1.72941 1.21840 -2.53572
H 2.27963 3.56828 -3.17291
H 2.98861 5.20758 -1.42156
H 3.16612 4.48645 0.96718
H 2.64292 2.13449 1.60753

INT(11’’-6)$_{Ph}$
SCF Energy = -1123.48729045
Enthalpy 0K = -1122.950591
Enthalpy 298K = -1122.913553
Free Energy 298K = -1123.019632
SCF(DCM) = -1123.53663207
SCF(BP86-D3) = -0.09817888
Lowest Frequencies = 12.8542
16.3030 cm-1
C -3.43564 -0.24416 1.09146
C -2.53579 -1.19506 1.70524
C -2.25145 -2.24174 0.73652
C -3.00932 -1.95396 -0.46382
C -3.73483 -0.72071 -0.25990
Rh -1.52322 -0.25037 -0.08045
P -1.37954 1.97441 -0.82017
C -0.34544 2.21863 -2.34451
C -2.05731 -1.16251 3.12743
C -1.44954 -3.48420 0.99133
C -3.08295 -2.82368 -1.68433
C -4.80507 -0.19068 -1.17197
C -4.12929 0.87889 1.81245
B 0.57162 -0.86578 -0.32168
P 2.25186 -0.13041 -0.87667
C 2.70353 1.44596 -0.01172
C 3.58489 -1.34507 -0.47803
C -2.96331 2.80277 -1.33804
C -0.68727 3.18042 0.41107
H -2.14844 -3.38217 -1.84610
H -3.29639 -2.23827 -2.59173
H -3.89517 -3.56582 -1.56902
H -5.73897 -0.76277 -1.01671
H -4.53505 -0.29300 -2.23514
H -5.03708 0.86545 -0.97361
H -4.49203 1.65620 1.12300
H -3.47412 1.35745 2.55707
H -5.01237 0.49007 2.35302
H -2.78269 -1.68854 3.77649
H -1.96286 -0.13316 3.50599
H -1.08546 -1.66502 3.24488
H -2.10341 -4.28261 1.38780
H -0.64797 -3.31321 1.72623
H -0.98174 -3.86360 0.06984
H 0.67232 -2.05495 -0.15832
H 0.05907 -0.05588 0.64220
H -0.29966 3.28941 -2.60217
H -0.80152 1.65864 -3.17582
H 0.67343 1.83598 -2.18174
H -2.73477 3.81802 -1.70237
H -3.65854 2.88125 -0.48920
H -3.43770 2.22869 -2.14746
H 0.33139 2.87522 0.69394
H -1.32333 3.17931 1.30990
H -0.66427 4.19485 -0.02015
H -0.39278 -0.63797 -1.31580
C 4.73975 -0.98526 0.24943
C 5.74872 -1.93356 0.47860
C 5.62191 -3.24087 -0.01746
C 4.48018 -3.59896 -0.75467
C 3.47130 -2.65619 -0.99468
H 4.84962 0.02974 0.64221
H 6.63771 -1.64620 1.04930
H 6.41307 -3.97559 0.16114
H 4.38010 -4.61277 -1.15550
H 2.59593 -2.93865 -1.58988
C 3.24062 2.51160 -0.76781
C 3.62025 3.70981 -0.13997

C 3.47380 3.85344 1.24910
C 2.95103 2.79528 2.01313
C 2.56580 1.59752 1.38909
H 3.36976 2.39776 -1.84957
H 4.03775 4.52734 -0.73625
H 3.77556 4.78478 1.73866
H 2.85032 2.89913 3.09853
H 2.17485 0.76994 1.98994

TS(11''-6)4_{Ph}
SCF Energy = -1123.46265178
Enthalpy 0K = -1122.926683
Enthalpy 298K = -1122.889835
Free Energy 298K = -1122.994440
SCF(DCM) = -1123.51128011
SCF(BP86-D3) = -0.09664338
Lowest Frequencies = -120.8580
16.6725 cm-1
C -3.42426 0.60756 0.56389
C -3.66624 -0.53032 -0.33083
C -3.19039 -0.16494 -1.65725
C -2.64902 1.16818 -1.58412
C -2.79880 1.64563 -0.21280
Rh -1.47927 -0.21879 -0.19574
P -0.79632 -1.58665 1.58648
C 0.42346 -2.94121 1.21077
C -4.49476 -1.74318 -0.02666
C -3.31545 -0.99949 -2.89767
C -2.14732 1.97712 -2.74449
C -2.41434 3.00914 0.27736
C -3.94076 0.76215 1.96801
B 0.50262 -0.15101 -1.55467
P 1.94252 -0.00690 -0.32358
C 3.44447 -1.01583 -0.56468
C 2.40955 1.69562 0.17910
C -0.04450 -0.68881 3.03515
C -2.13189 -2.58356 2.42445
H -1.63859 1.34850 -3.49126
H -1.44237 2.75989 -2.42484
H -2.99506 2.47819 -3.24721
H -3.22358 3.73266 0.06077
H -1.50275 3.37927 -0.21742
H -2.24330 3.02007 1.36469
H -3.26706 1.37394 2.58864
H -4.08123 -0.20750 2.46920
H -4.92547 1.26510 1.95652
H -5.52705 -1.58586 -0.39310
H -4.55991 -1.94500 1.05160
H -4.10513 -2.64364 -0.52804
H -4.25387 -0.75451 -3.42976
H -3.33645 -2.07507 -2.66461
H -2.48325 -0.81568 -3.59505
H 0.65982 -0.60601 -2.66331
H -0.39486 -1.13432 -1.13160
H 0.65026 -3.50430 2.13058
H 1.35465 -2.52199 0.80497
H -0.02074 -3.62049 0.46661
H 0.23667 -1.39933 3.83050
H -0.77292 0.03542 3.43260
H 0.84237 -0.14108 2.68507
H -2.60682 -3.25984 1.69795
H -2.89503 -1.91959 2.85564
H -1.68677 -3.18325 3.23524
H 0.04404 0.97911 -1.45057
C 2.92800 1.93675 1.47324
C 3.29628 3.23461 1.85887
C 3.13694 4.30669 0.96501
C 2.61717 4.07694 -0.31977
C 2.25764 2.77979 -0.71731
H 3.04601 1.10738 2.17917
H 3.70203 3.40884 2.86040
H 3.42239 5.31894 1.26772
H 2.50659 4.90810 -1.02372
H 1.88151 2.60006 -1.72860
C 4.71838 -0.62584 -0.08494
C 5.83023 -1.44974 -0.30144
C 5.68965 -2.67228 -0.98155
C 4.42687 -3.06790 -1.45645
C 3.30818 -2.25158 -1.24682
H 4.84120 0.32925 0.43413
H 6.81385 -1.13333 0.05975
H 6.56189 -3.31263 -1.14441
H 4.31404 -4.01288 -1.99717
H 2.33138 -2.55765 -1.63730

TS(11''-9')2_{Ph}
SCF Energy = -1123.48011730
Enthalpy 0K = -1122.941286
Enthalpy 298K = -1122.905652
Free Energy 298K = -1123.005054
SCF(DCM) = -1123.52623466
SCF(BP86-D3) = -0.10572648
Lowest Frequencies = -116.7700
31.4364 cm-1
C 1.11519 2.11279 1.04052
C 1.94292 2.08342 -0.12593
C 3.02901 1.13815 0.12009
C 2.86499 0.56348 1.42456
C 1.63500 1.11954 1.99121
Rh 0.97959 -0.02298 0.28788
P 1.34901 -2.19911 -1.46442
C 0.48138 -3.73633 -0.89293
C 1.82863 2.97229 -1.33253
C 4.20170 0.96664 -0.80275
C 3.84663 -0.29999 2.16856
C 1.17217 0.95006 3.41072
C 0.00492 3.07911 1.32621
B -0.17199 -0.79389 -1.84455
P -1.20307 -0.14292 -0.31234
C -2.18271 1.36994 -0.67835
C -2.38641 -1.35636 0.41231
C 1.77124 -2.52616 -3.25492
C 3.04954 -2.42449 -0.71881
H 4.48876 -0.88114 1.48938
H 3.33779 -1.00669 2.84270
H 4.51181 0.32785 2.79010
H 1.66559 1.69501 4.06333
H 1.41781 -0.04979 3.79952
H 0.08504 1.09529 3.50307
H -0.75724 2.64879 1.99370
H -0.49796 3.41973 0.40895
H 0.42133 3.97104 1.83120

```
H   2.46026  3.87301 -1.21678
H   0.79310  3.31243 -1.48809
H   2.15813  2.45910 -2.25049
H   4.80822  1.89162 -0.80359
H   3.88948  0.79527 -1.84688
H   4.86626  0.14666 -0.49178
H   0.17288  0.00429 -2.67722
H   0.71387 -1.35932  1.09723
H   1.17275 -4.59070 -0.96124
H  -0.39976 -3.92024 -1.52422
H   0.15615 -3.60586  0.14939
H   2.36980 -3.44755 -3.33509
H   2.35586 -1.68003 -3.64885
H   0.84837 -2.62291 -3.84416
H   2.97396 -2.40750  0.37583
H   3.71719 -1.62246 -1.05942
H   3.44762 -3.39528 -1.06152
H  -0.76345 -1.76315 -2.26618
C  -3.23436  1.75376  0.18462
C  -3.95854  2.92857 -0.06992
C  -3.64208  3.72841 -1.18045
C  -2.59926  3.34868 -2.04317
C  -1.86970  2.17583 -1.79636
H  -3.49535  1.12960  1.04520
H  -4.77372  3.21687  0.60118
H  -4.21233  4.64123 -1.37840
H  -2.36214  3.95968 -2.91997
H  -1.07187  1.87300 -2.48061
C  -3.57992 -1.66016 -0.28138
C  -4.47567 -2.60096  0.24711
C  -4.19423 -3.23686  1.46884
C  -3.01403 -2.93045  2.16489
C  -2.11025 -1.99191  1.64005
H  -3.80803 -1.16391 -1.23048
H  -5.39551 -2.83766 -0.29678
H  -4.89738 -3.96858  1.87874
H  -2.79693 -3.41766  3.12081
H  -1.18795 -1.74790  2.17772
```

9'$_{Ph}$
SCF Energy = -1123.49750371
Enthalpy 0K = -1122.960805
Enthalpy 298K = -1122.923325
Free Energy 298K = -1123.030752
SCF(DCM) = -1123.54640180
SCF(BP86-D3) = -0.10098690
Lowest Frequencies = 6.2332
23.0974 cm-1

```
C   2.76223  1.03928  0.19282
C   3.21004  0.03429 -0.69875
C   3.35926 -1.23711  0.05201
C   3.00405 -1.00853  1.41047
C   2.52451  0.38524  1.49850
Rh  1.17643 -0.61547  0.20574
P  -2.17593 -2.48935 -1.47954
C  -3.30949 -2.74301 -0.04364
C   3.56078  0.20317 -2.14760
C   3.88782 -2.50998 -0.53997
C   3.14430 -1.95895  2.56562
C   2.15500  1.09846  2.76159
C   2.60779  2.50381 -0.08197
B  -1.61433 -0.65527 -1.83791
P  -0.86719  0.35938 -0.28206
C  -0.77761  2.12168 -0.86720
C  -2.16783  0.41620  1.04449
C  -3.10216 -3.10796 -2.95603
C  -0.78649 -3.68717 -1.24281
H   3.10200 -3.00923  2.23907
H   2.35374 -1.80749  3.31638
H   4.11674 -1.80310  3.06839
H   3.06171  1.53768  3.22180
H   1.70558  0.41571  3.49864
H   1.44918  1.92150  2.57005
H   1.78631  2.94541  0.50238
H   2.39963  2.70275 -1.14403
H   3.53729  3.03846  0.18898
H   4.65853  0.25774 -2.27322
H   3.13017  1.12575 -2.56462
H   3.21010 -0.64683 -2.75638
H   4.98936 -2.46197 -0.63280
H   3.48759 -2.68521 -1.55212
H   3.64437 -3.38330  0.08386
H  -0.78797 -0.74631 -2.72605
H   0.27037 -1.59009  1.07640
H  -3.64515 -3.79108 -0.00434
H  -4.17927 -2.07700 -0.14867
H  -2.78356 -2.48172  0.88700
H  -3.40383 -4.15726 -2.81291
H  -2.45919 -3.02540 -3.84567
H  -3.99538 -2.48297 -3.10707
H  -0.18186 -3.36190 -0.37944
H  -0.15642 -3.68374 -2.14610
H  -1.17068 -4.70446 -1.06765
H  -2.61724 -0.06677 -2.19216
C  -0.97121  3.19169  0.03421
C  -0.84671  4.51930 -0.40647
C  -0.52484  4.79237 -1.74621
C  -0.33024  3.73207 -2.64718
C  -0.45455  2.40263 -2.21286
H  -1.23946  2.98832  1.07628
H  -1.00875  5.34116  0.29847
H  -0.43440  5.82784 -2.08896
H  -0.09440  3.93908 -3.69616
H  -0.32183  1.58411 -2.92741
C  -3.44213  0.94514  0.73592
C  -4.44803  0.97700  1.71325
C  -4.19459  0.48424  3.00553
C  -2.93005 -0.03769  3.31927
C  -1.91822 -0.06987  2.34371
H  -3.64540  1.33540 -0.26697
H  -5.43147  1.38948  1.46578
H  -4.98097  0.51226  3.76635
H  -2.72611 -0.41584  4.32632
H  -0.93011 -0.47691  2.58188
```

TS(9'-9)$_{Ph}$
SCF Energy = -1123.49506800
Enthalpy 0K = -1122.958032
Enthalpy 298K = -1122.921489
Free Energy 298K = -1123.024871
SCF(DCM) = -1123.54302570
SCF(BP86-D3) = -0.09814710

Lowest Frequencies = -34.9701 21.1670 cm-1

```
C   -2.77046 -0.30444  1.16167
C   -3.06316 -1.24423  0.14181
C   -3.55934 -0.50643 -1.04138
C   -3.60089  0.88644 -0.73560
C   -3.01557  1.04771  0.60517
Rh  -1.46447  0.27684 -0.60843
P    2.84065 -1.46165 -2.01094
C    3.48059 -2.75124 -0.84846
C   -2.98081 -2.74092  0.23643
C   -4.02202 -1.15812 -2.31116
C   -4.18711  1.98427 -1.57855
C   -2.97314  2.32392  1.39017
C   -2.34961 -0.58981  2.57241
B    1.09266 -0.67022 -1.65377
P    0.73420  0.26688  0.06295
C    1.08571 -0.75404  1.57020
C    1.75394  1.80432  0.23936
C    2.79120 -2.29021 -3.66420
C    4.17665 -0.19309 -2.14458
H   -4.12050  1.75394 -2.65281
H   -3.67580  2.94401 -1.40955
H   -5.25544  2.12489 -1.33038
H   -3.92019  2.45654  1.94821
H   -2.84719  3.19838  0.73410
H   -2.15156  2.32146  2.12283
H   -1.64635  0.16823  2.95167
H   -1.86030 -1.57156  2.66359
H   -3.23197 -0.58746  3.23936
H   -3.98128 -3.17318  0.42547
H   -2.32096 -3.05927  1.05800
H   -2.60728 -3.18974 -0.69870
H   -5.03458 -1.58313 -2.17557
H   -3.36030 -1.98818 -2.60814
H   -4.06928 -0.44146 -3.14485
H    1.01938  0.16790 -2.52893
H   -0.98506  1.54168 -1.42908
H    4.47277 -3.09703 -1.17912
H    2.78559 -3.60475 -0.83418
H    3.55129 -2.33860  0.16891
H    3.78596 -2.68049 -3.93084
H    2.46558 -1.55998 -4.42065
H    2.06565 -3.11751 -3.63472
H    4.26773  0.35436 -1.19434
H    3.90294  0.52421 -2.93349
H    5.13603 -0.67179 -2.39579
H    0.26071 -1.55774 -1.80008
C    1.37098 -0.16355  2.82196
C    1.52615 -0.96357  3.96623
C    1.39026 -2.35846  3.88062
C    1.09029 -2.95382  2.64307
C    0.93447 -2.15821  1.49726
H    1.48119  0.92236  2.90382
H    1.75327 -0.49194  4.92770
H    1.51486 -2.97902  4.77337
H    0.98045 -4.04087  2.56952
H    0.68641 -2.62850  0.53945
C    3.05072  1.75662  0.80228
C    3.83368  2.91924  0.87753
C    3.33506  4.13900  0.38976
C    2.04852  4.19294 -0.16897
C    1.25842  3.03407 -0.23969
H    3.44378  0.81537  1.20161
H    4.83314  2.87181  1.32188
H    3.94620  5.04481  0.45133
H    1.65189  5.14160 -0.54447
H    0.25109  3.07542 -0.66709
```

9_{Ph}

SCF Energy = -1123.51851123
Enthalpy 0K = -1122.980637
Enthalpy 298K = -1122.943789
Free Energy 298K = -1123.048229
SCF(DCM) = -1123.56470102
SCF(BP86-D3) = -0.09966562
Lowest Frequencies = 19.2594 34.1011 cm-1

```
C    2.58352  1.34432  0.52375
C    3.10251  0.62075 -0.59606
C    3.44613 -0.73138 -0.14143
C    3.14839 -0.82850  1.26220
C    2.55340  0.43955  1.67935
Rh   1.21601 -0.45154  0.20522
B   -0.43306 -0.88341 -1.50944
P   -1.49238 -2.51817 -1.66517
C   -1.60346 -3.53947 -0.13475
C    3.37078  1.16908 -1.97012
C    4.12745 -1.77793 -0.97770
C    3.48884 -1.97920  2.16894
C    2.24259  0.83773  3.09553
C    2.20996  2.79830  0.54859
P   -0.89697  0.37941 -0.09190
C   -2.32792 -0.00939  1.01406
C   -1.15472  2.11292 -0.66243
C   -3.22586 -2.17983 -2.20489
C   -0.76830 -3.62281 -2.95993
H    1.90953 -0.02392  3.69433
H    1.45511  1.60574  3.13881
H    3.14295  1.25547  3.58416
H    3.09837  3.41211  0.78727
H    1.44599  3.01128  1.31190
H    1.81859  3.13888 -0.42268
H    2.72836  2.03499 -2.19351
H    3.20549  0.41209 -2.75345
H    4.42090  1.50538 -2.05553
H    5.22284 -1.62535 -0.96391
H    3.80535 -1.73378 -2.03004
H    3.93040 -2.79306 -0.59986
H    4.49905 -1.83831  2.59606
H    3.48669 -2.93989  1.63132
H    2.78121 -2.05956  3.00800
H    0.74162 -1.41990 -1.19756
H    0.48812 -1.55367  1.08073
H   -3.76273 -3.12505 -2.38052
H   -3.20722 -1.58816 -3.13304
H   -3.73818 -1.60403 -1.41867
H   -1.38087 -4.53005 -3.08034
H    0.25318 -3.90726 -2.66344
H   -0.72370 -3.07805 -3.91540
H   -2.12528 -2.96659  0.64682
H   -0.58411 -3.76462  0.21461
```

```
H  -2.14661 -4.47499 -0.34075
H  -0.33716 -0.40985 -2.61836
C  -1.68059  3.07506  0.22977
C  -1.81043  4.41313 -0.17277
C  -1.41386  4.80631 -1.46152
C  -0.88398  3.85526 -2.34982
C  -0.75111  2.51517 -1.95495
H  -1.99996  2.77875  1.23413
H  -2.22383  5.14921  0.52398
H  -1.51985  5.84978 -1.77389
H  -0.58091  4.15441 -3.35838
H  -0.34518  1.77867 -2.65488
C  -3.64058  0.36110  0.63749
C  -4.72735  0.03937  1.46547
C  -4.51698 -0.64697  2.67417
C  -3.21473 -1.00559  3.05817
C  -2.12191 -0.68545  2.23466
H  -3.81031  0.91596 -0.29147
H  -5.73968  0.33125  1.16815
H  -5.36640 -0.89187  3.31935
H  -3.04498 -1.52498  4.00677
H  -1.10242 -0.95240  2.53270
```

10_{Cy}

SCF Energy = -1171.25892618
Enthalpy 0K = -1170.538943
Enthalpy 298K = -1170.496656
Free Energy 298K = -1170.612002
SCF(DCM) = -1171.30295617
SCF(BP86-D3) = -0.13241044
Lowest Frequencies = 12.3763 14.3405 cm-1

```
C   2.80633  1.82354  0.93636
C   3.80446  0.79940  0.65672
C   3.48735 -0.36648  1.49482
C   2.27233 -0.10229  2.18940
C   1.83543  1.25062  1.82144
Rh  1.90020  0.04216 -0.13167
B  -0.53015 -0.83358  0.10776
P  -2.30626 -0.30268 -0.57162
C  -3.55784 -1.55124  0.07663
C   5.09778  1.03458 -0.07239
C   4.39411 -1.54586  1.70526
C   1.61933 -0.96478  3.23127
C   0.66235  1.95893  2.43593
C   2.85432  3.24355  0.45125
P   2.33835 -1.77487 -1.49976
C   4.00808 -1.78293 -2.32345
C   1.80323  1.16662 -1.93058
C   2.28216 -3.43360 -0.65531
C   1.20838 -2.04237 -2.95640
C  -2.83605  1.47108 -0.26423
H   1.85320  3.70143  0.41352
H   3.30070  3.31931 -0.55285
H   3.47139  3.85677  1.13345
H   5.81830  1.56151  0.58098
H   4.95279  1.65843 -0.96881
H   5.57205  0.09228 -0.38634
H   4.92509 -1.84017  0.78585
H   3.85014 -2.42290  2.08874
H   5.16910 -1.29019  2.45181
H   1.85333 -0.59288  4.24621
H   1.96462 -2.00871  3.17322
H   0.52228 -0.96954  3.12616
H   0.94249  2.36593  3.42558
H  -0.18651  1.27444  2.59068
H   0.32156  2.80371  1.81738
H  -0.61305 -0.68458  1.30580
H  -2.42330 -0.42570 -1.99641
H   1.47591 -2.98178 -3.46636
H   1.30735 -1.20594 -3.66375
H   0.16798 -2.09944 -2.60434
H   4.11096 -2.66305 -2.97908
H   4.80278 -1.80837 -1.56267
H   4.12186 -0.86659 -2.92284
H   1.28070 -3.57624 -0.22200
H   3.02665 -3.46177  0.15362
H   2.49530 -4.24208 -1.37396
H   2.80225  1.23517 -2.39523
H   1.09817  0.75245 -2.66854
H   1.46454  2.18190 -1.66752
H   0.19546  0.03365 -0.53010
H  -0.44036 -1.98976 -0.24624
C  -4.27647  1.79673 -0.72368
H  -2.77080  1.58585  0.83669
C  -4.62422  3.27326 -0.43285
H  -4.36388  1.60396 -1.81115
H  -5.00551  1.13761 -0.22215
C  -3.61007  4.23797 -1.07269
H  -5.64532  3.48639 -0.79459
H  -4.64126  3.42966  0.66372
C  -2.17373  3.90597 -0.62882
H  -3.67826  4.16468 -2.17574
H  -3.85909  5.28148 -0.81236
C  -1.81362  2.43351 -0.92061
H  -1.44846  4.56830 -1.13411
H  -2.07199  4.09581  0.45829
H  -1.81647  2.27037 -2.01691
H  -0.79080  2.21067 -0.56953
C  -4.75239 -1.81365 -0.87049
H  -2.94718 -2.47579  0.12476
C  -5.65148 -2.93175 -0.29978
H  -5.34920 -0.89074 -0.99015
H  -4.39075 -2.09082 -1.87742
C  -6.11912 -2.61122  1.13230
H  -6.51804 -3.08175 -0.96740
H  -5.08682 -3.88470 -0.29873
C  -4.92362 -2.34048  2.06515
H  -6.77786 -1.72063  1.11243
H  -6.72960 -3.44158  1.52782
C  -4.01785 -1.21695  1.51645
H  -5.27296 -2.06961  3.07703
H  -4.32478 -3.26563  2.17578
H  -4.58410 -0.26564  1.51607
H  -3.14490 -1.07397  2.17867
```

TS(10-11’’)1_{Cy}

SCF Energy = -1171.23104503
Enthalpy 0K = -1170.513547
Enthalpy 298K = -1170.471535
Free Energy 298K = -1170.585311
SCF(DCM) = -1171.27758456

SCF(BP86-D3) = -0.13704275
Lowest Frequencies = -876.3015
11.9766 cm-1
C 2.49680 2.00667 0.60668
C 3.67475 1.22516 0.25428
C 3.68566 0.06393 1.09546
C 2.50518 0.10316 1.95803
C 1.80760 1.34067 1.68579
Rh 1.75344 -0.02442 -0.17834
B -0.31594 -0.80770 0.31866
P -2.06078 -0.23230 -0.45124
C -3.34007 -1.50789 0.09635
C 4.72675 1.64507 -0.73436
C 4.81455 -0.91823 1.23635
C 2.21545 -0.82273 3.10671
C 0.67887 1.89662 2.50512
C 2.18741 3.37116 0.05897
P 2.09260 -2.13817 -1.06210
C 3.73155 -2.34761 -1.93001
C 1.29031 0.73535 -2.24029
C 2.08411 -3.56056 0.13754
C 0.92320 -2.71194 -2.39414
C -2.66478 1.52243 -0.14296
H 1.12877 3.64305 0.19098
H 2.43232 3.45077 -1.01299
H 2.78983 4.13595 0.58338
H 5.39774 2.40830 -0.29867
H 4.28172 2.08856 -1.64157
H 5.35549 0.79811 -1.05080
H 5.46805 -0.93501 0.35066
H 4.46172 -1.94347 1.43072
H 5.44493 -0.63062 2.09830
H 2.59023 -0.39648 4.05609
H 2.69807 -1.80371 2.97568
H 1.13266 -0.99278 3.22262
H 1.07851 2.39042 3.41045
H -0.01074 1.10476 2.83674
H 0.09695 2.65070 1.95272
H -0.45951 -0.55673 1.49863
H -2.14207 -0.32312 -1.88431
H 1.19363 -3.73220 -2.71099
H 0.99350 -2.04017 -3.26310
H -0.10697 -2.70990 -2.01157
H 3.81323 -3.35889 -2.36149
H 4.55528 -2.19342 -1.21846
H 3.81543 -1.60258 -2.73647
H 1.11698 -3.58157 0.66083
H 2.88645 -3.41982 0.87769
H 2.24545 -4.51386 -0.39174
H 2.29151 0.56945 -2.66457
H 0.56081 0.26366 -2.91968
H 1.08152 1.81334 -2.19169
H 0.47707 0.21055 -1.12302
H -0.41445 -2.00179 0.09611
C -4.09659 1.81411 -0.65131
H -2.64371 1.62425 0.96095
C -4.49892 3.27717 -0.36183
H -4.14070 1.62905 -1.74285
H -4.82499 1.13166 -0.18216
C -3.49158 4.27869 -0.95377
H -5.51121 3.46307 -0.76159
H -4.56374 3.42345 0.73423
C -2.06468 3.98452 -0.45652
H -3.51406 4.21318 -2.05931
H -3.78199 5.31187 -0.69529
C -1.65384 2.52616 -0.74892
H -1.34045 4.67418 -0.92633
H -2.01155 4.16528 0.63563
H -1.61206 2.37705 -1.84683
H -0.64072 2.32526 -0.35946
C -4.48319 -1.76266 -0.91393
H -2.72150 -2.42656 0.15006
C -5.38090 -2.91847 -0.42134
H -5.09671 -0.85097 -1.03252
H -4.06956 -1.99830 -1.91166
C -5.92389 -2.65268 0.99567
H -6.21090 -3.06875 -1.13405
H -4.79352 -3.85775 -0.42067
C -4.78096 -2.38352 1.99282
H -6.60153 -1.77652 0.96973
H -6.53328 -3.50803 1.33605
C -3.87519 -1.22577 1.52111
H -5.18501 -2.15103 2.99387
H -4.16705 -3.29848 2.10574
H -4.46105 -0.28634 1.52198
H -3.03680 -1.08527 2.22685

INT(10-11’’)$_{Cy}$
SCF Energy = -1171.24386441
Enthalpy 0K = -1170.523122
Enthalpy 298K = -1170.480446
Free Energy 298K = -1170.594628
SCF(DCM) = -1171.28886353
SCF(BP86-D3) = -0.13708551
Lowest Frequencies = 23.8603
27.4137 cm-1
C 2.57305 1.97160 0.50431
C 3.73299 1.16039 0.09432
C 3.75954 0.01131 0.91616
C 2.58514 0.06817 1.82797
C 1.93165 1.35951 1.62606
Rh 1.70751 -0.09205 -0.12318
B -0.29415 -0.74013 0.57670
P -1.96335 -0.19420 -0.33977
C -3.32449 -1.43247 0.09296
C 4.73176 1.56636 -0.95251
C 4.85183 -1.01501 1.00298
C 2.37267 -0.82487 3.01574
C 0.90684 1.97482 2.53310
C 2.27564 3.32646 -0.07002
P 1.82504 -2.26380 -0.91255
C 3.35186 -2.61276 -1.93487
C 1.14344 0.79934 -2.71095
C 1.86389 -3.64760 0.33009
C 0.49188 -2.81810 -2.09338
C -2.53371 1.57557 -0.04859
H 1.27666 3.69151 0.21008
H 2.35482 3.33528 -1.16978
H 3.01377 4.05919 0.30720
H 5.41468 2.34600 -0.56621
H 4.24376 1.98718 -1.84810
H 5.35353 0.71726 -1.27620

H 5.46402 -1.05012 0.08847
H 4.46329 -2.02665 1.20143
H 5.52944 -0.76638 1.84124
H 2.90412 -0.41653 3.89669
H 2.75973 -1.84092 2.84244
H 1.30514 -0.90297 3.27224
H 1.40486 2.43930 3.40465
H 0.19856 1.22196 2.91186
H 0.32621 2.76237 2.02728
H -0.47136 -0.28749 1.69449
H -1.95620 -0.26948 -1.77700
H 0.69660 -3.84594 -2.43457
H 0.47131 -2.14868 -2.96670
H -0.48289 -2.78718 -1.58725
H 3.33499 -3.64865 -2.31249
H 4.25567 -2.46739 -1.32536
H 3.38547 -1.91735 -2.78815
H 0.95310 -3.60400 0.94491
H 2.74429 -3.53559 0.98118
H 1.92227 -4.61941 -0.18658
H 2.20801 0.55155 -2.80474
H 0.57053 0.28484 -3.49651
H 1.00043 1.88645 -2.78336
H 0.67849 0.47866 -1.72742
H -0.45634 -1.95166 0.58948
C -3.94577 1.92234 -0.57208
H -2.52421 1.67253 1.05581
C -4.29887 3.39507 -0.26714
H -3.98366 1.75175 -1.66610
H -4.70334 1.26186 -0.11868
C -3.24943 4.36885 -0.83171
H -5.29893 3.62409 -0.67525
H -4.37080 3.52777 0.83025
C -1.83918 4.01662 -0.32309
H -3.26063 4.32057 -1.93841
H -3.50599 5.40792 -0.56098
C -1.48071 2.54793 -0.63199
H -1.08645 4.68733 -0.77599
H -1.79235 4.18093 0.77195
H -1.44016 2.41189 -1.73173
H -0.47904 2.29473 -0.24061
C -4.44249 -1.57647 -0.96422
H -2.75217 -2.38241 0.11781
C -5.42539 -2.69728 -0.55996
H -4.99956 -0.62735 -1.06238
H -4.00602 -1.79050 -1.95729
C -6.00127 -2.46285 0.84932
H -6.23702 -2.76297 -1.30599
H -4.89705 -3.67089 -0.58384
C -4.88081 -2.32046 1.89537
H -6.61793 -1.54224 0.84454
H -6.67821 -3.28978 1.12660
C -3.88663 -1.20056 1.51697
H -5.30454 -2.11464 2.89423
H -4.33042 -3.27819 1.97888
H -4.41039 -0.22570 1.55889
H -3.06138 -1.15470 2.25022

TS(10-11’’)2_{Cy}
SCF Energy = -1171.24268206
Enthalpy 0K = -1170.524114
Enthalpy 298K = -1170.480955
Free Energy 298K = -1170.597980
SCF(DCM) = -1171.28823084
SCF(BP86-D3) = -0.13380075
Lowest Frequencies = -25.0841
18.4530 cm-1
C 3.67662 1.08045 -0.15187
C 3.88397 -0.11182 0.56610
C 2.91490 -0.13411 1.69717
C 2.24446 1.16762 1.74075
C 2.62843 1.85452 0.55134
Rh 1.62072 -0.15903 0.01157
B -0.32621 -0.64384 0.86487
P -1.92206 -0.12310 -0.19426
C -2.55741 1.63848 0.01611
C 4.95864 -1.13695 0.34837
C 2.92164 -1.12820 2.81899
C 1.45003 1.70039 2.89592
C 2.22026 3.23814 0.14088
C 4.42229 1.56679 -1.36162
P 1.52348 -2.26127 -0.96625
C 1.73386 -3.70467 0.18840
C 0.00017 -2.75354 -1.92317
C 2.84629 -2.55743 -2.25077
C -3.31504 -1.35779 0.15499
C 0.80755 1.15912 -3.15028
H 1.32539 3.58305 0.67788
H 2.02474 3.30351 -0.94183
H 3.03997 3.94783 0.36204
H 5.17757 2.32380 -1.07813
H 3.75004 2.04380 -2.09359
H 4.95218 0.74835 -1.87319
H 5.37798 -1.08946 -0.66869
H 4.60357 -2.16439 0.53093
H 5.79215 -0.96092 1.05415
H 3.65373 -0.81987 3.59076
H 3.21029 -2.13372 2.47668
H 1.93394 -1.19593 3.29975
H 2.13295 2.03861 3.69740
H 0.78202 0.93521 3.32158
H 0.82999 2.56305 2.60653
H -0.48269 -0.04176 1.91359
H -1.79951 -0.22815 -1.62316
H 0.15084 -3.74476 -2.38102
H -0.19655 -2.01681 -2.71688
H -0.86161 -2.79715 -1.24199
H 2.76769 -3.57689 -2.66352
H 3.84136 -2.42525 -1.80264
H 2.72680 -1.82570 -3.06444
H 0.95770 -3.64664 0.96594
H 2.72446 -3.65955 0.66700
H 1.64482 -4.65482 -0.36311
H 1.67595 1.07177 -3.81950
H -0.02661 0.56109 -3.54499
H 0.50081 2.21234 -3.07178
H 1.09460 0.78824 -2.14005
H -0.53460 -1.84158 1.00776
C -3.51809 2.09983 -1.10598
H -3.10839 1.60891 0.97780
C -4.02132 3.53720 -0.84875
H -2.98016 2.06528 -2.07429

H -4.37890 1.41758 -1.19985
C -2.85781 4.52903 -0.67306
H -4.67647 3.85018 -1.68080
H -4.65020 3.54185 0.06304
C -1.89134 4.05983 0.42971
H -2.30502 4.61909 -1.62930
H -3.24524 5.53627 -0.44023
C -1.38097 2.62779 0.16401
H -1.03465 4.75368 0.51181
H -2.40741 4.08381 1.40962
H -0.78563 2.62114 -0.77080
H -0.70904 2.29062 0.97134
C -4.44689 -1.42288 -0.89218
H -2.76103 -2.31905 0.12578
C -5.42198 -2.57286 -0.55442
H -5.01113 -0.47186 -0.89428
H -4.03357 -1.55218 -1.90928
C -5.98929 -2.42630 0.87012
H -6.23827 -2.59596 -1.29781
H -4.88835 -3.53987 -0.64262
C -4.86458 -2.33006 1.91712
H -6.61572 -1.51384 0.92046
H -6.65560 -3.27486 1.10444
C -3.87178 -1.19241 1.58891
H -5.28570 -2.17106 2.92551
H -4.31200 -3.28935 1.95588
H -4.39854 -0.22118 1.66855
H -3.04714 -1.17650 2.32382

11''$_{Cy}$
SCF Energy = -1130.73285223
Enthalpy 0K = -1130.060204
Enthalpy 298K = -1130.020495
Free Energy 298K = -1130.130033
SCF(DCM) = -1130.77873946
SCF(BP86-D3) = -0.12632675
Lowest Frequencies = 13.3988 22.6011 cm-1
C -2.08383 -0.89866 -2.10000
C -1.85057 0.50464 -2.19667
C -2.88289 1.20328 -1.39027
C -3.71750 0.24335 -0.77972
C -3.15509 -1.08124 -1.10772
Rh -1.48309 -0.19485 -0.06959
B 0.36097 -1.32159 -0.30417
P 1.91016 -0.42822 0.50107
C 1.91814 1.42328 0.20011
C -0.86936 1.18185 -3.11113
C -3.05032 2.69356 -1.34032
C -4.99439 0.50572 -0.03165
C -3.81134 -2.39772 -0.80993
C -1.48589 -1.98744 -2.94105
P -1.69865 -0.96453 2.12036
C -3.42786 -0.91190 2.82259
C -1.21123 -2.72097 2.50643
C -0.79697 -0.01556 3.45800
C 3.52462 -1.22639 -0.06593
H -2.08214 3.21709 -1.28760
H -3.66066 3.00962 -0.48051
H -3.55725 3.05145 -2.25646
H -5.84049 0.59241 -0.73898
H -4.95353 1.44505 0.54333
H -5.24320 -0.30797 0.66552
H -4.33986 -2.38949 0.15598
H -3.08404 -3.22400 -0.80512
H -4.56457 -2.62294 -1.58951
H -2.18167 -2.24089 -3.76311
H -1.29882 -2.90363 -2.35995
H -0.53188 -1.67795 -3.39166
H -1.31953 1.36153 -4.10549
H 0.03035 0.56399 -3.25867
H -0.55274 2.16147 -2.71777
H 0.39971 -2.45899 0.12826
H 0.68854 -1.26754 -1.48133
H 2.02767 -0.48794 1.93318
H -1.01678 -0.46213 4.44187
H -1.13222 1.03338 3.45700
H 0.28943 -0.04494 3.28899
H -3.41596 -1.22326 3.87996
H -4.08369 -1.59483 2.26225
H -3.82791 0.11025 2.74707
H -0.14094 -2.86203 2.29864
H -1.77637 -3.40447 1.85422
H -1.42542 -2.95646 3.56169
C 3.10288 2.19808 0.82629
H 1.96411 1.52066 -0.90372
C 3.00246 3.70758 0.51688
H 3.09885 2.04333 1.92324
H 4.06318 1.80244 0.45392
C 1.65422 4.29612 0.96850
H 3.83730 4.24007 1.00498
H 3.12672 3.86271 -0.57265
C 0.46904 3.51729 0.35882
H 1.58489 4.25531 2.07311
H 1.58560 5.36189 0.68927
C 0.58132 2.01787 0.68980
H -0.48986 3.91691 0.73403
H 0.46888 3.65201 -0.74078
H 0.48761 1.87598 1.78151
H -0.29503 1.46763 0.21735
C 4.66181 -1.17394 0.98017
H 3.21630 -2.28423 -0.19318
C 5.89766 -1.94735 0.46953
H 4.94549 -0.12223 1.17558
H 4.31929 -1.59507 1.94304
C 6.36322 -1.42977 -0.90399
H 6.71229 -1.87100 1.21107
H 5.64338 -3.02256 0.39124
C 5.22244 -1.47624 -1.93744
H 6.72213 -0.38660 -0.80052
H 7.22410 -2.02136 -1.26128
C 3.98072 -0.69931 -1.44709
H 5.55671 -1.06242 -2.90521
H 4.93764 -2.52959 -2.12684
H 4.23838 0.37497 -1.36855
H 3.15979 -0.78503 -2.18164

TS(11''-11)$_{Cy}$
SCF Energy = -1130.72503462
Enthalpy 0K = -1130.052420
Enthalpy 298K = -1130.012963
Free Energy 298K = -1130.122139

SCF(DCM) = -1130.77108809
SCF(BP86-D3) = -0.11774398
Lowest Frequencies = -21.0852
14.6139 cm-1

C	-2.52622	-0.40647	-2.12113
C	-2.90375	0.92730	-1.79156
C	-3.91969	0.88976	-0.70304
C	-4.12513	-0.45461	-0.34000
C	-3.15797	-1.26731	-1.11229
Rh	-1.82753	0.03125	-0.04131
B	0.20267	-0.21577	-0.71670
P	1.93613	-0.03195	0.22072
C	2.81243	1.56707	-0.24344
C	-2.48308	2.17516	-2.50713
C	-4.63418	2.10016	-0.17933
C	-5.16155	-1.00501	0.59552
C	-3.08301	-2.76323	-1.08944
C	-1.70836	-0.84901	-3.29757
P	-1.42203	-0.40032	2.21305
C	-2.92658	-0.64680	3.28932
C	-0.41414	-1.90273	2.66497
C	-0.57867	0.98747	3.13952
C	3.00860	-1.55410	-0.07438
H	-3.93891	2.93587	0.00654
H	-5.17056	1.88620	0.75787
H	-5.37901	2.45965	-0.91424
H	-6.07068	-1.28388	0.03041
H	-5.46368	-0.27318	1.36051
H	-4.81457	-1.91601	1.10822
H	-3.28712	-3.16810	-0.08587
H	-2.09424	-3.12088	-1.41380
H	-3.83950	-3.18921	-1.77714
H	-2.32247	-0.82375	-4.21605
H	-1.33577	-1.87608	-3.16943
H	-0.83410	-0.19635	-3.45268
H	-3.25226	2.46670	-3.24761
H	-1.53405	2.03242	-3.04492
H	-2.36553	3.02235	-1.81161
H	0.29497	-1.32305	-1.22227
H	0.25832	0.68426	-1.55002
H	1.96386	0.05308	1.65752
H	-0.41492	0.70047	4.19153
H	-1.22027	1.88196	3.10451
H	0.38535	1.23235	2.67169
H	-2.62343	-0.76298	4.34286
H	-3.47368	-1.54699	2.97334
H	-3.59238	0.22470	3.19814
H	0.58869	-1.82889	2.22109
H	-0.90876	-2.79687	2.25492
H	-0.32574	-2.00033	3.75967
C	4.24833	1.71187	0.30681
H	2.84544	1.54103	-1.35121
C	4.87000	3.06391	-0.10938
H	4.22598	1.64364	1.41260
H	4.88564	0.88571	-0.05132
C	3.99095	4.25344	0.31528
H	5.88074	3.15112	0.32674
H	5.00113	3.07759	-1.20913
C	2.56104	4.10694	-0.23454
H	3.95460	4.30754	1.42130
H	4.43836	5.20184	-0.02997
C	1.92727	2.76357	0.18793
H	1.92292	4.93967	0.11125
H	2.58208	4.16436	-1.34047
H	1.81875	2.75159	1.29199
H	0.91403	2.66413	-0.24002
C	4.10870	-1.81421	0.97960
H	2.25599	-2.36684	0.00422
C	4.82564	-3.15071	0.68913
H	4.85091	-0.99534	0.96310
H	3.67401	-1.82530	1.99645
C	5.39288	-3.18958	-0.74281
H	5.62983	-3.30651	1.42977
H	4.10889	-3.98479	0.82406
C	4.29521	-2.91752	-1.78847
H	6.18993	-2.42643	-0.84177
H	5.87057	-4.16595	-0.93640
C	3.56845	-1.58308	-1.51727
H	4.72267	-2.90504	-2.80661
H	3.55492	-3.74144	-1.76792
H	4.28123	-0.74661	-1.65392
H	2.75283	-1.43450	-2.24776

11_{Cy}
SCF Energy = -1130.74557428
Enthalpy 0K = -1130.072193
Enthalpy 298K = -1130.032564
Free Energy 298K = -1130.142874
SCF(DCM) = -1130.79039981
SCF(BP86-D3) = -0.11770655
Lowest Frequencies = 13.2549
24.5465 cm-1

C	4.12600	-0.23754	-0.40195
C	3.57885	1.11427	-0.27070
C	2.71577	1.37785	-1.42158
C	2.62034	0.15506	-2.16671
C	3.49577	-0.84113	-1.52502
Rh	1.78234	-0.17292	-0.10075
B	-0.17948	-0.09834	-0.60172
P	-1.94295	-0.09154	0.25762
C	-3.12588	-1.38629	-0.42968
C	4.08111	2.17123	0.67400
C	2.15069	2.71697	-1.80487
C	1.91276	-0.03697	-3.47797
C	3.75542	-2.22233	-2.05514
C	5.23399	-0.83361	0.42072
P	1.47217	-0.38053	2.18537
C	0.61662	1.01231	3.09161
C	0.52134	-1.88928	2.74153
C	3.00938	-0.56260	3.22816
C	-2.64353	1.65277	0.09740
H	2.85606	-2.66043	-2.51509
H	4.10091	-2.90497	-1.26336
H	4.54041	-2.19267	-2.83433
H	6.21194	-0.63951	-0.05862
H	5.13279	-1.92629	0.52065
H	5.28214	-0.40178	1.43145
H	4.44252	1.74455	1.62260
H	3.30444	2.91698	0.90413
H	4.93279	2.71155	0.21901
H	2.89255	3.28893	-2.39407
H	1.89578	3.32062	-0.91992

```
H   1.24472  2.61535 -2.42122
H   2.59462  0.18293 -4.32004
H   1.04230  0.63102 -3.57169
H   1.55739 -1.07235 -3.60257
H  -0.46310  0.21419 -1.74405
H  -1.99306 -0.34819  1.66781
H   0.38749 -1.89046  3.83592
H   1.08211 -2.78783  2.44004
H  -0.46355 -1.92064  2.25331
H   2.73317 -0.70497  4.28605
H   3.63117  0.34140  3.14332
H   3.59003 -1.43125  2.88370
H  -0.39080  1.16226  2.67616
H   1.19184  1.93855  2.93636
H   0.54321  0.80332  4.17204
H   0.44970 -1.28990 -0.45431
C  -4.51706 -1.37023  0.24487
H  -3.23111 -1.11625 -1.49943
C  -5.43261 -2.45984 -0.35667
H  -4.39647 -1.55078  1.33144
H  -4.99362 -0.38078  0.13684
C  -4.79081 -3.85571 -0.27137
H  -6.40588 -2.44552  0.16453
H  -5.64067 -2.21275 -1.41602
C  -3.40174 -3.86840 -0.93499
H  -4.69274 -4.15106  0.79181
H  -5.44667 -4.60642 -0.74535
C  -2.47418 -2.78864 -0.33691
H  -2.92594 -4.85892 -0.82655
H  -3.50879 -3.68855 -2.02255
H  -2.27866 -3.02913  0.72792
H  -1.49679 -2.79592 -0.85191
C  -3.54910  2.06708  1.28177
H  -1.72870  2.27922  0.12351
C  -4.01161  3.53128  1.12103
H  -4.43576  1.40687  1.32661
H  -3.01236  1.94024  2.24002
C  -4.70624  3.76152 -0.23414
H  -4.68577  3.79576  1.95448
H  -3.13270  4.20061  1.20312
C  -3.79859  3.34375 -1.40557
H  -5.64452  3.17367 -0.26951
H  -4.99775  4.82117 -0.33692
C  -3.33596  1.87682 -1.26949
H  -4.32206  3.47303 -2.36905
H  -2.90995  4.00409 -1.43963
H  -4.21848  1.21370 -1.35135
H  -2.65485  1.61101 -2.09747
```

TS(11-11')$_{Cy}$
SCF Energy = -1130.73930064
Enthalpy 0K = -1130.067731
Enthalpy 298K = -1130.028289
Free Energy 298K = -1130.138006
SCF(DCM) = -1130.78478388
SCF(BP86-D3) = -0.11803709
Lowest Frequencies = -346.8194
16.2589 cm-1

```
C  -4.08564  0.29597 -0.28227
C  -3.59876 -0.96247 -0.84790
C  -2.71225 -0.64865 -1.94962
C  -2.56176  0.78680 -1.99304
C  -3.42736  1.36433 -0.95891
Rh -1.72513  0.08323 -0.02029
B   0.15780 -0.02908 -0.57497
P   1.93723 -0.01315  0.28334
C   2.92537  1.54143 -0.10511
C  -4.14713 -2.32996 -0.54068
C  -2.14918 -1.62763 -2.94294
C  -1.84658  1.56645 -3.06212
C  -3.68113  2.83302 -0.75854
C  -5.20331  0.44454  0.71392
P  -1.47141 -0.69633  2.13827
C  -0.52619 -2.28608  2.40477
C  -0.66795  0.47303  3.35114
C  -3.04648 -1.08373  3.05717
C   2.84959 -1.58394 -0.22632
H  -2.79736  3.43923 -1.01059
H  -3.96134  3.06026  0.28180
H  -4.51011  3.17287 -1.40746
H  -6.16529  0.58363  0.18564
H  -5.06490  1.32172  1.36687
H  -5.31554 -0.44487  1.35206
H  -4.49110 -2.41417  0.50239
H  -3.40060 -3.11992 -0.71765
H  -5.01926 -2.54910 -1.18512
H  -2.83563 -1.73958 -3.80307
H  -2.00993 -2.62565 -2.49893
H  -1.17737 -1.29351 -3.33913
H  -2.51637  1.73996 -3.92459
H  -0.96069  1.02884 -3.43534
H  -1.51762  2.55193 -2.69677
H   0.43955 -0.02348 -1.76162
H   1.95186 -0.05761  1.71599
H  -0.58201  0.01058  4.34837
H  -1.28790  1.38052  3.41869
H   0.33127  0.76762  2.99773
H  -2.81393 -1.40202  4.08681
H  -3.59029 -1.89214  2.54588
H  -3.68172 -0.18654  3.08752
H   0.51225 -2.16671  2.06089
H  -0.99765 -3.08131  1.80672
H  -0.52658 -2.57536  3.46908
H  -0.76449  1.29667  0.41036
C   4.30242  1.59003  0.59797
H   3.07223  1.49845 -1.20276
C   5.05063  2.89551  0.24669
H   4.15216  1.53501  1.69424
H   4.91805  0.71841  0.31522
C   4.21055  4.14267  0.57559
H   6.01270  2.91842  0.78790
H   5.29794  2.89066 -0.83276
C   2.83897  4.08585 -0.12086
H   4.06347  4.20859  1.67159
H   4.75330  5.05627  0.27729
C   2.07482  2.79237  0.23187
H   2.22384  4.95971  0.15694
H   2.97782  4.13506 -1.21862
H   1.84005  2.79270  1.31523
H   1.10871  2.76117 -0.30266
C   3.82508 -2.10978  0.85413
H   2.02651 -2.31973 -0.33792
```

C   4.46560 -3.43932 0.39997
H   4.62173 -1.36337 1.03375
H   3.29813 -2.24807 1.81622
C   5.15524 -3.29791 -0.96988
H   5.18507 -3.77832 1.16575
H   3.68055 -4.21852 0.33974
C   4.17895 -2.76723 -2.03583
H   6.01227 -2.60212 -0.87864
H   5.57360 -4.26887 -1.28674
C   3.53851 -1.42992 -1.60431
H   4.69477 -2.62908 -3.00210
H   3.37898 -3.51301 -2.21028
H   4.32812 -0.65706 -1.53713
H   2.81508 -1.08837 -2.36607

11'$_{Cy}$
SCF Energy = -1130.74027814
Enthalpy 0K = -1130.067010
Enthalpy 298K = -1130.027432
Free Energy 298K = -1130.136866
SCF(DCM) = -1130.78554586
SCF(BP86-D3) = -0.11847805
Lowest Frequencies = 15.6440
21.7771 cm-1
C   4.05565 0.37876 0.21735
C   3.61446 -0.69864 1.09888
C   2.68202 -0.15002 2.05413
C   2.47603 1.24340 1.71717
C   3.34364 1.56310 0.58260
Rh 1.70707 0.04372 -0.03448
B -0.14400 -0.09121 0.59698
P -1.92035 -0.05920 -0.27555
C -2.78649 1.59914 -0.06119
C   4.22112 -2.07401 1.17468
C   2.13236 -0.85308 3.26354
C   1.72015 2.24690 2.54575
C   3.55842 2.93900 0.01254
C   5.17660 0.30483 -0.78434
P   1.49980 -1.34047 -1.86863
C   0.48338 -2.89573 -1.67990
C   0.77734 -0.56217 -3.40193
C   3.08155 -2.04546 -2.56318
C -2.96742 -1.50067 0.34782
H   2.64717 3.55360 0.07249
H   3.87197 2.90033 -1.04235
H   4.35109 3.46699 0.57508
H   6.12645 0.63575 -0.32414
H   4.99514 0.95563 -1.65518
H   5.33976 -0.72012 -1.15110
H   4.62736 -2.40260 0.20545
H   3.49304 -2.82750 1.51460
H   5.06238 -2.08279 1.89290
H   2.75879 -0.64200 4.15016
H   2.11125 -1.94535 3.12522
H   1.10820 -0.52214 3.50011
H   2.38298 2.68898 3.31284
H   0.87069 1.78381 3.07193
H   1.33094 3.07384 1.93108
H -0.41102 -0.07083 1.78685
H -1.90918 -0.22885 -1.69891
H   0.70597 -1.29644 -4.22120
H   1.42700 0.27190 -3.70929
H -0.22175 -0.15565 -3.18507
H   2.85849 -2.65319 -3.45565
H   3.57486 -2.68080 -1.81226
H   3.75986 -1.22635 -2.84423
H -0.56156 -2.63041 -1.45767
H   0.88068 -3.47786 -0.83414
H   0.51578 -3.50571 -2.59804
H   0.97821 1.08742 -0.96609
C -4.17239 1.66236 -0.74531
H -2.91157 1.69924 1.03530
C -4.81660 3.05247 -0.54546
H -4.05192 1.46409 -1.82853
H -4.84203 0.88003 -0.34797
C -3.89928 4.18542 -1.03935
H -5.78811 3.07962 -1.06933
H -5.03616 3.19683 0.53038
C -2.51859 4.11342 -0.36227
H -3.77502 4.10629 -2.13725
H -4.36874 5.16578 -0.84758
C -1.85725 2.73366 -0.56306
H -1.84963 4.89848 -0.75655
H -2.62790 4.30825 0.72259
H -1.64968 2.58254 -1.64129
H -0.88219 2.69314 -0.04659
C -3.98098 -2.02947 -0.69398
H -2.21022 -2.29274 0.52465
C -4.72544 -3.26407 -0.14030
H -4.71436 -1.23789 -0.93605
H -3.46733 -2.28497 -1.63907
C -5.39875 -2.96301 1.21161
H -5.47124 -3.60377 -0.88013
H -4.00465 -4.09614 -0.01625
C -4.38308 -2.42809 2.23774
H -6.20097 -2.21339 1.06424
H -5.88950 -3.87154 1.60146
C -3.63983 -1.18281 1.70592
H -4.88511 -2.17560 3.18800
H -3.64287 -3.21772 2.47302
H -4.36522 -0.35707 1.57489
H -2.88955 -0.84077 2.44123

TS(11''-9')1$_{Cy}$
SCF Energy = -1130.72889739
Enthalpy 0K = -1130.055693
Enthalpy 298K = -1130.016778
Free Energy 298K = -1130.124227
SCF(DCM) = -1130.77639936
SCF(BP86-D3) = -0.12509170
Lowest Frequencies = -39.4603
16.3274 cm-1
C -3.64770 -0.64390 0.26232
C -2.97216 -1.23187 1.35449
C -1.89457 -2.06680 0.80327
C -1.95524 -2.05830 -0.63084
C -2.97075 -1.08100 -0.99214
Rh -1.46572 0.06234 0.01063
P -1.84081 2.34832 -0.17863
C -2.49526 2.93192 -1.82000
C -3.31603 -1.12455 2.81382
C -1.01259 -2.92759 1.65987

```
C  -1.24617 -2.96938 -1.59188
C  -3.49823 -0.86374 -2.37914
C  -4.93785  0.12097  0.29913
B   0.10415  0.40866 -1.43775
P   1.47615  0.05035 -0.05250
C   2.14482 -1.70968 -0.11492
C   3.00481  1.17445  0.17759
C  -3.07889  3.05530  1.02888
C  -0.41585  3.51158  0.12028
H  -4.98596  0.90560 -0.47302
H  -5.12315  0.58007  1.28232
H  -5.77816 -0.57021  0.09759
H  -3.88754 -2.00965  3.15158
H  -3.93206 -0.23634  3.02332
H  -2.41447 -1.06338  3.44540
H  -0.62397 -2.37523  2.53149
H  -0.16169 -3.34551  1.10241
H  -1.59747 -3.77835  2.05751
H  -0.79901 -2.41393 -2.43206
H  -1.95705 -3.70427 -2.01245
H  -0.44518 -3.54287 -1.09966
H  -4.25985 -1.63236 -2.61604
H  -2.69790 -0.94140 -3.13019
H  -3.98147  0.11893 -2.49010
H   0.20273 -0.48356 -2.25876
H   0.91811  0.09478  1.28645
H  -0.75621  4.55195 -0.00942
H   0.39554  3.30031 -0.58960
H  -0.05120  3.38074  1.15093
H  -2.59812  4.02947 -1.82523
H  -3.48123  2.48027 -2.00957
H  -1.79941  2.61554 -2.61082
H  -2.72627  2.87467  2.05616
H  -4.05990  2.57790  0.90196
H  -3.18576  4.14091  0.86830
H   0.28381  1.51732 -1.89935
C   2.81023 -2.16139  1.20321
H   1.23606 -2.31537 -0.29280
C   3.30324 -3.62158  1.09658
H   3.67539 -1.50659  1.42672
H   2.10720 -2.05418  2.04970
C   4.24322 -3.81781 -0.10725
H   3.80572 -3.91123  2.03616
H   2.42735 -4.29244  0.99019
C   3.57663 -3.35893 -1.41683
H   5.17220 -3.23654  0.05545
H   4.54742 -4.87624 -0.18347
C   3.09084 -1.89548 -1.32661
H   4.27457 -3.46169 -2.26607
H   2.71190 -4.01410 -1.64137
H   3.97280 -1.23457 -1.21352
H   2.58236 -1.59800 -2.26064
C   2.94024  2.05960  1.43967
H   3.81927  0.43583  0.32170
C   4.24766  2.86622  1.60234
H   2.09050  2.76311  1.35531
H   2.75817  1.44124  2.33763
C   4.56761  3.69107  0.34175
H   4.16838  3.52117  2.48801
H   5.08087  2.16495  1.80474
C   4.61937  2.79993 -0.91267
H   3.79208  4.47084  0.20633
H   5.52557  4.22434  0.47061
C   3.31365  1.99552 -1.09272
H   4.80333  3.40807 -1.81570
H   5.47151  2.09685 -0.83002
H   2.47682  2.69106 -1.29424
H   3.38368  1.33577 -1.97583
```

INT(11′′-9')$_{Cy}$

SCF Energy = -1130.75116721
Enthalpy 0K = -1130.075282
Enthalpy 298K = -1130.036546
Free Energy 298K = -1130.141110
SCF(DCM) = -1130.79765704
SCF(BP86-D3) = -0.13239274
Lowest Frequencies = 26.7662 28.9552 cm-1

```
C  -1.22519 -2.05286  1.14916
C  -1.90264 -2.23451 -0.12937
C  -3.03310 -1.35555 -0.15322
C  -3.08334 -0.63896  1.12209
C  -1.96270 -1.07483  1.91840
Rh -1.15005  0.01018  0.07951
P   1.20535  0.09417 -0.50161
C   1.97647  1.72351  0.07760
C  -1.63611 -3.29877 -1.15835
C  -4.11053 -1.38556 -1.20078
C  -4.25701  0.13670  1.65355
C  -1.71832 -0.72614  3.36194
C  -0.16661 -2.94559  1.72970
P  -1.94004  1.95024 -0.98620
C  -3.14399  2.93089  0.05080
B   0.09105  0.01516 -2.00795
C  -0.78234  3.34780 -1.41900
C  -2.89402  1.72579 -2.56975
C   2.33441 -1.39545 -0.13820
H  -2.11273  0.27004  3.61340
H  -0.64631 -0.73872  3.61125
H  -2.22072 -1.45929  4.02014
H  -0.65353 -3.77867  2.27146
H   0.46452 -2.41423  2.45894
H   0.48179 -3.39153  0.96349
H  -0.83040 -3.97903 -0.84533
H  -1.36336 -2.87535 -2.13980
H  -2.54277 -3.91288 -1.30518
H  -4.75165 -2.27472 -1.04977
H  -3.69469 -1.45278 -2.21830
H  -4.77074 -0.50600 -1.14949
H  -4.87963 -0.52822  2.28103
H  -4.90723  0.51552  0.85045
H  -3.94896  0.98478  2.28412
H  -0.10080 -1.06771 -2.49491
H  -0.59801  1.11540  1.04629
H  -1.36111  4.10446 -1.97385
H   0.05464  3.00749 -2.04221
H  -0.40120  3.80837 -0.49435
H  -3.25570  2.70613 -2.92071
H  -3.75541  1.06484 -2.39850
H  -2.24143  1.27585 -3.33085
H  -2.68054  3.17105  1.02018
H  -4.06915  2.36728  0.22671
```

H -3.39368 3.86737 -0.47426
H -0.06440 0.99334 -2.68548
C 3.11121 2.26485 -0.82704
H 1.11778 2.40826 -0.03282
C 3.53629 3.67518 -0.36022
H 3.98826 1.59628 -0.78926
H 2.77940 2.29025 -1.88046
C 3.94221 3.68280 1.12504
H 4.36659 4.03562 -0.99272
H 2.69638 4.38164 -0.51460
C 2.80875 3.14118 2.01595
H 4.84618 3.05696 1.26079
H 4.21974 4.70376 1.44053
C 2.35941 1.73001 1.57659
H 3.12364 3.11502 3.07419
H 1.94198 3.83036 1.96477
H 3.18225 1.01589 1.76715
H 1.49922 1.39638 2.18646
C 3.84200 -1.04195 -0.22163
H 2.11324 -1.71343 0.89764
C 4.70808 -2.28095 0.10210
H 4.07869 -0.68867 -1.24352
H 4.10243 -0.22751 0.47270
C 4.38892 -3.46362 -0.82742
H 5.77453 -2.00386 0.02767
H 4.53467 -2.57830 1.15517
C 2.88732 -3.79735 -0.79425
H 4.68096 -3.20436 -1.86377
H 4.98718 -4.34714 -0.54413
C 2.02920 -2.55461 -1.11399
H 2.65111 -4.60073 -1.51454
H 2.62061 -4.18628 0.20931
H 2.24639 -2.21480 -2.14392
H 0.95884 -2.81042 -1.10036

TS(11’’-6)2_{Cy}
SCF Energy = -1130.73614891
Enthalpy 0K = -1130.062120
Enthalpy 298K = -1130.023528
Free Energy 298K = -1130.127501
SCF(DCM) = -1130.78182223
SCF(BP86-D3) = -0.13054770
Lowest Frequencies = -30.6197
26.5414 cm-1
C -2.45861 1.38116 1.53221
C -2.68562 -0.03029 1.87655
C -3.30670 -0.65776 0.75640
C -3.48306 0.34960 -0.29773
C -2.94667 1.59827 0.19541
Rh -1.25307 0.10393 -0.09963
P 1.07942 0.20736 0.51838
C 2.23307 -1.31191 0.69774
C -2.57828 -0.64267 3.24643
C -3.90026 -2.04028 0.77013
C -4.40663 0.24616 -1.48163
C -3.08184 2.93784 -0.47810
C -2.20270 2.50250 2.50325
P -0.89029 -1.54391 -1.69568
C 0.30292 -1.10934 -3.05604
B 0.34793 0.56388 2.20551
C -0.34018 -3.22838 -1.11455
C -2.34589 -2.03353 -2.75377
C 1.96446 1.75234 -0.13992
H -3.09775 2.84328 -1.57437
H -2.25739 3.61535 -0.20853
H -4.02640 3.42470 -0.17055
H -3.16893 2.98862 2.73877
H -1.54238 3.27834 2.08497
H -1.76336 2.15392 3.44758
H -1.93122 -0.05803 3.91499
H -2.18088 -1.66966 3.20978
H -3.58201 -0.69492 3.70906
H -4.91261 -2.01193 1.21562
H -3.30024 -2.73915 1.37411
H -4.00975 -2.46443 -0.23941
H -5.37403 0.71867 -1.22785
H -4.62216 -0.79442 -1.76128
H -4.01432 0.77166 -2.36693
H 0.20877 1.70327 2.53813
H -0.71239 1.02071 -1.24755
H -0.31021 -3.93171 -1.96335
H -1.05376 -3.59526 -0.36061
H 0.65641 -3.17001 -0.65804
H -2.00000 -2.75884 -3.50866
H -2.75873 -1.15272 -3.26554
H -3.13208 -2.50639 -2.14803
H 1.25766 -0.75196 -2.65031
H -0.14305 -0.29996 -3.65540
H 0.47685 -1.98658 -3.70025
H 0.13070 -0.35451 2.94416
C 3.10135 -1.21690 1.98248
H 1.50565 -2.13212 0.85811
C 3.89115 -2.52475 2.20800
H 3.81286 -0.37600 1.89067
H 2.46335 -1.00394 2.85720
C 4.76513 -2.87711 0.99209
H 4.50978 -2.42409 3.11722
H 3.17894 -3.35093 2.40355
C 3.91833 -2.95194 -0.28995
H 5.55079 -2.10616 0.86772
H 5.29000 -3.83387 1.15904
C 3.11551 -1.65157 -0.52422
H 4.55588 -3.15239 -1.16924
H 3.21334 -3.80431 -0.21343
H 3.82394 -0.82093 -0.70595
H 2.51130 -1.75552 -1.44118
C 2.17835 1.80854 -1.66751
H 1.21302 2.52425 0.12603
C 2.75741 3.17693 -2.09159
H 2.87957 1.00923 -1.97717
H 1.22145 1.62556 -2.18880
C 4.05699 3.50516 -1.33454
H 2.92938 3.18329 -3.18256
H 2.00491 3.96428 -1.88755
C 3.84255 3.43795 0.18780
H 4.84511 2.78342 -1.62723
H 4.42594 4.50429 -1.62524
C 3.27360 2.06892 0.62178
H 4.78887 3.62845 0.72428
H 3.13986 4.23624 0.49767
H 4.02470 1.28516 0.40510
H 3.09992 2.05649 1.71247

INT(11''-6)2_{Cy}
SCF Energy = -1130.73896453
Enthalpy 0K = -1130.063845
Enthalpy 298K = -1130.024888
Free Energy 298K = -1130.129878
SCF(DCM) = -1130.78504454
SCF(BP86-D3) = -0.13101470
Lowest Frequencies = 30.1781
35.3430 cm-1

C	-2.39961	-0.54603	-2.02854
C	-2.60135	0.84111	-1.58671
C	-3.28787	0.80663	-0.34005
C	-3.47886	-0.59919	0.05113
C	-2.91262	-1.41917	-0.98832
Rh	-1.24027	-0.29672	0.06861
P	1.08672	-0.16403	-0.48680
C	1.99857	1.51172	-0.47749
C	-2.34968	2.05743	-2.43177
C	-3.91385	1.99626	0.33309
C	-4.40410	-1.10987	1.12332
C	-3.04133	-2.91365	-1.10545
C	-2.29802	-0.97426	-3.47192
P	-0.93422	0.61772	2.18331
C	0.45606	0.00579	3.25514
B	0.23655	-0.51013	-2.12500
C	-0.70153	2.46707	2.21428
C	-2.33001	0.38319	3.39773
C	2.18678	-1.67807	-0.12982
H	-3.12167	-3.39399	-0.11855
H	-2.17844	-3.35360	-1.62899
H	-3.94971	-3.17555	-1.67994
H	-3.32808	-1.01724	-3.87767
H	-1.85784	-1.97507	-3.58440
H	-1.72196	-0.27086	-4.08773
H	-1.42012	1.96111	-3.01458
H	-2.28011	2.97423	-1.82572
H	-3.17564	2.20256	-3.15354
H	-4.93925	2.14266	-0.05473
H	-3.35760	2.92479	0.13028
H	-4.00344	1.87583	1.42318
H	-5.38129	-1.36981	0.67523
H	-4.59883	-0.36278	1.90563
H	-4.01437	-2.02004	1.60564
H	0.20311	-1.65367	-2.48737
H	-0.73733	-1.62104	0.73929
H	-0.62866	2.82840	3.25348
H	-1.55761	2.94977	1.71995
H	0.21520	2.73415	1.66847
H	-2.02787	0.79891	4.37313
H	-2.54281	-0.69032	3.50975
H	-3.24101	0.89782	3.06405
H	1.42641	0.11282	2.75419
H	0.28941	-1.05761	3.48426
H	0.46333	0.58180	4.19502
H	0.02044	0.40774	-2.86433
C	2.77888	1.76745	-1.79362
H	1.14658	2.22175	-0.44785
C	3.35955	3.19817	-1.81538
H	3.60646	1.03963	-1.88113
H	2.11981	1.60751	-2.66342
C	4.25134	3.46890	-0.59090
H	3.92724	3.34690	-2.75091
H	2.52684	3.92929	-1.83503
C	3.48925	3.19769	0.71812
H	5.14552	2.81649	-0.63542
H	4.62250	4.50849	-0.60722
C	2.89933	1.76958	0.75145
H	4.14848	3.34437	1.59199
H	2.66481	3.93199	0.81984
H	3.72866	1.03641	0.76081
H	2.33920	1.62383	1.69189
C	2.51103	-2.02640	1.33570
H	1.52506	-2.47341	-0.52836
C	3.30183	-3.35235	1.42371
H	3.11227	-1.21722	1.79475
H	1.57734	-2.11605	1.91516
C	4.57917	-3.31674	0.56755
H	3.54485	-3.56482	2.47999
H	2.65200	-4.18078	1.07849
C	4.24946	-2.96746	-0.89386
H	5.27722	-2.56072	0.97836
H	5.10241	-4.28734	0.62148
C	3.46926	-1.63816	-0.99699
H	5.17127	-2.89696	-1.49794
H	3.64220	-3.77900	-1.34042
H	4.12183	-0.81351	-0.65078
H	3.21507	-1.42833	-2.05100

6_{Cy}(=7)
SCF Energy = -1130.76581971
Enthalpy 0K = -1130.090191
Enthalpy 298K = -1130.051279
Free Energy 298K = -1130.156786
SCF(DCM) = -1130.81367653
SCF(BP86-D3) = -0.13226625
Lowest Frequencies = 19.1795
30.6876 cm-1

C	-3.09070	1.92092	-1.21443
C	-2.15889	1.48757	-0.05398
C	-2.94027	1.36681	1.27368
C	-3.64970	2.69720	1.61071
C	-4.57384	3.14818	0.46478
C	-3.80623	3.24491	-0.86657
P	-1.07991	-0.01306	-0.50666
C	-2.02940	-1.63359	-0.28611
C	-2.33320	-2.07697	1.16245
C	-2.96909	-3.48513	1.18492
C	-4.23494	-3.54557	0.31060
C	-3.94110	-3.08218	-1.12773
C	-3.31204	-1.67113	-1.15586
Rh	1.32353	-0.01801	-0.27609
P	1.49034	2.31660	-0.04928
C	3.21586	3.01227	-0.15770
C	2.99806	-0.54164	1.16698
C	3.49962	-0.69525	-0.19608
C	2.71727	-1.73387	-0.82451
C	1.75104	-2.23023	0.13405
C	1.92353	-1.48488	1.37176
C	3.65330	0.23269	2.27560
C	4.75071	-0.08783	-0.76915
C	2.93428	-2.28961	-2.20253

```
C   0.94430 -3.48283 -0.04962
C   1.30991 -1.79890  2.70777
B  -0.20217  0.05562 -2.21674
C   0.90395  3.04213  1.56454
C   0.68478  3.36685 -1.36098
H   4.35671 -0.43137  2.81240
H   2.92371  0.59333  3.01688
H   4.24076  1.08854  1.91278
H   5.04437  0.83422 -0.24739
H   4.64346  0.14517 -1.84047
H   5.59206 -0.79893 -0.67141
H   3.56714 -3.19473 -2.14751
H   3.44097 -1.56692 -2.85959
H   1.98451 -2.57475 -2.68113
H   0.56224 -3.58120 -1.07795
H   0.09602 -3.54229  0.64752
H   1.58763 -4.36260  0.14143
H   0.39070 -2.39383  2.61009
H   1.06635 -0.88781  3.27573
H   2.02012 -2.38917  3.31730
H   1.13228  0.31577 -1.93711
H  -0.18931 -1.00758 -2.79214
H  -0.35894  1.06561 -2.86182
H  -1.31356 -2.35068 -0.73069
H  -1.41442 -2.05411  1.77365
H  -3.03324 -1.36438  1.63340
H  -3.20180 -3.76935  2.22664
H  -2.23529 -4.22849  0.81346
H  -5.01484 -2.89475  0.75290
H  -4.64816 -4.56933  0.30693
H  -4.86518 -3.08223 -1.73252
H  -3.24867 -3.79737 -1.61392
H  -3.08361 -1.37353 -2.19397
H  -4.04802 -0.94532 -0.76153
H  -1.41240  2.29211  0.08347
H  -3.70594  0.57429  1.18001
H  -2.26933  1.06613  2.09938
H  -4.22031  2.58375  2.54942
H  -2.88879  3.48090  1.80073
H  -5.40074  2.41911  0.35657
H  -5.04194  4.11773  0.70908
H  -3.05593  4.05842 -0.79927
H  -4.48900  3.51978 -1.68987
H  -2.51552  2.02366 -2.15117
H  -3.84587  1.13483 -1.39653
H   1.02449  4.13793  1.56297
H   1.50259  2.61325  2.38311
H  -0.15160  2.78700  1.73673
H  -0.39233  3.17476 -1.44726
H   1.15279  3.12951 -2.32878
H   0.85059  4.43015 -1.12277
H   3.66479  2.73464 -1.12287
H   3.84253  2.62868  0.65894
H   3.17010  4.11124 -0.08265
```

TS(11’’-6)3_{Cy}
SCF Energy = -1130.69999668
Enthalpy 0K = -1130.030847
Enthalpy 298K = -1129.991383
Free Energy 298K = -1130.101135
SCF(DCM) = -1130.74634928
SCF(BP86-D3) = -0.12126533
Lowest Frequencies = -791.7445 13.0716 cm-1

```
C  -3.94281  0.05313  0.30202
C  -3.23227  0.83202 -0.71219
C  -2.36287  1.78496 -0.03351
C  -2.43115  1.48805  1.38051
C  -3.42295  0.42574  1.57239
Rh -1.54567 -0.26958  0.27110
B   0.23137 -0.05507  1.39315
P   1.62005 -0.10344  0.02577
C   3.35392 -0.82879  0.48915
C  -3.58381  0.88104 -2.17451
C  -1.69925  2.97014 -0.67437
C  -1.83183  2.30271  2.49206
C  -3.86363 -0.10821  2.90440
C  -5.10111 -0.87509  0.06516
P  -1.32347 -2.10666 -1.19279
C  -0.86304 -1.75626 -2.96261
C  -0.05001 -3.35216 -0.66241
C  -2.83267 -3.18461 -1.38762
C   1.94085  1.71401 -0.42370
H  -3.03516 -0.13012  3.62958
H  -4.27575 -1.12585  2.82166
H  -4.65526  0.53497  3.33290
H  -6.05103 -0.36286  0.30654
H  -5.05274 -1.77782  0.69566
H  -5.17057 -1.19339 -0.98567
H  -3.88357 -0.10479 -2.56472
H  -2.74763  1.25312 -2.78645
H  -4.43849  1.56474 -2.33566
H  -2.43622  3.78420 -0.81407
H  -1.28715  2.72669 -1.66640
H  -0.88442  3.36838 -0.05139
H  -2.56042  3.05862  2.83936
H  -0.92439  2.83479  2.16762
H  -1.56246  1.67918  3.35866
H   0.34118  0.40856  2.49966
H   0.17710 -0.35460 -0.73832
H   0.03537 -4.15535 -1.41190
H  -0.34577 -3.78085  0.30753
H   0.92610 -2.85319 -0.55266
H  -2.58278 -4.06195 -2.00678
H  -3.64274 -2.62404 -1.87678
H  -3.17832 -3.52244 -0.39910
H   0.12733 -1.27693 -2.98935
H  -1.60157 -1.06893 -3.40200
H  -0.83784 -2.68910 -3.54873
H  -0.59506 -1.13899  1.42374
C   3.99658 -1.59518 -0.68631
H   3.97269  0.06072  0.72408
C   5.37974 -2.15351 -0.28530
H   3.33614 -2.43703 -0.97801
H   4.08776 -0.94687 -1.57596
C   5.28900 -3.03709  0.97264
H   5.80979 -2.72107 -1.12967
H   6.06887 -1.30774 -0.09290
C   4.63042 -2.28119  2.14147
H   4.69427 -3.94286  0.74032
H   6.29340 -3.39032  1.26480
C   3.24832 -1.71377  1.74812
```

H 4.52183 -2.94129 3.02049
H 5.28651 -1.44631 2.45679
H 2.55533 -2.55598 1.54614
H 2.81641 -1.14229 2.58939
C 2.85169 1.82538 -1.66996
H 0.93459 2.08543 -0.69627
C 3.03032 3.29934 -2.09313
H 3.84708 1.39904 -1.43784
H 2.43792 1.23161 -2.50568
C 3.57234 4.15552 -0.93395
H 3.70441 3.35446 -2.96628
H 2.05334 3.70347 -2.42519
C 2.67359 4.04091 0.31064
H 4.59583 3.81752 -0.67787
H 3.66117 5.21090 -1.24596
C 2.48052 2.57033 0.74464
H 3.09498 4.62173 1.15022
H 1.68347 4.48711 0.08788
H 3.45558 2.16826 1.08291
H 1.80133 2.51304 1.61401

INT(11′′-6)$_{Cy}$
SCF Energy = -1130.74035842
Enthalpy 0K = -1130.067556
Enthalpy 298K = -1130.028162
Free Energy 298K = -1130.137539
SCF(DCM) = -1130.78809185
SCF(BP86-D3) = -0.11825769
Lowest Frequencies = 14.0849
20.2913 cm-1
C -3.84997 -1.00307 -0.32379
C -3.81811 -0.13758 0.85636
C -2.92688 -0.73152 1.82053
C -2.41010 -1.97372 1.25358
C -3.00043 -2.14662 -0.05226
Rh -1.75088 -0.23208 -0.04741
B 0.38081 -0.79194 -0.10190
P 2.06663 -0.11416 -0.70219
C 3.38285 -1.37113 -0.12670
C -4.72433 1.03107 1.12757
C -2.67857 -0.23753 3.21617
C -1.54961 -2.97075 1.97313
C -2.81179 -3.33350 -0.95093
C -4.78846 -0.88431 -1.49143
P -1.70748 1.81274 -1.18792
C -1.40246 3.31308 -0.12759
C -0.44602 1.94848 -2.54578
C -3.24943 2.30622 -2.10986
C 2.39885 1.58356 0.11639
H -1.80648 -3.76968 -0.84649
H -2.96733 -3.07530 -2.00948
H -3.54280 -4.12150 -0.69035
H -5.68843 -1.50327 -1.31758
H -4.32513 -1.23511 -2.42713
H -5.13011 0.14941 -1.64557
H -5.11093 1.48609 0.20336
H -4.22804 1.81485 1.72116
H -5.60219 0.69021 1.70738
H -3.40938 -0.69403 3.91043
H -2.79052 0.85479 3.29246
H -1.67332 -0.50627 3.57453
H -2.18365 -3.66627 2.55325
H -0.85832 -2.48252 2.67725
H -0.94983 -3.57185 1.27323
H 0.44913 -1.93770 0.26641
H -0.20136 0.16356 0.67547
H -0.44167 2.97106 -2.95704
H -0.71033 1.23420 -3.34137
H 0.55834 1.68995 -2.17781
H -3.07342 3.26799 -2.61926
H -4.09390 2.42316 -1.41552
H -3.49809 1.54201 -2.86154
H -0.44060 3.20968 0.39644
H -2.20324 3.39702 0.62331
H -1.38736 4.22499 -0.74718
H -0.56533 -0.74854 -1.16161
C 4.82143 -0.88994 -0.42449
H 3.26484 -1.49920 0.96752
C 5.86156 -1.94791 0.00554
H 4.92095 -0.69417 -1.51054
H 5.03233 0.06323 0.09048
C 5.59889 -3.31021 -0.65985
H 6.87646 -1.58582 -0.23767
H 5.82630 -2.06368 1.10697
C 4.16031 -3.78717 -0.38997
H 5.75387 -3.21958 -1.75300
H 6.32667 -4.05928 -0.30091
C 3.12379 -2.72840 -0.82291
H 3.96004 -4.73706 -0.91749
H 4.03762 -3.99610 0.69132
H 3.18388 -2.58394 -1.92013
H 2.10300 -3.08925 -0.60632
C 3.38122 2.46084 -0.69422
H 1.40068 2.06681 0.07380
C 3.54651 3.85491 -0.05226
H 4.36779 1.96403 -0.74270
H 3.03401 2.55470 -1.73908
C 3.97195 3.75200 1.42382
H 4.28067 4.44531 -0.62867
H 2.58501 4.40363 -0.11774
C 2.98058 2.89160 2.22798
H 4.98097 3.29883 1.48262
H 4.05534 4.75824 1.87105
C 2.80527 1.48767 1.60580
H 3.31285 2.79113 3.27697
H 1.99632 3.40157 2.25987
H 3.75944 0.93534 1.69501
H 2.05115 0.90672 2.16762

TS(11′′-6)4$_{Cy}$
SCF Energy = -1130.71540820
Enthalpy 0K = -1130.044491
Enthalpy 298K = -1130.004783
Free Energy 298K = -1130.115692
SCF(DCM) = -1130.76289508
SCF(BP86-D3) = -0.11757248
Lowest Frequencies = -133.5210
11.3184 cm-1
C -3.84697 -0.47552 0.47314
C -3.68699 0.54554 -0.57044
C -3.03484 1.67669 0.02737
C -2.80085 1.38361 1.44076

C -3.30829 0.06608 1.71489
Rh -1.68531 -0.16442 0.17639
B 0.34514 -0.03574 1.48035
P 1.73112 0.00891 0.17475
C 3.36969 -0.88035 0.55246
C -4.30261 0.52343 -1.94212
C -2.72276 2.97634 -0.65174
C -2.25656 2.34639 2.45488
C -3.34165 -0.60413 3.05685
C -4.66960 -1.72534 0.37232
P -1.06749 -1.71762 -1.47170
C -0.24722 -1.03147 -2.99734
C 0.06908 -3.08380 -0.92170
C -2.44391 -2.73001 -2.22122
C 2.12168 1.80165 -0.30849
H -2.45412 -0.35019 3.65770
H -3.38952 -1.69982 2.96472
H -4.23238 -0.27650 3.62468
H -5.69142 -1.52441 0.74698
H -4.25416 -2.54231 0.98345
H -4.76731 -2.07821 -0.66420
H -4.51369 -0.49946 -2.28656
H -3.66284 1.01523 -2.69191
H -5.26732 1.06372 -1.92873
H -3.55248 3.69249 -0.49629
H -2.59516 2.85092 -1.73791
H -1.81156 3.44303 -0.24638
H -3.08303 2.93891 2.88928
H -1.54102 3.05450 2.00897
H -1.74776 1.82564 3.28009
H 0.48823 -0.44905 2.60776
H -0.13014 1.08655 1.33924
H 0.32249 -3.73246 -1.77581
H -0.43828 -3.67810 -0.14603
H 0.99003 -2.65571 -0.50136
H -2.01745 -3.43954 -2.94921
H -3.16343 -2.08162 -2.74207
H -2.96375 -3.29601 -1.43422
H 0.70217 -0.56480 -2.69591
H -0.89537 -0.26516 -3.45088
H -0.06261 -1.83523 -3.72982
H -0.60790 -1.02631 1.15292
C 3.95577 -1.62380 -0.67328
H 4.06363 -0.05628 0.81939
C 5.31122 -2.27686 -0.33021
H 3.24510 -2.41157 -0.99341
H 4.06548 -0.93539 -1.52990
C 5.19116 -3.21874 0.88196
H 5.69517 -2.82180 -1.21098
H 6.05038 -1.48231 -0.10704
C 4.59769 -2.48602 2.09904
H 4.54002 -4.07528 0.61619
H 6.17795 -3.64404 1.13600
C 3.24170 -1.82305 1.76862
H 4.46936 -3.18097 2.94806
H 5.30624 -1.70519 2.43862
H 2.49638 -2.61342 1.54596
H 2.85990 -1.27191 2.64472
C 2.97840 1.91265 -1.58961
H 1.11589 2.21862 -0.51832
C 3.20950 3.38819 -1.98219
H 3.96097 1.43196 -1.41556
H 2.50101 1.36418 -2.42218
C 3.83731 4.18884 -0.82677
H 3.84837 3.43695 -2.88181
H 2.23909 3.84519 -2.26141
C 2.98639 4.07788 0.45081
H 4.85506 3.80123 -0.62364
H 3.95849 5.24767 -1.11576
C 2.75110 2.60527 0.85708
H 3.46602 4.61648 1.28766
H 2.00662 4.56815 0.28279
H 3.72179 2.15117 1.13648
H 2.10474 2.54799 1.75021

TS(11''-9')2_{Cy}
SCF Energy = -1130.72886015
Enthalpy 0K = -1130.055086
Enthalpy 298K = -1130.016465
Free Energy 298K = -1130.122235
SCF(DCM) = -1130.77528615
SCF(BP86-D3) = -0.12789587
Lowest Frequencies = -116.1963
20.1801 cm-1
C 1.45072 1.85345 1.39686
C 2.34378 1.96928 0.28825
C 3.31815 0.88691 0.38362
C 3.01789 0.08433 1.53093
C 1.81295 0.64267 2.14904
Rh 1.13777 -0.10057 0.25626
P -1.09347 -0.06109 -0.25866
C -2.14502 -1.47504 0.43278
C 2.43351 3.09975 -0.69961
C 4.54708 0.81100 -0.47668
C 3.87249 -1.00065 2.12850
C 1.27435 0.26361 3.50041
C 0.44905 2.87247 1.85724
P 1.39049 -1.92777 -1.88205
C 3.17234 -2.20343 -1.38738
B -0.18690 -0.52097 -1.91981
C 0.65177 -3.57729 -1.45285
C 1.61234 -1.98847 -3.73847
C -2.04179 1.58436 -0.32483
H 1.38950 -0.81349 3.69467
H 0.20686 0.51354 3.59928
H 1.81976 0.80865 4.29424
H 0.94108 3.58108 2.54997
H -0.38579 2.41191 2.40801
H 0.03559 3.46260 1.02560
H 1.54199 3.74392 -0.67077
H 2.55622 2.73832 -1.73409
H 3.30607 3.74109 -0.47359
H 5.23103 1.63797 -0.20706
H 4.31807 0.93366 -1.54885
H 5.10434 -0.12672 -0.33620
H 4.54295 -0.57548 2.89847
H 4.51304 -1.48842 1.37711
H 3.26535 -1.77965 2.61512
H 0.10040 0.37206 -2.67121
H 0.78845 -1.53906 0.80408
H 1.27208 -4.37268 -1.89560
H -0.37234 -3.65059 -1.84587

H 0.63692 -3.69597 -0.35928
H 2.23691 -2.85378 -4.01196
H 2.10918 -1.06366 -4.07111
H 0.63234 -2.05825 -4.23111
H 3.23676 -2.34932 -0.30174
H 3.78101 -1.34032 -1.68613
H 3.53783 -3.10109 -1.91501
H -0.78146 -1.45286 -2.42056
C -3.27805 -1.99014 -0.48591
H -1.37801 -2.27231 0.50132
C -3.93568 -3.24492 0.13110
H -4.04924 -1.21165 -0.61780
H -2.88356 -2.21687 -1.49280
C -4.45265 -2.96860 1.55532
H -4.75818 -3.58760 -0.52132
H -3.19545 -4.06919 0.16284
C -3.32779 -2.44312 2.46613
H -5.26778 -2.21970 1.50927
H -4.89463 -3.88410 1.98595
C -2.64472 -1.19258 1.86930
H -3.71950 -2.20642 3.47126
H -2.56685 -3.23672 2.60446
H -3.36910 -0.35538 1.85826
H -1.79960 -0.87834 2.50894
C -3.55751 1.45312 -0.61221
H -1.92421 2.01019 0.69162
C -4.23545 2.84237 -0.57945
H -3.70545 0.99322 -1.60807
H -4.04466 0.79481 0.12573
C -3.57638 3.83136 -1.55634
H -5.30955 2.72689 -0.80861
H -4.17740 3.24694 0.45040
C -2.06246 3.93749 -1.29777
H -3.74392 3.48742 -2.59575
H -4.05168 4.82439 -1.47403
C -1.39148 2.54845 -1.34305
H -1.58714 4.60129 -2.04184
H -1.89136 4.40217 -0.30574
H -1.50663 2.12364 -2.35747
H -0.30547 2.62907 -1.16474

$9'_{Cy}$
SCF Energy = -1130.74658001
Enthalpy 0K = -1130.073670
Enthalpy 298K = -1130.033758
Free Energy 298K = -1130.143868
SCF(DCM) = -1130.79494368
SCF(BP86-D3) = -0.12384370
Lowest Frequencies = 17.0360 28.2712 cm-1
C 2.24496 0.81726 1.87734
C 2.36402 1.73916 0.72061
C 3.09430 1.06387 -0.28467
C 3.51457 -0.25069 0.25329
C 3.06403 -0.37036 1.59996
Rh 1.23945 -0.22551 0.32798
P -0.96104 0.03067 -0.37248
C -2.30547 -0.53156 0.85196
C 1.91191 3.16915 0.71838
C 3.49045 1.58739 -1.63437
C 4.37947 -1.22112 -0.49455
C 3.40247 -1.45618 2.58292
C 1.67213 1.18359 3.21208
B -1.02999 -1.25673 -1.91282
P -0.57280 -3.13781 -1.66414
C 1.24151 -3.46881 -1.53591
C -1.32960 -4.06303 -0.25068
C -1.11863 -4.05313 -3.17694
C -1.57755 1.63802 -1.18583
H 3.62608 -2.40944 2.07952
H 2.57934 -1.63556 3.29122
H 4.29467 -1.17277 3.17119
H 2.42123 1.75261 3.79674
H 1.40241 0.29079 3.79628
H 0.77713 1.81708 3.11355
H 1.01802 3.31624 1.34300
H 1.68782 3.53535 -0.29501
H 2.71110 3.81142 1.13501
H 4.55851 1.87545 -1.63826
H 2.90520 2.47539 -1.91632
H 3.35727 0.82852 -2.42306
H 5.43364 -0.88568 -0.46744
H 4.09319 -1.28864 -1.55686
H 4.34386 -2.23005 -0.05655
H -0.28213 -0.91524 -2.80758
H 0.53192 -1.44171 1.06503
H -1.08639 -5.13461 -0.32815
H -2.42305 -3.93753 -0.27044
H -0.93491 -3.66802 0.69822
H -0.81057 -5.10911 -3.12517
H -0.67087 -3.57908 -4.06377
H -2.21431 -3.99072 -3.26086
H 1.62688 -2.93933 -0.64713
H 1.73732 -3.07386 -2.43626
H 1.44098 -4.54850 -1.44819
H -2.19223 -1.26335 -2.27995
C -0.60151 2.10279 -2.28996
H -2.52827 1.33388 -1.66947
C -1.11751 3.36822 -3.00752
H 0.38351 2.31439 -1.82773
H -0.43725 1.29586 -3.02396
C -1.41138 4.50711 -2.01612
H -0.38165 3.69039 -3.76605
H -2.04415 3.11632 -3.55941
C -2.38697 4.04336 -0.92027
H -0.46433 4.84230 -1.54673
H -1.82136 5.38402 -2.54734
C -1.87450 2.78160 -0.18989
H -2.56115 4.84937 -0.18514
H -3.37086 3.82058 -1.37792
H -0.95002 3.02922 0.36532
H -2.61764 2.46262 0.56077
C -3.74878 -0.36898 0.32391
H -2.09075 -1.61564 0.93705
C -4.76149 -0.99864 1.30607
H -3.98605 0.70625 0.21365
H -3.85256 -0.82189 -0.67814
C -4.61756 -0.40727 2.72028
H -5.78782 -0.85117 0.92570
H -4.59687 -2.09401 1.34800
C -3.17538 -0.55217 3.24161
H -4.89181 0.66586 2.69582

```
H  -5.32597 -0.89450  3.41318
C  -2.15090  0.06911  2.26765
H  -3.07414 -0.08440  4.23730
H  -2.94126 -1.62744  3.37539
H  -2.30425  1.16422  2.23020
H  -1.12076 -0.09918  2.62885
```

TS(9'-9)$_{Cy}$
SCF Energy = -1130.74552875
Enthalpy 0K = -1130.072249
Enthalpy 298K = -1130.033230
Free Energy 298K = -1130.140632
SCF(DCM) = -1130.79289180
SCF(BP86-D3) = -0.11984307
Lowest Frequencies = -104.5029
26.0772 cm-1

```
C  -2.98285  1.27591  0.19770
C  -2.99738  0.12661  1.12772
C  -3.32734 -1.02219  0.37074
C  -3.61797 -0.60749 -1.02151
C  -3.48574  0.81052 -1.10737
Rh -1.48400  0.02400 -0.63207
P   0.75855  0.15533 -0.06605
C   1.81930  1.50260 -0.87797
C  -2.82979  0.22243  2.61646
C  -3.45418 -2.43273  0.86805
C  -4.09001 -1.53572 -2.10296
C  -3.84783  1.68882 -2.27231
C  -2.81778  2.70871  0.60804
B   0.82664 -1.51590 -1.14501
P   2.42208 -2.62603 -1.31708
C   3.68421 -1.92465 -2.47424
C   3.35741 -3.07723  0.21523
C   1.93788 -4.24088 -2.07904
C   1.38857 -0.07992  1.70890
H  -3.74712  1.15756 -3.23088
H  -3.21380  2.58727 -2.31768
H  -4.89771  2.02335 -2.18034
H  -3.76914  3.09873  1.01893
H  -2.53663  3.34408 -0.24515
H  -2.04702  2.81812  1.38674
H  -2.07863  0.97541  2.90074
H  -2.53694 -0.73994  3.06384
H  -3.78632  0.52424  3.08393
H  -4.51925 -2.70182  0.99731
H  -2.95467 -2.57255  1.83875
H  -3.02428 -3.15448  0.15399
H  -5.16113 -1.77662 -1.96546
H  -3.53742 -2.48918 -2.09055
H  -3.97455 -1.08867 -3.10190
H   0.62805 -1.22337 -2.31033
H  -0.88498  0.82173 -1.85391
H   4.21886 -3.71454 -0.03944
H   2.69363 -3.62078  0.90453
H   3.71281 -2.16083  0.71036
H   2.82465 -4.86556 -2.26940
H   1.41465 -4.04424 -3.02734
H   1.25195 -4.76940 -1.39962
H   4.08193 -0.98060 -2.07255
H   3.20079 -1.72181 -3.44220
H   4.51138 -2.63750 -2.61773
H  -0.03085 -2.28334 -0.72473
C   0.74688 -1.31857  2.37164
H   2.47973 -0.25977  1.61210
C   1.28539 -1.53504  3.80222
H  -0.35018 -1.17682  2.40392
H   0.91916 -2.22052  1.75749
C   1.10306 -0.28046  4.67637
H   0.78272 -2.40495  4.26204
H   2.36300 -1.78880  3.75091
C   1.73757  0.95552  4.01400
H   0.02153 -0.09708  4.83226
H   1.53837 -0.44576  5.67749
C   1.19298  1.17893  2.58695
H   1.56037  1.85827  4.62511
H   2.83650  0.82283  3.96504
H   0.11350  1.42165  2.63807
H   1.68687  2.05401  2.12996
C   3.30411  1.49126 -0.44913
H   1.75808  1.21532 -1.94754
C   4.12463  2.50148 -1.28146
H   3.38351  1.76526  0.62038
H   3.73796  0.47706 -0.54101
C   3.53590  3.92064 -1.17565
H   5.17742  2.49265 -0.94693
H   4.12692  2.18240 -2.34270
C   2.04713  3.93949 -1.56973
H   3.64355  4.28151 -0.13369
H   4.11022  4.61916 -1.80908
C   1.22536  2.92500 -0.74628
H   1.62637  4.95250 -1.44017
H   1.94847  3.69420 -2.64591
H   1.22565  3.23418  0.31708
H   0.17227  2.91926 -1.07764
```

9$_{Cy}$
SCF Energy = -1130.76599419
Enthalpy 0K = -1130.092447
Enthalpy 298K = -1130.052954
Free Energy 298K = -1130.161515
SCF(DCM) = -1130.81231420
SCF(BP86-D3) = -0.12191109
Lowest Frequencies = 19.4915
31.9060 cm-1

```
C   2.48685  0.54804  1.89281
C   2.44364  1.63070  0.90564
C   3.12633  1.19118 -0.27337
C   3.63668 -0.15805 -0.01919
C   3.28548 -0.53562  1.32236
Rh  1.36827 -0.26877  0.20814
P  -0.88229  0.15980 -0.07956
C  -1.45234  1.92678 -0.48315
C   1.89643  3.00597  1.16720
C   3.41881  2.00138 -1.50636
C   4.51116 -0.94055 -0.95922
C   3.74748 -1.76763  2.05138
C   2.05607  0.65707  3.32977
B  -0.15606 -0.82667 -1.59760
P  -0.67897 -2.65627 -2.04628
C   0.61860 -3.42643 -3.11631
C  -0.89290 -3.82629 -0.63283
C  -2.23049 -2.72442 -3.04861
```

```
C  -2.07470 -0.67340  1.13889
H   1.83570 -0.33074  3.76250
H   1.15552  1.28153  3.43802
H   2.85545  1.11846  3.94004
H   2.66914  3.62993  1.65431
H   1.02673  2.98209  1.84279
H   1.59943  3.51967  0.24016
H   2.72050  2.84502 -1.61992
H   3.36236  1.39136 -2.42239
H   4.43929  2.42540 -1.45799
H   5.56724 -0.63274 -0.84442
H   4.23504 -0.77171 -2.01209
H   4.46013 -2.02196 -0.75820
H   4.71115 -1.57168  2.55708
H   3.89897 -2.61509  1.36497
H   3.02506 -2.08074  2.82030
H   1.12750 -1.07998 -1.32595
H   0.78032 -1.55524  0.91751
H  -2.46606 -3.76490 -3.32111
H  -2.08851 -2.12758 -3.96278
H  -3.06418 -2.29608 -2.47241
H   0.30752 -4.43281 -3.43731
H   1.55841 -3.49596 -2.54730
H   0.78185 -2.79119 -4.00042
H  -1.79275 -3.55776 -0.05959
H  -0.01202 -3.74070  0.02267
H  -0.98889 -4.85940 -1.00155
H  -0.15742 -0.21986 -2.64394
C  -2.96692  2.09471 -0.74613
H  -1.19494  2.49258  0.43600
C  -3.32044  3.57921 -0.99479
H  -3.25525  1.49058 -1.62862
H  -3.55700  1.72202  0.10738
C  -2.48687  4.19291 -2.13313
H  -4.39979  3.66554 -1.21271
H  -3.14432  4.15026 -0.06183
C  -0.98046  4.00505 -1.87725
H  -2.75588  3.70455 -3.09045
H  -2.72844  5.26398 -2.24878
C  -0.63624  2.51731 -1.65526
H  -0.39122  4.39838 -2.72483
H  -0.68169  4.59158 -0.98557
H  -0.87077  1.95363 -2.57786
H   0.44514  2.38428 -1.47700
C  -3.44733 -1.13093  0.59302
H  -1.49230 -1.57540  1.41211
C  -4.23358 -1.89880  1.67981
H  -4.04287 -0.26073  0.26622
H  -3.31605 -1.76850 -0.30086
C  -4.40215 -1.05415  2.95649
H  -5.21794 -2.20147  1.28061
H  -3.69376 -2.83431  1.93046
C  -3.03958 -0.58196  3.49551
H  -5.03393 -0.17259  2.73050
H  -4.93916 -1.63318  3.72792
C  -2.23410  0.18007  2.42077
H  -3.17532  0.06229  4.38215
H  -2.45229 -1.45927  3.83144
H  -2.75988  1.12372  2.17751
H  -1.23937  0.45678  2.81410
```

$H_2BP^tBu_2$

```
SCF Energy = -348.292407166
Enthalpy 0K = -348.032539
Enthalpy 298K = -348.016916
Free Energy 298K = -348.071547
SCF(DCM) = -348.111651907
SCF(BP86-D3) = -0.03465588
Lowest Frequencies = 56.7318
76.7858 cm-1
C   1.62904 -0.24656  0.00773
P  -0.00233  0.70846 -0.37423
C  -1.63937 -0.23183  0.00806
B   0.04860  2.48537  0.06830
H  -0.96801  3.12396  0.11785
H   1.11716  3.03827  0.11153
C   1.55626 -1.69727 -0.51331
C   2.73757  0.50504 -0.76480
C   1.95625 -0.23554  1.51707
C  -1.91250 -1.26373 -1.11079
C  -1.60433 -0.93011  1.38319
C  -2.76595  0.82377 -0.00394
H   3.71117  0.01803 -0.56685
H   2.55965  0.48640 -1.85290
H   2.81691  1.55750 -0.44904
H   2.54157 -2.18219 -0.37820
H   0.81846 -2.30648  0.03503
H   1.30918 -1.73520 -1.58767
H   2.91686 -0.75747  1.69569
H   2.05100  0.79571  1.89301
H   1.18358 -0.74548  2.11414
H  -2.88137 -1.76451 -0.92337
H  -1.96596 -0.77767 -2.09912
H  -1.13868 -2.04601 -1.15790
H  -2.57810 -1.42198  1.57257
H  -0.82965 -1.71274  1.43241
H  -1.42111 -0.21068  2.19789
H  -3.73949  0.30767  0.09352
H  -2.66985  1.53741  0.82918
H  -2.78317  1.39899 -0.94506
```

10_{Me}

```
SCF Energy = -780.538917299
Enthalpy 0K = -780.059700
Enthalpy 298K = -780.025917
Free Energy 298K = -780.122313
SCF(DCM) = -780.587899821
SCF(BP86-D3) = -0.08636568
Lowest Frequencies = 22.4371
29.9027 cm-1
C  -2.38129 -0.90854 -0.32157
C  -2.13757 -0.63393  1.10392
C  -1.03344 -1.42741  1.53034
C  -0.60480 -2.24471  0.38595
C  -1.46838 -1.97142 -0.72449
Rh -0.36861 -0.04982 -0.24487
P  -0.56530  2.25734 -0.06692
C  -0.59354  2.92170  1.67230
C  -3.02045  0.21052  1.97867
C  -0.48609 -1.54209  2.92442
C   0.44854 -3.31259  0.45543
C  -1.50425 -2.69783 -2.03772
```

C -3.56957 -0.44988 -1.11891
C -0.04502 0.28681 -2.31867
B 2.03782 0.15872 0.71424
P 3.80808 0.16535 -0.13490
C 4.19488 -1.27799 -1.22466
C 5.17535 0.28220 1.10405
C 0.78012 3.28703 -0.84107
C -2.09378 3.00432 -0.82298
H -0.54536 -3.19083 -2.26216
H -1.74381 -2.02429 -2.87601
H -2.28162 -3.48341 -2.01418
H -4.38836 -1.19037 -1.04949
H -3.32326 -0.33370 -2.18655
H -3.96609 0.50987 -0.75420
H -3.41272 1.09526 1.45177
H -2.50079 0.54760 2.88883
H -3.89678 -0.38108 2.30321
H -0.89699 -2.43495 3.43145
H -0.74324 -0.66467 3.53797
H 0.61140 -1.63870 2.92304
H 0.04082 -4.21276 0.95196
H 1.32092 -2.98278 1.04144
H 0.79394 -3.61555 -0.54489
H 2.05995 -0.83190 1.40889
H 4.04249 1.28890 -0.99099
H 0.61515 4.35176 -0.60965
H 0.77907 3.15126 -1.93245
H 1.75380 2.97444 -0.43561
H -2.08538 4.10056 -0.70770
H -2.99088 2.60000 -0.33015
H -2.13432 2.75010 -1.89305
H 0.34281 2.63793 2.17563
H -1.43808 2.48528 2.22418
H -0.69288 4.01956 1.66322
H -0.97775 0.63330 -2.79594
H 0.75095 1.01791 -2.53384
H 0.24560 -0.67704 -2.76779
H 1.36963 0.04381 -0.39048
H 1.98257 1.20749 1.31575
H 6.15482 0.31815 0.60398
H 5.03291 1.18926 1.71054
H 5.12780 -0.59696 1.76508
H 5.20690 -1.19158 -1.64796
H 4.12283 -2.20122 -0.62904
H 3.45639 -1.32010 -2.03964

TS(10-11’’)$_{Me}$
SCF Energy = -780.513033770
Enthalpy 0K = -780.036051
Enthalpy 298K = -780.002847
Free Energy 298K = -780.097254
SCF(DCM) = -780.563801379
SCF(BP86-D3) = -0.08932233
Lowest Frequencies = -905.8513
10.4611 cm-1
C -2.33019 -1.09196 -0.43854
C -2.19053 -0.60068 0.91770
C -1.06351 -1.27192 1.54495
C -0.50473 -2.18518 0.57088
C -1.27946 -2.06295 -0.64817
Rh -0.28413 -0.03571 -0.20662
P -0.54117 2.26691 -0.10582
C -0.67394 3.03145 1.58482
C -3.18948 0.26079 1.63807
C -0.67677 -1.19366 2.99427
C 0.56102 -3.20779 0.84807
C -1.09942 -2.89077 -1.88856
C -3.44405 -0.77816 -1.39944
C 0.48782 0.13542 -2.31925
B 1.73732 0.30446 0.76016
P 3.48813 0.14530 -0.13047
C 3.90513 -1.45495 -0.96762
C 4.85450 0.44516 1.08361
C 0.79445 3.29313 -0.90274
C -2.05863 2.92249 -0.97189
H -0.05559 -3.21799 -2.02060
H -1.40794 -2.34701 -2.79598
H -1.72071 -3.80331 -1.82943
H -4.24939 -1.53312 -1.33167
H -3.09422 -0.77193 -2.44520
H -3.89990 0.20289 -1.19355
H -3.67344 0.99170 0.97209
H -2.74013 0.80105 2.48537
H -3.99215 -0.37753 2.05204
H -1.15760 -2.00847 3.56688
H -0.98544 -0.24102 3.45174
H 0.41244 -1.29029 3.12620
H 0.12180 -4.09064 1.34865
H 1.34192 -2.80765 1.51422
H 1.04196 -3.56549 -0.07609
H 1.84582 -0.59268 1.57353
H 3.77130 1.11087 -1.15390
H 0.56211 4.36405 -0.78729
H 0.85837 3.05688 -1.97581
H 1.75980 3.07802 -0.42186
H -2.10033 4.02206 -0.90250
H -2.96589 2.50086 -0.51397
H -2.03066 2.62711 -2.03245
H 0.22061 2.77055 2.16922
H -1.56435 2.63863 2.09750
H -0.75968 4.12743 1.50381
H -0.44563 0.51443 -2.75893
H 1.29705 0.81651 -2.63311
H 0.69659 -0.87197 -2.70587
H 1.12006 0.11283 -0.99376
H 1.84905 1.41006 1.25938
H 5.83734 0.38919 0.59159
H 4.71850 1.43876 1.53607
H 4.79038 -0.31557 1.87696
H 4.94068 -1.44648 -1.33962
H 3.78248 -2.27181 -0.23977
H 3.21638 -1.62191 -1.80964

INT(10-11’’)$_{Me}$
SCF Energy = -780.528425879
Enthalpy 0K = -780.048829
Enthalpy 298K = -780.014381
Free Energy 298K = -780.111837
SCF(DCM) = -780.587899821
SCF(BP86-D3) = -0.08911780
Lowest Frequencies = 19.8174
30.7325 cm-1

C -2.01524 -1.54844 -0.60943
C -2.31143 -0.79712 0.54929
C -1.19690 -0.98430 1.51613
C -0.29492 -1.99404 0.96270
C -0.73882 -2.25202 -0.37076
Rh -0.24956 -0.01468 -0.14627
P -0.64936 2.27179 -0.10113
C -0.81607 3.09129 1.55959
C -3.58328 -0.05882 0.85054
C -1.21750 -0.54623 2.95137
C 0.77314 -2.72617 1.72110
C -0.13617 -3.23402 -1.33411
C -2.85702 -1.69966 -1.84458
C 0.54639 0.17860 -2.83726
B 1.61494 0.58198 0.86950
P 3.27281 0.20473 -0.10271
C 3.64472 -1.52311 -0.66844
C 4.78924 0.72908 0.83290
C 0.57160 3.36745 -0.98779
C -2.23426 2.78338 -0.95130
H 0.92603 -3.42764 -1.11927
H -0.22657 -2.89700 -2.37967
H -0.66657 -4.20248 -1.26544
H -3.41063 -2.65702 -1.82846
H -2.24567 -1.70139 -2.76276
H -3.59992 -0.89193 -1.93572
H -4.12238 0.23209 -0.06442
H -3.40953 0.84464 1.45687
H -4.26054 -0.70793 1.43695
H -1.71002 -1.31631 3.57553
H -1.77696 0.39218 3.08769
H -0.19819 -0.39792 3.33874
H 0.31604 -3.52413 2.33566
H 1.32566 -2.05725 2.39857
H 1.49735 -3.20991 1.04720
H 1.74221 -0.05978 1.89815
H 3.40601 0.94483 -1.32457
H 0.24397 4.41753 -0.91991
H 0.62111 3.07775 -2.04858
H 1.56603 3.26339 -0.53207
H -2.36327 3.87697 -0.89393
H -3.09619 2.29441 -0.47509
H -2.19807 2.48172 -2.01001
H 0.10318 2.92660 2.14020
H -1.66721 2.65240 2.10199
H -0.99116 4.17232 1.43446
H -0.52930 0.37755 -2.91947
H 1.11588 0.99844 -3.29822
H 0.78994 -0.77770 -3.32158
H 0.91052 0.10148 -1.76573
H 1.79124 1.77120 1.09418
H 5.69928 0.57965 0.23187
H 4.69035 1.79073 1.10417
H 4.85273 0.13470 1.75764
H 4.60667 -1.56230 -1.20152
H 3.68979 -2.18090 0.21310
H 2.84101 -1.87115 -1.33437

TS(10-11’’)2_{Me}
SCF Energy = -780.528286083
Enthalpy 0K = -780.049123
Enthalpy 298K = -780.015234
Free Energy 298K = -780.110929
SCF(DCM) = -780.577784361
SCF(BP86-D3) = -0.08816918
Lowest Frequencies = -59.4659
25.9429 cm-1
C 2.37538 -0.71461 -0.46572
C 1.32287 -0.96784 -1.48857
C 0.43633 -2.01097 -0.96876
C 0.81316 -2.21918 0.39220
C 2.04490 -1.45596 0.68746
Rh 0.24058 -0.00813 0.08002
P 0.52184 2.29512 0.07658
C -0.81269 3.30205 0.90161
C 1.39458 -0.53749 -2.92336
C -0.55560 -2.80130 -1.77036
C 0.19900 -3.20085 1.34747
C 2.81781 -1.54643 1.97230
C 3.62720 0.07633 -0.71084
B -1.60913 0.46888 -0.98328
P -3.23153 0.09835 0.04483
C -4.78145 0.55144 -0.87434
C -3.54157 -1.62202 0.66471
C 2.03096 2.88580 1.00754
C 0.72861 3.13294 -1.57035
C -0.68463 0.19657 3.05981
H -0.81864 -3.49223 1.04625
H 0.16207 -2.80662 2.37618
H 0.80951 -4.12290 1.38196
H 3.41258 -2.47839 2.00866
H 2.15396 -1.55596 2.85259
H 3.51818 -0.70472 2.08754
H 4.10595 0.39473 0.22813
H 3.44622 0.96882 -1.33150
H 4.36063 -0.54557 -1.25787
H 1.97660 -1.27413 -3.51040
H 1.89358 0.43766 -3.03457
H 0.39130 -0.46532 -3.36963
H -0.03534 -3.60675 -2.32151
H -1.07860 -2.17334 -2.50754
H -1.31319 -3.27946 -1.13007
H -1.73288 -0.25567 -1.95605
H -3.35834 0.87375 1.24445
H -0.54847 4.37153 0.87002
H -0.91029 2.98753 1.95203
H -1.76791 3.14522 0.38145
H 2.09010 3.98649 0.98325
H 2.94114 2.46787 0.55349
H 1.97225 2.55105 2.05489
H -0.15132 2.92530 -2.19601
H 1.62630 2.73839 -2.07081
H 0.84361 4.22057 -1.43318
H 0.28639 0.66211 3.27269
H -1.50335 0.87606 3.33567
H -0.77670 -0.74971 3.61274
H -0.77217 -0.04428 1.96691
H -1.81839 1.63393 -1.29138
H -5.67485 0.39402 -0.25074
H -4.71884 1.60675 -1.17903
H -4.84522 -0.07183 -1.77988
H -4.49873 -1.67845 1.20481

H -3.56929 -2.30515 -0.19833
H -2.72231 -1.92278 1.33418

$11''_{Me}$
SCF Energy = -740.009640582
Enthalpy 0K = -739.576267
Enthalpy 298K = -739.545153
Free Energy 298K = -739.635859
SCF(DCM) = -740.061246888
SCF(BP86-D3) = -0.07397428
Lowest Frequencies = 23.0288
33.0444 cm-1
C -1.14448 -2.07790 -0.24482
C -1.92963 -1.16696 -1.12122
C -2.59388 -0.20287 -0.32188
C -2.09976 -0.38773 1.04199
C -1.30965 -1.64200 1.09743
Rh -0.24392 -0.03746 0.00589
P 0.26257 2.24689 -0.10836
C 1.45564 3.01212 1.09776
C -2.04343 -1.33559 -2.60570
C -3.62867 0.79138 -0.76822
C -2.55608 0.36847 2.25105
C -0.89180 -2.34247 2.35455
C -0.44634 -3.32229 -0.70988
B 1.61451 -0.47996 1.00904
P 3.21581 -0.38957 -0.10220
C 4.76101 -0.73420 0.86988
C 3.30178 -1.54154 -1.55261
C 0.96533 2.75938 -1.76462
C -1.19343 3.40270 0.04033
H -1.06114 -1.53434 -3.06662
H -2.48277 -0.45108 -3.09121
H -2.68760 -2.20329 -2.84362
H -4.63483 0.33297 -0.74527
H -3.45611 1.14070 -1.79921
H -3.66490 1.67290 -0.10992
H -2.84445 1.40248 2.00677
H -1.77578 0.39278 3.02724
H -3.44530 -0.12609 2.68838
H -1.74744 -2.91253 2.76306
H -0.55990 -1.63279 3.12805
H -0.06947 -3.04949 2.17344
H -1.16038 -4.16444 -0.78011
H 0.35202 -3.61828 -0.01201
H -0.00054 -3.19163 -1.70925
H 1.88265 0.27467 1.92945
H 1.64840 -1.65711 1.33698
H 3.52570 0.87696 -0.70484
H 1.12931 3.84949 -1.78675
H 0.26021 2.48110 -2.56293
H 1.92325 2.24923 -1.94714
H -0.86197 4.44847 -0.06907
H -1.66645 3.27935 1.02692
H -1.93053 3.17247 -0.74301
H 2.43627 2.52133 1.02015
H 1.07908 2.85943 2.12069
H 1.56322 4.09051 0.89789
H 4.28038 -1.47918 -2.05212
H 3.14286 -2.56804 -1.18680
H 2.50423 -1.29327 -2.26916
H 5.65438 -0.67949 0.22887
H 4.83912 -0.00088 1.68638
H 4.67717 -1.74042 1.30889

$TS(11''-11)_{Me}$
SCF Energy = -740.006403439
Enthalpy 0K = -739.573990
Enthalpy 298K = -739.543347
Free Energy 298K = -739.632858
SCF(DCM) = -740.056807168
SCF(BP86-D3) = -0.07343151
Lowest Frequencies = -133.4316
13.8407 cm-1
C -1.82471 -1.59697 -0.85935
C -2.57400 -0.31459 -0.85330
C -2.57347 0.17453 0.46624
C -1.76263 -0.75811 1.29136
C -1.43636 -1.92697 0.47733
Rh -0.32220 -0.08745 -0.15909
P 0.50708 2.08433 0.00911
C 1.18492 2.59397 1.66919
C -3.25560 0.26987 -2.05573
C -3.32647 1.35042 1.01443
C -1.60189 -0.66136 2.77785
C -0.88399 -3.23574 0.95877
C -1.65018 -2.45535 -2.07485
B 1.51897 -1.11770 0.15226
P 3.39805 -0.55393 0.07078
C 4.49680 -1.74129 0.97733
C 4.13288 -0.43710 -1.62892
C 1.85497 2.62758 -1.16704
C -0.75291 3.42325 -0.31629
H -2.61620 0.21627 -2.95250
H -3.53423 1.32307 -1.89816
H -4.18285 -0.28690 -2.28802
H -4.25634 1.00366 1.50323
H -3.61791 2.06245 0.22724
H -2.75138 1.89350 1.78244
H -1.54124 0.38479 3.11729
H -0.70100 -1.19162 3.12123
H -2.47582 -1.11973 3.28075
H -1.70430 -3.96355 1.09658
H -0.36430 -3.12708 1.92218
H -0.16630 -3.66621 0.24242
H -2.60261 -2.96435 -2.31696
H -0.88273 -3.22846 -1.92292
H -1.37224 -1.85599 -2.95785
H 1.49099 -1.77462 1.17661
H 1.44584 -1.81197 -0.86694
H 3.78796 0.69646 0.66562
H 2.02444 3.71251 -1.06783
H 1.55410 2.40272 -2.20222
H 2.79436 2.10206 -0.94520
H -0.28710 4.41720 -0.21275
H -1.58602 3.34169 0.39576
H -1.14715 3.31284 -1.33831
H 2.03240 1.94611 1.93790
H 0.39976 2.46749 2.43056
H 1.51145 3.64684 1.65264
H 5.19471 -0.15164 -1.58640
H 4.03658 -1.42597 -2.10421

H 3.57592 0.29364 -2.23390
H 5.55462 -1.44846 0.89635
H 4.19542 -1.76723 2.03538
H 4.35377 -2.74478 0.54716

11_{Me}
SCF Energy = -740.023642917
Enthalpy 0K = -739.590560
Enthalpy 298K = -739.559742
Free Energy 298K = -739.649451
SCF(DCM) = -740.074150640
SCF(BP86-D3) = -0.07322582
Lowest Frequencies = 22.2070
33.2834 cm-1
C -2.06674 -0.09105 1.13328
C -1.48043 -1.43303 1.16060
C -1.56563 -1.95730 -0.17202
C -2.29622 -0.98206 -1.00289
C -2.65427 0.12855 -0.19165
Rh -0.30093 -0.09842 -0.18640
P 0.49595 2.08099 -0.10289
C 1.55539 2.60385 -1.55070
C -0.99608 -2.15396 2.38684
C -1.16373 -3.33411 -0.61896
C -2.68665 -1.20473 -2.43558
C -3.56397 1.25873 -0.58469
C -2.32706 0.74842 2.35269
B 1.50611 -1.00951 -0.21508
P 3.39995 -0.53219 -0.10049
C 4.08685 -1.10536 1.51887
C 4.46526 -1.29913 -1.41148
C -0.77724 3.44358 -0.12668
C 1.51026 2.59982 1.37831
H -1.89992 -1.73198 -2.99779
H -2.90305 -0.25791 -2.95396
H -3.59935 -1.82788 -2.48987
H -4.61991 0.96253 -0.44023
H -3.44761 1.53587 -1.64481
H -3.39829 2.15918 0.02518
H -2.43204 1.81708 2.10840
H -1.52558 0.64129 3.09993
H -3.27221 0.43284 2.83389
H -1.84163 -2.66467 2.88521
H -0.54978 -1.46202 3.11792
H -0.24485 -2.91877 2.13938
H -2.01761 -4.03087 -0.53119
H -0.34214 -3.73723 -0.00703
H -0.83756 -3.34346 -1.67131
H 1.57133 -2.21638 -0.06410
H 3.77569 0.84867 -0.15223
H 1.91033 3.64002 -1.42395
H 0.95115 2.53425 -2.46879
H 2.42176 1.93535 -1.66217
H -0.27599 4.42504 -0.15846
H -1.39950 3.39408 0.77958
H -1.42134 3.33538 -1.01176
H 2.38938 1.94665 1.48053
H 0.89386 2.48511 2.28381
H 1.83857 3.64909 1.29206
H 0.97171 -0.58728 -1.36970
H 5.52731 -1.07270 -1.23026

H 4.30847 -2.38870 -1.39601
H 4.16549 -0.91089 -2.39673
H 5.16907 -0.91420 1.57153
H 3.57341 -0.58083 2.33861
H 3.89500 -2.18540 1.61290

$TS(11\text{-}11')_{Me}$
SCF Energy = -740.016305361
Enthalpy 0K = -739.585090
Enthalpy 298K = -739.554432
Free Energy 298K = -739.643879
SCF(DCM) = -740.067693598
SCF(BP86-D3) = -0.07342819
Lowest Frequencies = -351.3024
24.6601 cm-1
C -2.20212 -0.12158 1.08044
C -1.56750 -1.42128 1.17720
C -1.48865 -1.96717 -0.15706
C -2.14953 -1.02775 -1.07106
C -2.61424 0.08336 -0.30828
Rh -0.24410 -0.08855 -0.12221
P 0.47648 2.11066 -0.11012
C 1.40030 2.66789 -1.63284
C -1.17746 -2.12391 2.44831
C -1.03668 -3.35471 -0.52038
C -2.41887 -1.28148 -2.52852
C -3.50822 1.18275 -0.81257
C -2.61979 0.72523 2.25204
B 1.47858 -0.94317 0.24228
P 3.38153 -0.54124 -0.10491
C 4.40064 -0.79877 1.41854
C 4.11783 -1.61909 -1.42116
C -0.84383 3.42324 -0.01388
C 1.59846 2.66105 1.27976
H -1.60867 -1.85710 -3.00178
H -2.54114 -0.34297 -3.09116
H -3.35145 -1.86404 -2.64961
H -4.56222 0.84733 -0.80333
H -3.27013 1.46993 -1.84962
H -3.45575 2.08405 -0.18373
H -2.75136 1.78234 1.97264
H -1.88887 0.67698 3.07443
H -3.59020 0.37558 2.65196
H -2.02703 -2.71835 2.83318
H -0.88704 -1.41206 3.23665
H -0.33646 -2.81682 2.28979
H -1.88380 -4.06219 -0.45415
H -0.25030 -3.71949 0.15870
H -0.64698 -3.40127 -1.54951
H 1.55375 -2.08965 0.65307
H 3.72502 0.78158 -0.52911
H 1.71928 3.71938 -1.54331
H 0.73518 2.55694 -2.50319
H 2.28288 2.03135 -1.79692
H -0.38100 4.42249 -0.06617
H -1.39335 3.33331 0.93529
H -1.54817 3.30582 -0.85039
H 2.54773 2.10501 1.24135
H 1.10750 2.43967 2.23991
H 1.81173 3.74100 1.21339
H 0.72123 -0.31463 -1.39221

H   5.19292 -1.40988 -1.53194
H   3.97060 -2.67178 -1.13458
H   3.60086 -1.43082 -2.37420
H   5.46630 -0.63054 1.20187
H   4.06810 -0.10615 2.20636
H   4.25052 -1.83234 1.76681

$11'_{Me}$
SCF Energy = -740.017537343
Enthalpy 0K = -739.584878
Enthalpy 298K = -739.553982
Free Energy 298K = -739.644160
SCF(DCM) = -740.068765650
SCF(BP86-D3) = -0.07363429
Lowest Frequencies = 13.7699
34.0692 cm-1
C -2.22218 -0.14539 1.06758
C -1.54100 -1.40980 1.20591
C -1.40578 -1.98391 -0.11808
C -2.07398 -1.08893 -1.06559
C -2.58929 0.02573 -0.33722
Rh -0.20776 -0.07423 -0.11904
P   0.44267 2.14738 -0.13045
C   1.25839 2.74275 -1.69754
C -1.16849 -2.07976 2.49860
C -0.92170 -3.37422 -0.43097
C -2.30572 -1.38259 -2.52264
C -3.49851 1.08953 -0.89088
C -2.69492 0.71381 2.20889
B   1.46899 -0.83028 0.53654
P   3.32344 -0.56275 -0.10923
C   4.63547 -0.58186 1.19878
C   3.77840 -1.87672 -1.33449
C -0.91344 3.41207 0.06234
C   1.62769 2.71901 1.19480
H -1.47343 -1.95252 -2.96332
H -2.43309 -0.46031 -3.11045
H -3.22405 -1.98585 -2.64984
H -4.54067 0.72065 -0.92444
H -3.22436 1.37580 -1.91932
H -3.50299 1.99804 -0.26951
H -2.87157 1.75491 1.89664
H -1.97429 0.72356 3.04182
H -3.65354 0.33135 2.60675
H -1.98828 -2.73914 2.83880
H -0.97673 -1.34734 3.29823
H -0.26823 -2.70606 2.39206
H -1.75937 -4.09353 -0.36662
H -0.14785 -3.70668 0.27835
H -0.50632 -3.44320 -1.44851
H   1.55099 -1.77240 1.30326
H   3.56270 0.65190 -0.82563
H   1.52795 3.80899 -1.62260
H   0.55877 2.59561 -2.53483
H   2.16268 2.14935 -1.90117
H -0.48560 4.42701 0.01211
H -1.41261 3.28051 1.03422
H -1.65346 3.29148 -0.74227
H   2.58134 2.17611 1.10579
H   1.19208 2.49605 2.18078
H   1.82127 3.80142 1.11117

H   0.61395 -0.14125 -1.46956
H   4.80542 -1.72146 -1.69852
H   3.70247 -2.85903 -0.84339
H   3.07111 -1.83772 -2.17632
H   5.63417 -0.47894 0.74780
H   4.46369 0.24289 1.90698
H   4.57097 -1.53615 1.74418

TS(11''-9')1_{Me}
SCF Energy = -740.009205595
Enthalpy 0K = -739.575031
Enthalpy 298K = -739.545240
Free Energy 298K = -739.631309
SCF(DCM) = -740.061886685
SCF(BP86-D3) = -0.07715848
Lowest Frequencies = -52.0055
24.1907 cm-1
C   0.88717 -2.10981 -0.51443
C   0.92593 -1.89486 0.90035
C   1.76699 -0.71950 1.11156
C   2.38596 -0.35631 -0.19521
C   1.82313 -1.18613 -1.18564
Rh 0.09591 -0.01089 -0.01199
P   0.00941 2.31746 -0.07041
C -1.61068 3.12019 -0.53090
C   0.33685 -2.75993 1.97648
C   2.22290 -0.21678 2.44812
C   3.52022 0.61317 -0.35293
C   2.15155 -1.22193 -2.65113
C   0.17278 -3.21784 -1.23188
B -1.60777 0.13473 1.30694
P -2.81512 -0.44389 -0.14405
C -3.39880 -2.20002 -0.03328
C -4.36410 0.54145 -0.46420
C   0.44395 3.19398 1.51217
C   1.14176 3.12459 -1.31682
H   3.43065 1.47630 0.32613
H   3.61084 0.98550 -1.38502
H   4.47351 0.11150 -0.10064
H   2.82529 -2.06859 -2.88158
H   2.65774 -0.30094 -2.97855
H   1.24981 -1.35038 -3.27230
H -0.32801 -2.86592 -2.14943
H -0.56933 -3.71749 -0.59210
H   0.90299 -3.98736 -1.54582
H   1.08004 -3.50466 2.31648
H -0.54757 -3.31403 1.62488
H   0.02617 -2.16698 2.84983
H   3.10671 -0.79389 2.78367
H   1.43854 -0.33061 3.21124
H   2.52065 0.84246 2.41328
H -1.65529 -0.73991 2.14826
H -2.20469 -0.41209 -1.45568
H -1.47925 4.21324 -0.57913
H -2.36797 2.87993 0.22854
H -1.93717 2.75794 -1.51881
H   0.35283 4.28519 1.38526
H   1.47870 2.95101 1.79845
H -0.23581 2.85004 2.30594
H   0.88221 2.76977 -2.32636
H   2.18907 2.86479 -1.10945

H   1.03001 4.22072 -1.27720
H -2.00266 1.21883 1.68936
H -3.94651 -2.49442 -0.94109
H -2.53832 -2.86616 0.11753
H -4.06093 -2.27772 0.84312
H -4.97295 0.51997 0.45312
H -4.10171 1.58421 -0.69112
H -4.93281 0.11088 -1.30277

INT(11’-9')$_{Me}$
SCF Energy = -740.043358204
Enthalpy 0K = -739.608642
Enthalpy 298K = -739.578506
Free Energy 298K = -739.663882
SCF(DCM) = -740.093962560
SCF(BP86-D3) = -0.08391334
Lowest Frequencies = 39.5891
61.4573 cm-1
C -2.01915 -0.80442 -0.72273
C -1.98360 -0.75813 0.73447
C -0.89870 -1.58946 1.18967
C -0.29502 -2.20300 0.01859
C -0.97720 -1.71519 -1.15923
Rh -0.02418 0.10378 -0.15527
P   2.31596 0.06412 -0.00771
C   3.29900 1.62469 -0.24470
C -3.01182 -0.14100 1.63971
C -0.61090 -1.93986 2.62237
C   0.67327 -3.35100 0.05136
C -0.78113 -2.20911 -2.56677
C -3.09485 -0.23736 -1.60781
B   0.27510 1.66923 1.71249
P -0.45452 2.40244 0.15468
C -2.21964 2.96122 0.06643
C   0.43065 3.63513 -0.90792
C   3.03977 -0.63614 1.55670
C   3.07969 -0.99779 -1.33554
H   1.43152 -3.25169 0.84371
H   1.18441 -3.49698 -0.91212
H   0.11630 -4.28316 0.26253
H -1.42764 -3.08677 -2.75259
H   0.25754 -2.51949 -2.75730
H -1.04504 -1.44085 -3.30914
H -2.69929 0.05817 -2.59181
H -3.58108 0.64128 -1.15875
H -3.88193 -0.99464 -1.78084
H -3.55038 -0.94142 2.17902
H -3.76480 0.43276 1.07976
H -2.55981 0.51344 2.40409
H -1.26454 -2.76887 2.95359
H -0.79048 -1.08558 3.29263
H   0.42984 -2.26847 2.76552
H -0.48932 1.29228 2.55978
H   0.35815 0.68921 -1.56807
H   4.36894 1.38413 -0.13460
H   3.02314 2.38045 0.50360
H   3.12688 2.01429 -1.25964
H   4.13702 -0.69591 1.46855
H   2.63844 -1.64481 1.73282
H   2.76855 0.00938 2.40457
H   2.76294 -0.62295 -2.32079
H   2.75402 -2.04177 -1.22822
H   4.17913 -0.95794 -1.26368
H   1.42121 1.90746 1.98128
H -2.60670 2.86680 -0.95991
H -2.83799 2.37718 0.75922
H -2.25442 4.02096 0.36839
H   0.21320 4.64576 -0.52576
H   1.51331 3.46717 -0.87549
H   0.07751 3.55432 -1.94792

TS(11’’-6)2$_{Me}$
SCF Energy = -740.024508379
Enthalpy 0K = -739.590706
Enthalpy 298K = -739.560870
Free Energy 298K = -739.646322
SCF(DCM) = -740.074488893
SCF(BP86-D3) = -0.08181577
Lowest Frequencies = -55.1734
27.9411 cm-1
C   2.12047 0.11634 -1.08425
C   2.23257 -0.88636 -0.05841
C   1.92418 -0.25662 1.23366
C   1.61725 1.11781 0.98499
C   1.73809 1.36348 -0.45634
Rh 0.02293 -0.07716 -0.22256
P -1.78872 1.36634 -0.03892
C -3.20191 1.01990 -1.20300
C   2.91824 -2.21039 -0.26719
C   2.16409 -0.85278 2.59505
C   1.41950 2.16741 2.04392
C   1.82885 2.70656 -1.12684
C   2.52943 -0.05531 -2.52243
B -0.08136 -2.87683 1.06258
P -1.35878 -1.98087 0.01000
C -1.77054 -2.84034 -1.58797
C -2.96377 -1.96790 0.96708
C -2.59663 1.55277 1.63155
C -1.46591 3.14909 -0.46815
H   0.97422 1.74379 2.95761
H   0.77971 2.99621 1.70184
H   2.39257 2.60987 2.32838
H   2.89273 2.99018 -1.23312
H   1.34097 3.50282 -0.54765
H   1.39457 2.69425 -2.13889
H   1.99526 0.64042 -3.18689
H   2.34298 -1.07907 -2.88062
H   3.61200 0.14328 -2.63286
H   3.98624 -2.02741 -0.49012
H   2.50168 -2.77200 -1.11811
H   2.86958 -2.85391 0.62177
H   3.14366 -0.51408 2.98198
H   2.17725 -1.95158 2.57332
H   1.39887 -0.54156 3.32335
H   0.75701 -3.55070 0.54198
H -0.51398 -0.18860 -1.69221
H -4.03139 1.72059 -1.01429
H -3.56920 -0.01030 -1.09728
H -2.83756 1.15390 -2.23347
H -3.40864 2.29707 1.58102
H -1.84002 1.89166 2.35615
H -3.00454 0.59230 1.97456

H -1.04449 3.22252 -1.48125
H -0.76142 3.59267 0.24983
H -2.41456 3.70929 -0.42643
H -0.13686 -2.70784 2.24875
H -2.41610 -2.19561 -2.20446
H -0.83498 -3.02853 -2.13464
H -2.28336 -3.79522 -1.39117
H -3.37567 -2.98972 0.96867
H -2.74851 -1.67270 2.00476
H -3.71357 -1.28937 0.53581

INT(11''-6)2_{Me}
SCF Energy = -740.028795941
Enthalpy 0K = -739.594380
Enthalpy 298K = -739.564068
Free Energy 298K = -739.650403
SCF(DCM) = -740.079538564
SCF(BP86-D3) = -0.08259835
Lowest Frequencies = 27.6996 50.2112 cm-1

C 2.17554 0.05081 -1.00354
C 2.24158 -0.90750 0.08270
C 1.79927 -0.22684 1.30788
C 1.54748 1.13891 0.98246
C 1.76629 1.32103 -0.46074
Rh 0.02242 -0.10600 -0.28389
P -1.76404 1.36184 -0.05020
C -3.36912 0.93495 -0.88926
C 3.12369 -2.13056 0.04474
C 1.83673 -0.82826 2.68437
C 1.31355 2.24790 1.96950
C 1.89491 2.63252 -1.18820
C 2.67466 -0.19235 -2.40130
B 0.24563 -2.78752 -0.13423
P -1.42468 -1.95414 -0.04799
C -2.53093 -2.41676 -1.46779
C -2.41152 -2.24515 1.50246
C -2.31525 1.69320 1.69827
C -1.48873 3.07705 -0.71810
H 0.83510 1.88511 2.89239
H 0.69934 3.06253 1.55406
H 2.28175 2.69631 2.26042
H 2.96504 2.88573 -1.30430
H 1.42738 3.46476 -0.64297
H 1.45794 2.59208 -2.19804
H 2.21502 0.49899 -3.12345
H 2.46588 -1.22076 -2.73345
H 3.76990 -0.04341 -2.44463
H 4.17463 -1.78552 0.10322
H 3.01696 -2.70244 -0.88804
H 2.94620 -2.80967 0.88924
H 2.86287 -0.79070 3.09571
H 1.52905 -1.88598 2.67255
H 1.18135 -0.28886 3.38584
H 0.64477 -3.14150 -1.20728
H -0.49410 -0.21324 -1.76341
H -4.05476 1.79446 -0.81578
H -3.84137 0.06917 -0.40228
H -3.18536 0.70344 -1.94907
H -3.15462 2.40803 1.70556
H -1.47924 2.11061 2.27787

H -2.63187 0.75053 2.16878
H -1.22722 3.02024 -1.78550
H -0.67201 3.57303 -0.17589
H -2.40918 3.67161 -0.59761
H 0.75259 -3.09568 0.90539
H -3.41822 -1.77315 -1.54289
H -1.94379 -2.33981 -2.39473
H -2.85561 -3.46034 -1.32892
H -2.79117 -3.27979 1.50158
H -1.75573 -2.10321 2.37430
H -3.26646 -1.55414 1.56995

TS(11''-6)2_{Me}
SCF Energy = -740.028500109
Enthalpy 0K = -739.594319
Enthalpy 298K = -739.564734
Free Energy 298K = -739.649046
SCF(DCM) = -740.079634536
SCF(BP86-D3) = -0.08245235
Lowest Frequencies = -33.4525 35.3394 cm-1

C 2.16988 0.23371 -0.99082
C 2.24833 -0.87769 -0.06474
C 1.79847 -0.39239 1.25056
C 1.51629 0.99960 1.13633
C 1.74090 1.39894 -0.26257
Rh 0.02587 -0.08550 -0.30476
P -1.77353 1.37188 -0.05099
C -3.28304 1.07379 -1.09607
C 3.14859 -2.06594 -0.29311
C 1.83829 -1.20011 2.51695
C 1.23396 1.94092 2.27464
C 1.87191 2.80671 -0.77667
C 2.67671 0.21340 -2.40648
B 0.25031 -2.72417 -0.54955
P -1.39678 -1.96561 -0.09651
C -2.72783 -2.31684 -1.34734
C -2.10185 -2.46258 1.55129
C -2.49071 1.48086 1.66517
C -1.45064 3.16131 -0.44871
H 0.75654 1.42668 3.12319
H 0.59033 2.78410 1.97634
H 2.17943 2.37742 2.64676
H 2.94339 3.07732 -0.82148
H 1.38384 3.54355 -0.12357
H 1.46738 2.92001 -1.79460
H 2.21954 1.00778 -3.01526
H 2.47366 -0.75129 -2.89572
H 3.77167 0.37063 -2.41912
H 4.19555 -1.70653 -0.25620
H 2.99571 -2.53385 -1.27657
H 3.03197 -2.83936 0.47784
H 2.86701 -1.22951 2.92229
H 1.52904 -2.24347 2.34318
H 1.19079 -0.77263 3.29835
H 0.50285 -2.91803 -1.70505
H -0.48401 -0.11639 -1.79017
H -3.96998 1.92894 -0.99218
H -3.80226 0.16303 -0.76934
H -2.99025 0.96321 -2.15078
H -3.32625 2.19995 1.68487

```
H  -1.71506  1.80649  2.37452
H  -2.85301  0.48915  1.97316
H  -1.06787  3.25326 -1.47584
H  -0.71436  3.58369  0.24856
H  -2.39222  3.72705 -0.35726
H   0.88540 -3.17907  0.35629
H  -3.68592 -1.84740 -1.08221
H  -2.39638 -1.95687 -2.33203
H  -2.87181 -3.40801 -1.39025
H  -2.42861 -3.51413  1.50587
H  -1.31925 -2.36054  2.31724
H  -2.96356 -1.83315  1.82312
```

6_{Me}

```
SCF Energy = -740.053796342
Enthalpy 0K = -739.619076
Enthalpy 298K = -739.588958
Free Energy 298K = -739.674818
SCF(DCM) = -740.106033752
SCF(BP86-D3) = -0.08260266
Lowest Frequencies = 29.4163
45.1276 cm-1
C  -1.23175 -1.16231  1.25410
C  -1.48587 -1.76623 -0.05969
C  -2.10962 -0.77007 -0.88252
C  -2.28515  0.44011 -0.09156
C  -1.76944  0.18044  1.24143
Rh -0.06301  0.02951 -0.24992
P   2.02870 -1.03127 -0.07405
C   3.28990 -0.60408 -1.37418
C  -1.36272 -3.22271 -0.40863
C  -2.61276 -0.95867 -2.28452
C  -3.09374  1.62514 -0.54012
C  -1.91566  1.07030  2.44290
C  -0.76190 -1.89922  2.47730
B   0.60342  1.66157 -2.01143
P   0.84798  2.20110 -0.18605
C   2.53738  2.65983  0.44554
C  -0.19225  3.60594  0.43534
C   2.93496 -0.81421  1.53971
C   2.02189 -2.88620 -0.23300
H  -2.74614  2.02051 -1.50872
H  -3.07639  2.44075  0.19790
H  -4.15104  1.32902 -0.66865
H  -2.83081  0.79616  3.00020
H  -2.00926  2.13008  2.16428
H  -1.06583  0.97078  3.13561
H  -0.31167 -1.22049  3.21813
H  -0.02629 -2.68240  2.23474
H  -1.61705 -2.40062  2.96786
H  -2.34929 -3.71006 -0.29642
H  -0.66540 -3.75620  0.25226
H  -1.04191 -3.37826 -1.45060
H  -3.68762 -1.21864 -2.26407
H  -2.08458 -1.77139 -2.80567
H  -2.50584 -0.03952 -2.88086
H   1.62099  1.54360 -2.65007
H   0.34376  0.30593 -1.90599
H   4.23608 -1.12485 -1.15483
H   3.46932  0.47795 -1.42964
H   2.90753 -0.93205 -2.35319
H   3.87824 -1.38493  1.53276
H   2.29595 -1.18090  2.35776
H   3.15285  0.24804  1.71740
H   1.53484 -3.17933 -1.17436
H   1.48460 -3.34298  0.61068
H   3.06111 -3.25387 -0.23132
H  -0.38008  2.13906 -2.52389
H  -0.23350  3.61598  1.53568
H   0.26096  4.54898  0.08722
H  -1.20537  3.53018  0.01995
H   2.54449  2.68767  1.54680
H   3.30190  1.95634  0.09009
H   2.78374  3.66384  0.06165
```

TS(11''-6)3_{Me}

```
SCF Energy = -739.979801160
Enthalpy 0K = -739.550677
Enthalpy 298K = -739.520095
Free Energy 298K = -739.609171
SCF(DCM) = -740.029021633
SCF(BP86-D3) = -0.07521582
Lowest Frequencies = -776.1673
11.9898 cm-1
C  -2.47378  0.19277  0.35831
C  -1.62021 -0.47974  1.34635
C  -1.17928 -1.74326  0.77243
C  -1.60415 -1.74757 -0.61026
C  -2.43879 -0.56159 -0.84227
Rh -0.14436 -0.06933 -0.26529
P   0.52343  2.19743 -0.02693
C   2.07685  2.67906 -0.92909
C  -1.52641 -0.11184  2.80149
C  -0.59616 -2.89969  1.53477
C  -1.46217 -2.88910 -1.57547
C  -3.17795 -0.27169 -2.11668
C  -3.32862  1.39520  0.63931
B   1.42372 -1.11312 -1.18212
P   2.93413 -0.66399 -0.03399
C   3.09484 -2.09697  1.14801
C   4.68180 -0.56665 -0.76408
C  -0.69390  3.40810 -0.75157
C   0.81394  2.85919  1.68802
H  -0.59252 -3.52054 -1.33913
H  -1.35297 -2.53752 -2.61356
H  -2.36272 -3.52963 -1.53536
H  -4.11909 -0.85131 -2.15843
H  -2.58718 -0.55131 -3.00345
H  -3.44399  0.79297 -2.20562
H  -3.58215  1.95109 -0.27678
H  -2.85633  2.08955  1.35305
H  -4.28289  1.07503  1.09841
H  -2.36715 -0.56112  3.36360
H  -1.57933  0.97705  2.95739
H  -0.59198 -0.47468  3.25626
H  -1.41484 -3.52733  1.93562
H   0.00261 -2.56468  2.39603
H   0.03223 -3.54399  0.90174
H   1.37470 -2.08595 -1.89170
H   1.67439  0.18555  0.57372
H   2.28761  3.74811 -0.76599
H   1.94412  2.49130 -2.00573
```

H 2.92718 2.08560 -0.55925
H -0.30333 4.43521 -0.66273
H -1.65708 3.34101 -0.22700
H -0.84961 3.16613 -1.81414
H 1.63035 2.29249 2.16171
H -0.09717 2.73552 2.29290
H 1.08078 3.92789 1.65042
H 0.76063 -0.07145 -1.75271
H 3.87226 -1.86117 1.89168
H 2.13889 -2.25947 1.66415
H 3.37837 -3.01295 0.60640
H 5.41168 -0.37657 0.03833
H 4.91366 -1.52454 -1.25954
H 4.72929 0.24630 -1.50337

INT(11’’-6)$_{Me}$
SCF Energy = -740.022532657
Enthalpy 0K = -739.589577
Enthalpy 298K = -739.559005
Free Energy 298K = -739.648177
SCF(DCM) = -740.073251311
SCF(BP86-D3) = -0.07449256
Lowest Frequencies = 21.2231 22.1635 cm-1
C -2.31834 0.03166 0.87114
C -1.73038 -1.23046 1.26487
C -1.50426 -2.02107 0.06541
C -1.98514 -1.25624 -1.06727
C -2.47670 0.01435 -0.58423
Rh -0.27892 -0.11084 -0.05181
P 0.42036 2.12478 -0.09324
C 1.66467 2.50522 -1.42181
C -1.49276 -1.68766 2.67454
C -1.01497 -3.43868 0.02385
C -2.03015 -1.73023 -2.48995
C -3.23871 1.01353 -1.40839
C -2.88796 1.05159 1.81675
B 1.67490 -1.09835 -0.25194
P 3.53852 -0.73970 -0.48191
C 4.27398 0.52631 0.69616
C 4.43133 -2.31289 0.00521
C -0.85247 3.44553 -0.40751
C 1.20545 2.71968 1.48559
H -1.19077 -2.40181 -2.72605
H -2.01244 -0.89161 -3.20249
H -2.96480 -2.29527 -2.66583
H -4.27587 0.65975 -1.55722
H -2.79502 1.15117 -2.40731
H -3.29893 1.99579 -0.91809
H -2.99256 2.04193 1.34879
H -2.27260 1.15988 2.72327
H -3.89859 0.74007 2.14026
H -2.40153 -2.18663 3.06089
H -1.26762 -0.84638 3.34760
H -0.66682 -2.41193 2.73771
H -1.86713 -4.13562 0.12541
H -0.30945 -3.65359 0.84105
H -0.50855 -3.66831 -0.92615
H 1.50183 -2.28348 -0.40053
H 1.17334 -0.46331 0.84814
H 2.05036 3.53166 -1.30976

H 1.16898 2.40647 -2.40043
H 2.49800 1.78724 -1.38932
H -0.35218 4.42766 -0.43606
H -1.60533 3.45630 0.39454
H -1.34815 3.26655 -1.37289
H 2.05146 2.06871 1.74839
H 0.46000 2.66701 2.29428
H 1.55520 3.75985 1.37907
H 0.93244 -0.42110 -1.23165
H 5.36616 0.52631 0.55351
H 3.90971 1.53789 0.46365
H 4.05358 0.28415 1.74878
H 5.49653 -2.21779 -0.25674
H 4.33937 -2.52128 1.08331
H 4.00370 -3.15052 -0.56563

TS(11’’-6)4$_{Me}$
SCF Energy = -739.995443753
Enthalpy 0K = -739.563791
Enthalpy 298K = -739.533160
Free Energy 298K = -739.622749
SCF(DCM) = -740.046395105
SCF(BP86-D3) = -0.07300416
Lowest Frequencies = -173.6894 11.2934 cm-1
C 2.24928 0.27007 0.87982
C 2.37040 0.36127 -0.57874
C 2.07412 -0.95602 -1.13714
C 1.75604 -1.83938 -0.04830
C 1.85614 -1.07660 1.19555
Rh 0.26987 -0.10226 -0.12207
P -0.78680 1.99656 0.05908
C -2.06279 2.39439 -1.23574
C 2.96699 1.50069 -1.34828
C 2.15499 -1.33417 -2.58596
C 1.49836 -3.31348 -0.15237
C 1.66537 -1.63827 2.57142
C 2.68287 1.30705 1.87824
B -1.54004 -1.45608 -0.99522
P -3.04351 -0.84554 0.00902
C -4.65846 -0.75294 -0.93065
C -3.39991 -2.00061 1.43469
C -1.65562 2.34474 1.66982
C 0.31366 3.49455 -0.09668
H 1.04940 -3.58205 -1.12033
H 0.82756 -3.67357 0.64306
H 2.45187 -3.86561 -0.05887
H 2.61331 -2.08642 2.92677
H 0.90475 -2.43391 2.58923
H 1.37674 -0.86203 3.29647
H 2.05946 1.29511 2.78605
H 2.66164 2.32337 1.45813
H 3.72450 1.11062 2.19328
H 4.05092 1.32198 -1.48368
H 2.85900 2.46058 -0.82352
H 2.52598 1.59566 -2.35311
H 3.15826 -1.73743 -2.81826
H 1.98306 -0.46853 -3.24349
H 1.41717 -2.11064 -2.84194
H -1.62068 -1.92786 -2.10690
H -0.79179 -0.35173 -1.42618

H -2.49092 3.39168 -1.04477
H -2.86526 1.64409 -1.21141
H -1.58497 2.38304 -2.22736
H -2.02259 3.38464 1.68843
H -0.95908 2.18799 2.50801
H -2.49646 1.64256 1.76738
H 0.82617 3.48741 -1.07006
H 1.06017 3.51343 0.71089
H -0.30408 4.40477 -0.02524
H -0.93325 -2.09300 -0.14158
H -4.14924 -1.55259 2.10489
H -3.77787 -2.96643 1.06035
H -2.47113 -2.17027 1.99953
H -5.44684 -0.29284 -0.31523
H -4.52030 -0.15366 -1.84380
H -4.97330 -1.76914 -1.22340

TS(11''-9')2$_{Me}$
SCF Energy = -740.014758479
Enthalpy 0K = -739.580432
Enthalpy 298K = -739.551075
Free Energy 298K = -739.635787
SCF(DCM) = -740.064895108
SCF(BP86-D3) = -0.07796614
Lowest Frequencies = -123.2787 20.8173 cm-1
C -2.36490 0.26502 0.22712
C -1.83153 -0.52505 1.29707
C -1.31178 -1.75846 0.71824
C -1.49367 -1.72742 -0.70560
C -2.10229 -0.44008 -1.03615
Rh -0.17168 0.09002 -0.24686
P 2.53817 -0.60566 0.09149
C 3.57440 -0.07273 -1.35438
C -1.92889 -0.23920 2.77015
C -0.83739 -2.92065 1.54263
C -1.29539 -2.86186 -1.67313
C -2.65425 -0.05375 -2.37945
C -3.21041 1.49919 0.35537
B 1.80962 1.17175 0.99965
P 0.50693 2.22938 0.02626
C -0.40771 3.41418 1.12099
C 1.17650 3.26595 -1.35658
C 3.76945 -0.99950 1.44074
C 2.09261 -2.35042 -0.41291
H -0.55986 -3.59611 -1.31060
H -0.96220 -2.50472 -2.66019
H -2.24814 -3.40265 -1.82420
H -3.68175 -0.44795 -2.49435
H -2.04540 -0.46179 -3.20024
H -2.70098 1.03892 -2.50355
H -3.08307 2.18022 -0.50029
H -3.00239 2.05650 1.28086
H -4.27695 1.20717 0.38309
H -2.76718 -0.79971 3.22444
H -2.10800 0.82892 2.96779
H -1.01164 -0.53119 3.30655
H -1.69831 -3.36546 2.07607
H -0.11265 -2.61720 2.31683
H -0.39223 -3.71876 0.93031
H 1.58209 0.98867 2.16712
H 0.52007 0.20119 -1.66959
H 4.33044 -0.84689 -1.56050
H 4.07731 0.87922 -1.13149
H 2.92927 0.04597 -2.23780
H 4.47200 -1.77179 1.08918
H 3.22937 -1.37684 2.32319
H 4.31749 -0.09026 1.72531
H 1.47383 -2.32278 -1.31886
H 1.54677 -2.84845 0.39916
H 3.03031 -2.89910 -0.60682
H 2.91897 1.57786 0.72772
H -1.27492 3.84207 0.59407
H -0.74560 2.88154 2.02145
H 0.27203 4.22975 1.41674
H 1.76527 4.10596 -0.95328
H 1.81356 2.63964 -1.99691
H 0.34498 3.66099 -1.96176

9'$_{Me}$
SCF Energy = -740.034877218
Enthalpy 0K = -739.601345
Enthalpy 298K = -739.570960
Free Energy 298K = -739.659266
SCF(DCM) = -740.085920998
SCF(BP86-D3) = -0.07330278
Lowest Frequencies = 19.8874 40.8438 cm-1
C -2.36032 0.45713 0.96691
C -2.05570 -0.89229 1.26962
C -2.07929 -1.67999 0.01376
C -2.39715 -0.80705 -1.06394
C -2.48033 0.55757 -0.50484
Rh -0.47308 -0.06309 -0.23435
P 3.31546 -0.65840 0.07106
C 3.67446 -0.70826 -1.74213
C -1.83257 -1.47852 2.63299
C -1.91629 -3.17083 -0.05761
C -2.68203 -1.18402 -2.49031
C -2.93966 1.77130 -1.25288
C -2.62691 1.56821 1.94030
B 2.75075 1.04906 0.80638
P 1.03020 1.69342 0.05285
C 0.53370 3.05162 1.23181
C 1.40133 2.66094 -1.49799
C 4.85430 -1.26074 0.90017
C 2.04569 -1.97503 0.35807
H -2.16326 -2.11037 -2.78011
H -2.37480 -0.39216 -3.18984
H -3.76669 -1.34994 -2.62709
H -4.04643 1.80749 -1.26373
H -2.59785 1.75934 -2.29900
H -2.58551 2.69973 -0.77886
H -2.32350 2.54902 1.54389
H -2.10650 1.41046 2.89762
H -3.70964 1.62528 2.15899
H -2.76875 -1.92655 3.01594
H -1.50808 -0.71671 3.35810
H -1.07752 -2.28108 2.61638
H -2.86561 -3.67099 0.21182
H -1.15112 -3.53674 0.64597
H -1.64263 -3.50533 -1.06993

```
H   2.58745  0.82698  1.99062
H   0.20832 -0.03527 -1.66635
H   4.05357 -1.69979 -2.03541
H   4.42836  0.05755 -1.98161
H   2.74906 -0.49203 -2.29838
H   5.14389 -2.25042  0.51441
H   4.68111 -1.31894  1.98553
H   5.66470 -0.54002  0.71116
H   1.09830 -1.67798 -0.16184
H   1.86025 -2.06896  1.43880
H   2.35978 -2.94693 -0.05549
H   3.61606  1.86990  0.57506
H  -0.32380  3.61577  0.83206
H   0.26801  2.61605  2.20575
H   1.38473  3.73897  1.36567
H   2.13423  3.45319 -1.27562
H   1.81194  1.98573 -2.26307
H   0.47662  3.11264 -1.88995
```

TS(9'-9)$_{Me}$

```
SCF Energy = -740.033379540
Enthalpy 0K = -739.600573
Enthalpy 298K = -739.570433
Free Energy 298K = -739.659330
SCF(DCM) = -740.084485369
SCF(BP86-D3) = -0.06942391
Lowest Frequencies = -71.8798
13.3292 cm-1
C  -2.64207  0.76112  0.49293
C  -2.42824 -0.37642  1.31022
C  -2.30246 -1.56322  0.43162
C  -2.46449 -1.15824 -0.92355
C  -2.57359  0.31093 -0.91906
Rh -0.64858 -0.10384 -0.15843
P   0.98305  1.49907  0.04996
C   0.62734  2.98351  1.12231
C  -2.41200 -0.42858  2.81041
C  -2.11375 -2.96352  0.93438
C  -2.56686 -2.04208 -2.13483
C  -2.88173  1.16075 -2.11358
C  -2.97582  2.15515  0.93894
B   2.35761  0.47768  1.06677
P   3.65496 -0.61768  0.10901
C   4.73989  0.26731 -1.10120
C   1.66663  2.28607 -1.49603
C   4.82258 -1.32793  1.35525
C   2.96173 -2.07274 -0.79581
H  -2.03563 -2.99535 -1.99105
H  -2.14990 -1.55628 -3.02990
H  -3.62638 -2.27856 -2.34468
H  -3.97595  1.19194 -2.28107
H  -2.41547  0.75983 -3.02641
H  -2.53834  2.19759 -1.97522
H  -2.57726  2.91653  0.24994
H  -2.58937  2.36823  1.94726
H  -4.07350  2.28776  0.96923
H  -3.37378 -0.81865  3.19295
H  -2.25851  0.56761  3.25246
H  -1.62002 -1.09490  3.18988
H  -3.07283 -3.36577  1.31188
H  -1.39795 -3.00233  1.77200
H  -1.75796 -3.63910  0.14202
H   1.79829 -0.30459  1.81933
H   0.21663 -0.43516 -1.45089
H   5.54020 -0.40190 -1.45382
H   5.18847  1.14350 -0.60825
H   4.14660  0.60678 -1.96304
H   5.56851 -1.97206  0.86398
H   4.25177 -1.91507  2.09070
H   5.33096 -0.50439  1.87953
H   2.24244 -1.72613 -1.55381
H   2.42644 -2.71006 -0.07465
H   3.76403 -2.65519 -1.27506
H   3.02170  1.28436  1.69182
H   1.53733  3.59706  1.22431
H  -0.17629  3.59405  0.68193
H   0.31803  2.63870  2.12042
H   2.57292  2.87066 -1.26919
H   1.89230  1.50426 -2.23603
H   0.90726  2.95747 -1.92782
```

9$_{Me}$

```
SCF Energy = -740.051459135
Enthalpy 0K = -739.618276
Enthalpy 298K = -739.587595
Free Energy 298K = -739.677850
SCF(DCM) = -740.101774541
SCF(BP86-D3) = -0.06979090
Lowest Frequencies = 10.5148
27.8475 cm-1
C  -2.15056 -0.68844  1.31074
C  -2.05008 -1.66005  0.21286
C  -2.37057 -0.98393 -1.01445
C  -2.59811  0.42579 -0.70298
C  -2.51369  0.57700  0.75569
Rh -0.52778 -0.02961 -0.19877
P   0.94748  1.70514 -0.00468
C   0.73374  3.07308  1.23411
C  -1.81920 -3.13638  0.37855
C  -2.54912 -1.62196 -2.36456
C  -3.11364  1.46185 -1.66291
C  -2.85964  1.82349  1.52164
C  -2.00340 -1.02221  2.76904
B   1.70368  0.09564  0.77897
P   3.33762 -0.71140  0.08017
C   4.83222  0.32908  0.39549
C   1.74199  2.52901 -1.47139
C   3.66065 -2.31885  0.93435
C   3.32809 -1.09281 -1.72574
H  -2.72512  1.30068 -2.68020
H  -2.83522  2.48034 -1.35102
H  -4.21799  1.42100 -1.71636
H  -3.94746  1.85777  1.71775
H  -2.60160  2.73696  0.96333
H  -2.35069  1.86133  2.49744
H  -1.75618 -0.13429  3.37106
H  -1.21793 -1.77571  2.93923
H  -2.94764 -1.43897  3.16609
H  -2.77599 -3.65575  0.57389
H  -1.15141 -3.35416  1.22692
H  -1.37944 -3.58265 -0.52679
H  -3.60133 -1.93627 -2.49395
```

H -1.91803 -2.51593 -2.48285
H -2.30736 -0.92412 -3.18028
H   0.86211 -0.89421 0.50010
H   0.18975 -0.16909 -1.60554
H   5.74336 -0.20109 0.07715
H   4.89490 0.55253 1.47161
H   4.74765 1.27472 -0.16131
H   4.60119 -2.76811 0.57898
H   2.82603 -3.00839 0.73443
H   3.72282 -2.14062 2.01881
H   3.33012 -0.15581 -2.30245
H   2.41100 -1.65278 -1.96540
H   4.21462 -1.68762 -1.99503
H   1.82169 0.16378 1.98199
H   1.69726 3.56732 1.43903
H   0.02058 3.82065 0.85154
H   0.34095 2.64097 2.16578
H   2.69403 3.01179 -1.19596
H   1.90381 1.78656 -2.26589
H   1.05444 3.29921 -1.85734

$H_3BP^tBu_2H$
SCF Energy = -349.511556760
Enthalpy 0K = -349.233577
Enthalpy 298K = -349.217395
Free Energy 298K = -349.272823
SCF(DCM) = -349.408812655
SCF(BP86-D3) = -0.03960832
Lowest Frequencies = 59.6393
75.2490 cm-1
C   1.61133 -0.29226 0.01337
P -0.00239 0.63183 -0.44634
C -1.61690 -0.28954 0.01490
B   0.01140 2.51628 0.09682
H -0.82936 3.06237 -0.59323
H -0.27153 2.49567 1.28549
H   1.14387 2.91341 -0.11928
H -0.00820 0.43219 -1.87032
C   1.54823 -1.81758 -0.19184
C   2.68672 0.30463 -0.92744
C   1.96756 0.04149 1.47965
C -1.80016 -1.59805 -0.78135
C -1.64874 -0.55649 1.53459
C -2.75624 0.68985 -0.35928
H   3.67630 -0.10208 -0.64877
H   2.49999 0.03896 -1.98260
H   2.73405 1.40264 -0.84783
H   2.55216 -2.24705 -0.01471
H   0.85706 -2.30689 0.51402
H   1.24921 -2.08770 -1.21893
H   2.95605 -0.39088 1.72106
H   2.01655 1.12965 1.64536
H   1.23658 -0.38014 2.18948
H -2.79320 -2.02674 -0.54989
H -1.76378 -1.42054 -1.87017
H -1.04582 -2.35905 -0.53185
H -2.64293 -0.94957 1.81652
H -0.89952 -1.30517 1.84289
H -1.47610 0.36824 2.11003
H -3.72710 0.21541 -0.12372
H -2.68218 1.63579 0.19859

H -2.75397 0.93034 -1.43682

10_{CptBu}
SCF Energy = -819.812758782
Enthalpy 0K = -819.301804
Enthalpy 298K = -819.269063
Free Energy 298K = -819.362518
SCF(DCM) = -819.861215310
SCF(BP86-D3) = -0.09135497
Lowest Frequencies = 20.8444
30.8038 cm-1
C   3.04424 -0.74712 -1.84346
C   1.80973 -1.32450 -2.22166
C   1.52093 -2.41305 -1.29799
C   2.59909 -2.51256 -0.37413
C   3.52757 -1.44288 -0.65627
Rh 1.64504 -0.49054 -0.03963
C   1.62986 -0.86302 2.04487
H   1.17593 -1.00589 -3.04831
H   0.64850 -3.06526 -1.33706
H   2.68863 -3.23573 0.43645
H   4.48438 -1.28138 -0.15971
H   3.54714 0.07873 -2.34682
B -0.68731 0.43165 -0.55387
P -2.48150 0.32306 0.29102
C -3.76749 1.26903 -0.77566
C -4.99420 1.65217 0.08108
P   2.30002 1.65495 0.49828
C   1.30402 2.57060 1.77194
C -3.00187 -1.42210 0.87743
C -2.11195 -1.72148 2.10772
C -4.48413 -1.50179 1.29507
C -2.70047 -2.43508 -0.24906
C   4.03174 1.77319 1.16517
C   2.32431 2.82733 -0.94554
C -3.07805 2.56328 -1.26866
C -4.18094 0.40492 -1.98598
H -0.80313 -0.11693 -1.62431
H -2.52711 1.07136 1.51386
H -2.31923 -1.03304 2.94469
H -1.03721 -1.67282 1.87032
H -2.32920 -2.74577 2.46015
H -4.67352 -2.50284 1.72284
H -5.17103 -1.37964 0.44285
H -4.74263 -0.76033 2.06930
H -1.64065 -2.40400 -0.54991
H -3.31869 -2.26409 -1.14499
H -2.92060 -3.45358 0.11722
H -5.68614 2.24487 -0.54381
H -4.71361 2.27796 0.94513
H -5.55091 0.77927 0.45011
H -2.23646 2.35569 -1.94668
H -2.70922 3.18422 -0.43391
H -3.82310 3.16322 -1.82124
H -3.31178 0.09273 -2.58876
H -4.84093 1.00207 -2.63980
H -4.74410 -0.49399 -1.68734
H   1.69199 3.59641 1.87939
H   1.37397 2.05495 2.74094
H   0.25216 2.60685 1.45292
H   4.28668 2.82091 1.39398

```
H   4.74091  1.38408  0.41859
H   4.11495  1.17017  2.08234
H   1.32454  2.85601 -1.40319
H   3.04780  2.47273 -1.69520
H   2.61246  3.83834 -0.61360
H   2.65705 -0.81797  2.44301
H   0.99469 -0.15782  2.60255
H   1.23900 -1.88194  2.19677
H  -0.02016 -0.21039  0.37653
H  -0.46378  1.61700 -0.61135
```

10_{CpPh}

SCF Energy = -967.407307566
Enthalpy 0K = -966.958313
Enthalpy 298K = -966.927077
Free Energy 298K = -967.022294
SCF(DCM) = -967.455106886
SCF(BP86-D3) = -0.07788565
Lowest Frequencies = 10.8154
23.1201 cm-1

```
C   3.22405 -2.50018 -0.65065
C   3.41868 -1.35479  0.14813
C   4.72259 -0.95111  0.51106
C   5.82458 -1.70050  0.07709
C   5.63157 -2.84382 -0.71825
C   4.33553 -3.24165 -1.08190
P   1.96181 -0.39810  0.72863
C   2.31387  1.39044  0.50487
C   2.11635  2.28970  1.57356
C   2.31968  3.66517  1.37575
C   2.72017  4.14388  0.11761
C   2.92320  3.24751 -0.94673
C   2.71988  1.87171 -0.75877
B   0.26319 -1.02474 -0.05418
Rh -1.91286  0.30423 -0.03544
P  -2.99947 -1.68796  0.37275
C  -2.76469 -2.94554 -0.97706
C  -2.93839  0.76218 -2.04280
C  -1.57348  1.09206 -2.21480
C  -1.23518  2.13034 -1.25150
C  -2.41117  2.45444 -0.51645
C  -3.46668  1.56900 -0.94906
H  -3.50045  0.03004 -2.62337
H  -0.88759  0.63944 -2.93030
H  -0.25616  2.59611 -1.13564
H  -2.49115  3.20117  0.27341
H  -4.50330  1.59826 -0.61360
C  -2.12713  0.73575  2.02713
C  -2.56731 -2.63118  1.91363
C  -4.84773 -1.52594  0.49483
H   0.38777 -0.87466 -1.24483
H   1.97670 -0.51403  2.15619
H  -3.09833 -3.59686  1.91577
H  -2.86438 -2.05390  2.80158
H  -1.48229 -2.80949  1.94219
H  -5.31074 -2.51352  0.65457
H  -5.24187 -1.08746 -0.43462
H  -5.10338 -0.86443  1.33676
H  -1.68940 -3.15174 -1.08546
H  -3.14419 -2.53841 -1.92674
H  -3.30209 -3.87771 -0.73646
H  -3.18768  0.91853  2.26459
H  -1.74222 -0.06782  2.67281
H  -1.55024  1.65334  2.22430
H  -0.45571 -0.21912  0.68751
H   0.11775 -2.16893  0.31633
H   1.81446  1.92123  2.55998
H   2.17543  4.35966  2.20909
H   2.88562  5.21528 -0.03186
H   3.24929  3.61895 -1.92318
H   2.89331  1.17558 -1.58623
H   4.87727 -0.05518  1.12126
H   6.83547 -1.39035  0.35863
H   6.49567 -3.42423 -1.05614
H   4.18646 -4.13016 -1.70291
H   2.21348 -2.81025 -0.93266
```

10_{CpMe}

SCF Energy = -583.946330241
Enthalpy 0K = -583.601214
Enthalpy 298K = -583.576257
Free Energy 298K = -583.654670
SCF(DCM) = -583.999216628
SCF(BP86-D3) = -0.05451190
Lowest Frequencies = 23.2670
35.0103 cm-1

```
C   1.18168 -2.64071  0.57415
C   2.27962 -1.87765  0.02970
C   1.94433 -1.55624 -1.35255
C   0.64182 -2.04179 -1.61743
C   0.15658 -2.69839 -0.41269
Rh  0.56268 -0.50570  0.14921
B  -1.62822  0.47764 -0.68488
P  -3.40482  0.50804  0.15454
C  -4.68766  1.14901 -1.01065
H   3.23717 -1.69768  0.51774
H   2.59030 -1.03390 -2.05888
H   0.09087 -1.93532 -2.55136
H  -0.81091 -3.18833 -0.30228
H   1.13133 -3.05440  1.58130
P   1.51391  1.58958 -0.04353
C   3.27930  1.67612  0.53190
C   0.44873 -0.14925  2.23527
C   1.60178  2.22432 -1.79014
C   0.71160  2.99439  0.87093
C  -4.02655 -1.10427  0.81002
H  -1.75546 -0.30363 -1.59818
H  -3.50512  1.38464  1.28134
H   1.22001  3.93834  0.61656
H   0.78399  2.82733  1.95563
H  -0.34749  3.06034  0.58121
H   3.67123  2.69940  0.41346
H   3.89662  0.98300 -0.05969
H   3.33321  1.38593  1.59211
H   0.58499  2.28828 -2.20577
H   2.19353  1.53056 -2.40628
H   2.07244  3.22128 -1.81116
H   1.46107 -0.17189  2.66925
H  -0.03267  0.80868  2.48302
H  -0.14714 -0.96578  2.67369
H  -1.01268  0.11212  0.41463
H  -1.42508  1.62624 -1.00900
```

```
H  -5.67368  1.18265 -0.52340
H  -4.40046  2.15881 -1.34017
H  -4.72864  0.48666 -1.88918
H  -5.02713 -0.98615  1.25242
H  -4.07467 -1.82838 -0.01810
H  -3.32881 -1.47806  1.57482
```

10_{CpCy}

```
SCF Energy = -974.667447188
Enthalpy 0K = -974.082075
Enthalpy 298K = -974.048311
Free Energy 298K = -974.146746
SCF(DCM) = -974.714671553
SCF(BP86-D3) = -0.09931083
Lowest Frequencies = 18.1021
26.3994 cm-1
C  -3.62612 -1.26901  1.47581
C  -3.02131 -1.56867  0.08255
C  -4.11442 -1.88963 -0.96401
C  -4.98147 -3.07117 -0.47689
C  -5.58908 -2.79756  0.91196
C  -4.49890 -2.45331  1.94437
P  -1.78998 -0.24594 -0.44690
C  -2.42232  1.48778 -0.11844
C  -3.78199  1.78374 -0.79549
C  -4.24012  3.23112 -0.51265
C  -3.17505  4.25847 -0.93479
C  -1.82528  3.96441 -0.25668
C  -1.34826  2.52317 -0.53814
B  -0.04109 -0.73517  0.33103
Rh  2.29003  0.26896  0.24954
C   2.27710  1.38324 -1.55154
C   3.18901  2.04956  1.31697
C   4.14735  0.97591  1.20261
C   3.67585 -0.11984  2.04049
C   2.42300  0.24704  2.58729
C   2.10791  1.58920  2.12203
H   3.26042  3.02160  0.82884
H   5.11025  1.02951  0.69451
H   4.20050 -1.05983  2.21331
H   1.79523 -0.36671  3.23225
H   1.21332  2.15670  2.37907
P   2.97371 -1.53182 -1.01789
C   2.03631 -1.89014 -2.58149
C   4.73439 -1.41053 -1.60327
C   2.93319 -3.16014 -0.12024
H  -0.19276 -0.61137  1.52414
H  -1.79951 -0.32419 -1.87937
H   2.41371 -2.81890 -3.03910
H   2.16387 -1.06056 -3.29218
H   0.96862 -2.00612 -2.34370
H   5.00411 -2.29097 -2.20900
H   5.40684 -1.34997 -0.73360
H   4.85453 -0.49932 -2.20893
H   1.90564 -3.35896  0.21870
H   3.59340 -3.11089  0.75868
H   3.27071 -3.97285 -0.78446
H   3.31106  1.54484 -1.89736
H   1.69772  0.89874 -2.35193
H   1.81634  2.35867 -1.32972
H   0.66529  0.17022 -0.30479
H   0.11533 -1.87551 -0.04864
H  -2.54569  1.53004  0.98275
H  -3.68191  1.63959 -1.88946
H  -4.55224  1.07347 -0.44908
H  -5.19326  3.42009 -1.03688
H  -4.45058  3.34054  0.56922
H  -3.04783  4.22672 -2.03478
H  -3.51171  5.28035 -0.68812
H  -1.05461  4.67812 -0.59806
H  -1.92406  4.10595  0.83767
H  -1.14405  2.41449 -1.62225
H  -0.39671  2.32444 -0.01409
H  -2.36846 -2.45963  0.18124
H  -4.75850 -1.00449 -1.12218
H  -3.65602 -2.12885 -1.94078
H  -5.77585 -3.27238 -1.21682
H  -4.35519 -3.98375 -0.43233
H  -6.30457 -1.95489  0.83980
H  -6.17108 -3.67217  1.25086
H  -4.95141 -2.21144  2.92204
H  -3.85076 -3.33650  2.10725
H  -4.25310 -0.35824  1.41910
H  -2.82769 -1.06838  2.21307
```

TS(11''-9')1_{CptBu}

```
SCF Energy = -779.272006001
Enthalpy 0K = -778.808104
Enthalpy 298K = -778.778505
Free Energy 298K = -778.864039
SCF(DCM) = -779.324066383
SCF(BP86-D3) = -0.08675788
Lowest Frequencies = -109.9345
29.1101 cm-1
C  -1.31404 -2.76606 -0.40413
C  -1.97626 -2.01357 -1.41002
C  -3.00673 -1.23757 -0.74012
C  -3.06388 -1.66114  0.66303
C  -2.00865 -2.56042  0.86506
Rh -1.13159 -0.46385 -0.07386
P  -1.90408  1.70174  0.23445
C  -1.23409  2.55156  1.75683
H  -1.75011 -1.99870 -2.47487
H  -3.74015 -0.60874 -1.24568
H  -3.77749 -1.31725  1.41065
H  -1.73523 -3.02170  1.81549
H  -0.48478 -3.45409 -0.56602
B   0.31833  0.02586 -1.59550
P   1.66050  0.04942 -0.10760
C   2.55245 -1.62424  0.36467
C   1.69742 -2.28445  1.46506
C   2.82286  1.62174 -0.16929
C   1.96397  2.85499 -0.50704
C   3.54471  1.86341  1.17108
C   3.81161  1.37606 -1.33193
C  -1.74464  2.95984 -1.12400
C  -3.73450  1.79411  0.58878
C   2.60198 -2.49460 -0.90972
C   3.98160 -1.44437  0.91887
H   0.53355 -0.89695 -2.34594
H   0.90860  0.29000  1.12529
H   1.23715  3.09388  0.28591
```

H 1.43408 2.73704 -1.46367
H 2.63917 3.72458 -0.59938
H 4.09957 2.81653 1.09915
H 4.27577 1.08210 1.41593
H 2.83628 1.95932 2.01155
H 3.28412 1.22759 -2.28883
H 4.47554 0.51512 -1.16058
H 4.45528 2.26738 -1.43920
H 2.06233 -3.31275 1.63880
H 0.63284 -2.34587 1.18821
H 1.77055 -1.73473 2.41872
H 3.21764 -2.03386 -1.69998
H 1.60246 -2.67945 -1.33139
H 3.05618 -3.46920 -0.65726
H 4.66896 -0.95689 0.21132
H 4.38891 -2.45199 1.11763
H 3.99760 -0.89812 1.87445
H -1.64541 3.57129 1.84018
H -0.13608 2.60468 1.72596
H -1.52881 1.96514 2.64135
H -2.22613 3.90432 -0.82262
H -2.23444 2.56539 -2.02767
H -0.68790 3.14014 -1.35770
H -3.98412 1.16308 1.45445
H -4.31574 1.46034 -0.28372
H -4.00833 2.83769 0.81445
H 0.29914 1.11961 -2.10952

TS(11’’-9’)1_{CpPh}
SCF Energy = -926.847315762
Enthalpy 0K = -926.448103
Enthalpy 298K = -926.419665
Free Energy 298K = -926.508361
SCF(DCM) = -926.896783129
SCF(BP86-D3) = -0.06988083
Lowest Frequencies = -106.7787
19.3299 cm-1
C 2.21788 2.37654 0.99770
C 1.56081 1.97518 -0.18561
C 1.23098 2.92608 -1.17521
C 1.54563 4.27912 -0.96896
C 2.19217 4.68034 0.21134
C 2.53067 3.72968 1.19049
P 1.14726 0.20187 -0.45715
C 2.58100 -0.85854 -0.03439
C 2.70301 -1.43564 1.24781
C 3.81807 -2.23512 1.54387
C 4.80300 -2.45907 0.56771
C 4.67774 -1.88700 -0.71000
C 3.56780 -1.08710 -1.01831
Rh -1.37467 -0.63318 -0.38166
B 0.02681 -0.24306 -1.95716
C -1.20702 -2.83366 -1.14478
C -2.47887 -2.44349 -0.60506
C -2.24393 -2.30506 0.82650
C -0.97845 -2.97619 1.16138
C -0.34170 -3.26932 -0.02262
H -1.01581 -3.05824 -2.19488
H -3.42494 -2.36377 -1.14159
H -3.01989 -2.07480 1.56045
H -0.59914 -3.12719 2.17274

H 0.65641 -3.69237 -0.14564
P -2.86630 0.93407 0.42265
C -2.76248 1.40236 2.22125
C -2.71649 2.56162 -0.46311
C -4.65560 0.48287 0.19654
H 0.41012 -0.70993 -2.98871
H 0.11149 -0.07143 0.64402
H -3.45160 3.27923 -0.06285
H -2.90062 2.40676 -1.53720
H -1.69997 2.96193 -0.32793
H -5.30296 1.30630 0.54061
H -4.89054 -0.42455 0.77377
H -4.84633 0.28653 -0.86958
H -1.76079 1.80769 2.43213
H -2.91936 0.50841 2.84411
H -3.52443 2.15945 2.47064
H -1.03432 0.44101 -1.95556
H 1.93176 -1.26766 2.00674
H 3.91574 -2.68402 2.53710
H 5.67018 -3.08403 0.80200
H 5.44446 -2.06509 -1.47001
H 3.47080 -0.64604 -2.01580
H 0.74456 2.60963 -2.10392
H 1.29776 5.01604 -1.73910
H 2.44286 5.73432 0.36537
H 3.04322 4.04154 2.10567
H 2.49771 1.63809 1.75629

TS(11’’-9’)1_{CpMe}
SCF Energy = -543.412642724
Enthalpy 0K = -543.113760
Enthalpy 298K = -543.092108
Free Energy 298K = -543.162934
SCF(DCM) = -543.470832203
SCF(BP86-D3) = -0.04558664
Lowest Frequencies = -23.9695
30.3135 cm-1
C 0.89422 -2.59833 -1.08842
C -0.37505 -2.68726 -0.35809
C -0.15168 -2.36652 1.00259
C 1.23462 -1.92360 1.10233
C 1.88447 -2.17422 -0.19593
Rh 0.27697 -0.48226 -0.10633
B -1.10630 0.69018 1.04112
P -2.64242 0.62109 -0.20383
C -3.70840 2.14211 -0.11682
H -1.31809 -3.01785 -0.79644
H -0.87435 -2.39024 1.81675
H 1.74640 -1.65554 2.02787
H 2.93688 -2.00695 -0.42244
H 1.02498 -2.80689 -2.15092
P 1.62240 1.39496 -0.02841
C 3.29006 1.14582 -0.82110
C 0.94783 2.86248 -0.95483
C 2.05723 2.06434 1.64691
C -3.79695 -0.80914 0.01749
H -1.52544 0.14601 2.04556
H -2.34097 0.56638 -1.60748
H 1.67420 3.69098 -0.91903
H 0.00465 3.18399 -0.49023
H 0.76849 2.58770 -2.00612

H 2.69515 2.95748 1.54612
H 2.60189 1.29274 2.21266
H 1.13513 2.32016 2.18808
H 3.15796 0.79234 -1.85506
H 3.86018 0.39261 -0.25590
H 3.85563 2.09213 -0.82568
H -0.89064 1.87839 1.19100
H -4.61028 -0.77172 -0.72259
H -3.24337 -1.75260 -0.08887
H -4.21647 -0.75654 1.03404
H -4.06771 2.25081 0.91817
H -3.10164 3.02255 -0.37493
H -4.56242 2.05993 -0.80652

TS(11’’-9’)1_{CpCy}
SCF Energy = -934.105699437
Enthalpy 0K = -933.570368
Enthalpy 298K = -933.539528
Free Energy 298K = -933.630596
SCF(DCM) = -934.155434764
SCF(BP86-D3) = -0.09523345
Lowest Frequencies = -136.6890
24.0412 cm-1
C -0.54023 2.98643 -0.97420
C 0.82446 2.47656 -0.47648
C 1.14838 2.99945 0.94162
C 1.08085 4.54194 0.98470
C -0.28188 5.06161 0.49529
C -0.60725 4.52924 -0.91256
P 1.00642 0.59442 -0.59310
C 2.80724 0.20919 -0.16806
C 3.44814 -0.70107 -1.24017
C 4.93809 -0.94886 -0.91780
C 5.13216 -1.51076 0.50259
C 4.48527 -0.59637 1.55931
C 2.99011 -0.35264 1.26217
Rh -1.07930 -1.07499 -0.30410
B 0.10447 -0.37629 -1.98030
C -0.27685 -3.18865 -0.92829
C 0.72014 -3.26949 0.16284
C 0.07851 -3.08224 1.36785
C -1.33405 -2.82661 1.07363
C -1.57982 -3.13731 -0.33380
H 1.78980 -3.41847 0.00566
H -0.07240 -3.42764 -1.97245
H -2.52612 -3.39852 -0.80887
H -2.10986 -2.77325 1.83983
H 0.52983 -3.04120 2.35980
P -3.09542 -0.13509 0.32162
C -4.45761 -1.33187 0.74427
C -3.14034 1.03206 1.77412
C -3.85191 0.83389 -1.07441
H 0.61924 -0.83729 -2.95581
H 0.09091 0.13793 0.56625
H -4.81461 1.26819 -0.75772
H -4.01482 0.15844 -1.92800
H -3.16823 1.63687 -1.38467
H -5.39789 -0.77706 0.89540
H -4.21852 -1.88118 1.66691
H -4.59024 -2.04909 -0.07956
H -2.46994 1.88539 1.58929
H -2.79099 0.49946 2.67235
H -4.16472 1.40359 1.94386
H -1.10866 -0.06727 -2.01871
H 3.29324 1.20526 -0.21863
H 2.90832 -1.66654 -1.27101
H 3.34334 -0.25155 -2.24347
H 5.36477 -1.63414 -1.67084
H 5.49177 0.00547 -1.01449
H 4.68141 -2.52120 0.56472
H 6.20744 -1.63771 0.71679
H 4.59220 -1.02970 2.56925
H 5.01503 0.37623 1.57826
H 2.43135 -1.30268 1.34831
H 2.55809 0.33851 2.00860
H 1.60933 2.82421 -1.18019
H 0.41364 2.57785 1.65655
H 2.14584 2.65978 1.27256
H 1.28495 4.88709 2.01328
H 1.88592 4.95854 0.34891
H -1.07236 4.74176 1.20343
H -0.29265 6.16517 0.49603
H -1.60821 4.86491 -1.23617
H 0.11256 4.94849 -1.64179
H -1.33351 2.55948 -0.32929
H -0.73452 2.63077 -2.00173

INT(11’’-9’)$_{CptBu}$
SCF Energy = -779.299035241
Enthalpy 0K = -778.833314
Enthalpy 298K = -778.803671
Free Energy 298K = -778.887946
SCF(DCM) = -779.350374108
SCF(BP86-D3) = -0.09122952
Lowest Frequencies = 30.9082
54.1344 cm-1
C -0.22840 -2.72089 0.65777
C -0.87314 -2.73614 -0.63746
C -2.19318 -2.24982 -0.46642
C -2.38572 -1.94382 0.94576
C -1.17674 -2.26837 1.63524
Rh -0.78181 -0.53931 0.17654
P -2.36966 1.15864 -0.26922
C -2.20293 2.60362 -1.43636
H -0.42669 -3.05266 -1.57906
H -2.94252 -2.15945 -1.25121
H -3.31175 -1.60879 1.41298
H -0.99895 -2.17494 2.70635
H 0.77566 -3.07902 0.87497
B 0.33225 0.36192 -1.81154
P 1.46332 0.28960 -0.31674
C 2.93514 -0.98726 -0.38160
C 3.28413 -1.57996 1.00046
C 1.99575 1.97396 0.50045
C 2.69094 2.82399 -0.59361
C 0.75306 2.74076 0.98678
C 2.92087 1.76151 1.71730
C -3.96720 0.45775 -0.93555
C -2.92860 1.97714 1.30730
C 2.56886 -2.10981 -1.37399
C 4.16932 -0.25287 -0.96557
H 0.33121 -0.54984 -2.59255

H -0.45853 0.40427 1.37252
H 2.01400 3.02146 -1.44048
H 3.60425 2.35769 -0.98859
H 2.98097 3.79597 -0.15475
H 1.07202 3.74431 1.32109
H 0.26780 2.24230 1.84212
H 0.01273 2.87811 0.18601
H 3.89082 1.31559 1.45306
H 2.44443 1.13844 2.49274
H 3.13355 2.74668 2.17040
H 4.07160 -2.34424 0.87108
H 2.42440 -2.06841 1.48464
H 3.67389 -0.82623 1.69958
H 2.34112 -1.71447 -2.37552
H 1.71877 -2.71881 -1.04092
H 3.43912 -2.78409 -1.46383
H 3.95988 0.18566 -1.95502
H 4.97554 -0.99716 -1.09766
H 4.56410 0.53200 -0.30380
H -3.10344 3.22767 -1.31040
H -2.15113 2.23528 -2.47073
H -1.31180 3.21144 -1.23325
H -4.70329 1.27313 -1.02619
H -4.37508 -0.31482 -0.26835
H -3.78952 0.02563 -1.93214
H -2.11032 2.58799 1.71606
H -3.19400 1.20671 2.04674
H -3.80187 2.62091 1.11331
H -0.07464 1.43858 -2.13968

INT(11''-9')$_{CpPh}$
SCF Energy = -926.919061629
Enthalpy 0K = -926.515760
Enthalpy 298K = -926.487422
Free Energy 298K = -926.573914
SCF(DCM) = -926.968990490
SCF(BP86-D3) = -0.07720109
Lowest Frequencies = 23.3592
32.4601 cm-1
C -1.24713 2.35941 -1.26492
C -1.35301 1.92107 0.07238
C -1.86851 2.79077 1.05900
C -2.26434 4.09057 0.70616
C -2.15552 4.52499 -0.62497
C -1.64938 3.65961 -1.60964
P -0.91382 0.21177 0.58702
C -2.38206 -0.86254 0.32772
C -3.17848 -0.73304 -0.83096
C -4.29770 -1.56096 -1.00408
C -4.62795 -2.51712 -0.02867
C -3.84244 -2.64068 1.12976
C -2.72180 -1.81618 1.31375
Rh 1.10246 -0.71305 -0.21795
B 0.26700 -0.10862 2.00570
C 0.48068 -2.89525 -0.02740
C 1.78528 -2.79163 0.55955
C 2.68919 -2.36842 -0.47618
C 1.95033 -2.22492 -1.70205
C 0.57527 -2.54910 -1.42489
H -0.43436 -3.18411 0.48995
H 2.04518 -3.00428 1.59559

H 3.76240 -2.21277 -0.35883
H 2.36192 -1.94144 -2.67053
H -0.24290 -2.56212 -2.14414
P 2.64040 0.95600 0.28175
C 3.84244 1.16478 -1.12338
C 2.03854 2.68809 0.54400
C 3.74180 0.60724 1.73578
H 0.17635 -1.14358 2.60729
H 0.78775 0.34675 -1.32887
H 2.90431 3.34463 0.72923
H 1.35282 2.73029 1.40139
H 1.50289 3.02498 -0.35605
H 4.50240 1.39990 1.82729
H 4.24059 -0.36247 1.58758
H 3.13697 0.56219 2.65238
H 3.29365 1.47615 -2.02531
H 4.34839 0.20961 -1.32912
H 4.59541 1.92854 -0.86878
H 0.82186 0.84096 2.48711
H -2.93790 0.02145 -1.58678
H -4.91557 -1.45464 -1.90109
H -5.50376 -3.15852 -0.16650
H -4.10888 -3.37110 1.90002
H -2.12437 -1.89722 2.22748
H -1.95947 2.45400 2.09677
H -2.65898 4.76285 1.47420
H -2.46639 5.53860 -0.89608
H -1.56807 3.99575 -2.64809
H -0.84782 1.68909 -2.03406

INT(11''-9')$_{CpMe}$
SCF Energy = -543.453059312
Enthalpy 0K = -543.153253
Enthalpy 298K = -543.131559
Free Energy 298K = -543.200667
SCF(DCM) = -543.508822582
SCF(BP86-D3) = -0.05207579
Lowest Frequencies = 35.6607
57.6705 cm-1
C -0.16140 -2.44540 -1.16791
C -1.50454 -2.12696 -0.76391
C -1.51753 -2.03668 0.67880
C -0.19564 -2.29045 1.16500
C 0.64289 -2.53431 0.01824
Rh -0.18200 -0.38953 -0.16611
P -1.65868 1.40443 0.18988
C -1.47384 2.93565 -0.83146
H -2.36526 -2.02659 -1.42435
H -2.38977 -1.83180 1.29940
H 0.11348 -2.31287 2.20929
H 1.70259 -2.79046 0.04817
H 0.18166 -2.60299 -2.19023
P 1.91756 0.59771 0.00860
C 3.05867 -0.04606 -1.31248
B -0.68655 1.10187 1.75838
C 2.11214 2.43259 -0.18873
C 2.81912 0.22071 1.58793
C -3.44869 0.94781 0.05194
H -1.16984 0.37638 2.58259
H -0.10933 0.35870 -1.54343
H 3.18046 2.68771 -0.09621

H 1.54802 2.96609 0.58950
H 1.75856 2.73418 -1.18651
H 3.84121 0.63124 1.54307
H 2.87042 -0.86923 1.72974
H 2.27628 0.66614 2.43405
H 2.65105 0.21027 -2.30227
H 3.14540 -1.14020 -1.23868
H 4.05870 0.40393 -1.19991
H 0.21870 1.84550 2.01793
H -3.70144 0.67274 -0.98384
H -3.67312 0.11016 0.72658
H -4.05411 1.81834 0.35353
H -2.17524 3.69575 -0.45081
H -0.45029 3.32343 -0.75030
H -1.70540 2.71947 -1.88582

INT(11''-9')$_{CpCy}$
SCF Energy = -934.169686541
Enthalpy 0K = -933.629432
Enthalpy 298K = -933.598853
Free Energy 298K = -933.688234
SCF(DCM) = -934.219828829
SCF(BP86-D3) = -0.09933082
Lowest Frequencies = 25.2704
34.8725 cm-1
C 2.03463 -2.44628 -0.89920
C 2.19944 -1.17290 -0.03466
C 3.61609 -0.58218 -0.25223
C 4.69759 -1.63047 0.09707
C 4.52547 -2.92936 -0.70882
C 3.10586 -3.49887 -0.54145
P 0.82107 0.07688 -0.42203
B -0.31168 -0.22764 -1.87904
Rh -1.37582 -0.54521 0.32417
P -2.82430 0.96087 -0.69677
C -3.64762 0.39730 -2.26509
C -0.77650 -2.59003 1.19071
C -1.66394 -2.85388 0.08436
C -2.91870 -2.24815 0.38955
C -2.82111 -1.62076 1.69367
C -1.49543 -1.85042 2.19037
H 0.25042 -2.94134 1.28279
H -1.42295 -3.41396 -0.81837
H -3.81228 -2.28755 -0.23445
H -3.62548 -1.11887 2.23033
H -1.10336 -1.52570 3.15386
C 1.24509 1.85779 0.03595
C 2.21772 2.54151 -0.95728
C 2.36575 4.03955 -0.60960
C 2.81327 4.24076 0.84974
C 1.84709 3.55077 1.83075
C 1.67737 2.04964 1.50743
C -4.28107 1.29183 0.41936
C -2.26507 2.69289 -1.06243
H -0.32381 -1.30800 -2.40118
H -1.08783 0.73640 1.17311
H -3.10809 3.25142 -1.50093
H -1.42544 2.69031 -1.77042
H -1.96002 3.18031 -0.12357
H -4.40697 1.13695 -2.56791
H -4.13514 -0.57332 -2.08884
H -2.89849 0.28086 -3.05983
H -3.92271 1.68440 1.38308
H -4.84203 0.36194 0.59659
H -4.95160 2.02979 -0.05088
H -0.71557 0.73873 -2.46854
H 0.25367 2.33499 -0.08692
H 3.21190 2.06325 -0.90809
H 1.85464 2.41704 -1.99286
H 3.08424 4.50659 -1.30580
H 1.39570 4.55041 -0.77128
H 3.83092 3.82334 0.98221
H 2.88589 5.31747 1.08211
H 2.19956 3.66536 2.87087
H 0.85691 4.04633 1.77942
H 2.63801 1.53212 1.68994
H 0.93431 1.59271 2.18702
H 2.09266 -1.42898 1.03742
H 3.72472 -0.27902 -1.31124
H 3.77119 0.31851 0.36373
H 5.69470 -1.19088 -0.08122
H 4.64398 -1.85506 1.18052
H 4.71101 -2.72365 -1.78101
H 5.27797 -3.67459 -0.39776
H 2.96685 -4.39100 -1.17709
H 2.96231 -3.83359 0.50558
H 2.13858 -2.16358 -1.96351
H 1.02470 -2.87627 -0.79653

TS(11''-6)3$_{CptBu}$
SCF Energy = -779.249415041
Enthalpy 0K = -778.789151
Enthalpy 298K = -778.759263
Free Energy 298K =
SCF(DCM) = -779.299386214
SCF(BP86-D3) = -0.08289262
Lowest Frequencies = -840.0229
36.0604 cm-1
C -2.88833 -1.47154 0.82754
C -1.85465 -2.39522 0.43383
C -1.91093 -2.49020 -0.99832
C -3.08300 -1.76469 -1.46283
C -3.68436 -1.14911 -0.35460
Rh -1.35486 -0.38345 -0.35925
P -1.75892 1.75317 0.54758
C -1.60510 3.14067 -0.68067
H -1.16773 -2.91742 1.09633
H -1.26391 -3.10623 -1.62289
H -3.39898 -1.67744 -2.50290
H -4.57100 -0.51574 -0.37102
H -3.13817 -1.21120 1.85652
B 0.51679 -0.66594 -1.28176
P 1.80625 -0.01421 0.04900
C 2.73060 -1.51345 0.85399
C 3.30726 -1.05995 2.21463
C 3.02580 1.32151 -0.72982
C 2.16415 2.57152 -1.00184
C 4.16553 1.67626 0.24054
C 3.57273 0.77959 -2.06634
C -3.50244 1.98316 1.16042
C -0.76263 2.32368 2.01042
C 1.67199 -2.60757 1.10905

C 3.84040 -2.07356 -0.05770
H 0.74667 -1.44625 -2.16906
H 0.25123 0.09836 0.65750
H 1.74200 2.99619 -0.07530
H 1.34248 2.35549 -1.70696
H 2.79463 3.35115 -1.46691
H 4.75712 2.50363 -0.19318
H 4.85690 0.83415 0.40127
H 3.79142 2.01601 1.22093
H 2.76418 0.52734 -2.77307
H 4.20589 -0.11035 -1.93812
H 4.19449 1.56305 -2.53873
H 3.76823 -1.93060 2.71567
H 2.51938 -0.67026 2.88130
H 4.08577 -0.28905 2.11491
H 1.22896 -2.98554 0.17217
H 0.86462 -2.24020 1.76367
H 2.15673 -3.45992 1.61915
H 3.45822 -2.36222 -1.05084
H 4.26305 -2.98082 0.41234
H 4.67350 -1.36553 -0.19292
H -1.88779 4.09753 -0.21163
H -2.26967 2.93818 -1.53469
H -0.57006 3.20450 -1.04462
H -3.62016 3.00419 1.55899
H -3.72648 1.25887 1.95754
H -4.20683 1.83658 0.32828
H 0.31205 2.25703 1.78367
H -0.97901 1.66459 2.86575
H -1.02519 3.36193 2.27048
H -0.40003 0.27408 -1.64712

TS(11''-6)3_{CpPh}
SCF Energy = -926.852428890
Enthalpy 0K = -926.454111
Enthalpy 298K = -926.425628
Free Energy 298K =
SCF(DCM) = -926.902589913
SCF(BP86-D3) = -0.06825966
Lowest Frequencies = -686.0495
24.4978 cm-1
C -2.50865 2.34719 -0.61912
C -1.71735 1.62906 0.30448
C -1.27649 2.25797 1.48916
C -1.62222 3.59505 1.74447
C -2.40670 4.30708 0.82160
C -2.85080 3.68245 -0.35646
P -1.27822 -0.13902 -0.01872
C -2.88972 -1.04531 -0.21914
C -3.94595 -0.84571 0.69679
C -5.12146 -1.59998 0.57552
C -5.24580 -2.55776 -0.44650
C -4.19286 -2.76220 -1.35346
C -3.01064 -2.01475 -1.23820
B -0.04521 0.00186 -1.53023
Rh 1.77548 0.02238 -0.49466
P 1.97246 -1.91434 0.81455
C 1.74496 -1.70714 2.64742
C 3.16970 1.38276 0.57588
C 2.17412 2.16676 -0.11403
C 2.37620 1.94844 -1.52137

C 3.57635 1.14959 -1.69549
C 4.06584 0.80841 -0.42002
H 3.31501 1.35335 1.65637
H 1.39928 2.78217 0.34254
H 1.78961 2.39444 -2.32489
H 3.99268 0.83568 -2.65320
H 4.94713 0.20241 -0.20982
C 0.78370 -3.26850 0.36987
C 3.63367 -2.74208 0.68162
H -0.33954 0.41259 -2.62149
H 0.21270 -0.23019 0.65582
H 0.89987 -4.11939 1.06032
H 0.98490 -3.60064 -0.66014
H -0.25002 -2.89201 0.43189
H 3.64176 -3.67307 1.27234
H 4.41371 -2.06573 1.06310
H 3.84689 -2.97394 -0.37293
H 0.72751 -1.33797 2.84816
H 2.47143 -0.97179 3.02547
H 1.89578 -2.66836 3.16521
H 0.97960 -0.92148 -1.66867
H -3.85692 -0.09478 1.48894
H -5.94418 -1.43802 1.27901
H -6.16573 -3.14349 -0.53686
H -4.29251 -3.50101 -2.15491
H -2.19453 -2.17245 -1.95310
H -0.67807 1.69838 2.21618
H -1.28940 4.07588 2.66980
H -2.67815 5.34779 1.02387
H -3.46589 4.23529 -1.07324
H -2.86249 1.86223 -1.53463

TS(11''-6)3_{CpMe}
SCF Energy = -543.387067649
Enthalpy 0K = -543.092183
Enthalpy 298K = -543.070241
Free Energy 298K =
SCF(DCM) = -543.441026466
SCF(BP86-D3) = -0.04570253
Lowest Frequencies = -722.3922
22.3409 cm-1
C -2.29711 -1.92475 -0.91414
C -2.81398 -1.02534 0.03927
C -1.98756 -1.11323 1.23477
C -1.01508 -2.16004 1.03287
C -1.15389 -2.60166 -0.32919
Rh -0.49092 -0.43596 -0.26583
B 1.31726 -0.93949 -1.20027
P 2.58260 -0.17011 0.07702
C 4.25815 0.47425 -0.52065
H -3.66804 -0.36257 -0.09873
H -2.16758 -0.59261 2.17585
H -0.32053 -2.54649 1.77775
H -0.57302 -3.39036 -0.80777
H -2.66045 -2.06658 -1.93253
P -0.63307 1.88032 0.09021
C -0.35261 2.48498 1.82536
C 0.55472 2.88261 -0.92461
C -2.28983 2.59683 -0.35598
C 3.06142 -1.62000 1.14922
H 1.57475 -1.80654 -1.99344

H 1.11201 0.26281 0.64164
H 0.44021 3.95473 -0.69755
H 0.35896 2.70827 -1.99357
H 1.58614 2.57442 -0.69031
H -2.27521 3.69185 -0.22883
H -3.06466 2.16769 0.29723
H -2.52679 2.35141 -1.40234
H 0.65638 2.19323 2.15513
H -1.09233 2.02679 2.49959
H -0.45084 3.58193 1.86966
H 0.33594 -0.12402 -1.72728
H 3.71048 -1.26285 1.96406
H 2.15634 -2.07081 1.58064
H 3.60134 -2.37569 0.55787
H 4.85406 0.80650 0.34348
H 4.78348 -0.33929 -1.04819
H 4.10628 1.31875 -1.20853

TS(11''-6) 3_{CpCy}
SCF Energy = -934.109409085
Enthalpy 0K = -933.574982
Enthalpy 298K = -933.544069
Free Energy 298K =
SCF(DCM) = -934.158699590
SCF(BP86-D3) = -0.08924740
Lowest Frequencies = -666.7399 28.8019 cm-1
C -2.30151 2.46624 -0.85611
C -1.57843 1.77223 0.32038
C -2.40026 1.86313 1.62830
C -2.74198 3.33157 1.96300
C -3.46901 4.02321 0.79534
C -2.64967 3.93002 -0.50452
P -1.08265 -0.02867 -0.03800
C -2.73746 -0.98221 -0.34029
C -3.13250 -1.82699 0.88993
C -4.45751 -2.57898 0.63684
C -4.38248 -3.44076 -0.63712
C -3.97776 -2.59689 -1.86005
C -2.65082 -1.84374 -1.61729
B 0.19246 0.13459 -1.51351
Rh 2.00941 0.13066 -0.44895
P 2.20202 -1.78213 0.88846
C 3.89109 -2.56155 0.84184
C 4.32097 0.85099 -0.43702
C 3.48077 1.43190 0.60106
C 2.48786 2.26226 -0.03655
C 2.62961 2.06764 -1.45407
C 3.79294 1.23066 -1.68655
H 5.18759 0.21075 -0.27301
H 3.66264 1.37891 1.67491
H 1.77330 2.91395 0.46469
H 2.03556 2.55258 -2.22884
H 4.16152 0.92112 -2.66502
C 1.88428 -1.56024 2.70562
C 1.07665 -3.17312 0.40120
H -0.03372 0.55718 -2.61752
H 0.44353 -0.11675 0.62886
H 1.17744 -4.01424 1.10579
H 1.33454 -3.50829 -0.61505
H 0.03507 -2.81372 0.40929
H 3.89812 -3.48539 1.44360
H 4.63405 -1.85965 1.25043
H 4.15760 -2.79840 -0.19933
H 0.85552 -1.19764 2.85291
H 2.58548 -0.81418 3.11026
H 2.02037 -2.51487 3.23949
H 1.20391 -0.80763 -1.61819
H -3.49268 -0.18537 -0.49114
H -2.33071 -2.56459 1.09640
H -3.21501 -1.19328 1.79098
H -4.70235 -3.20171 1.51548
H -5.27847 -1.84230 0.53657
H -3.64008 -4.25006 -0.48865
H -5.35153 -3.93682 -0.82002
H -3.88177 -3.23193 -2.75858
H -4.77524 -1.86069 -2.08032
H -1.83161 -2.58338 -1.50622
H -2.39906 -1.21752 -2.49189
H -0.60482 2.27552 0.48370
H -3.34391 1.29506 1.51018
H -1.84902 1.39580 2.46468
H -3.35612 3.36584 2.88020
H -1.80615 3.87904 2.19197
H -4.45530 3.54240 0.64206
H -3.67238 5.07934 1.04417
H -3.19908 4.38885 -1.34536
H -1.71181 4.50927 -0.39006
H -3.23888 1.92367 -1.08838
H -1.68167 2.42618 -1.76932

6_{CptBu}
SCF Energy = -779.306922868
Enthalpy 0K = -778.840665
Enthalpy 298K = -778.811237
Free Energy 298K = -778.895683
SCF(DCM) = -779.359296311
SCF(BP86-D3) = -0.09014571
Lowest Frequencies = 18.3694 50.0929 cm-1
C 0.95118 -1.80664 1.80219
C 2.25679 -1.69822 1.21536
C 2.26688 -2.40652 -0.04620
C 0.95611 -2.94662 -0.24159
C 0.14793 -2.58131 0.88587
Rh 0.80876 -0.63075 -0.11492
P 2.35642 1.14792 -0.22488
C 2.17073 2.56243 -1.42040
H 3.11495 -1.20987 1.67839
H 3.12210 -2.53623 -0.70827
H 0.62560 -3.52604 -1.10411
H -0.88523 -2.87868 1.04903
H 0.64399 -1.42986 2.77666
B -0.51323 0.37892 -1.95712
P -1.42777 0.29327 -0.27367
C -1.91906 1.97867 0.57019
C -0.64184 2.62076 1.13635
C -2.93772 -0.94383 -0.34383
C -2.58402 -2.13164 -1.26556
C -3.37417 -1.47096 1.04151
C -4.11096 -0.17974 -1.01030
C 2.85361 1.97317 1.37049

```
C   3.98650  0.46621 -0.82916
C  -2.52493  2.90715 -0.50945
C  -2.90481  1.78683  1.74149
H  -0.12134  1.47321 -2.26564
H   0.69276 -0.32451 -1.77211
H  -1.74472 -2.73642 -0.89453
H  -2.34490 -1.80540 -2.28839
H  -3.46667 -2.79361 -1.32018
H  -4.17072 -2.22329  0.89921
H  -3.78477 -0.68515  1.69052
H  -2.55597 -1.96146  1.59226
H  -3.83322  0.21485 -2.00144
H  -4.49468  0.64679 -0.39359
H  -4.94650 -0.88862 -1.15601
H  -0.89997  3.60108  1.57651
H   0.10611  2.79991  0.35134
H  -0.18982  1.99683  1.92417
H  -3.45598  2.50919 -0.93987
H  -1.81842  3.08844 -1.33497
H  -2.76499  3.88151 -0.04529
H  -3.89163  1.42232  1.42079
H  -3.06717  2.76918  2.22096
H  -2.50752  1.10617  2.51242
H   3.05098  3.21623 -1.30607
H   1.25727  3.14609 -1.24586
H   2.13956  2.16362 -2.44499
H   3.72713  2.62081  1.18922
H   3.11942  1.21128  2.11889
H   2.02685  2.57970  1.76440
H   3.83657 -0.00081 -1.81457
H   4.38054 -0.28743 -0.13196
H   4.72007  1.28382 -0.92214
H  -0.93090 -0.35109 -2.82152
```

6_{CpPh}

```
SCF Energy = -926.928422361
Enthalpy 0K = -926.523779
Enthalpy 298K = -926.496025
Free Energy 298K = -926.580035
SCF(DCM) = -926.979867055
SCF(BP86-D3) = -0.07700940
Lowest Frequencies = 32.8435
43.5042 cm-1
C  -1.39996  2.90785 -1.18127
C  -1.11153  1.96544 -0.16909
C  -1.04299  2.38387  1.17874
C  -1.26297  3.73081  1.50897
C  -1.54471  4.66607  0.49837
C  -1.61239  4.25457 -0.84285
P  -0.88553  0.20133 -0.63992
C  -2.44889 -0.68349 -0.22905
C  -3.26985 -0.26652  0.84111
C  -4.46051 -0.95456  1.12099
C  -4.84058 -2.05863  0.34112
C  -4.03044 -2.47107 -0.73091
C  -2.83941 -1.78907 -1.01973
Rh  1.10996 -0.96182 -0.18148
B   0.01196 -0.25156 -2.27224
C   2.07267 -2.04919  1.52162
C   2.48355 -2.72702  0.31056
C   1.29899 -3.24605 -0.30800
```

```
C   0.16776 -2.90830  0.50975
C   0.64711 -2.17400  1.65726
H   2.73744 -1.57299  2.24334
H   3.50351 -2.84216 -0.05371
H   1.25837 -3.78302 -1.25648
H  -0.87107 -3.16748  0.30717
H   0.04346 -1.80749  2.48666
P   2.53509  0.87654 -0.02183
C   4.31057  0.36136 -0.23832
C   2.36735  2.24530 -1.25931
C   2.54309  1.75658  1.61397
H   0.44237  0.70310 -2.86970
H   1.26299 -0.73696 -1.85859
H   3.14818  2.99810 -1.06341
H   1.37590  2.71426 -1.17960
H   2.49164  1.83935 -2.27412
H   3.32325  2.53573  1.61722
H   2.74095  1.03949  2.42517
H   1.56012  2.22253  1.77615
H   4.42953 -0.12613 -1.21798
H   4.59936 -0.34831  0.55179
H   4.96865  1.24440 -0.18643
H  -0.45830 -1.17708 -2.88232
H  -2.99631  0.60522  1.44249
H  -5.09489 -0.62027  1.94775
H  -5.77213 -2.58943  0.55983
H  -4.33324 -3.31723 -1.35560
H  -2.22582 -2.09639 -1.87204
H  -0.82063  1.65622  1.96723
H  -1.21537  4.04972  2.55499
H  -1.71392  5.71571  0.75739
H  -1.83591  4.98077 -1.63049
H  -1.46078  2.58826 -2.22637
```

6_{CpMe}

```
SCF Energy = -543.461710266
Enthalpy 0K = -543.161595
Enthalpy 298K = -543.140118
Free Energy 298K = -543.208892
SCF(DCM) = -543.519295299
SCF(BP86-D3) = -0.05091069
Lowest Frequencies = 42.3511
48.5364 cm-1
C  -1.46914 -1.62444  1.27225
C  -0.12497 -2.12364  1.26120
C   0.13183 -2.69882 -0.05046
C  -1.04156 -2.51907 -0.83998
C  -2.03069 -1.84074 -0.03947
Rh -0.24921 -0.45011 -0.21767
P   1.93708  0.32110  0.04772
C   2.36280  1.02426  1.71593
P  -1.31555  1.62763 -0.09098
B  -0.95787  1.20441 -1.92036
C  -0.58878  3.12468  0.72836
C  -3.06964  1.58210  0.50576
C   3.20106 -1.02834 -0.15802
C   2.53046  1.59599 -1.16206
H   0.55225 -2.14247  2.11530
H   1.04475 -3.20150 -0.36667
H  -1.16398 -2.81573 -1.88228
H  -3.04168 -1.59103 -0.36014
```

```
H  -1.97334 -1.16868  2.12402
H  -0.13003  1.90527 -2.44729
H  -0.12046  0.07095 -1.82684
H   3.56564  1.88370 -0.91677
H   1.88700  2.48675 -1.14343
H   2.49581  1.16988 -2.17625
H   3.42970  1.30011  1.74922
H   2.15753  0.27079  2.49192
H   1.74940  1.91386  1.91954
H   3.09025 -1.48689 -1.15228
H   3.06144 -1.79919  0.61494
H   4.21452 -0.60492 -0.06258
H  -1.91530  0.80776 -2.53309
H  -3.54204  2.55370  0.28586
H  -3.61475  0.79433 -0.03301
H  -3.11628  1.39679  1.58999
H  -1.19614  4.00138  0.44765
H  -0.60247  3.01516  1.82420
H   0.44081  3.29022  0.38237
```

6_{CpCy}

```
SCF Energy = -934.176911457
Enthalpy 0K = -933.636568
Enthalpy 298K = -933.606023
Free Energy 298K =  -933.695757
SCF(DCM) = -934.228570614
SCF(BP86-D3) = -0.09859793
Lowest Frequencies = 20.1534
33.2447 cm-1
C   1.25853 -2.16512  1.64783
C   2.63833 -1.92535  1.32798
C   2.93540 -2.54447  0.05460
C   1.72612 -3.15494 -0.41391
C   0.69560 -2.92735  0.55684
Rh  1.38233 -0.88355 -0.20768
P   2.71303  1.02932 -0.12515
C   2.99497  1.75612  1.56428
P  -0.78828  0.03723 -0.53993
B   0.11101 -0.18836 -2.21739
C  -1.50611  1.68644  0.07565
C  -2.18175 -1.21623 -0.31030
C   4.44226  0.64019 -0.69515
C   2.29758  2.48664 -1.19518
H   3.35472 -1.41247  1.97050
H   3.90442 -2.57459 -0.44188
H   1.60251 -3.68336 -1.35997
H  -0.32437 -3.30344  0.49973
H   0.75138 -1.87417  2.56672
H   0.44414  0.81723 -2.79291
H   1.41122 -0.60847 -1.87708
H   3.09028  3.24537 -1.08941
H   1.33257  2.92557 -0.90538
H   2.23579  2.15924 -2.24342
H   3.61526  2.66411  1.48613
H   3.51537  1.02104  2.19708
H   2.03144  2.00296  2.03111
H   4.40719  0.26657 -1.72991
H   4.88997 -0.13186 -0.05153
H   5.06548  1.54883 -0.65500
H  -0.28750 -1.13916 -2.84450
C  -3.39757 -0.84303 -1.19903
H  -1.74391 -2.14772 -0.71804
C  -4.50386 -1.91642 -1.08624
H  -3.81289  0.13190 -0.87673
H  -3.07983 -0.72879 -2.25019
C  -4.92796 -2.14551  0.37526
H  -5.36944 -1.61136 -1.70017
H  -4.13260 -2.86609 -1.51861
C  -3.71851 -2.50936  1.25599
H  -5.40266 -1.22533  0.76916
H  -5.69231 -2.93983  0.43164
C  -2.60565 -1.44251  1.15728
H  -4.02484 -2.62783  2.31024
H  -3.31329 -3.48974  0.93475
H  -2.98410 -0.49260  1.58398
H  -1.73500 -1.73217  1.77330
C  -1.75326  2.68299 -1.08224
H  -2.49444  1.36279  0.45896
C  -2.49108  3.93910 -0.56696
H  -0.78698  2.97691 -1.53400
H  -2.33432  2.20102 -1.88787
C  -1.75248  4.59737  0.61204
H  -2.61675  4.65541 -1.39785
H  -3.51160  3.65204 -0.24579
C  -1.51474  3.59196  1.75415
H  -0.77771  4.99203  0.26173
H  -2.32374  5.46623  0.98213
C  -0.75776  2.34203  1.25359
H  -0.94987  4.06284  2.57824
H  -2.48809  3.28004  2.18057
H   0.25066  2.65106  0.92145
H  -0.61586  1.61341  2.07233
```
